# Designing New Magnesium Pincer Complexes for Catalytic Hydrogenation of Imines and *N*-Heteroarenes: H_2_ and N–H Activation by Metal–Ligand Cooperation as Key Steps

**DOI:** 10.1021/jacs.3c01091

**Published:** 2023-04-17

**Authors:** Yaoyu Liang, Jie Luo, Yael Diskin-Posner, David Milstein

**Affiliations:** † Department of Molecular Chemistry and Materials Science, Weizmann Institute of Science, Rehovot 7610001, Israel; ‡ Department of Chemical Research Support, Weizmann Institute of Science, Rehovot 7610001, Israel

## Abstract

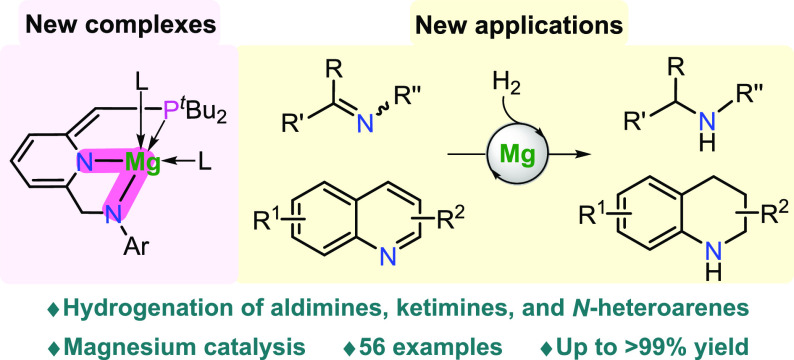

Utilization of main-group metals as alternatives to transition metals in homogeneous catalysis has become a hot research area in recent years. However, their application in catalytic hydrogenation is less common due to the difficulty in heterolytic cleavage of the H–H bond. Employing aromatization/de-aromatization metal–ligand cooperation (MLC) highly enhances the H_2_ activation process, offering an efficient approach for the hydrogenation of unsaturated molecules catalyzed by main-group metals. Herein, we report a series of new magnesium pincer complexes prepared using PNNH-type pincer ligands. The complexes were characterized by NMR and X-ray single-crystal diffraction. Reversible activation of H_2_ and N–H bonds by MLC employing these pincer complexes was developed. Using the new magnesium complexes, homogeneously catalyzed hydrogenation of aldimines and ketimines was achieved, affording secondary amines in excellent yields. Control experiments and DFT studies reveal that a pathway involving MLC is favorable for the hydrogenation reactions. Moreover, the efficient catalysis was extended to the selective hydrogenation of quinolines and other *N*-heteroarenes, presenting the first example of hydrogenation of *N*-heteroarenes homogeneously catalyzed by early main-group metal complexes. This study provides a new strategy for hydrogenation of C=N bonds catalyzed by magnesium compounds and enriches the research of main-group metal catalysis.

## Introduction

**Figure 1:**
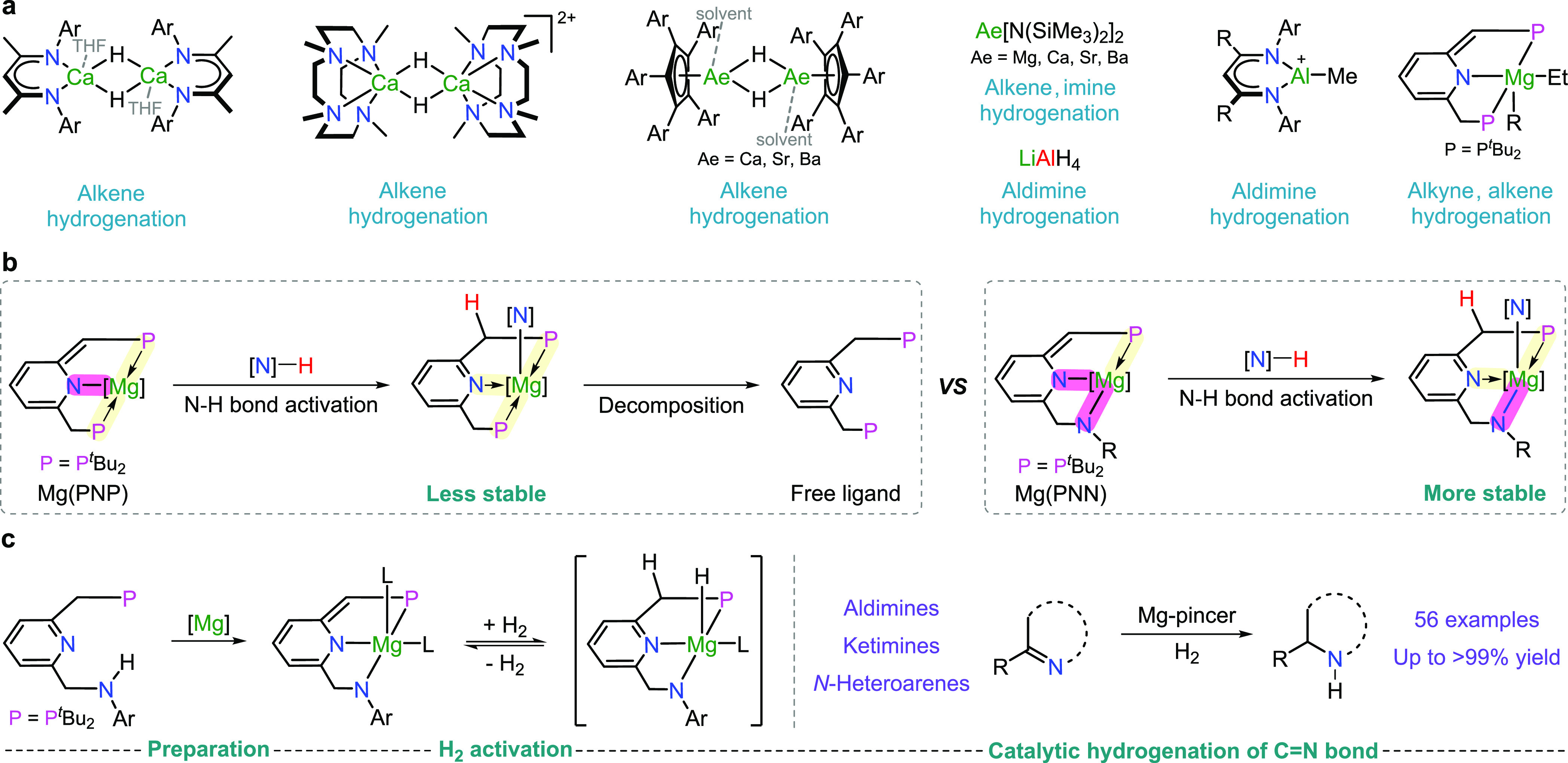
Overview of this work. (a) Representative main-group metal complexes for hydrogenation of unsaturated molecules. (b) Comparison of the Mg(PNP) complex with the new Mg(PNN) complex. (c) This work, preparation of new magnesium pincer complexes, and their application in reversible activation of H_2_ and catalytic hydrogenation of the C=N bond.

Catalytic hydrogenation of unsaturated molecules by H_2_ is one of the most practical transformations in laboratory and industry.^1^ Classic catalysts mainly rely on late transition metals due to their efficiency in the cleavage of H_2_.^1,2^ Due to the toxic and costly characteristics, it is increasingly interesting to use cheaper and more environmentally benign catalysts, such as the first-row transition metal compounds and frustrated Lewis pairs (FLPs).^3−5^ However, as another large group of elements in the periodic table, the main-group metals have received much less attention in catalytic hydrogenation due to their lack of partially filled d orbitals and the instability of many main-group metal compounds, which largely impeded their application in bond activation.^6−12^ In the 1960s, simple metal hydrides and inorganic bases were known as catalysts for the hydrogenation of benzophenone, alkenes, and alkynes, but harsh conditions were required.^7^ In 2008, Harder and co-workers reported a β-diketiminate calcium hydride complex and K, Ca, and Sr benzyl catalysts, providing relatively mild conditions for the catalytic hydrogenation of conjugated alkenes (Figure 1a).^8a^ Subsequently, Okuda’s group developed the hydrogenation of non-activated alkenes catalyzed by cationic calcium hydride complexes.^8b,8c^ Cheng et al. reported the bulky penta-arylcyclopentadienyl ligand-supported heavy alkaline earth metals (Ca, Sr, and Ba) hydrides that exhibited activity in the hydrogenation of various alkenes.^8e−8g^ Recently, group 1 and group 2 metal amides have been prepared for the catalytic hydrogenation of challenging alkenes and extended to imine hydrogenation.^9^ In addition, cationic β-diketiminate aluminum complexes were found to be efficient catalysts in the hydrogenation of imines.^10^ Several heterobimetallic systems also exhibited capability in activation of H_2_ and efficiency in hydrogenation of alkenes and imines.^11^ Very recently, magnesium pincer complex-catalyzed semihydrogenation of alkynes and hydrogenation of alkenes were reported by our group.^12^ Despite continuous breakthroughs in the main-group metal-catalyzed hydrogenation, it is not on par with the classical transition metal catalysts. Thus, enriching the main-group metal catalysis (especially by the abundant early main-group metals) to broaden their application in hydrogenation is still highly necessary.

In the specific area of hydrogenation, the reduction of imines by H_2_ offers an atom-economic approach to access amines (linear and cyclic).^13^ Although various catalysts based on FLPs^5d^ and sustainable metals^4,9b,9c,10,11a,11c,11d,14,15^ have been developed to avoid using expensive and toxic metals, examples of using early main-group metals are scarce.^9b,9c,11d^ In 2018, Harder’s group reported the first successful example of hydrogenation of imines catalyzed by alkaline earth and alkali metal amides via a metal hydride mechanism.^9b^ While this system is active with aldimines and inefficient with ketimine substrates, further studies by the same group and by Pfaltz et al. reported that substoichiometric amounts of LiHMDS and 100 bar of H_2_ were required to ensure high yield.^9c^ Magnesium, in these cases, exhibited lower catalytic activity than heavy alkaline earth metals.

In recent years, magnesium compounds have emerged as redox-neutral catalysts for a variety of reactions^6,16−20^ such as hydroamination,^17^ hydrosilylation,^18^ hydroboration,^19^ and dehydrocoupling.^20^ However, the application in hydrogenation is still challenging because of its lower efficiency in activating the H–H bond.^9b,11d,12^ Metal–ligand cooperation (MLC) involving aromatization/de-aromatization of pincer metal complexes offers a versatile tactic for activating small molecules.^2d,21^ Recently, we developed a series of de-aromatized (with a deprotonated side arm) magnesium pincer complexes supported by PNP-, PNN-, and NNN-type ligands.^12^ We showed that a Mg(PNP) complex is capable of reversible heterolysis of H_2_ via an MLC process and catalyzes the hydrogenation of unsaturated C–C bonds, providing a new strategy for enhancing the catalytic activity of magnesium compounds. Nevertheless, the de-aromatized complex is incompatible with compounds that contain active protons, such as amines, since the derived aromatized (with fully protonated side arms) intermediate from the N–H bond activation is unstable due to the weakly coordinating phosphines of the (PNP)^*t*^Bu ligand and the magnesium center (Figure 1b, left). To meet the requirement of C=N bond hydrogenation, we speculated that using PNNH-type ligands can construct a more stable Mg(PNN) complex that may be compatible with amine products since the aromatized intermediate from the N–H bond activation would bear a stronger covalent Mg–N bond, which can stabilize the intermediate (Figure 1b, right).

Herein, we report the preparation of a series of new magnesium pincer complexes using PNNH-type ligands (Figure 1c, left). The amine proton of the PNNH ligand was replaced by magnesium, forming a new Mg–N covalent bond, further stabilizing the complexes. The activation of H–H and N–H bonds via the MLC process was studied. Catalytic hydrogenation of various aldimines and ketimines was proved feasible, generating secondary amines in up to >99% yields (Figure 1c, right). A plausible mechanism involving an MLC process is proposed on the basis of control experiments and DFT studies. The developed catalytic system is also efficient in the selective hydrogenation of a variety of *N*-heteroarenes, generating cyclic amines in high yields.

## Results and Discussion

### Preparation and Characterization of Magnesium Pincer Complexes

**Figure 2:**
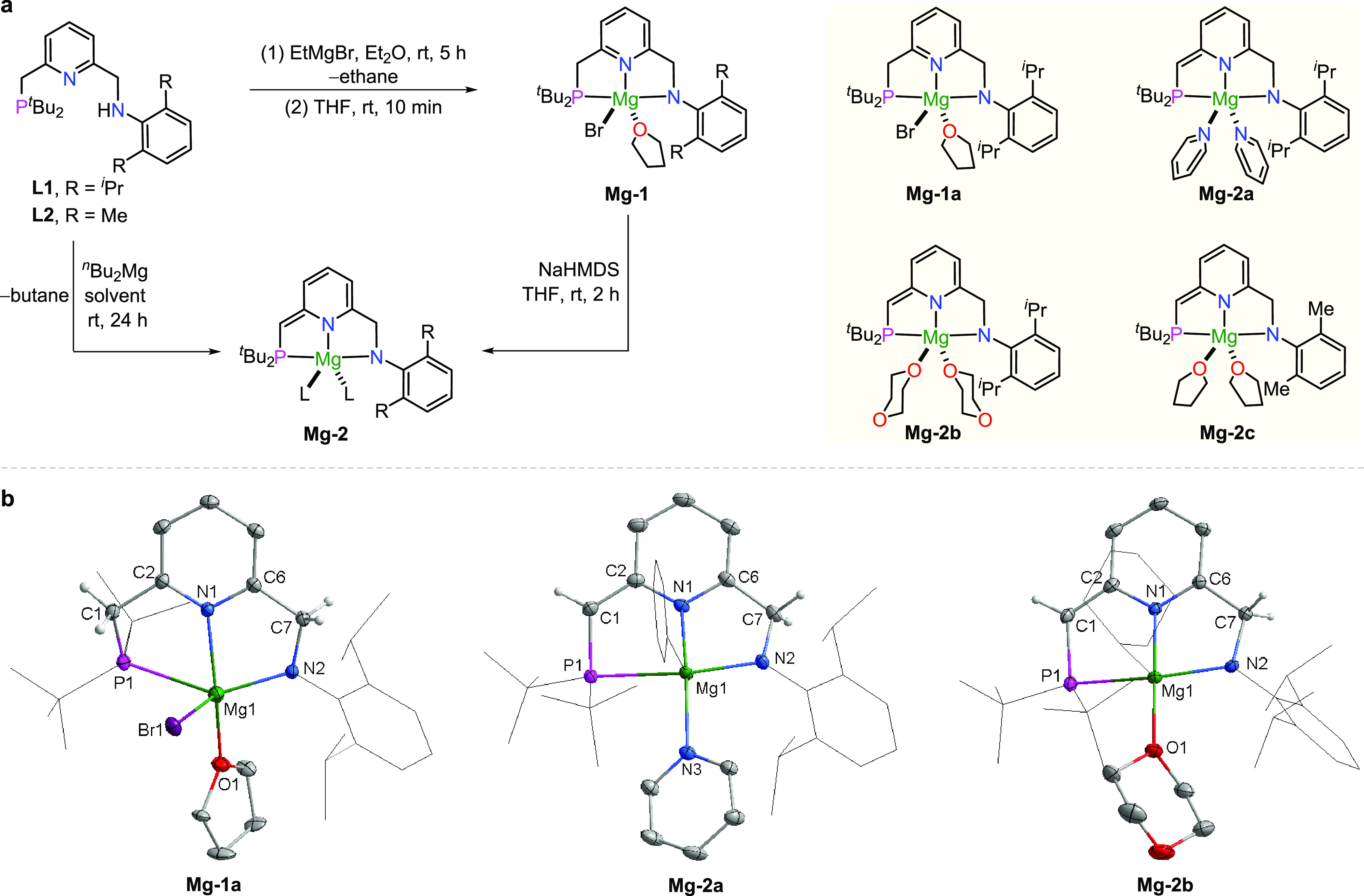
Preparation and characterization of magnesium pincer complexes. (a) Procedures for preparing magnesium pincer complexes and the obtained complexes. (b) Crystal structures of complexes **Mg-1a** (left), **Mg-2a** (middle), and **Mg-2b** (right). Selected hydrogen atoms were omitted for clarity. Some groups were displayed as wireframes for clarity. Selected bond lengths (Å) and angles (deg): (**Mg-1a**) Mg1–P1 2.8367(6), Mg1–N1 2.1594(12), Mg1–N2 1.9939(13), Mg1–O1 2.0554(12), Br–Mg1 2.5120(5), C1–C2 1.503(2), C6–C7 1.499(2), Br1–Mg1–O1 99.83(4), Br1–Mg1–N2 122.21(4), and Br1–Mg1–P1 99.047(17). (**Mg-2a**) Mg1–P1 2.7644(5), Mg1–N1 2.1086(10), Mg1–N2 2.0129(10), C1–C2 1.3857(16), C6–C7 1.5052(16), and N3–Mg1–N4 94.25(4). (**Mg-2b**) Mg1–P1 2.7335(4), Mg1–N1 2.0700(9), Mg1–N2 2.0151(9), C1–C2 1.3967(15), C6–C7 1.5155(14), and O1–Mg1–O3 90.91(3).

The aromatized magnesium bromide complex **Mg-1a** was prepared by the reaction of the PNNH-type ligand (**L1**) with EtMgBr in Et_2_O (Figure 2a, left). A yellowish precipitate was formed after stirring the solution at room temperature for 5 h. The solid is soluble in THF, generating complex **Mg-1a** with a coordinated THF molecule. The ^31^P{^1^H} NMR spectrum of **Mg-1a** exhibits a singlet at 22.89 ppm, suggesting formation of an aromatized structure, in line with the reported chemical shifts in previous studies.^12,22^ Two methylene (C*H*_2_) groups of the phosphine and amine side arms were observed by ^1^H NMR, supporting the aromatized structure. Single crystals of **Mg-1a** suitable for X-ray diffraction studies were grown at room temperature. X-ray crystallography reveals that **Mg-1a** is formed as a five-coordinated complex, in line with many reported examples (Figure 2b, left).^12,18c,23^ The bromo group and the THF ligand formed at a 99.8° angle (Br1–Mg1–O1). The bond length of Mg1–N2 (1.99 Å) is considerably shorter than that of Mg1–N1 (2.16 Å), suggesting the formation of a Mg–N covalent bond between Mg1 and N2, which differed from the reported transition-metal complexes prepared by PNNH-type ligands that possessed a free amine proton.^24^ It is noted that an analogous aromatized structure was not observed in previous PNP magnesium complexes, confirming that the covalent bond between Mg1 and N2 is crucial for stabilizing the aromatized structure.

Next, an equivalent amount of base (NaHMDS) was added to the THF solution of **Mg-1a** in order to deprotonate the side arm and remove the bromo group. A white precipitate gradually formed during the reaction, implying the generation of sodium bromide. A singlet was observed at 3.15 ppm in the ^31^P{^1^H} NMR spectrum after the full consumption of **Mg-1a**, revealing that the deprotonation only took place at the phosphine side arm to generate a single product. Removing the solvent provided thick oil, which is soluble in pyridine or dioxane to yield the de-aromatized complexes **Mg-2a** and **Mg-2b** with pyridine and dioxane, respectively, as additional ligands (Figure 2a, right). The ^31^P{^1^H} NMR spectra of **Mg-2a** and **Mg-2b** exhibit similar singlets at 2.66 and 3.35 ppm, respectively. The chemical shifts suggest that they are de-aromatized structures. The methine (C*H*) signals of the phosphine side arms of **Mg-2a** and **Mg-2b** were observed at 3.69 (d, *J* = 5.1 Hz) and 3.16 (d, *J* = 4.9 Hz) ppm by ^1^H NMR. X-ray crystallography of **Mg-2a** shows a considerably shorter C1–C2 bond (1.39 Å) compared to the C6–C7 bond (1.51 Å), supporting its de-aromatized structure. Two pyridine ligands are coordinated to the magnesium center at a 94.3° angle, similar to that of aromatized **Mg-1a**. Likewise, a shorter C1–C2 bond (1.40 Å) compared to a C6–C7 bond (1.52 Å) can be observed in the X-ray structure of **Mg-2b**. Following similar two-step procedures using **L2** as a ligand, a de-aromatized complex **Mg-2c** was obtained with two THF ligands coordinated to the magnesium center. The ^31^P{^1^H} NMR spectrum exhibits a singlet at 4.04 ppm, similar to that of **Mg-2a** and **Mg-2b**. The ^1^H NMR spectrum exhibits a characteristic doublet at 3.55 ppm (*J* = 5.2 Hz, 1H) and a singlet at 4.43 ppm (2H), which are assigned to the methine (C*H*) and methylene (C*H*_2_) of the phosphine side arm and amine side arm, respectively, confirming the de-aromatized structure. Signals assigned to two THF ligands were also observed by ^1^H NMR, supporting the structure of **Mg-2c**. It should be mentioned that **Mg-2c** is less stable than **Mg-2a** and **Mg-2b**, possibly due to the lower steric hindrance of the anilido ligand. In addition, a one-step procedure to access the de-aromatized complexes from ligands **L1** and **L2** was developed by using ^*n*^Bu_2_Mg as a precursor. The deprotonation occurs by the generated butyl ligand, avoiding the use of an additional base and generating 2 equiv of butane as the only byproduct. Following this procedure, **Mg-2b** and **Mg-2c** were obtained at room temperature using dioxane or THF as solvents. However, a trace amount of de-aromatized product originating from deprotonation of the amine side arm was observed by NMR as well.

### Bond Activation by Magnesium Pincer Complexes

**Figure 3:**
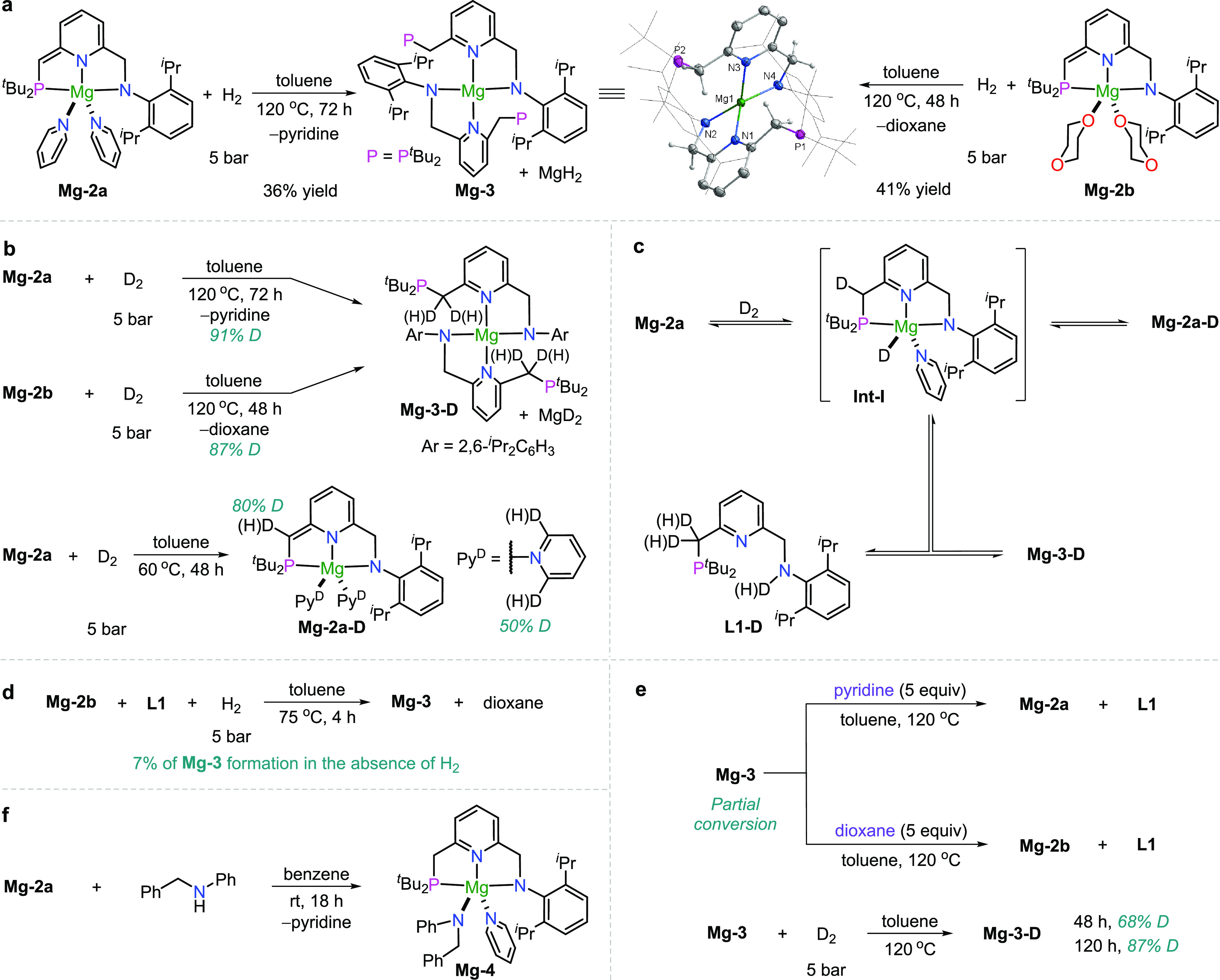
Studies of H_2_ (D_2_) and amine activation by de-aromatized complexes. (a) Formation of **Mg-3** by the reaction of **Mg-2a** and **Mg-2b** with H_2_. (b) Formation of **Mg-3-D** by the reaction of **Mg-2a** and **Mg-2b** with D_2_. (c) Proposed pathway for the reversible activation of D_2_ by **Mg-2a**. (d) Control experiments to confirm that H_2_ is required for the generation of **Mg-3**. (e) Conversion of **Mg-3** to de-aromatized complexes and the free ligand and activation of D_2_ by **Mg-3**. (f) Activation of the N–H bond by **Mg-2a**.

To test the ability of the de-aromatized complexes for H_2_ activation, a toluene solution of **Mg-2a** was treated with 5 bar of H_2_ in a J. Young NMR tube (Figure 3a, left). Heating at 120 °C, the orange solution turned green. A new species with a ^31^P{^1^H} NMR signal at 20.81 ppm was gradually formed, suggesting that an aromatized compound may be formed. After the full consumption of **Mg-2a**, the ^1^H NMR showed two characteristic methylene (C*H*_2_) signals assigned to the phosphine and amine side arms, verifying the formation of an aromatized structure. Single crystals of the new species were obtained at room temperature using a mixed solvent of THF/pentane. X-ray crystallography confirmed the structure as **Mg-3**, a four-coordinated dimer with two PNN ligands and uncoordinated phosphorous atoms. Similarly, **Mg-2b** fully converted into the dimer complex **Mg-3** under H_2_ pressure with shorter reaction time, implying its higher activity (Figure 3a, right). These results suggested that the de-aromatized complexes **Mg-2a** and **Mg-2b** may undergo activation of H_2_ by MLC, as reported in the case of a Mg(PNP) complex.^12^ To confirm that, a toluene solution of **Mg-2a** was pressurized with 5 bar of D_2_ and heated at 120 °C in a J. Young NMR tube (Figure 3b, top). After the full consumption of **Mg-2a**, **Mg-3-D** was obtained with deuterium signals on the phosphine side arm of **Mg-3**, as confirmed by ^2^H NMR (Figure S38). It was found that 91% of deuterium was incorporated into the phosphine side arm according to the integration of the ^1^H NMR (Figure S39). Likewise, the treatment of **Mg-2b** with D_2_ under similar conditions produced **Mg-3-D** with 87% of the deuterated phosphine side arm (Figure S42). These results show that the de-aromatized complexes **Mg-2a** and **Mg-2b** can reversibly activate D_2_ (H_2_). In addition, upon treatment of a toluene solution of **Mg-2a** with 5 bar of D_2_ at a lower temperature (60 °C), **Mg-2a-D** was formed with 80% deuterium incorporated into the phosphine side arm of **Mg-2a** after 48 h, further confirming the reversible activation of D_2_ (H_2_) by the de-aromatized complexes (Figure 3b, bottom). It is noteworthy that in the above results, the deuteration of the amine side arm was not observed, in line with the observation in the preparation process that the protons on the phosphine side arm undergo deprotonation more readily.

The generation of **Mg-3-D** and **Mg-2a-D** likely proceeds via the aromatized intermediate **Int-I**, which is derived from the reaction of **Mg-2a** with D_2_ (Figure 3c). **Int-I** was not observed during the reaction since it quickly underwent the elimination of DH (or D_2_) to generate **Mg-2a-D** (or **Mg-2a**) to enter the next cycle of D_2_ activation. Meanwhile, the instability of **Int-I** resulted in partial decomposition in the presence of D_2_ to produce the deuterium-labeled free ligand **L1-D**, which reacted with **Int-I** to generate the dimer complex **Mg-3-D**. Theoretically, the reaction of the ligand **L1-D** with **Mg-2a** (or **Mg-2a-D**) can produce the dimer complex **Mg-3-D** via the deuterium (proton) transfer from amine to the de-aromatized phosphine side arm without the participation of D_2_ (H_2_). To clarify this point, a toluene solution of **Mg-2b** and **L1** was heated in the presence of H_2_ (5 bar) at 75 °C, resulting in the formation of **Mg-3** after 4 h (Figure 3d). On the contrary, only about 7% of **Mg-3** can be observed in the absence of H_2_ under the same conditions. These results suggest that H_2_ is required for the formation of **Mg-3** and indirectly confirm the formation of the active intermediate **Int-I**.

The dimer complex **Mg-3** is unstable in the absence of H_2_. Upon treatment of **Mg-3** with 5 equiv of pyridine or dioxane at 120 °C, the dearomatized complexes **Mg-2a** and **Mg-2b** were gradually regenerated together with the formation of free ligand **L1** (Figure 3e, top). The decomposition of **Mg-3** originated from the deprotonation of the phosphine side arm by the anilido ligand of the complex itself. As a result, the free ligand can be observed along with the formation of de-aromatized complexes. However, the consumption of **Mg-3** is incomplete (Figures S45 and S46), even upon prolonging the reaction time to 2 days, suggesting the existence of an equilibrium between the dimer, the de-aromatized complexes, and the free ligand. To confirm that, a toluene solution of **Mg-3** was treated with D_2_ and heated at 120 °C (Figure 3e, bottom), resulting in the incorporation of 68% of deuterium into the phosphine side arm after 48 h (Figure S47), and the deuterated ratio increased to 87% after 5 days (Figure S49). This result confirms that the formation of **Mg-3** is reversible.

The activation of the N–H bond by the de-aromatized complexes was studied as well. Upon treatment of the benzene solution of **Mg-2a** with *N*-benzylaniline in a J. Young NMR tube, a singlet can be observed at 24.87 ppm in the ^31^P{^1^H} NMR spectrum after rotating the tube for 18 h at room temperature. The chemical shift of the signal implies the generation of an aromatized species (Figure 3f). In the ^1^H NMR, a doublet (*J* = 2.9 Hz) assigned to the methylene (C*H*_2_) group of the phosphine side arm was found at 2.99 ppm, confirming the aromatized structure of the generated magnesium complex. The methylene (C*H*_2_) signals of the amine side arm and the benzyl group of *N*-benzylaniline were also found at 5.06 and 4.75 ppm, respectively. The ^1^H NMR exhibits one pyridine ligand, suggesting that the secondary amine replaced a pyridine ligand to generate a new Mg–N covalent bond. The new complex was finally confirmed as **Mg-4**, which was derived from the N–H bond activation by **Mg-2a** via the MLC process.^25^ It should be mentioned that the analogous structure could not be observed by using previously reported PNP magnesium complexes (see page S53 in the SI),^12^ indicating that the current complexes are more stable.

### Catalytic Hydrogenation of Imines

**Figure 4:**
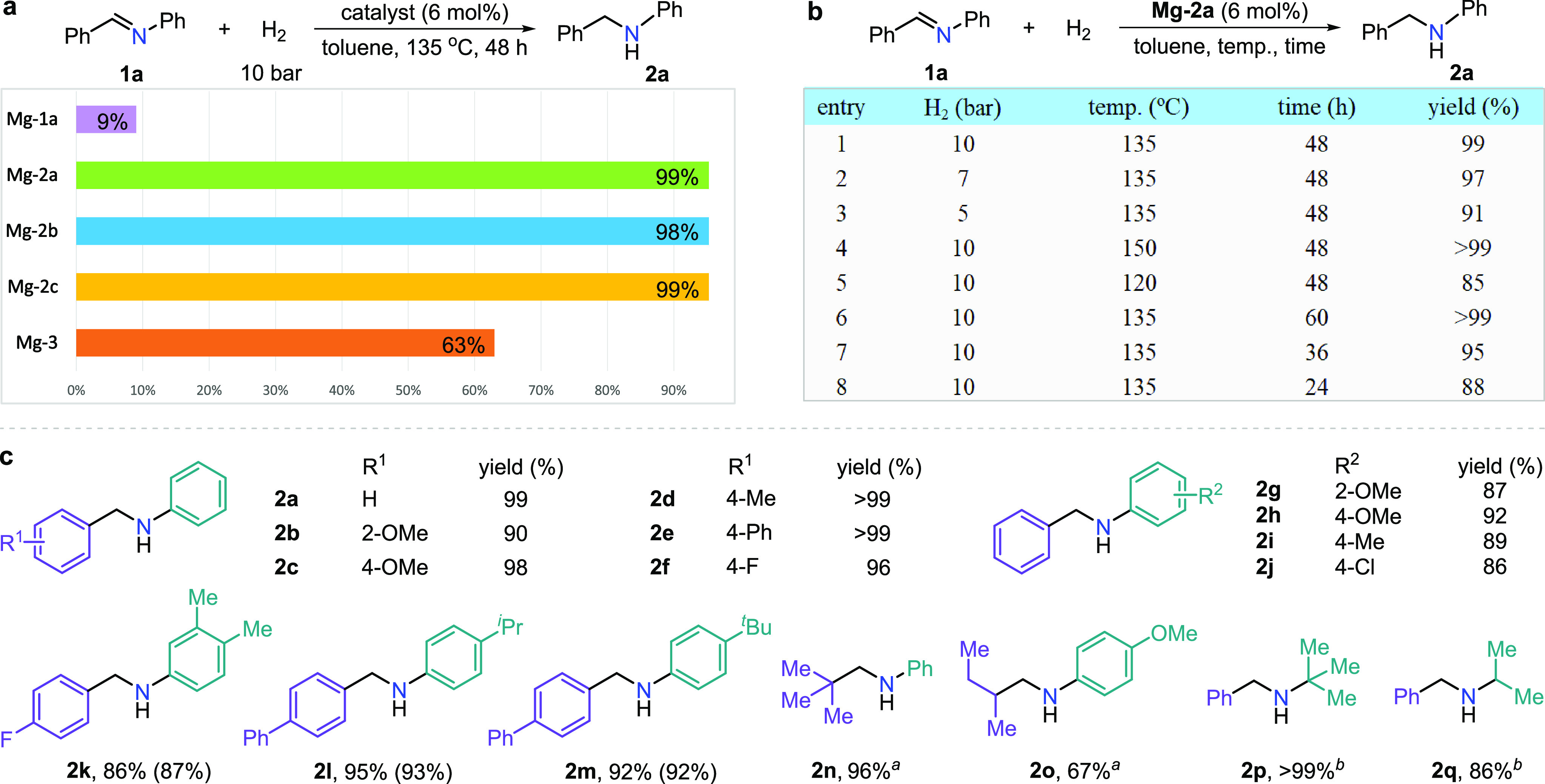
Catalytic hydrogenation of aldimines. (a) Effect of different magnesium complexes. (b) Effect of hydrogen pressure, reaction temperature, and reaction time. (c) Scope of aldimines. Reaction conditions: **1** (0.3 mmol), **Mg-2a** (6 mol %), H_2_ (10 bar), toluene (1 mL), 135 °C, 48 h. Reaction yields were determined by ^1^H NMR spectroscopy of the crude reaction mixture with respect to 1,3,5-trimethoxybenzene as an internal standard. Isolated yields are in parentheses. Superscript a signifies that 10 mol % of **Mg-2b** was used as the catalyst at 150 °C. Superscript b signifies that 6 mol % of **Mg-2b** was used as the catalyst.

As the reversible activation of H_2_ (D_2_) by **Mg-2a** and **Mg-2b** via the MLC process was shown to be feasible and activation of a secondary amine by **Mg-2a** was observed as well, we believed that hydrogenation of imines catalyzed by the new magnesium complexes might be possible. Examining this possibility, commercially available (*E*)-*N*,1-diphenylmethanimine (**1a**) was employed as a substrate to test the catalytic activity of the obtained magnesium complexes (Figure 4a). High conversion of **1a** was observed using 6 mol % of the de-aromatized **Mg-2a** as a catalyst, generating the desired amine **2a** in 99% yield after heating at 135 °C for 48 h under 10 bar of H_2_. The de-aromatized complexes **Mg-2b** and **Mg-2c** exhibited similar catalytic activity in the hydrogenation of **1a**, resulting in 98 and 99% yield of **2a**, respectively. However, the aromatic magnesium bromide complex **Mg-1a** (which is incapable of de-aromatization in the absence of base) showed much lower activity (9% yield), indicating the importance of the de-aromatized structure in H_2_ activation. As expected, the dimeric complex, **Mg-3**, can catalyze the imine hydrogenation, resulting in the desired amine **2a** in 63% yield under standard conditions. The result is in line with the observation that **Mg-3** can slowly be converted into the de-aromatized complexes, which act as the active catalysts for hydrogenation. However, the lower conversion of **1a** suggests that the formation of **Mg-3** deactivates the de-aromatized catalysts. These results also indicate that the de-aromatized complexes **Mg-2a**, **Mg-2b**, and **Mg-2c** were only partially converted into the dimer in the catalytic process. Other reaction parameters for the hydrogenation of **1a** were also explored by using **Mg-2a** as a catalyst (Figure 4b). Lower hydrogen pressures (7 and 5 bar) only slightly affected the reaction yields (97 and 91%). However, the temperature and reaction time significantly influence the reaction. Increasing the temperature to 150 °C led to full consumption of **1a**, affording **2a** in >99% yield after 48 h. Decreasing the temperature to 120 °C provided 85% yield of **2a** after 48 h. Upon prolonging the reaction time to 60 h, the imine **1a** was completely converted into the secondary amine. Reducing the reaction time to 36 and 24 h resulted in 95 and 88% yields, respectively. These results are encouraging since magnesium-catalyzed hydrogenation of *N*-aryl aldimines in high yields is still challenging.^9b,^

With the optimal conditions in hand, the scope of aldimines was explored (Figure 4c). Using **Mg-2a** as a catalyst, a variety of *N*-aryl arylaldimines bearing different substituents smoothly afforded the desired amines (**2a**-**2m**) in high yields (86 to >99%). Aromatic aldimines having substituents at the *para*-position gave higher yields than the *ortho*-OMe-substituted one (**2c**–**2f** vs **2b**). Placing the OMe group at the *ortho*-position of the *N*-aryl group also resulted in a lower yield (**2g**, 87%). Placing different substituents on both aryl rings of *N*-aryl arylaldimines did not affect the reaction, affording the desired products in 86–95% yields (**2k**-**2m**). The reaction tolerated various functional groups, such as methoxy, fluoro, and chloro, which are generally sensitive toward common magnesium compounds. Likewise, aliphatic *N*-aryl aldimines were suitable for hydrogenation, but a higher catalyst loading (10 mol %) and higher reaction temperature (150 °C) were required due to the less electrophilic C=N bond. Using **Mg-2b** as a catalyst, tertiary butyl-substituted imine **1n** successfully afforded the corresponding amine **2n** in 96% yield. Interestingly, *sec*-butyl-substituted aldimine gave only 67% yield of **2o**. Prolonging the reaction time did not efficiently improve the conversion. This result indicates that significant steric hindrance was required to ensure high yields of the aliphatic aldimines. Additionally, *N*-alkyl aldimines also gave rise to the corresponding amines in high yields. For example, the (*E*)-*N*-*tert*-butyl-1-phenylmethanimine (**1p**) with large steric hindrance efficiently generated **2p** in >99% yield catalyzed by **Mg-2b**. Replacing the tertiary butyl group with the smaller isopropyl group resulted in a lower yield (**2q**, 86%). The results of hydrogenating aliphatic aldimines and *N*-alkyl aldimines suggest that the steric hindrance of the generated amines is related to the reaction yields due to the de-aromatized complexes being more compatible with bulky amines. The above results indicate that the developed magnesium pincer complexes are efficient in the hydrogenation of various aldimines, generating much better results than previously reported magnesium catalysis.^9b,11d^

Next, the catalytic hydrogenation of ketimines by the de-aromatized magnesium complexes was explored (Table 1). It is noted that the hydrogenation of ketimines catalyzed by early main-group metal complexes is challenging due to the larger steric hindrance compared to aldimines.^9b^ Employing **Mg-2a** as a catalyst, the simple ketimine **1r** was successfully converted into the secondary amine **2r** in 96% yield. Ketimines (**1s**–**1u**) bearing different chain lengths of *N*-alkyl substituents also gave quantitative yields (97 to >99%). Steric hindrance appears to be an important factor in the reaction. For example, the *N*-aryl-substituted ketimine **1v** generated amine **2v** in a lower yield (85%), catalyzed by 10 mol % of **Mg-2b** at 150 °C. Replacing the substituents on the carbon atom of the ketimines by phenethyl and phenyl groups, the reactions were successfully performed to produce **2w** and **2x** in 98 and >99% yield, respectively. Interestingly, the bromo group and C–C double and triple bonds were not affected by the current conditions, generating the desired products **2y**–**2aa** in 92–98% isolated yields. It is worth mentioning that both *E-* and *Z*-ketimines efficiently undergo hydrogenation in this system, providing a new strategy for the hydrogenation of mixed imines of *Z* and *E* configurations.

**Table 1:**
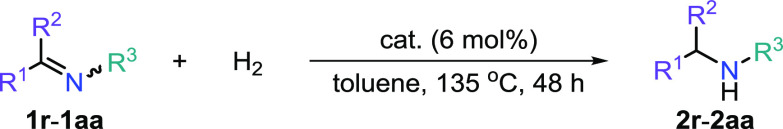

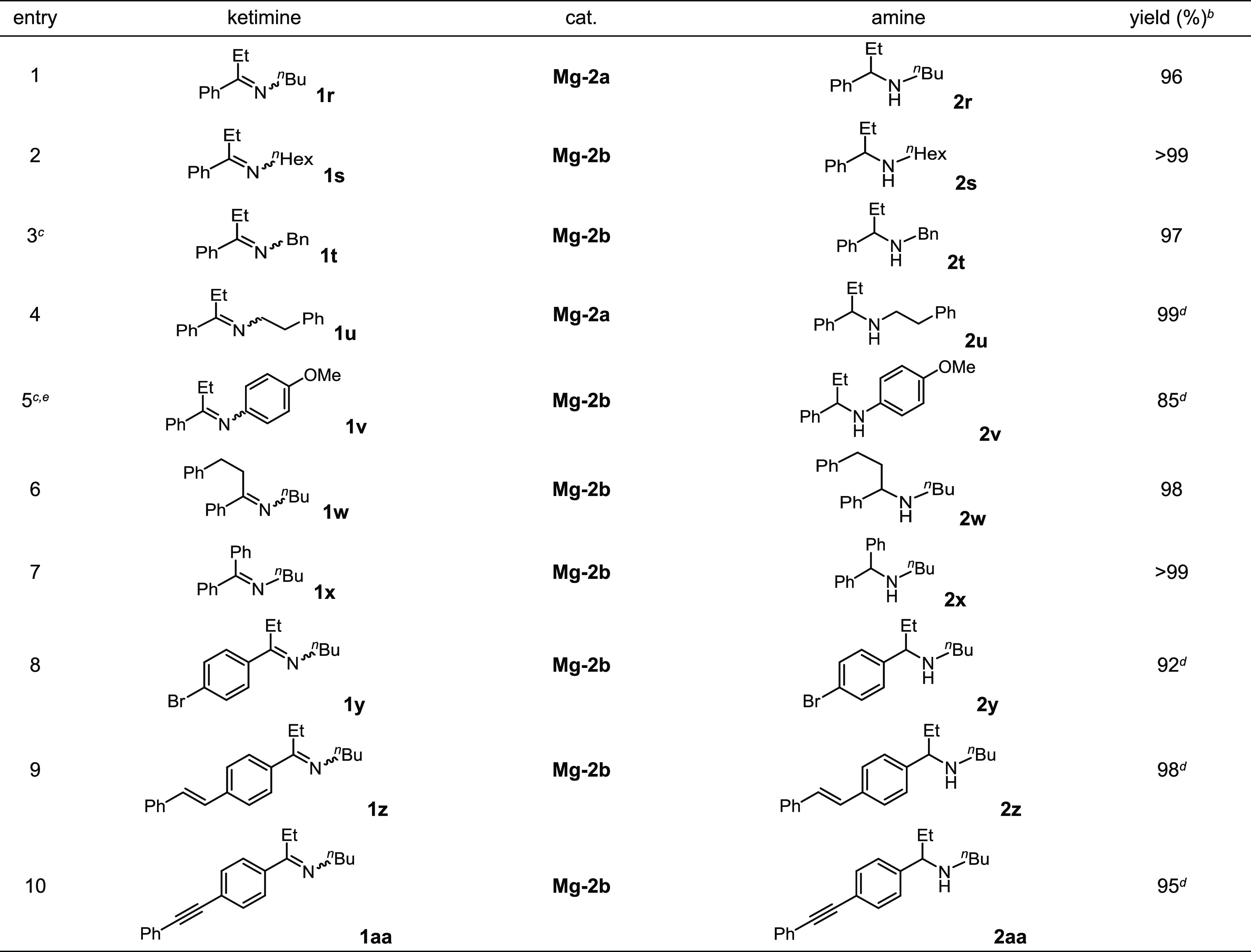
Scope of Magnesium-Catalyzed Hydrogenation of Ketimines^a^

a Conditions: **1** (0.3 mmol), cat. (6 mol %), H_2_ (10 bar), toluene (1 mL), 135 °C, 48 h.

b Yield determined by ^1^H NMR spectroscopy of the crude reaction mixture with respect to 1,3,5-trimethoxybenzene as an internal standard.

c The reaction temperature was 150 °C.

d Isolated yield.

e With 10 mol % of catalyst.

### Proposed Mechanism

**Figure 5:**
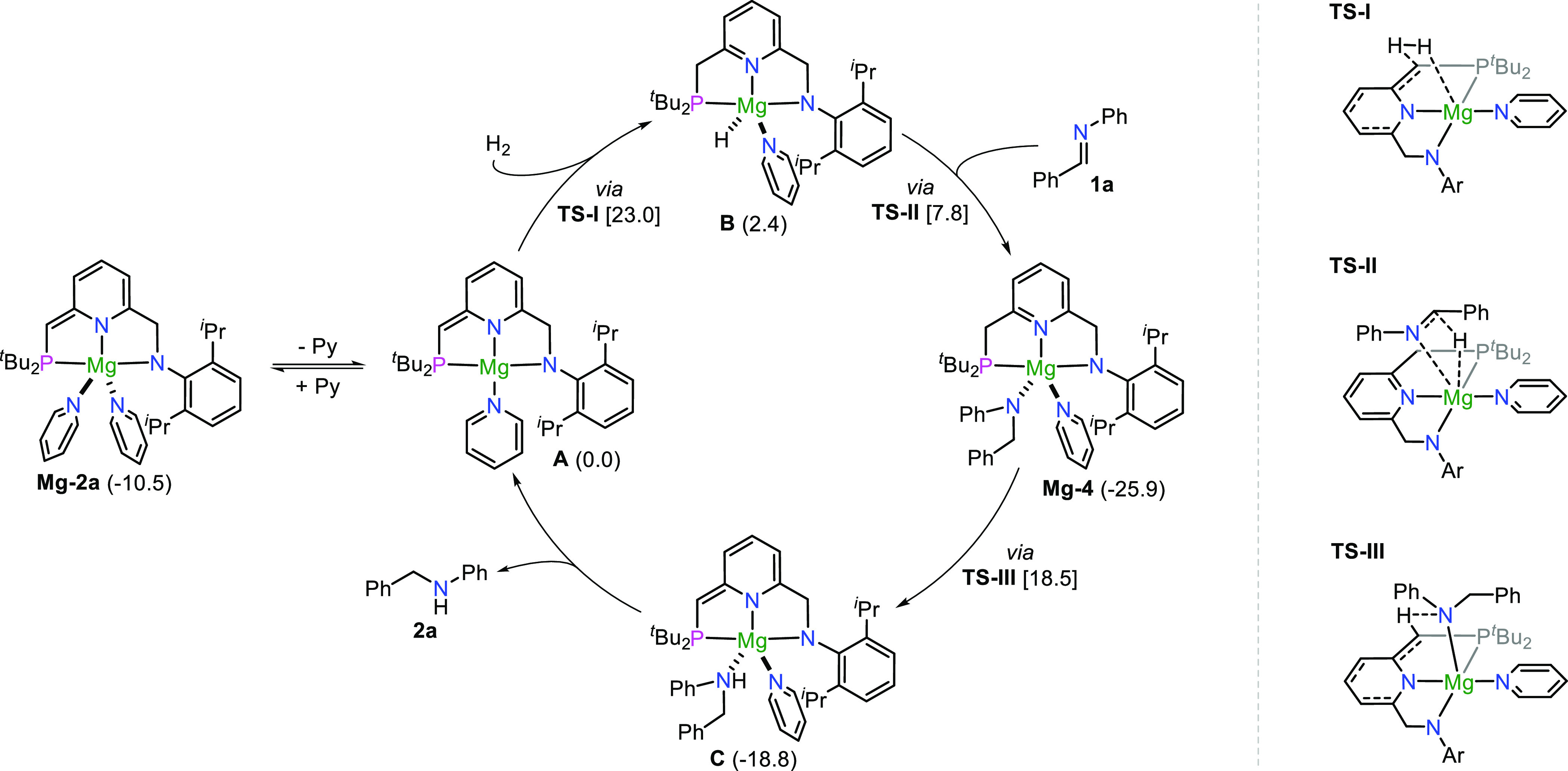
Proposed mechanism for hydrogenation of imines catalyzed by de-aromatized magnesium complexes. In parentheses, the relative free energies (in kcal/mol) of intermediates with respect to active intermediate **A** as 0.0 kcal/mol are given. In square brackets, the activation free energy barriers for the corresponding steps are given.

A plausible pathway for the hydrogenation of imines is proposed on the basis of the above experiments and supported by DFT studies (Figure 5). The reaction starts with the reversible dissociation of one pyridine ligand from **Mg-2a** to generate the four-coordinated intermediate **A**, which provides a vacant site for the activation of hydrogen. This step is an endergonic process with Δ*G* = 10.5 kcal/mol according to the DFT calculations. H_2_ activation by de-aromatized intermediate **A** generates the five-coordinated aromatized magnesium hydride intermediate **B**. The activation proceeds via transition state **TS-I**, in which the H_2_ undergoes heterolytic cleavage by the magnesium center and the de-aromatized phosphine side arm. This step has an activation free energy of 23.0 kcal/mol, and it is endergonic by 2.4 kcal/mol, suggesting the high reactivity of the unobserved intermediate **B**. It should be mentioned that the activation of H_2_ by intermediate **A′** derived from the dissociation of one dioxane from **Mg-2b** is also favorable according to the DFT studies (see page S85 in the SI). The active intermediate **B** is partially transformed into the dimeric complex **Mg-3**, as mentioned above in the control experiments (Figure 3e). Insertion of imine **1a** into the Mg–H bond of intermediate **B** produces intermediate **Mg-4**, which is relatively stable and experimentally observed in the N–H bond activation reaction of **Mg-2a** with amine **2a** (Figure 3f). It is noted that using the *in situ*-generated **Mg-4** as a catalyst for the hydrogenation of **1a**, 81% yield of **2a** was obtained under the standard conditions, suggesting that **Mg-4** is involved in the catalytic cycle. The monitoring experiments using **Mg-2a** as a catalyst further confirmed that **Mg-4** is a key intermediate in catalytic reaction (Figure S57). DFT studies also indicate that the formation of **Mg-4** is an exergonic step (Δ*G* = −28.3 kcal/mol) with a free energy barrier of 7.8 kcal/mol to overcome transition state **TS-II**. The next step is the proton migration from the phosphine side arm of intermediate **Mg-4** to the bound amine to give the de-aromatized intermediate **C** with the secondary amine coordinating to the magnesium center. This step is an endergonic process with Δ*G* = 7.1 kcal/mol and a transition state **TS-III** with a free energy barrier of 18.5 kcal/mol. Finally, the amine product **2a** is released from intermediate **C** to regenerate the four-coordinated intermediate **A** to propagate the cycle, which is endergonic (Δ*G* = 5.4 kcal/mol). The DFT studies highlight the significance of MLC in this reaction.

### Catalytic Hydrogenation of *N*-Heteroarenes

**Figure 6:**
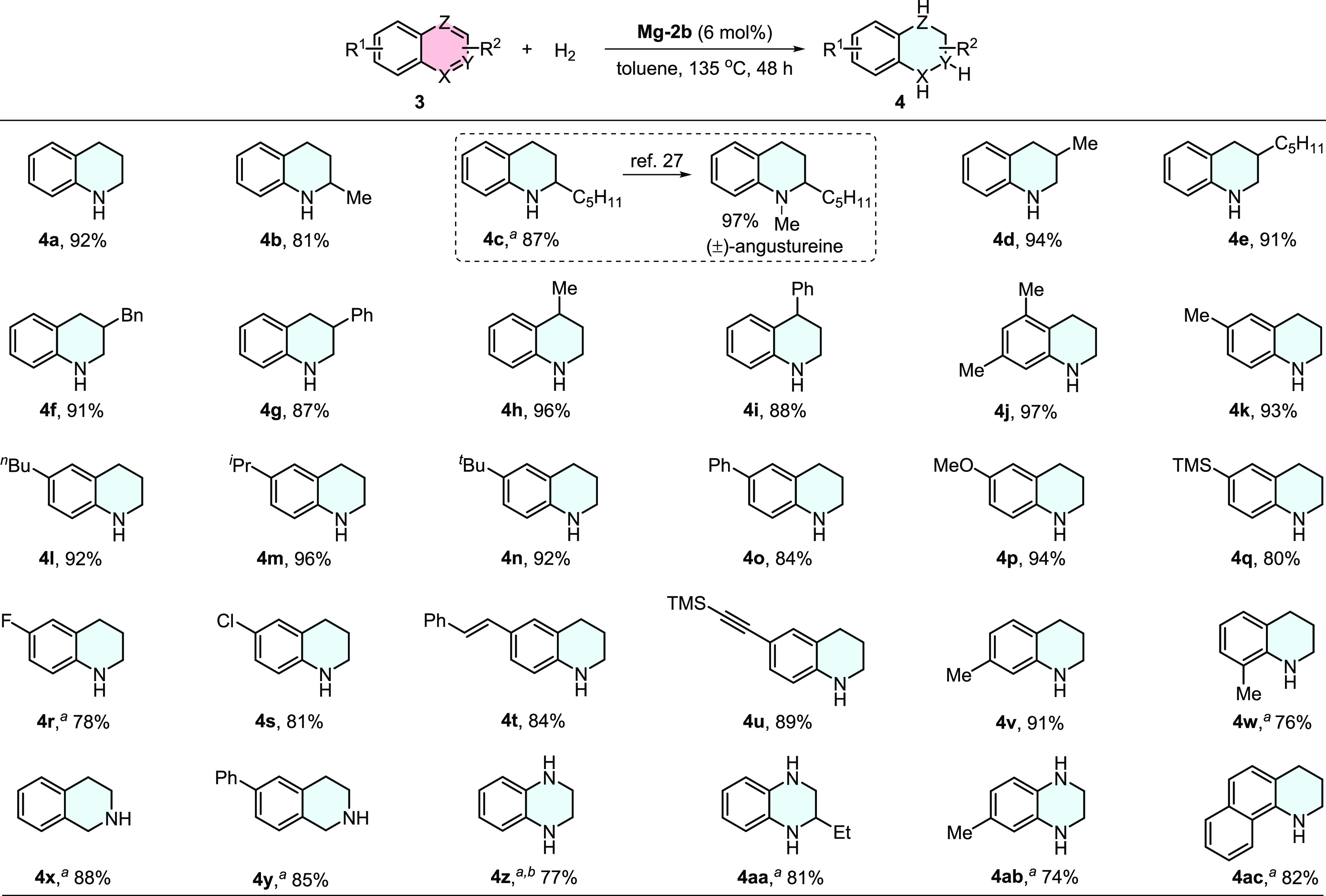
Catalytic hydrogenation of *N*-heteroarenes. Reaction conditions: **3** (0.3 mmol), **Mg-2b** (6 mol %), H_2_ (10 bar), toluene (1 mL), 135 °C, 48 h. The yields refer to isolated yields. Superscript a signifies that the reaction temperature was 150 °C. Superscript b signifies that 10 mol % of **Mg-2b** was used as the catalyst.

The successful hydrogenation of imines catalyzed by the de-aromatized magnesium complexes encouraged us to extend the developed catalysis to the hydrogenation of other compounds that contain C=N bond. Selective hydrogenation of quinolines and their closely related *N*-heteroarenes is an efficient way to access tetrahydroquinolines and related compounds, which are ubiquitous in natural products and drugs. Homogeneously catalyzed hydrogenation under mild conditions based on precious metals has been developed.^26^ In recent years, FLPs^5d^ and first-row transition metals^15^ have been reported to catalyze the hydrogenation of *N*-heteroarenes, avoiding the use of noble metals. However, to the best of our knowledge, the utilization of early main-group metal catalysts in this field has never been documented. The de-aromatized magnesium complexes may be capable of catalyzing the hydrogenation of quinolines and related *N*-heteroarenes. Surprisingly, under the same conditions as the imine hydrogenation, quinoline **3a** was smoothly converted into tetrahydroquinoline **4a** in 92% isolated yield catalyzed by **Mg-2b** (Figure 6). Substituted quinolines with a series of functional groups at different positions successfully afforded the desired cyclic amines (**4b**–**4w**). It is worth mentioning that the racemic noralkaloids **4c** was obtained in 87% yield, which can be converted to (±)-angustureine followed by methylation.^27^ Reducible functional groups, such as alkene and alkyne, were not affected in the current catalysis (**4t** and **4u**), showing great significance in organic synthesis. It is noted that 8-substituted quinoline (**3w**) generated relatively lower yields than others, probably because of its more steric hindrance. Other *N*-heteroarenes, including isoquinolines (**3x** and **3y**), quinoxalines (**3z**–**3ab**), and 7,8-benzoquinoline (**3ac**), were hydrogenated at 150 °C, generating corresponding products in high yields. These results indicated that the de-aromatized complexes are not only suitable for the hydrogenation of imines but also efficient in the selective hydrogenation of *N*-heteroarenes.

## Conclusions

In summary, we have developed a series of new de-aromatized magnesium pincer complexes by using the PNNH-type ligands. The complexes were well-characterized and are relatively stable compared to the reported Mg(PNP) complexes. Activation of H–H and N–H bonds by the de-aromatized complexes via an MLC process was demonstrated. The new magnesium pincer complexes were applied to the catalytic hydrogenation of various aldimines to generate secondary amines in excellent yields, providing a new strategy for the hydrogenation of imines using magnesium complexes. It is noted that, compared to previously reported hydrogenation of aldimines catalyzed by magnesium complexes, the reaction reported here represents a broader scope and higher yields. In addition, the developed complexes are efficient in the hydrogenation of ketimines, offering a successful example of homogeneous early main-group metal-catalyzed hydrogenation of ketimines. Mechanistic studies indicate that the aromatization/de-aromatization MLC process plays a significant role in achieving imine hydrogenation, as supported by control experiments and DFT studies. Furthermore, the developed catalysis is extended to the selective hydrogenation of *N*-heteroarenes, showing an unprecedented example of early main-group metal homogeneously catalyzed hydrogenation of *N*-heteroarenes. A variety of functional groups, including the fluoro, chloro, and C–C double and triple bonds, are well tolerated in the developed system. This study is a significant advancement in main-group metal catalysis and C=N bond hydrogenation. We believe that it will inspire the area of main-group metal chemistry and hydrogenation reactions.

## Supporting Information Available

Experimental procedures, NMR spectra, and computational details (PDF)

## References

[ref1] aJäkel C.; Paciello R. High-Throughput and Parallel Screening Methods in Asymmetric Hydrogenation. Chem. Rev. 2006, 106, 2912–2942. 10.1021/cr040675a. 16836304

[ref2] aXie J.-H.; Zhu S.-F.; Zhou Q.-L. Transition Metal-Catalyzed Enantioselective Hydrogenation of Enamines and Imines. Chem. Rev. 2011, 111, 1713–1760. 10.1021/cr100218m. 21166392

[ref3] aZell T.; Milstein D. Hydrogenation and Dehydrogenation Iron Pincer Catalysts Capable of Metal-Ligand Cooperation by Aromatization/Dearomatization. Acc. Chem. Res. 2015, 48, 1979–1994. 10.1021/acs.accounts.5b00027. 26079678

[ref4] aWerkmeister S.; Fleischer S.; Junge K.; Beller M. Towards a Zinc-Catalyzed Asymmetric Hydrogenation/Transfer Hydrogenation of Imines. Chem. – Asian J. 2012, 11, 2562–2568. 10.1002/asia.201200309. 22807402

[ref5] aStephan D. W. Frustrated Lewis Pairs: From Concept to Catalysis. Acc. Chem. Res. 2015, 48, 306–316. 10.1021/ar500375j. 25535796

[ref6] aHarder S. Early Main Group Metal Catalysis: Concepts and Reactions; Wiley-VCH Verlag, 2020; pp. 175–199.

[ref7] aWalling C.; Bollyky L. Base Catalyzed Homogeneous Hydrogenation. J. Am. Chem. Soc. 1961, 83, 2968–2969. 10.1021/ja01474a054.

[ref8] aSpielman J.; Buch F.; Harder S. Early Main-Group Metal Catalysts for the Hydrogenation of Alkenes with H_2_. Angew. Chem., Int. Ed. 2008, 47, 9434–9438. 10.1002/anie.200804657. 18979488

[ref9] aBauer H.; Alonso M.; Fischer C.; Rösch B.; Elsen H.; Harder S. Simple Alkaline-Earth Metal Catalysts for Effective Alkene Hydrogenation. Angew. Chem., Int. Ed. 2018, 57, 15177–15182. 10.1002/anie.201810026. 30230150

[ref10] Friedrich A.; Eyselein J.; Elsen H.; Langer J.; Pahl J.; Wiesinger M.; Harder S. Cationic Aluminium Complexes as Catalysts for Imine Hydrogenation. Chem. – Eur. J. 2021, 27, 7756–7763. 10.1002/chem.202100641. 33780071 PMC8252007

[ref11] aElsen H.; Färber C.; Ballmann G.; Harder S. LiAlH_4_: From Stoichiometric Reduction to Imine Hydrogenation Catalysis. Angew. Chem., Int. Ed. 2018, 57, 7156–7160. 10.1002/anie.201803804. 29683545

[ref12] Liang Y.; Das U. K.; Luo J.; Posner-Diskin Y.; Avram L.; Milstein D. Magnesium Pincer Complexes and Their Applications in Catalytic Semihydrogenation of Alkynes and Hydrogenation of Alkenes: Evidence for Metal–Ligand Cooperation. J. Am. Chem. Soc. 2022, 144, 19115–19126. 10.1021/jacs.2c08491. 36194894 PMC9585592

[ref13] aWang C.; Villa-Marcos B.; Xiao J. Hydrogenation of Imino Bonds with Half-Sandwich Metal Catalysts. Chem. Commun. 2011, 47, 9773–9785. 10.1039/c1cc12326b. 21625723

[ref14] aZhang G.; Scott B. L.; Hanson S. K. Mild and Homogeneous Cobalt-Catalyzed Hydrogenation of C=C, C=O, and C=N Bonds. Angew. Chem., Int. Ed. 2012, 51, 12102–12106. 10.1002/anie.201206051. 23042754

[ref15] aChakraborty S.; Brennessel W. W.; Jones W. D. A Molecular Iron Catalyst for the Acceptorless Dehydrogenation and Hydrogenation of *N*-Heterocycles. J. Am. Chem. Soc. 2014, 136, 8564–8567. 10.1021/ja504523b. 24877556

[ref16] aMagre M.; Szewczyk M.; Rueping M. s-Block Metal Catalysts for the Hydroboration of Unsaturated Bonds. Chem. Rev. 2022, 122, 8261–8312. 10.1021/acs.chemrev.1c00641. 35254061 PMC9100617

[ref17] aCrimmin M. R.; Arrowsmith M.; Barrett A. G. M.; Casely I. J.; Hill M. S.; Procopiou P. A. Intramolecular Hydroamination of Aminoalkenes by Calcium and Magnesium Complexes: A Synthetic and Mechanistic Study. J. Am. Chem. Soc. 2009, 131, 9670–9685. 10.1021/ja9003377. 19552442

[ref18] aLampland N. L.; Pindwal A.; Neal S. R.; Schlauderaff S.; Ellern A.; Sadow A. D. Magnesium-Catalyzed Hydrosilylation of α,β-Unsaturated Esters. Chem. Sci. 2015, 6, 6901–6907. 10.1039/C5SC02435H. 29861929 PMC5947512

[ref19] aArrowsmith M.; Hill M. S.; Hadlington T.; Kociok-köhn G.; Weetman C. Magnesium-Catalyzed Hydroboration of Pyridines. Organometallics 2011, 30, 5556–5559. 10.1021/om2008138.

[ref20] aSpielmann J.; Bolte M.; Harder S. Synthesis and Structure of A Magnesium–Amidoborane Complex and Its Role in Catalytic Formation of A New Bis-Aminoborane Ligand. Chem. Commun. 2009, 6934–6936. 10.1039/b914979a. 19904352

[ref21] aGunanathan C.; Milstein D. Metal–Ligand Cooperation by Aromatization-Dearomatization: A New Paradigm in Bond Activation and “Green” Catalysis. Acc. Chem. Res. 2011, 44, 588–602. 10.1021/ar2000265. 21739968

[ref22] In previously reported magnesium complexes, the ^31^P{^1^H} NMR chemical shifts of the deprotonated phosphine side arm (close to 0 ppm) appear at higher fields than the protonated side arm (close to 20 ppm).

[ref23] aGreen S. P.; Jones C.; Stasch A. Stable Adducts of a Dimeric Magnesium(I) Compound. Angew. Chem., Int. Ed. 2008, 47, 9079–9083. 10.1002/anie.200803960. 18925599

[ref24] aFogle E.; Garg J. A.; Hu P.; Leitus G.; Shimon L. J. W.; Milstein D. System with Potential Dual Modes of Metal–Ligand Cooperation: Highly Catalytically Active Pyridine-Based PNNH–Ru Pincer Complexes. Chem. – Eur. J. 2014, 20, 15727–15731. 10.1002/chem.201405295. 25331061

[ref25] aDankert F.; Siewert J.-E.; Cupta P.; Weigend F.; Junghans-Hering C. Metal-Free N–H Bond Activation by Phospha-Wittig Reagents. Angew. Chem., Int. Ed. 2022, 61, e202207064. 10.1002/anie.202207064 PMC9400956 35594171

[ref26] aWang W.-B.; Lu S.-M.; Yang P.-Y.; Han X.-W.; Zhou Y.-G. Highly Enantioselective Iridium-Catalyzed Hydrogenation of Heteroaromatic Compounds, Quinolines. J. Am. Chem. Soc. 2003, 125, 10536–10537. 10.1021/ja0353762. 12940733

[ref27] Shahane S.; Louafi F.; Moreau J.; Hurvois J.-P.; Renaud J.-L.; van de Weghe P.; Roisnel T. Synthesis of Alkaloids of Galipea *officinalis* by Alkylation of an *α*-Amino Nitrile. Eur. J. Org. Chem. 2008, 4622–4631. 10.1002/ejoc.200800512.

