# Supplementary material for: Designing New Magnesium Pincer Complexes for Catalytic Hydrogenation of Imines and N-Heteroarenes: H2 and N–H Activation by Metal–Ligand Cooperation as Key Steps
Source: J Am Chem Soc. 2023 Apr 17;145(16):9164–75. doi: 10.1021/jacs.3c01091 (PMC10141328; doi:10.1021/jacs.3c01091)
Supplement: Supplementary file 1 — ja3c01091_si_001.pdf [file ja3c01091_si_001.pdf]

Supporting Information

Designing New Magnesium Pincer Complexes for Catalytic Hydrogenation of Imines and *N*-Heteroarenes: H_2 and N–H Activation by Metal-Ligand Cooperation as Key Steps

Yaoyu Liang,[†] Jie Luo,[†] Yael Diskin-Posner,[‡] David Milstein[†]*

[†]*Department of Molecular Chemistry and Materials Science, The Weizmann Institute of Science, Rehovot 7610001, Israel;*
[‡]*Department of Chemical Research Support, The Weizmann Institute of Science, Rehovot 7610001, Israel;*
**e-mail: david.milstein@weizmann.ac.il.*

Table of Contents

1 General considerations 2
2 Synthesis and characterization of magnesium complexes 3
3 Bond activation by magnesium pincer complexes 32
4 Catalytic hydrogenation of imines and *N*-heteroarenes 57
5 Mechanistic studies 80
6 Computational details 84
7 NMR spectra 116
8 References 178

1 General considerations

All the experiments were carried out under an atmosphere of purified nitrogen in a Vacuum Atmosphere glovebox equipped with a MO 40-2 inert gas purifier or using standard Schlenk techniques. All commercial reagents were purchased from Strem, Sigma Aldrich, Acros, or Alfa Aesar, and used as received without further purification. Toluene, benzene, pentane, Et_2O, THF, and dioxane were refluxed over sodium/benzophenone, distilled under argon or nitrogen atmosphere, and stored over activated 4Å molecular sieves (MS). Other solvents were used after degassing with nitrogen. Deuterated benzene, toluene, and THF were degassed with nitrogen and stored in the glovebox over 4Å MS. NMR spectra were recorded on Bruker AVANCE III (300 MHz or 400 MHz) or AVANCE III HD (500 MHz) spectrometers and are reported in ppm (δ). ^{1}H NMR spectra are referred to the residual solvent peaks (THF-d_8: 1.72 ppm, benzene-d_6: 7.15 ppm, and $CHCl_3$: 7.26 ppm), and ^{13}C NMR spectra are referred to the residual solvent peaks (THF-d_8: 67.21 ppm, benzene-d_6: 128.06 ppm, and $CHCl_3$: 77.16 ppm). ^{31}P NMR chemical shifts are referenced with respect to an external solution of 85% phosphoric acid in D_2O. NMR spectroscopy abbreviations: s, singlet; d, doublet; t, triplet; q, quartet; m, multiplet; br, broad. GC-MS analyses were carried out on HP 6890/5973 (MS detector) instruments equipped with a 30 m column (Restek 5MS, 0.32 mm internal diameter) with a 5% phenylmethylsilicone coating (0.25 mm) with He as carrier gas.

2 Synthesis and characterization of magnesium complexes

2.1 Synthesis and characterization of Mg-1a

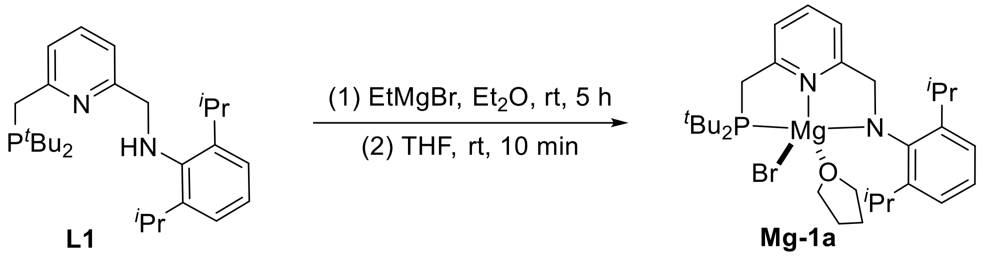

In a N_2 glovebox, the PNNH ligand **L1** (426.3 mg, 1 mmol) was dissolved in a 20 mL vial by Et_2O (3 mL). EtMgBr (3.0 M in Et_2O, 333 μL, 1 mmol) was slowly dropped in the solution at room temperature. The resulting solution was stirred at room temperature for 5 h, affording a yellowish precipitate. The solid was isolated by filtration and was washed with Et_2O three times. THF (2 mL) was added to dissolve the solid, and the solution was stirred at room temperature for about 10 min. Removing the solvent provides **Mg-1a** as a yellowish solid. To further purify the complex, 1 mL of THF was added to dissolve the solid, and pentane (about 1 mL) was slowly added. The resulting solution was kept at room temperature until yellowish crystals were formed. The crystals were suitable for X-ray diffraction. Pentane was decanted, and the solid was dried under vacuum to give **Mg-1a** (474.2 mg, 79% yield). The PNNH ligand **L1** was prepared according to the literature procedure.[1]

^{1}H NMR (400 MHz, THF-*d*$_8$) δ 7.71 (t, *J* = 7.7 Hz, 1H, Py***H***), 7.63 (d, *J* = 7.6 Hz, 1H, Py***H***), 7.13 (d, *J* = 7.7 Hz, 1H, Py***H***), 6.99 (d, *J* = 7.5 Hz, 2H, Ar***H***), 6.85 (t, *J* = 7.5 Hz, 1H, Ar***H***), 4.63 (s, 2H, PyC***H***$_2$N), 3.90 (dt, *J* = 13.7, 6.8 Hz, 2H, C***H***(CH$_3$)$_2$), 3.70 – 3.62 (m, 6H, OC***H***$_2$ and PyC***H***$_2$P), 1.87 – 1.81 (m, 4H, OCH$_2$C***H***$_2$), 1.28 (d, *J* = 11.3 Hz, 18H, PC(C***H***$_3$)$_3$), 1.19 (d, *J* = 6.9 Hz, 12H, CH(C***H***$_3$)$_2$).

^{13}C NMR (101 MHz, THF-*d*$_8$) δ 165.89 (s, Py***C***), 158.87 (d, *J* = 9.3 Hz, Py***C***), 156.49 (s, Ar***C***), 147.88 (s, Ar***C***), 137.77 (s, Py***C***), 123.17 (s, Ar***C***), 122.43 (d, *J* = 13.3 Hz, Py***C***), 121.24 (s, Ar***C***), 120.21 (s, Py***C***), 68.03 (s, O***C***H$_2$), 62.20 (s, Py***C***H$_2$N), 32.45 (d, *J* = 13.7 Hz, P***C***(CH$_3$)$_3$), 30.31 (d, *J* = 10.8 Hz, PC(***C***H$_3$)$_3$), 29.57 (d, *J* = 11.1 Hz, Py***C***H$_2$P), 28.21 (s, ***C***H(CH$_3$)$_2$), 27.71 (s, CH(***C***H$_3$)$_2$), 26.18 (s, OCH$_2$***C***H$_2$).

^{31}P NMR (162 MHz, THF-*d*$_8$) δ 22.89 (s).

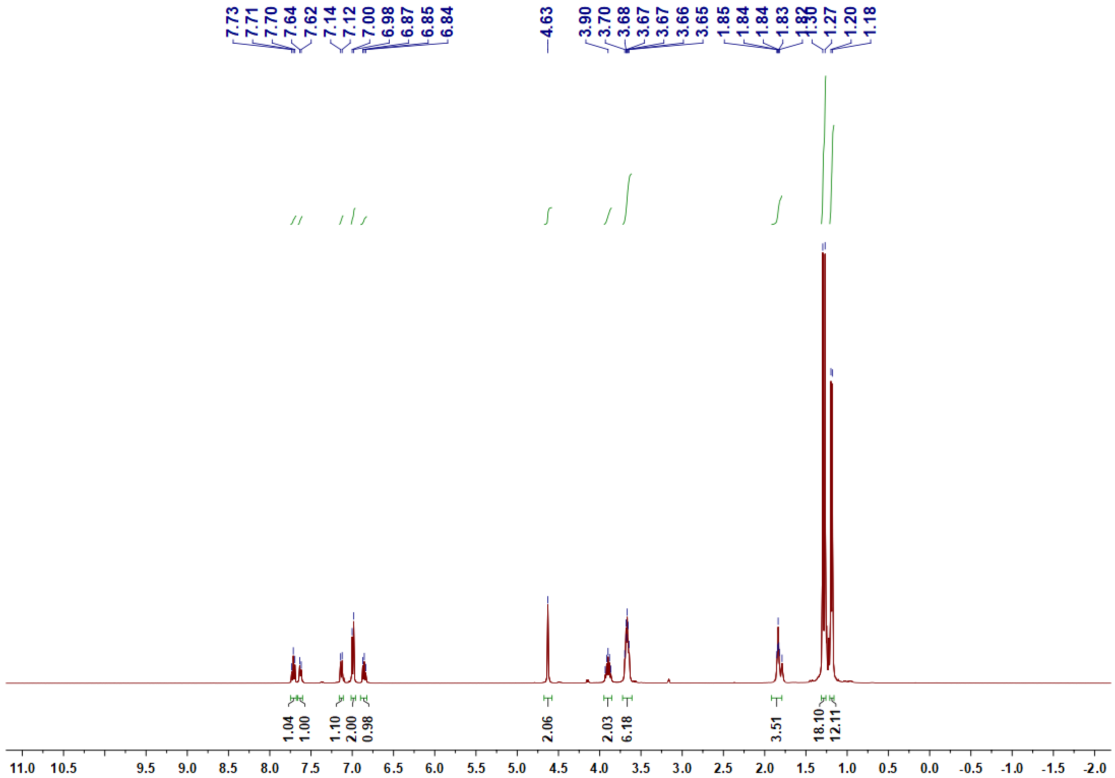

Figure S1. ^{1}H NMR (400 MHz, THF-d_8) spectrum of **Mg-1a**

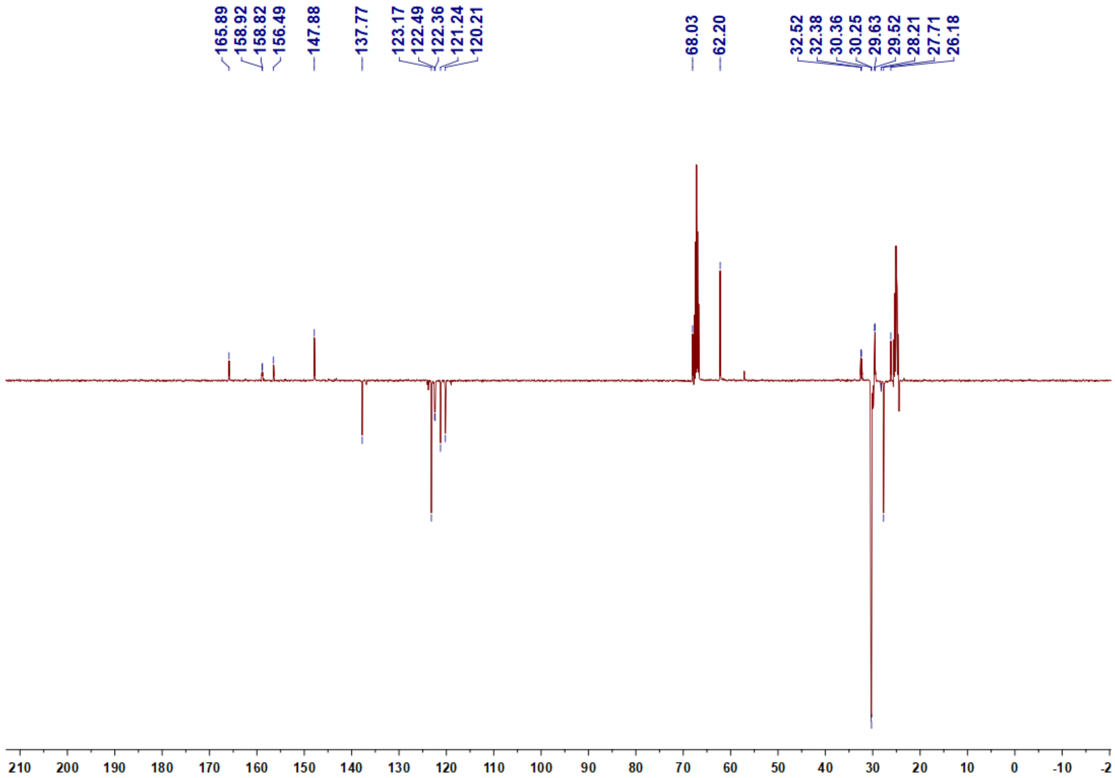

Figure S2. ^{13}C-DEPTQ NMR (101 MHz, THF-d_8) spectrum of **Mg-1a**

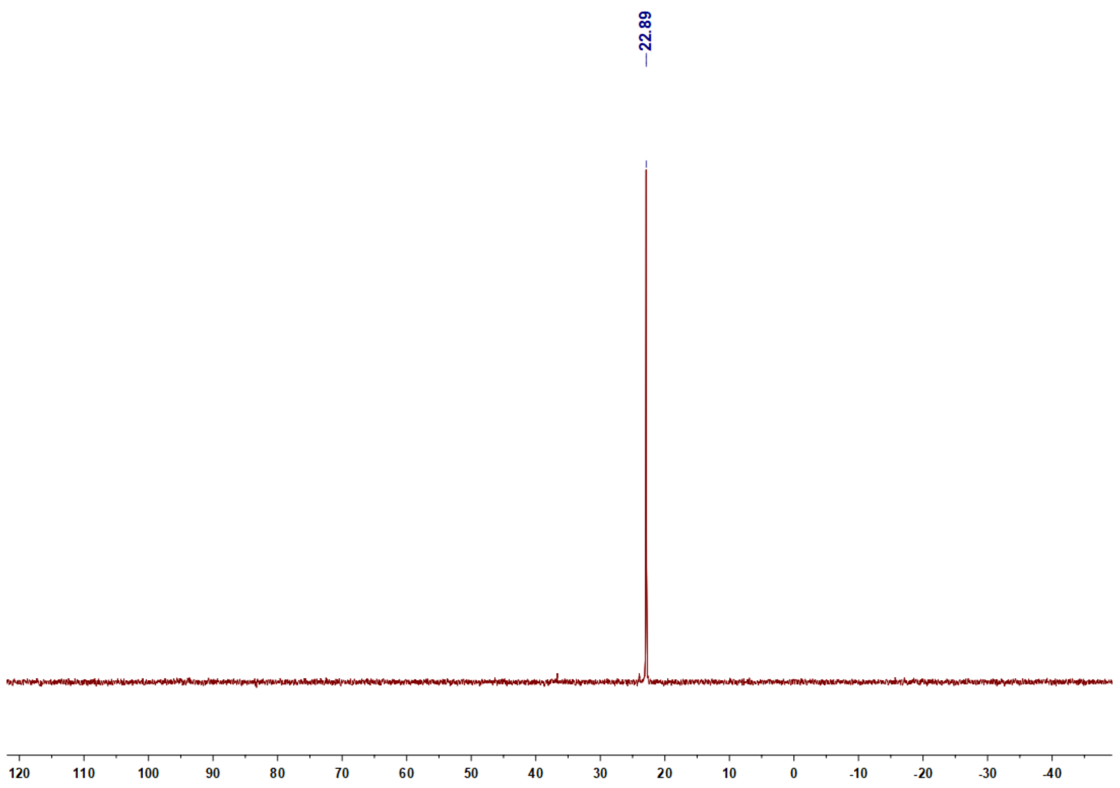

Figure S3. ^{31}P NMR (162 MHz, THF-d_8) spectrum of **Mg-1a**

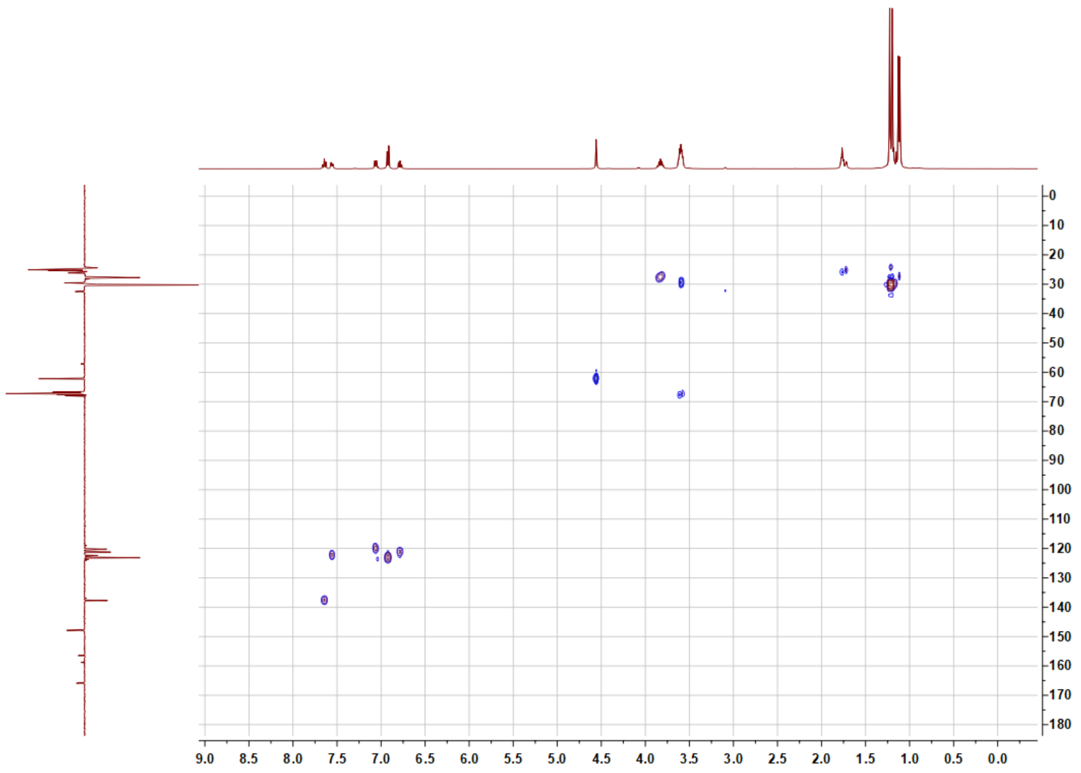

Figure S4. HSQC spectrum of **Mg-1a** in THF-d_8

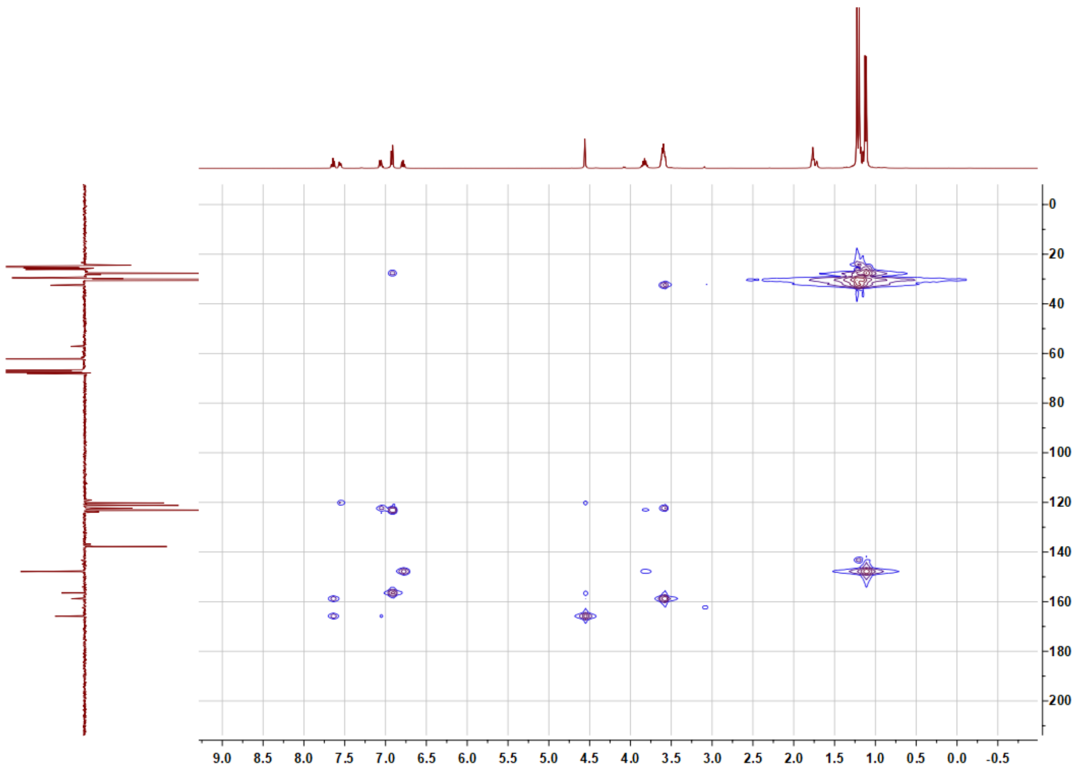

Figure S5. HMBC spectrum of **Mg-1a** in THF-d_8

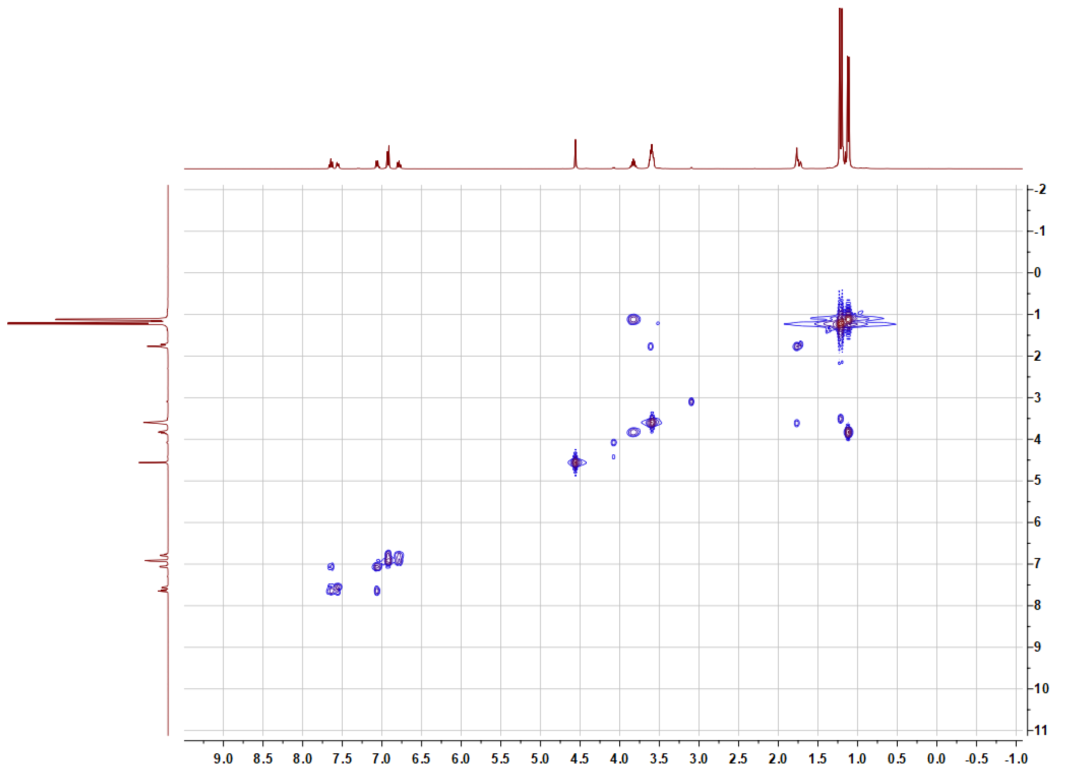

Figure S6. H-H COSY spectrum of **Mg-1a** in THF-d_8

The diffraction data from single crystals of **Mg-1a** were collected on Rigaku Synergy-R diffractometer dual source equipped with a HyPix ARC 150º detector, with CuKa (λ = 1.54184 Å), at 250 K Degrees. The data were processed with CrysAlis^{PRO}[2]. The structures were solved with SHELXT[3]. Full matrix least-squares and refined based on F^2 with SHELXL[4]. All structure solution and refinement programs are implemented in Olex-2 GUI[5]. All non-hydrogen atoms were refined with anisotropic displacement coefficients. Hydrogens were placed in calculated positions and refined in riding mode. Supplementary crystallographic data have been deposited at the Cambridge Crystallographic Data Center (CCDC 2237494).

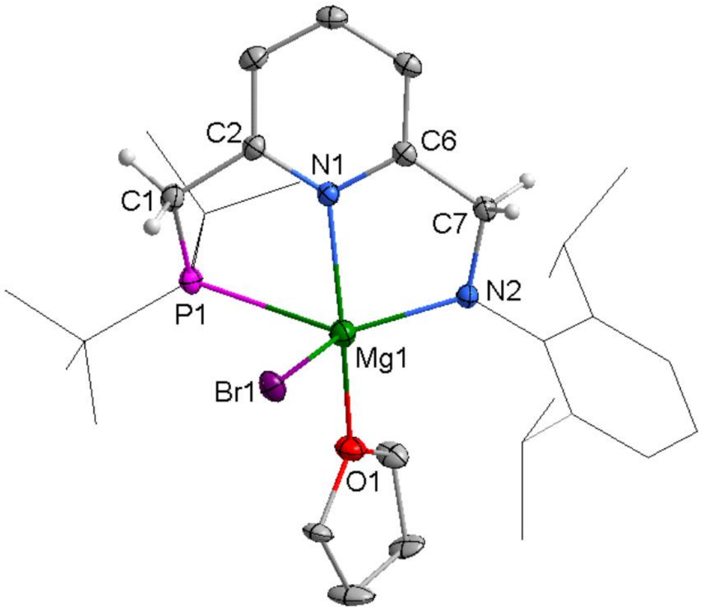

Figure S7. X-ray crystal structure of **Mg-1a**

Table S1. Crystal data and structure refinement for Mg-1a

Empirical formula	$C_{31}H_{50}BrMgN_2OP$
Formula weight	601.92
Temperature/K	250.00(10)
Crystal system	triclinic
Space group	*P-1*
a/Å	10.5549(2)
b/Å	11.2639(2)
c/Å	14.4184(3)
α/°	89.0590(10)

β/°	87.3840(10)
γ/°	75.7970(10)
Volume/Å^3	1660.04(6)
Z	2
ρ_{calc}g/cm^3	1.204
μ/mm^{-1}	2.501
F(000)	640.0
Crystal size/mm^3	0.777 × 0.466 × 0.269
Radiation	Cu Kα (λ = 1.54184)
2Θ range for data collection/°	10.14 to 149.004
Index ranges	-13 ≤ h ≤ 12, -14 ≤ k ≤ 13, -16 ≤ l ≤ 18
Reflections collected	30155
Independent reflections	6661 [R_{int} = 0.0161, R_{sigma} = 0.0109]
Data/restraints/parameters	6661/0/354
Goodness-of-fit on F^2	1.046
Final R indexes [I>=2σ (I)]	R_1 = 0.0283, wR_2 = 0.0767
Final R indexes [all data]	R_1 = 0.0291, wR_2 = 0.0772
Largest diff. peak/hole / e Å^{-3}	0.42/-0.45

Table S2. Bond lengths for Mg-1a

Atom	Atom	Length/Å	Atom	Atom	Length/Å
Br1	Mg1	2.5120(5)	C6	C7	1.499(2)
P1	Mg1	2.8367(6)	C6	C5	1.392(2)
P1	C8	1.8853(18)	C24	C22	1.523(3)
P1	C1	1.8500(17)	C22	C21	1.517(3)
P1	C12	1.8862(18)	C22	C23	1.505(3)
Mg1	O1	2.0554(12)	C2	C1	1.503(2)
Mg1	N1	2.1594(12)	C2	C3	1.381(2)
Mg1	N2	1.9939(13)	C19	C18	1.374(3)
O1	C28	1.437(2)	C25	C26	1.535(3)
O1	C31	1.432(3)	C25	C27	1.533(3)
N1	C6	1.3399(18)	C25	C17	1.514(3)
N1	C2	1.3488(18)	C16	C17	1.419(2)
N2	C7	1.4470(19)	C16	C21	1.414(2)

N2	C16	1.4102(18)	C12	C14	1.543(3)
C9	C8	1.526(3)	C12	C13	1.518(3)
C20	C19	1.372(3)	C5	C4	1.374(2)
C20	C21	1.395(2)	C3	C4	1.385(2)
C8	C11	1.522(3)	C31	C30	1.446(5)
C8	C10	1.535(3)	C31	C30A	1.403(17)
C15	C12	1.530(3)	C17	C18	1.396(2)
C28	C29	1.457(5)	C29	C30	1.450(7)
C28	C29A	1.60(2)	C29A	C30A	1.13(3)

Table S3. Bond angles for Mg-1a

Atom	Atom	Atom	Angle/°	Atom	Atom	Atom	Angle/°
C8	P1	Mg1	124.82(6)	N2	C7	C6	112.38(12)
C8	P1	C12	111.46(9)	C21	C22	C24	113.67(18)
C1	P1	Mg1	88.27(5)	C23	C22	C24	109.51(19)
C1	P1	C8	102.18(8)	C23	C22	C21	111.41(16)
C1	P1	C12	103.80(9)	N1	C2	C1	115.61(13)
C12	P1	Mg1	118.25(6)	N1	C2	C3	121.53(14)
Br1	Mg1	P1	99.047(17)	C3	C2	C1	122.86(14)
O1	Mg1	Br1	99.83(4)	C20	C19	C18	119.11(17)
O1	Mg1	P1	92.54(4)	C2	C1	P1	111.72(10)
O1	Mg1	N1	156.09(5)	C27	C25	C26	109.71(18)
N1	Mg1	Br1	100.46(4)	C17	C25	C26	112.25(16)
N1	Mg1	P1	71.90(3)	C17	C25	C27	112.06(18)
N2	Mg1	Br1	122.21(4)	N2	C16	C17	120.96(14)
N2	Mg1	P1	133.44(4)	N2	C16	C21	120.48(14)
N2	Mg1	O1	99.93(5)	C21	C16	C17	118.55(14)
N2	Mg1	N1	79.64(5)	C15	C12	P1	106.89(12)
C28	O1	Mg1	128.24(13)	C15	C12	C14	106.8(2)
C31	O1	Mg1	122.32(12)	C14	C12	P1	114.37(16)
C31	O1	C28	109.44(16)	C13	C12	P1	109.77(16)
C6	N1	Mg1	112.69(9)	C13	C12	C15	108.1(2)
C6	N1	C2	119.56(12)	C13	C12	C14	110.6(2)

C2	N1	Mg1	127.72(10)	C4	C5	C6	119.12(14)
C7	N2	Mg1	115.74(9)	C2	C3	C4	118.96(14)
C16	N2	Mg1	131.81(10)	C5	C4	C3	119.45(14)
C16	N2	C7	112.34(11)	O1	C31	C30	106.0(3)
C19	C20	C21	121.91(19)	C30A	C31	O1	103.2(8)
C9	C8	P1	107.10(12)	C16	C17	C25	121.98(14)
C9	C8	C10	108.3(2)	C18	C17	C25	118.74(16)
C11	C8	P1	115.66(14)	C18	C17	C16	119.24(17)
C11	C8	C9	107.7(2)	C20	C21	C22	120.05(17)
C11	C8	C10	109.61(19)	C20	C21	C16	119.38(17)
C10	C8	P1	108.21(16)	C16	C21	C22	120.54(14)
O1	C28	C29	106.7(3)	C19	C18	C17	121.75(18)
O1	C28	C29A	94.8(11)	C30	C29	C28	107.1(3)
N1	C6	C7	116.85(12)	C31	C30	C29	107.8(3)
N1	C6	C5	121.29(13)	C30A	C29A	C28	111.4(19)
C5	C6	C7	121.85(13)	C29A	C30A	C31	115.0(18)

2.2 Synthesis and characterization of Mg-2a

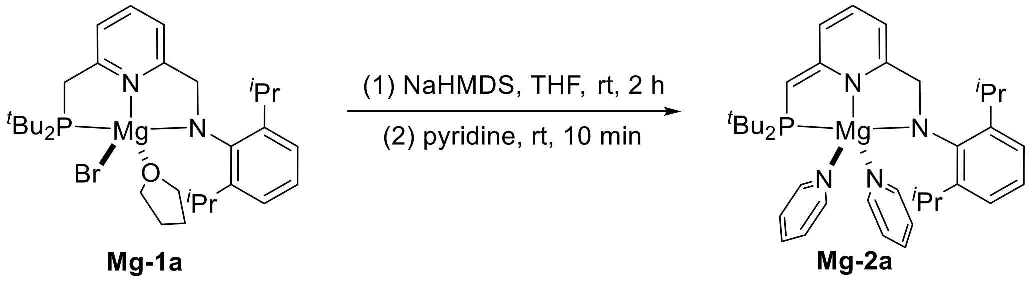

In a N_2 glovebox, **Mg-1a** (180.4 mg, 0.3 mmol) was dissolved by THF in a 20 mL vial. NaHMDS (55.0 mg, 0.3 mmol) was added, and the resulting solution was stirred at room temperature for 2 h, then filtered through a small piece of cotton to remove the solid. The orange solution was concentrated under vacuum to afford a thick oil. The thick oil was dissolved by pyridine and then kept at room temperature for 10 min. The pyridine was removed to give an orange solid. To further purify the product, benzene (about 0.5 mL) was added to dissolve the solid, and pentane (about 1 mL) was slowly added. The resulting solution was kept at -30 ºC until orange crystals formed. The crystals were suitable for X-ray diffraction. The solvent was decanted, and the solid

was washed with pentane and then dried under vacuum to give the pure **Mg-2a** (120.1 mg, 66% yield).

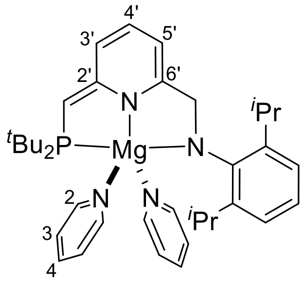

^{1}H NMR (400 MHz, C_6D_6) δ 8.45 (d, *J* = 4.7 Hz, 4H, Py***H***^{2}), 7.25 – 7.16 (m, 3H, NAr***H***), 6.94 – 6.87 (m, 1H, Py***H***$^{4'}$), 6.75 (t, *J* = 7.7 Hz, 2H, Py***H***^{4}), 6.51 (d, *J* = 8.6 Hz, 1H, Py***H***$^{3'}$), 6.45 (dd, *J* = 7.4, 6.2 Hz, 4H, Py***H***^{3}), 5.74 (d, *J* = 6.6 Hz, 1H, Py***H***$^{5'}$), 4.57 (s, 2H, ArNC***H***$_2$), 3.98 – 3.84 (m, 2H, C***H***(CH_3)$_2$), 3.68 (d, *J* = 5.1 Hz, 1H, PC***H***), 1.29 (br, 6H, CH(C***H***$_3$)$_2$), 1.18 (d, *J* = 11.7 Hz, 18H, PC(C***H***$_3$)$_3$), 0.89 (br, 6H, CH(C***H***$_3$)$_2$).

^{13}C NMR (101 MHz, C_6D_6) δ 168.10 (d, *J* = 16.0 Hz, Py***C***$^{2'}$), 161.74 (d, *J* = 2.8 Hz, Py***C***$^{6'}$), 158.87 (s, Ar***C***), 149.97 (s, Py***C***^{2}), 147.85 (s, Ar***C***), 138.48 (s, Py***C***^{4}), 134.86 (d, *J* = 2.1 Hz, Py***C***$^{4'}$), 124.40 (s, Py***C***^{3}), 123.57 (s, Ar***C***), 120.98 (s, Ar***C***), 113.76 (d, *J* = 8.4 Hz, Py***C***$^{3'}$), 97.70 (s, Py***C***$^{5'}$), 62.50 (s, N***C***H_2), 55.68 (d, *J* = 33.2 Hz, P***C***H), 33.91 (s, P***C***(CH_3)$_3$), 30.22 (d, *J* = 8.4 Hz, PC(***C***H_3)$_3$), 27.30 (s, ***C***H(CH_3)$_2$), 26.26 (s, CH(***C***H_3)$_2$), 24.57 (s, CH(***C***H_3)$_2$).

^{31}P NMR (162 MHz, C_6D_6) δ 2.66 (s).

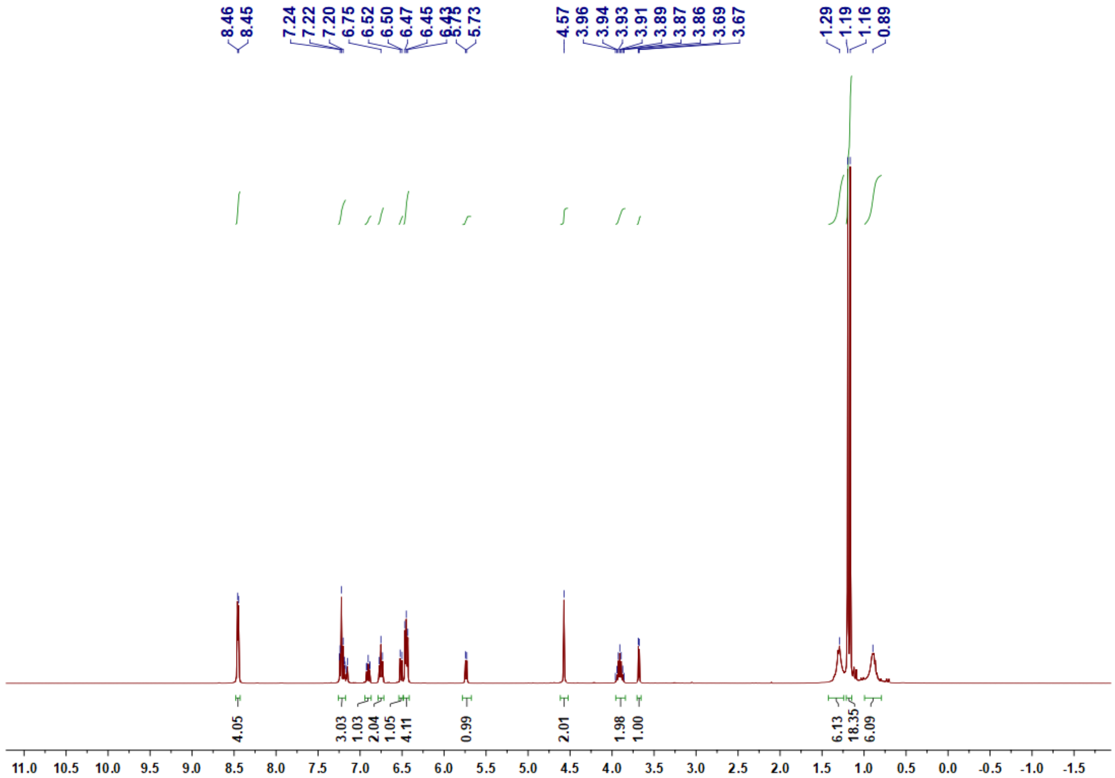

Figure S8. ^{1}H NMR (400 MHz, C_6D_6) spectrum of **Mg-2a**

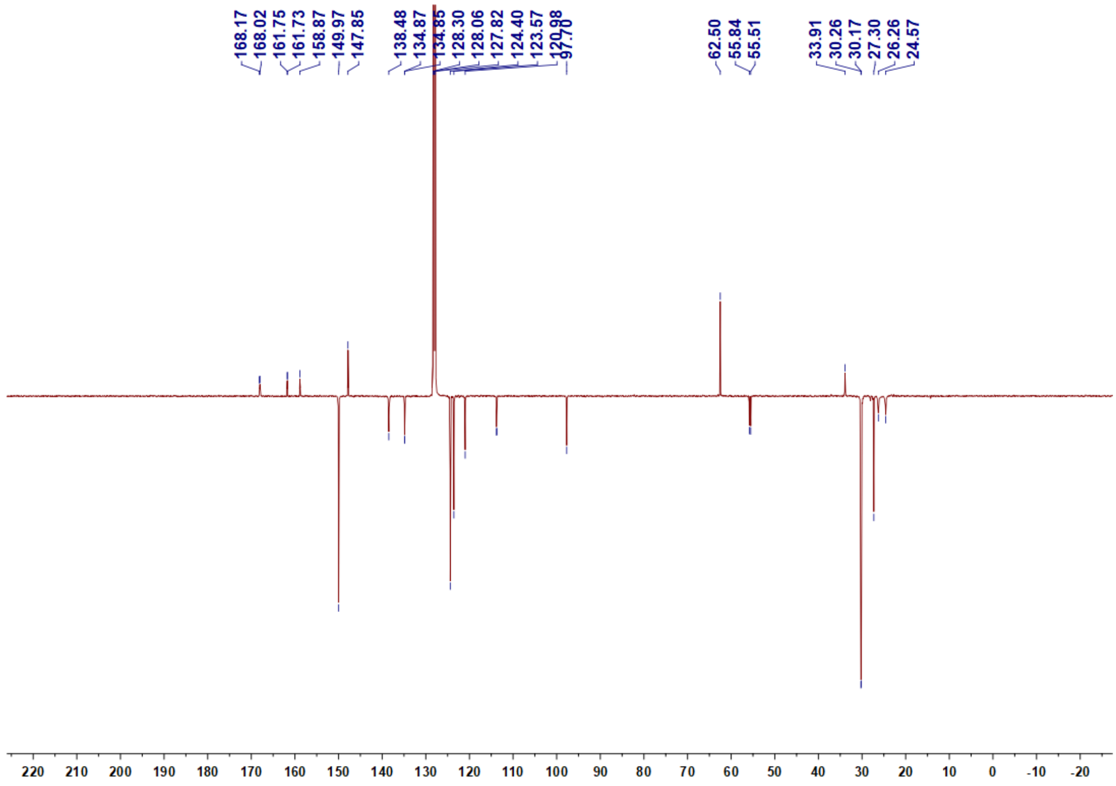

Figure S9. ^{13}C-DEPTQ NMR (101 MHz, C_6D_6) spectrum of **Mg-2a**

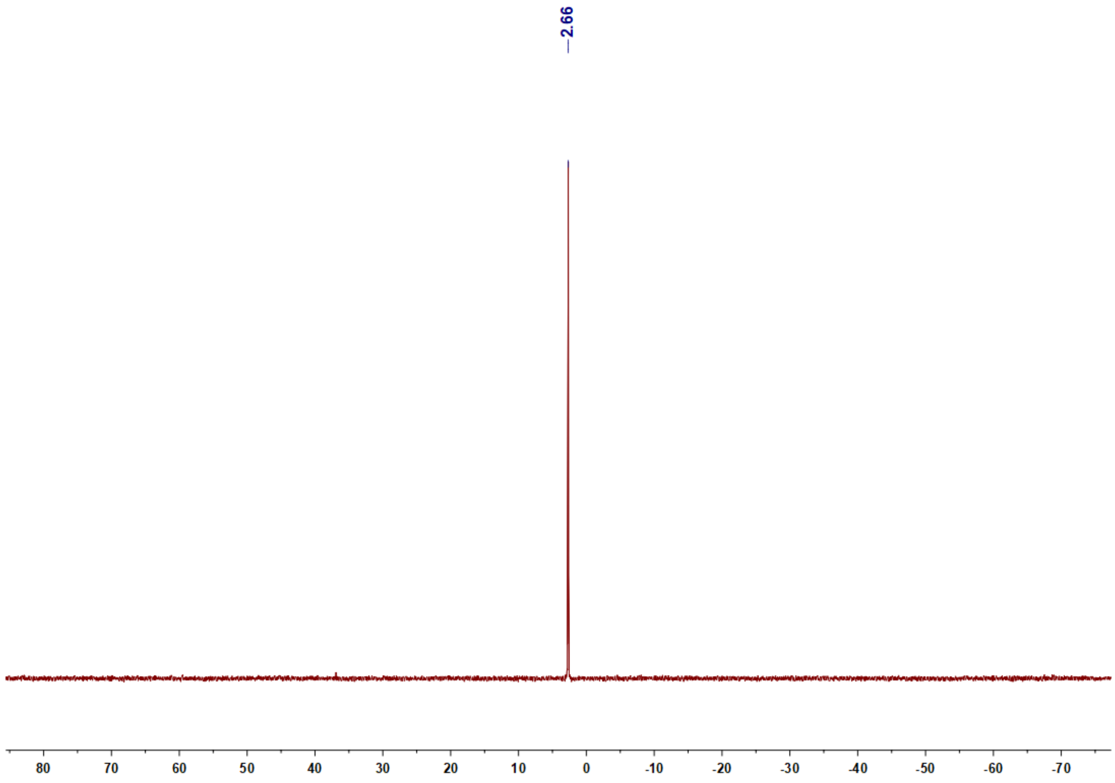

Figure S10. ^{31}P NMR (162 MHz, C_6D_6) spectrum of **Mg-2a**

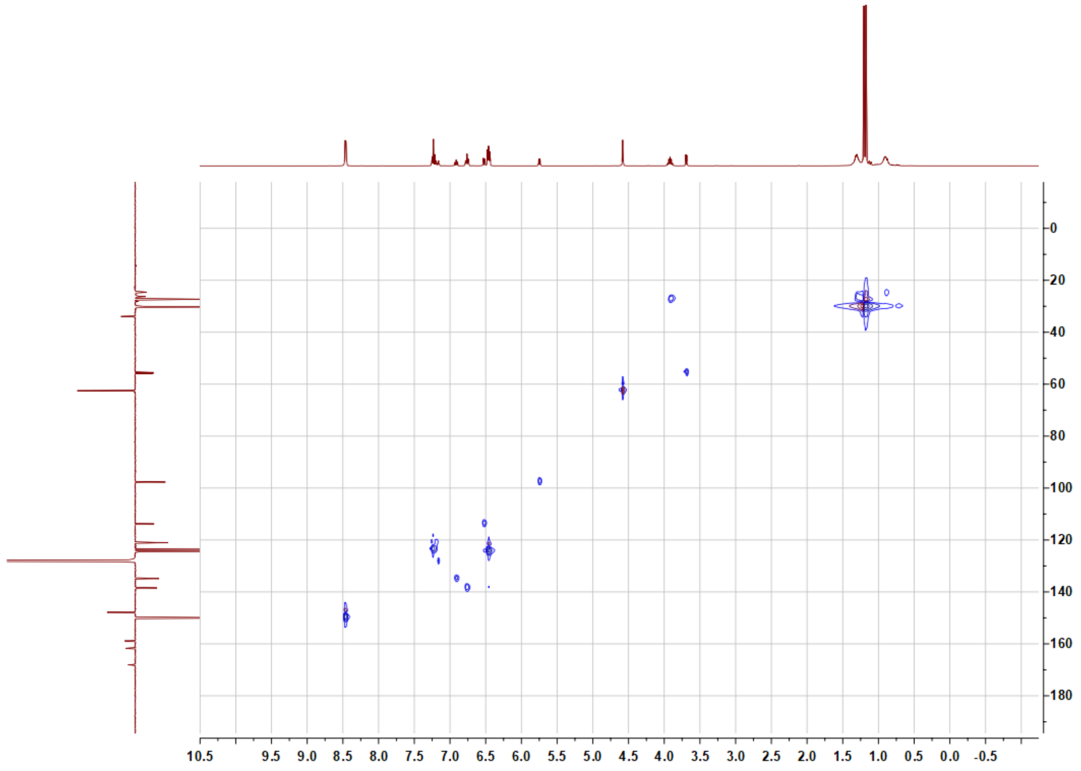

Figure S11. HSQC spectrum of **Mg-2a** in C_6D_6

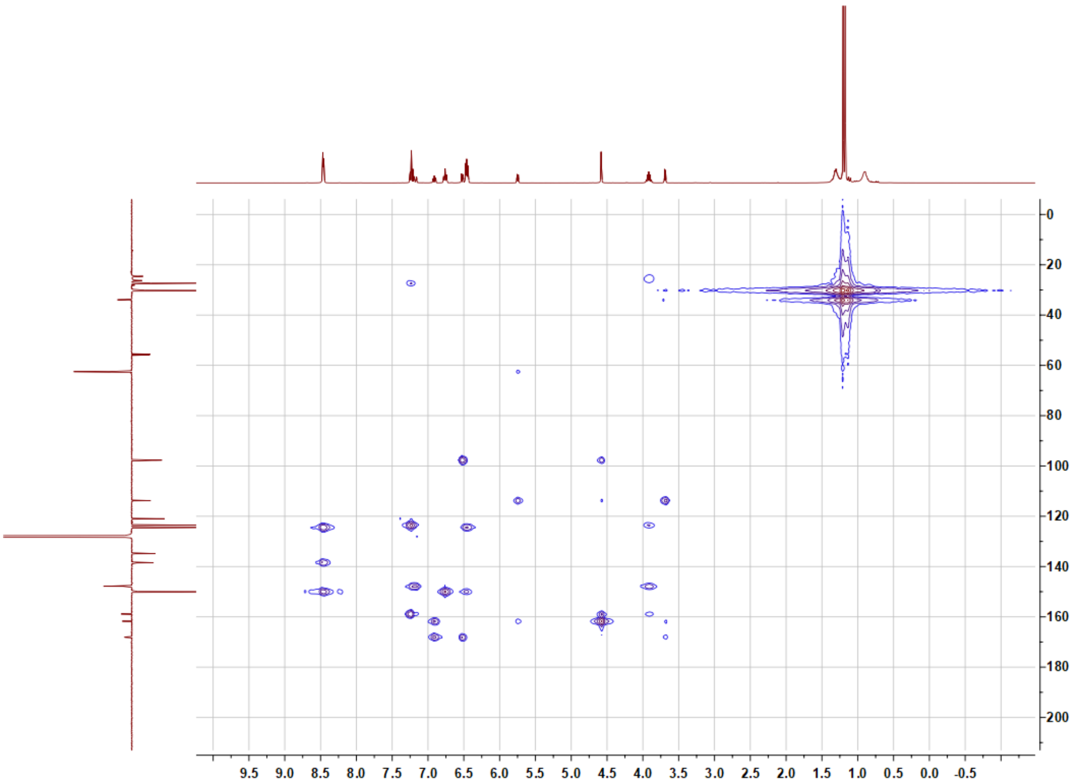

Figure S12. HMBC spectrum of **Mg-2a** in C_6D_6

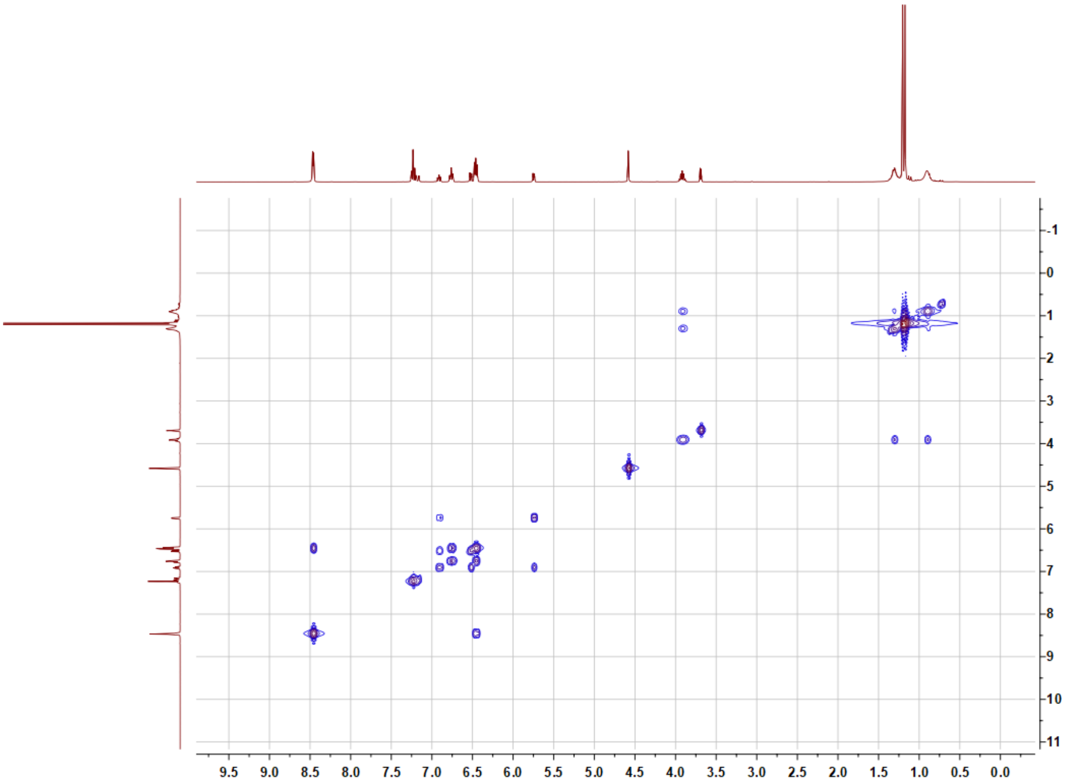

Figure S13. H-H COSY spectrum of **Mg-2a** in C_6D_6

The diffraction data from single crystals of **Mg-2a** were collected on Rigaku Synergy-S diffractometer dual source equipped with Dectris Pilatus3 R CdTe 300 K detector and microfocus, with MoKα (λ=0.71073Å). The data were processed with CrysAlis^{PRO}[2]. The structures were solved with SHELXT[3]. Full matrix least-squares and refined based on F^2 with SHELXL[4]. All structure solution and refinement programs are implemented in Olex-2 GUI[5]. All non-hydrogen atoms were refined with anisotropic displacement coefficients. Hydrogens were placed in calculated positions and refined in riding mode. Supplementary crystallographic data have been deposited at the Cambridge Crystallographic Data Center (CCDC 2237495).

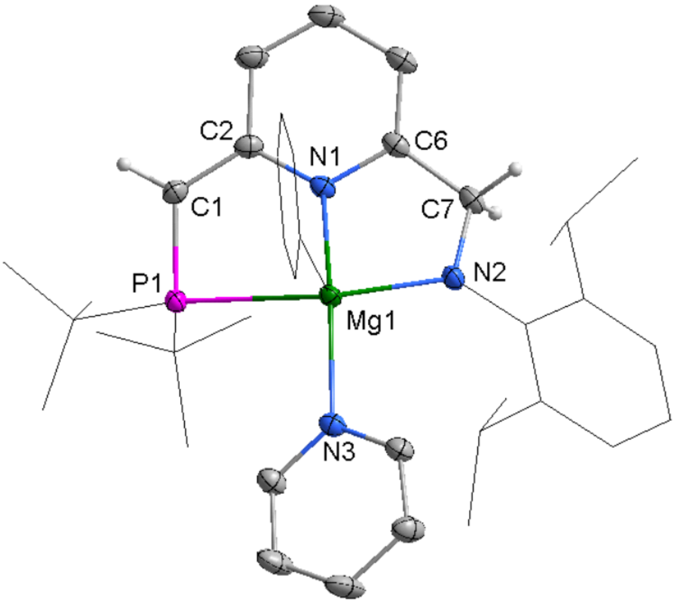

Figure S14. X-ray crystal structure of **Mg-2a**

Table S4. Crystal data and structure refinement for Mg-2a

Empirical formula	$C_{37}H_{51}MgN_4P$
Formula weight	607.09
Temperature/K	100.00(10)
Crystal system	monoclinic
Space group	*P2₁/c*
a/Å	17.1498(5)
b/Å	18.5251(6)
c/Å	22.8967(8)
α/°	90

β/°	93.315(3)
γ/°	90
Volume/Å^3	7262.1(4)
Z	8
ρ_{calc}g/cm^3	1.111
μ/mm^{-1}	0.122
F(000)	2624.0
Crystal size/mm^3	0.383 × 0.24 × 0.102
Radiation	MoKα (λ = 0.71073)
2Θ range for data collection/°	3.24 to 61.016
Index ranges	-24 ≤ h ≤ 24, -26 ≤ k ≤ 26, -32 ≤ l ≤ 31
Reflections collected	127861
Independent reflections	22169 [R_{int} = 0.1199, R_{sigma} = 0.0671]
Data/restraints/parameters	22169/0/795
Goodness-of-fit on F^2	1.070
Final R indexes [I>=2σ (I)]	R_1 = 0.0470, wR_2 = 0.1124
Final R indexes [all data]	R_1 = 0.0683, wR_2 = 0.1195
Largest diff. peak/hole / e Å^{-3}	0.42/-0.47

Table S5. Bond lengths for Mg-2a

Atom	Atom	Length/Å	Atom	Atom	Length/Å
P1	Mg1	2.7644(5)	C8	C11	1.5336(18)
P1	C1	1.7695(11)	C12	C13	1.5374(19)
P1	C8	1.8979(12)	C12	C14	1.5328(17)
P1	C12	1.8890(11)	C12	C15	1.5327(18)
Mg1	N1	2.1086(10)	C16	C17	1.4265(15)
Mg1	N2	2.0129(10)	C16	C21	1.4277(16)
Mg1	N3	2.2189(10)	C17	C18	1.3917(16)
Mg1	N4	2.2006(10)	C17	C22	1.5261(16)
N1	C2	1.3814(14)	C18	C19	1.3890(18)
N1	C6	1.3490(14)	C19	C20	1.3892(18)
N2	C7	1.4563(14)	C20	C21	1.3896(17)
N2	C16	1.4009(14)	C21	C25	1.5278(17)
N3	C28	1.3471(15)	C22	C23	1.5299(17)

N3	C32	1.3425(14)	C22	C24	1.5307(17)
N4	C33	1.3382(15)	C25	C26	1.5293(18)
N4	C37	1.3308(16)	C25	C27	1.5294(19)
C1	C2	1.3857(16)	C28	C29	1.3877(16)
C2	C3	1.4418(15)	C29	C30	1.3855(18)
C3	C4	1.3616(18)	C30	C31	1.3813(19)
C4	C5	1.4090(18)	C31	C32	1.3842(17)
C5	C6	1.3783(15)	C33	C34	1.3848(18)
C6	C7	1.5052(16)	C34	C35	1.365(2)
C8	C9	1.5404(16)	C35	C36	1.376(2)
C8	C10	1.5396(17)	C36	C37	1.3851(19)

Table S6. Bond angles for Mg-2a

Atom	Atom	Atom	Angle/°	Atom	Atom	Atom	Angle/°
C1	P1	Mg1	92.73(4)	C9	C8	P1	104.01(8)
C1	P1	C8	106.64(5)	C10	C8	P1	111.45(8)
C1	P1	C12	102.88(5)	C10	C8	C9	107.08(10)
C8	P1	Mg1	115.46(4)	C11	C8	P1	114.56(9)
C12	P1	Mg1	124.42(4)	C11	C8	C9	108.21(10)
C12	P1	C8	110.35(5)	C11	C8	C10	110.96(10)
N1	Mg1	P1	73.99(3)	C13	C12	P1	104.86(8)
N1	Mg1	N3	167.79(4)	C14	C12	P1	113.21(8)
N1	Mg1	N4	96.91(4)	C14	C12	C13	108.73(11)
N2	Mg1	P1	143.57(3)	C15	C12	P1	113.37(8)
N2	Mg1	N1	81.13(4)	C15	C12	C13	108.34(11)
N2	Mg1	N3	100.31(4)	C15	C12	C14	108.12(10)
N2	Mg1	N4	107.09(4)	N2	C16	C17	121.29(10)
N3	Mg1	P1	98.85(3)	N2	C16	C21	120.56(10)
N4	Mg1	P1	102.03(3)	C17	C16	C21	118.10(11)
N4	Mg1	N3	94.25(4)	C16	C17	C22	120.42(10)
C2	N1	Mg1	123.41(7)	C18	C17	C16	119.94(10)
C6	N1	Mg1	111.19(7)	C18	C17	C22	119.57(10)
C6	N1	C2	120.95(9)	C19	C18	C17	121.37(11)

C7	N2	Mg1	113.65(7)	C18	C19	C20	119.16(11)
C16	N2	Mg1	134.34(7)	C19	C20	C21	121.61(11)
C16	N2	C7	111.99(8)	C16	C21	C25	119.91(11)
C28	N3	Mg1	118.89(7)	C20	C21	C16	119.77(11)
C32	N3	Mg1	123.99(8)	C20	C21	C25	120.29(11)
C32	N3	C28	117.09(10)	C17	C22	C23	112.16(10)
C33	N4	Mg1	123.79(8)	C17	C22	C24	111.22(10)
C37	N4	Mg1	119.57(8)	C23	C22	C24	110.51(11)
C37	N4	C33	116.33(11)	C21	C25	C26	114.11(12)
C2	C1	P1	120.45(9)	C21	C25	C27	110.14(10)
N1	C2	C1	119.44(10)	C26	C25	C27	110.55(10)
N1	C2	C3	117.44(10)	N3	C28	C29	123.26(10)
C1	C2	C3	123.12(11)	C30	C29	C28	118.57(12)
C4	C3	C2	120.32(11)	C31	C30	C29	118.86(12)
C3	C4	C5	120.63(11)	C30	C31	C32	118.94(11)
C6	C5	C4	117.68(11)	N3	C32	C31	123.28(12)
N1	C6	C5	122.82(11)	N4	C33	C34	124.30(12)
N1	C6	C7	114.27(9)	C35	C34	C33	118.44(12)
C5	C6	C7	122.84(10)	C34	C35	C36	118.35(13)
N2	C7	C6	112.56(9)	C35	C36	C37	119.62(14)

2.3 Synthesis and characterization of Mg-2b

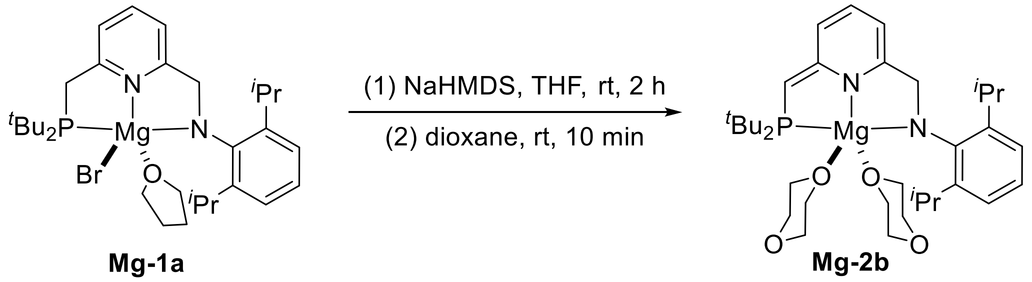

In a N_2 glovebox, **Mg-1a** (180.4 mg, 0.3 mmol) was dissolved by THF in a 20 mL vial. NaHMDS (55.0 mg, 0.3 mmol) was added, and the resulting solution was stirred at room temperature for 2 h, then filtered through a small piece of cotton to remove the solid. The orange solution was concentrated under vacuum to afford a thick oil. The thick oil was dissolved by dioxane (0.5 mL) and then kept at room temperature for 10

min. Pentane (about 1 mL) was slowly added to the vial, and the resulting solution was kept at room temperature until yellow crystals formed. The crystals were suitable for X-ray diffraction. The solvent was decanted, and the solid was washed with pentane and then dried under vacuum to give **Mg-2b** as a yellow solid (146.6 mg, 78% yield).

Mg-2b can be also prepared by the reaction of **L1** with $^{n}Bu_2Mg$ in dioxane:

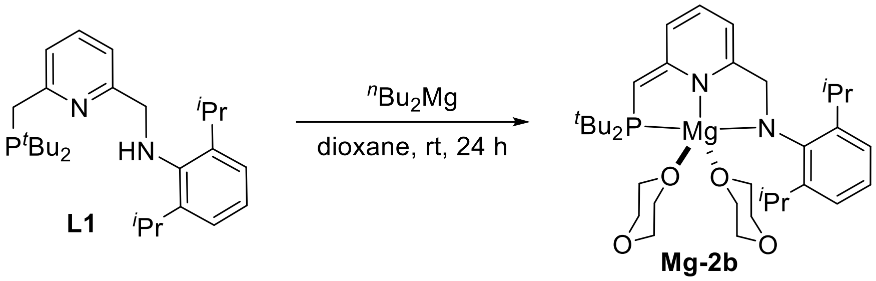

To a dioxane (0.5 mL) solution of **L1** (213.2 mg, 0.5 mmol) was added $^{n}Bu_2Mg$ (0.5 M in heptane, 1 mL, 0.5 mmol) at room temperature. The resulting solution was stirred at the same temperature for 24 h. Pentane (2 mL) was added to the solution, and a yellow precipitate was slowly formed. The mixture was kept at room temperature for 3 h. The solvent was decanted, and the solid was washed with pentane, and then dried under vacuum to give the pure product (285.0 mg, 91% yield).

^{1}H NMR (400 MHz, THF-d_8) δ 6.89 (d, J = 7.6 Hz, 2H, Ar***H***), 6.77 – 6.69 (m, 1H, Ar***H***), 6.35 (ddd, J = 8.2, 6.7, 1.2 Hz, 1H, Py***H***), 5.77 (d, J = 8.5 Hz, 1H, Py***H***), 5.09 (d, J = 6.6 Hz, 1H, Py***H***), 3.98 (s, 2H, NC***H***$_2$), 3.85 – 3.73 (m, 2H, C***H***$(CH_3)_2$), 3.55 (s, 16H, OC***H***$_2$), 3.16 (d, J = 5.0 Hz, 1H, PC***H***), 1.19 (d, J = 11.7 Hz, 18H, PC(C***H***$_3$)$_3$), 1.14 – 1.07 (m, 12H, CH(C***H***$_3$)$_2$).

^{13}C NMR (101 MHz, THF-d_8) δ 167.73 (d, J = 15.5 Hz, Py***C***), 161.09 (d, J = 3.3 Hz, Py***C***), 158.74 (s, Ar***C***), 147.68 (s, Ar***C***), 134.03 (d, J = 2.4 Hz, Py***C***), 123.08 (s, Ar***C***), 120.64 (s, Ar***C***), 113.07 (d, J = 8.3 Hz, Py***C***), 96.61 (s, Py***C***), 67.63 (s, O***C***H_2), 62.38 (s, N***C***H_2), 55.54 (d, J = 34.2 Hz, P***C***H), 32.44 (d, J = 8.6 Hz, PC(***C***H_3)$_3$), 33.74 (s, P***C***$(CH_3)_3$), 27.44 (s, ***C***$H(CH_3)_2$), 26.53 (s, CH(***C***H_3)$_2$), 24.72 (s, CH(***C***H_3)$_2$).

^{31}P NMR (162 MHz, THF-d_8) δ 3.35 (s).

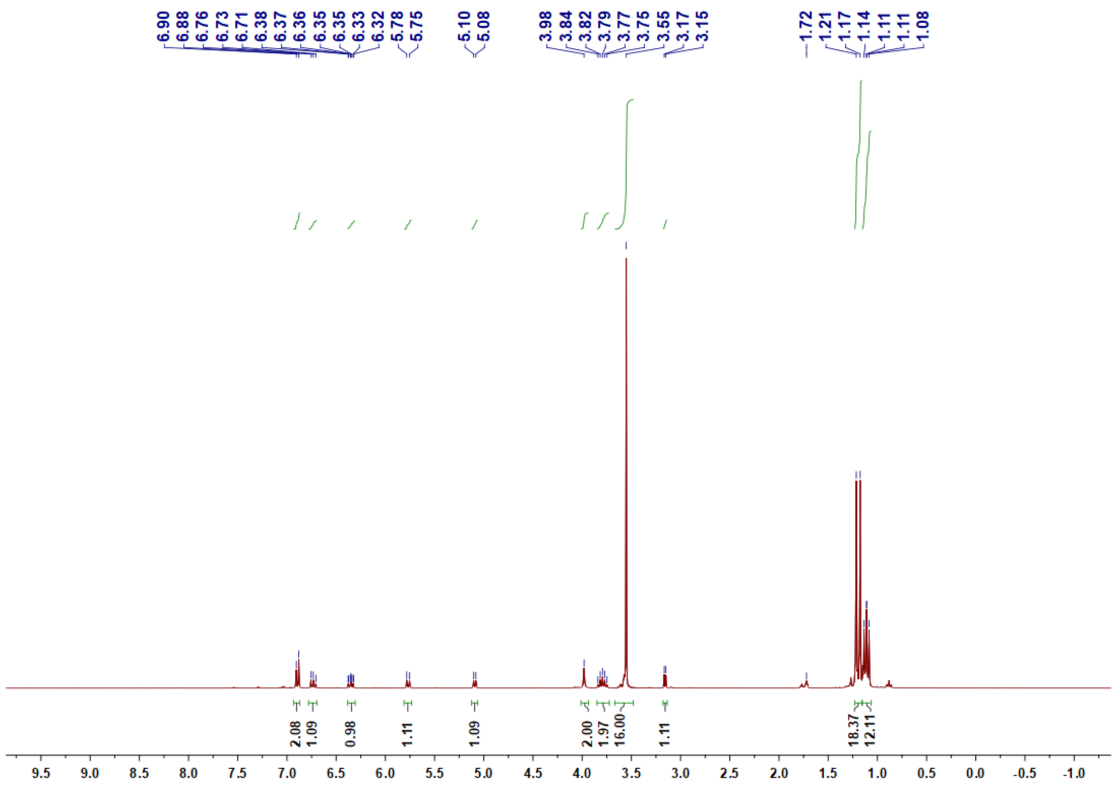

Figure S15. ^{1}H NMR (400 MHz, THF-d_8) spectrum of **Mg-2b**

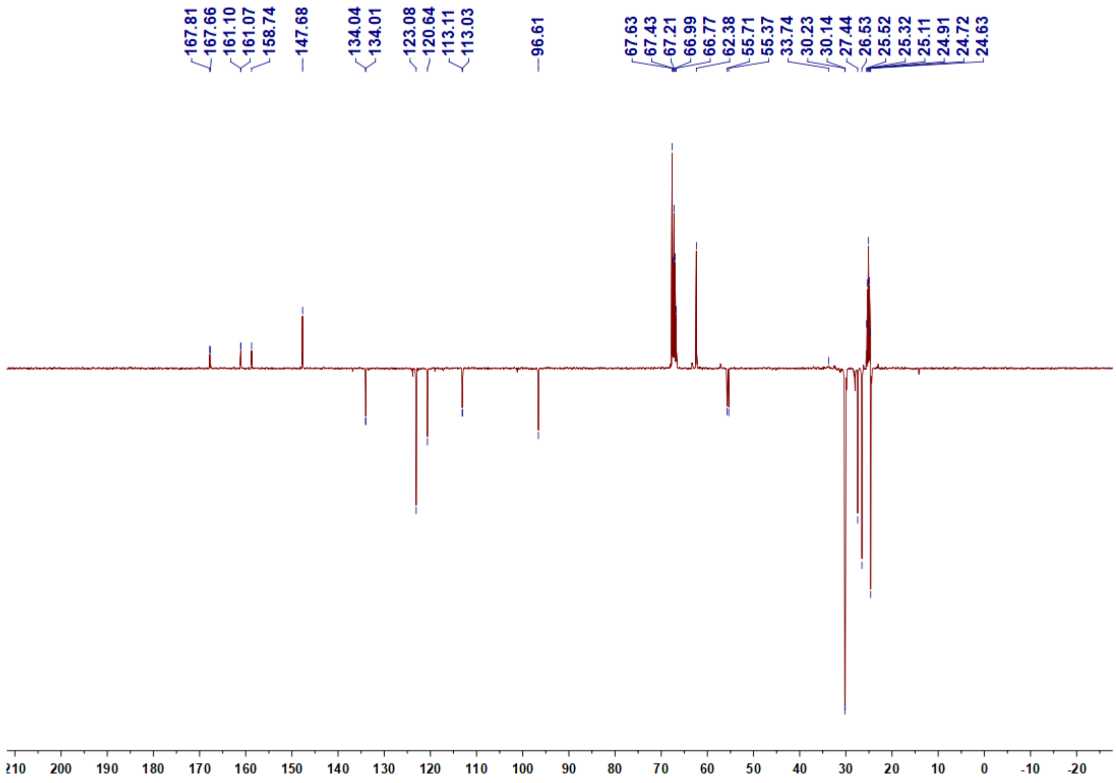

Figure S16. ^{13}C-DEPTQ NMR (101 MHz, THF-d_8) spectrum of **Mg-2b**

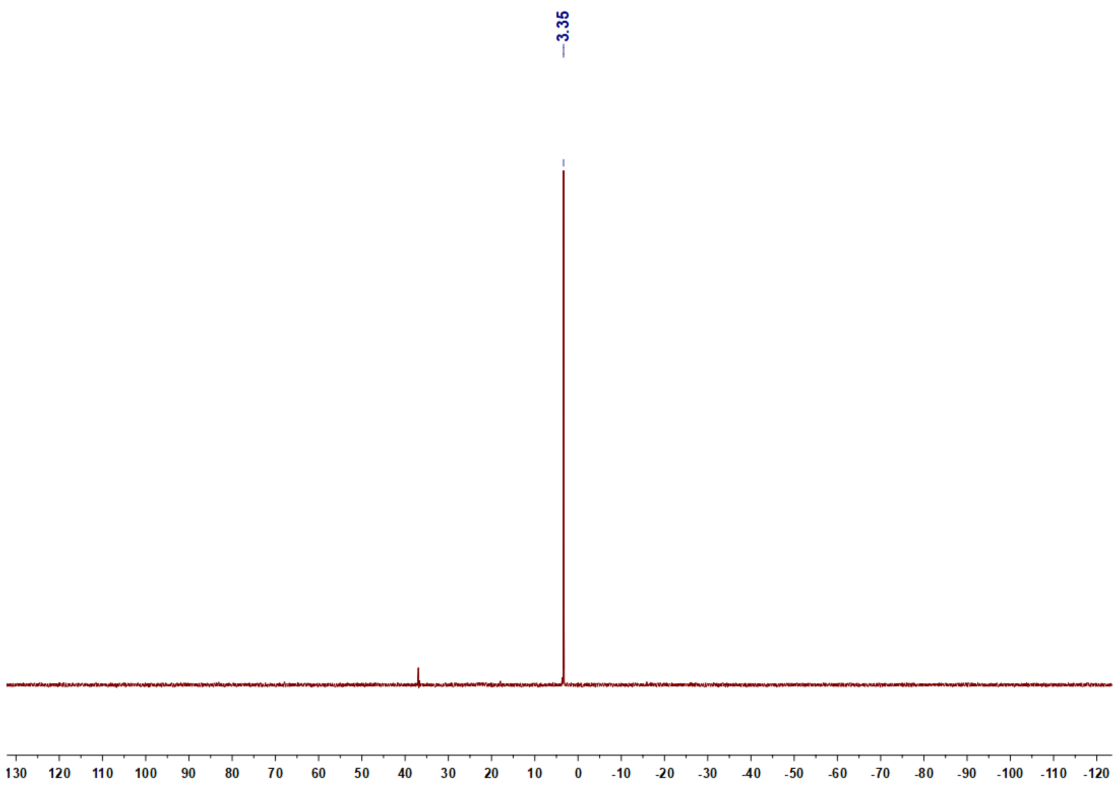

Figure S17. ^{31}P NMR (162 MHz, THF-d_8) spectrum of **Mg-2b**

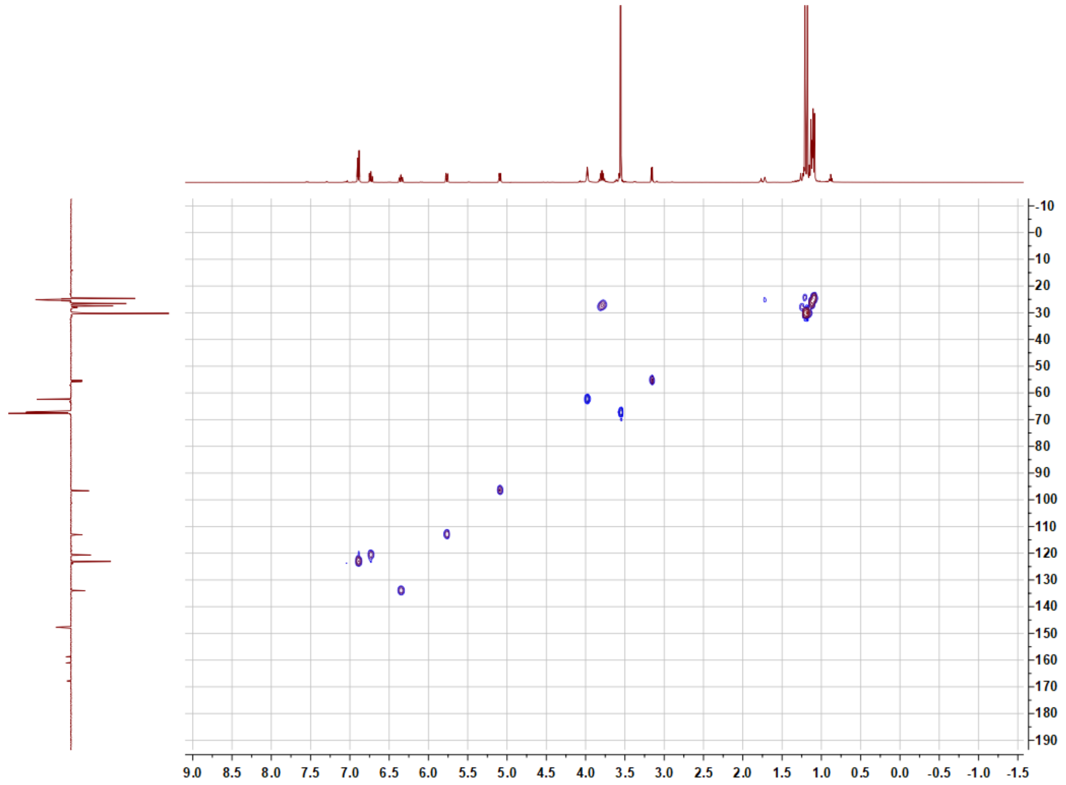

Figure S18. HSQC spectrum of **Mg-2b** in THF-d_8

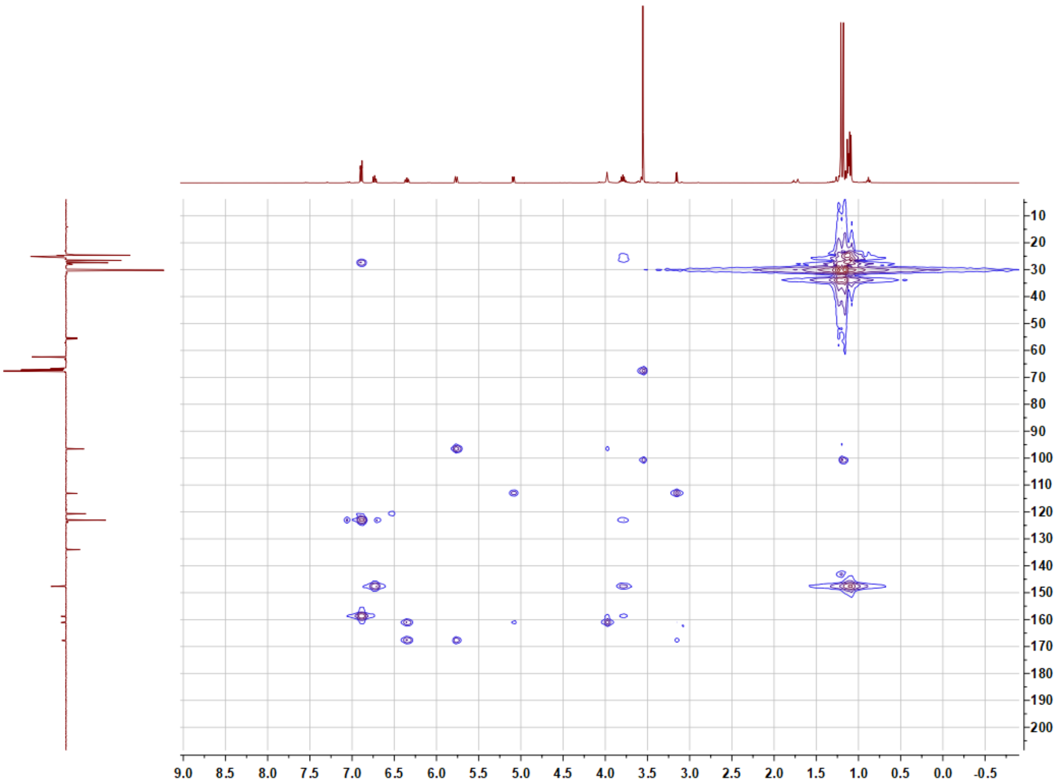

Figure S19. HMBC spectrum of **Mg-2b** in THF-d_8

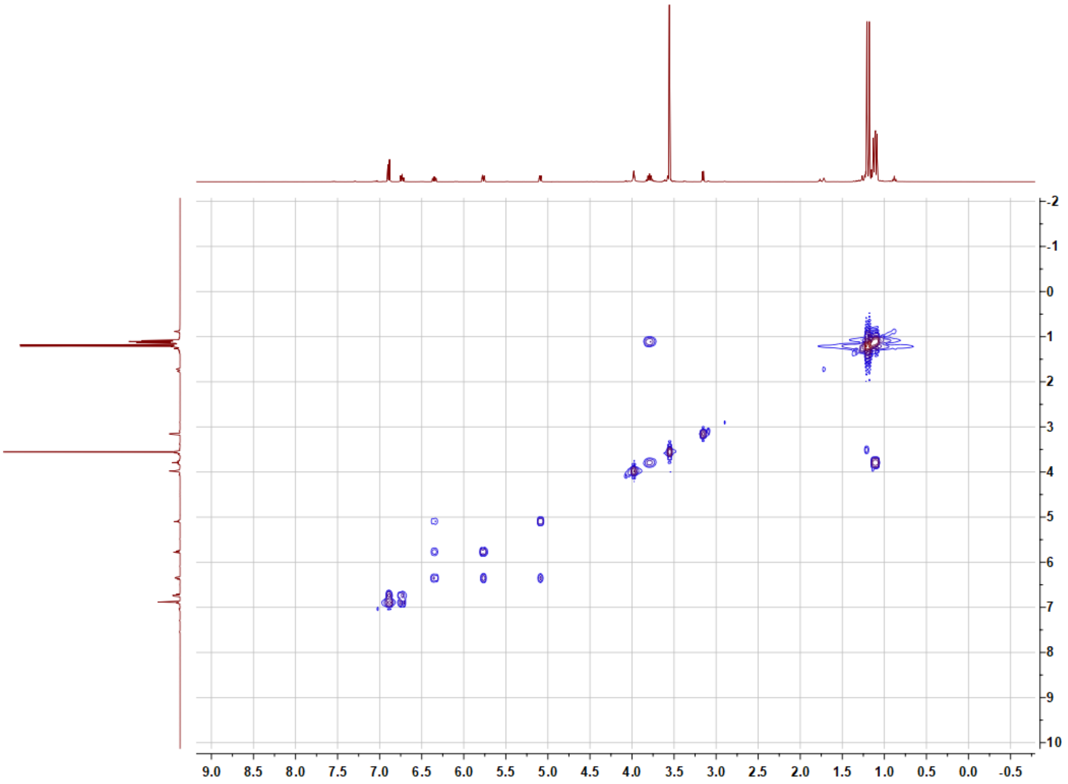

Figure S20. H-H COSY spectrum of **Mg-2b** in THF-d_8

The diffraction data from single crystals of **Mg-2b** were collected on Rigaku Synergy-S diffractometer dual source equipped with Dectris Pilatus3 R CdTe 300 K detector and microfocus, with MoKα (λ=0.71073Å). The data were processed with CrysAlis[PRO 2]. The structures were solved with SHELXT[3]. Full matrix least-squares and refined based on F^2 with SHELXL[4]. All structure solution and refinement programs are implemented in Olex-2 GUI[5]. All non-hydrogen atoms were refined with anisotropic displacement coefficients. Hydrogens were placed in calculated positions and refined in riding mode. Supplementary crystallographic data have been deposited at the Cambridge Crystallographic Data Center (CCDC 2237497).

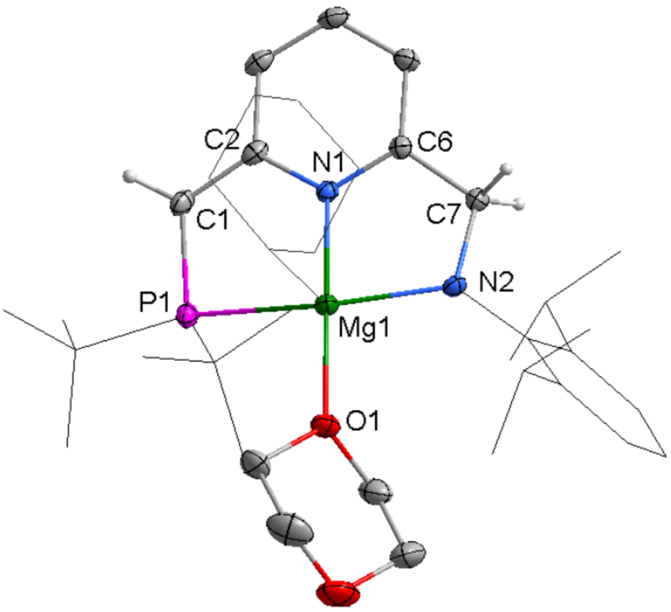

Figure S21. X-ray crystal structure of **Mg-2b**

Table S7. Crystal data and structure refinement for Mg-2b

Empirical formula	$C_{35}H_{57}MgN_2O_4P$
Formula weight	625.10
Temperature/K	100.0(2)
Crystal system	monoclinic
Space group	$P2_1/c$
a/Å	11.9052(4)
b/Å	17.3291(6)
c/Å	17.4860(6)
α/°	90

β/°	103.868(3)
γ/°	90
Volume/Å^3	3502.3(2)
Z	4
ρ_{calc}g/cm^3	1.186
μ/mm^{-1}	0.135
F(000)	1360.0
Crystal size/mm^3	0.248 × 0.16 × 0.134
Radiation	MoKα (λ = 0.71073)
2Θ range for data collection/°	4.236 to 67.596
Index ranges	-17 ≤ h ≤ 18, -26 ≤ k ≤ 23, -25 ≤ l ≤ 27
Reflections collected	71080
Independent reflections	12713 [R_{int} = 0.0548, R_{sigma} = 0.0477]
Data/restraints/parameters	12713/0/398
Goodness-of-fit on F^2	1.056
Final R indexes [I>=2σ (I)]	R_1 = 0.0460, wR_2 = 0.1112
Final R indexes [all data]	R_1 = 0.0694, wR_2 = 0.1193
Largest diff. peak/hole / e Å^{-3}	0.50/-0.25

Table S8. Bond lengths for Mg-2b

Atom	Atom	Length/Å	Atom	Atom	Length/Å
P1	Mg1	2.7335(4)	C5	C6	1.3831(14)
P1	C1	1.7676(11)	C6	C7	1.5155(14)
P1	C8	1.8865(12)	C8	C9	1.5373(18)
P1	C12	1.9030(11)	C8	C10	1.5367(18)
Mg1	O1	2.0991(8)	C8	C11	1.5323(18)
Mg1	O3	2.0918(8)	C12	C13	1.5304(16)
Mg1	N1	2.0700(9)	C12	C14	1.5366(16)
Mg1	N2	2.0151(9)	C12	C15	1.5330(16)
Mg1	C6	2.8496(10)	C16	C17	1.4225(14)
O1	C28	1.4468(14)	C16	C21	1.4268(15)
O1	C31	1.4483(14)	C17	C18	1.3960(15)
O2	C29	1.4148(19)	C17	C22	1.5146(16)
O2	C30	1.4218(18)	C18	C19	1.3828(17)

O3	C32	1.4468(14)	C19	C20	1.3881(17)
O3	C35	1.4511(13)	C20	C21	1.3972(15)
O4	C33	1.4229(16)	C21	C25	1.5198(15)
O4	C34	1.4274(15)	C22	C23	1.5324(17)
N1	C2	1.3841(13)	C22	C24	1.5336(17)
N1	C6	1.3466(13)	C25	C26	1.5332(18)
N2	C7	1.4616(13)	C25	C27	1.5322(17)
N2	C16	1.4175(13)	C28	C29	1.4962(19)
C1	C2	1.3967(15)	C30	C31	1.4945(19)
C2	C3	1.4358(15)	C32	C33	1.5119(19)
C3	C4	1.3682(16)	C34	C35	1.5083(16)
C4	C5	1.4083(15)			

Table S9. Bond angles for Mg-2b

Atom	Atom	Atom	Angle/°	Atom	Atom	Atom	Angle/°
C1	P1	Mg1	92.41(4)	N1	C6	C7	115.90(9)
C1	P1	C8	105.63(5)	C5	C6	Mg1	157.85(8)
C1	P1	C12	107.36(5)	C5	C6	C7	121.62(9)
C8	P1	Mg1	125.81(4)	C7	C6	Mg1	76.25(5)
C8	P1	C12	109.45(5)	N2	C7	C6	113.48(8)
C12	P1	Mg1	112.95(4)	C9	C8	P1	106.22(8)
P1	Mg1	C6	100.82(2)	C10	C8	P1	114.35(10)
O1	Mg1	P1	101.97(3)	C10	C8	C9	107.16(11)
O1	Mg1	C6	155.71(3)	C11	C8	P1	111.06(9)
O3	Mg1	P1	100.03(3)	C11	C8	C9	108.28(11)
O3	Mg1	O1	90.91(3)	C11	C8	C10	109.49(11)
O3	Mg1	C6	93.31(3)	C13	C12	P1	115.42(8)
N1	Mg1	P1	75.03(3)	C13	C12	C14	107.92(10)
N1	Mg1	O1	174.92(4)	C13	C12	C15	109.89(10)
N1	Mg1	O3	93.66(3)	C14	C12	P1	105.45(7)
N1	Mg1	C6	26.13(3)	C15	C12	P1	109.57(8)
N2	Mg1	P1	143.65(3)	C15	C12	C14	108.28(10)
N2	Mg1	O1	97.54(4)	N2	C16	C17	121.65(9)

N2	Mg1	O3	110.08(4)	N2	C16	C21	120.82(9)
N2	Mg1	N1	83.00(4)	C17	C16	C21	117.48(9)
N2	Mg1	C6	58.61(3)	C16	C17	C22	121.46(9)
C28	O1	Mg1	127.22(7)	C18	C17	C16	120.36(10)
C28	O1	C31	110.01(9)	C18	C17	C22	118.17(10)
C31	O1	Mg1	115.85(7)	C19	C18	C17	121.46(11)
C29	O2	C30	109.17(10)	C18	C19	C20	119.02(10)
C32	O3	Mg1	118.51(7)	C19	C20	C21	121.47(11)
C32	O3	C35	109.83(9)	C16	C21	C25	121.81(9)
C35	O3	Mg1	126.41(6)	C20	C21	C16	120.05(10)
C33	O4	C34	108.28(9)	C20	C21	C25	118.14(10)
C2	N1	Mg1	124.35(7)	C17	C22	C23	111.75(10)
C6	N1	Mg1	111.27(7)	C17	C22	C24	111.73(10)
C6	N1	C2	121.76(9)	C23	C22	C24	109.31(10)
C7	N2	Mg1	111.59(6)	C21	C25	C26	110.48(10)
C16	N2	Mg1	135.79(7)	C21	C25	C27	113.43(10)
C16	N2	C7	111.27(8)	C27	C25	C26	108.52(10)
C2	C1	P1	119.87(8)	O1	C28	C29	109.99(11)
N1	C2	C1	119.09(9)	O2	C29	C28	111.91(11)
N1	C2	C3	117.10(9)	O2	C30	C31	110.70(11)
C1	C2	C3	123.80(9)	O1	C31	C30	110.28(11)
C4	C3	C2	120.11(10)	O3	C32	C33	109.64(10)
C3	C4	C5	121.20(10)	O4	C33	C32	110.63(11)
C6	C5	C4	117.27(10)	O4	C34	C35	110.88(10)
N1	C6	Mg1	42.61(5)	O3	C35	C34	111.09(9)
N1	C6	C5	122.45(9)				

2.4 Synthesis and characterization of Mg-2c

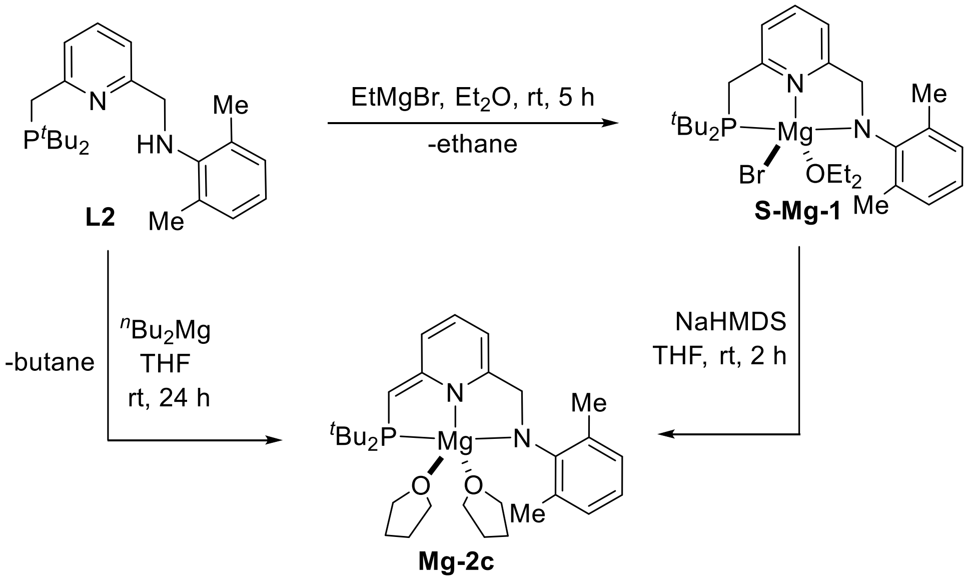

Two-step procedures:

In a N_2 glovebox, **L2** (185.1 mg, 0.5 mmol) was dissolved in a 20 mL vial by Et_2O (3 mL). EtMgBr (3.0 M in Et_2O, 170 μL, 0.5 mmol) was slowly dropped into the solution at room temperature. The resulting solution was stirred at room temperature for 5 h to afford a yellowish precipitate. The solvent was decanted, and the solid was washed with Et_2O three times and then dried under vacuum to give **S-Mg-1** as a yellowish solid (232.1 mg, 85% yield). THF (2 mL) was added to dissolve the solid, and NaHMDS (77.9 mg, 0.425 mmol) was added to the solution. The resulting solution was stirred at room temperature for 2 h. The generated solid was removed by filtration, affording an orange-red solution, which was concentrated to give **Mg-2c** as an orange solid. To further purify the complex, the solid was dissolved in THF (1 mL) and pentane (2 mL). The solution was kept at room temperature until crystals formed. The solvent was decanted, and the solid was dried under vacuum to give **Mg-2c** as a pure product (180.1 mg, 79% yield).

One-step procedures:

To a THF (0.5 mL) solution of **L2** (111.1 mg, 0.3 mmol) was added ^{n}Bu$_2$Mg (0.5 M in heptane, 0.6 mL, 0.3 mmol) at room temperature. The resulting solution was stirred at the same temperature for 24 h. The solvent was removed to provide the crude product,

which was purified by recrystallization with a mixture solvent of THF and pentane at room temperature (103.0 mg, 64% yield).

^{1}H NMR (400 MHz, C_6D_6) δ 7.26 (d, *J* = 7.3 Hz, 2H, Ar***H***), 6.94 (t, *J* = 7.3 Hz, 1H, Ar***H***), 6.85 – 6.78 (m, 1H, Py**H**), 6.37 (d, *J* = 8.5 Hz, 1H, Py**H**), 5.64 (d, *J* = 6.7 Hz, 1H, Py**H**), 4.43 (s, 2H, NC$\boldsymbol{H}_2$), 3.55 (d, *J* = 5.2 Hz, 1H, PC***H***), 3.50 (t, *J* = 6.5 Hz, 8H, OC$\boldsymbol{H}_2$), 2.46 (s, 6H, ArC$\boldsymbol{H}_3$), 1.24 (d, *J* = 11.9 Hz, 18H, PC(C$\boldsymbol{H}_3$)$_3$), 1.18 – 1.13 (m, 8H, OCH$_2$C$\boldsymbol{H}_2$).

^{13}C NMR (101 MHz, C_6D_6) δ 167.86 (d, *J* = 15.1 Hz, Py***C***), 161.45 (d, *J* = 3.1 Hz, Py***C***), 161.05 (s, Ar***C***), 135.28 (s, Ar***C***), 134.83 (s, Py***C***), 128.55 (s, Ar***C***), 118.78 (s, Ar***C***), 113.23 (d, *J* = 8.4 Hz, Py***C***), 97.59 (s, Py***C***), 69.50 (s, O***C***H$_2$CH$_2$), 58.60 (s, N***C***H$_2$), 55.03 (d, *J* = 34.6 Hz, P***C***H), 33.57 (d, *J* = 4.0 Hz, P***C***(CH$_3$)$_3$), 30.25 (d, *J* = 8.5 Hz, PC(***C***H$_3$)$_3$), 25.06 (s, OCH$_2$***C***H$_2$), 20.46 (s, Ar***C***H$_3$).

^{31}P NMR (162 MHz, THF) δ 4.04 (s).

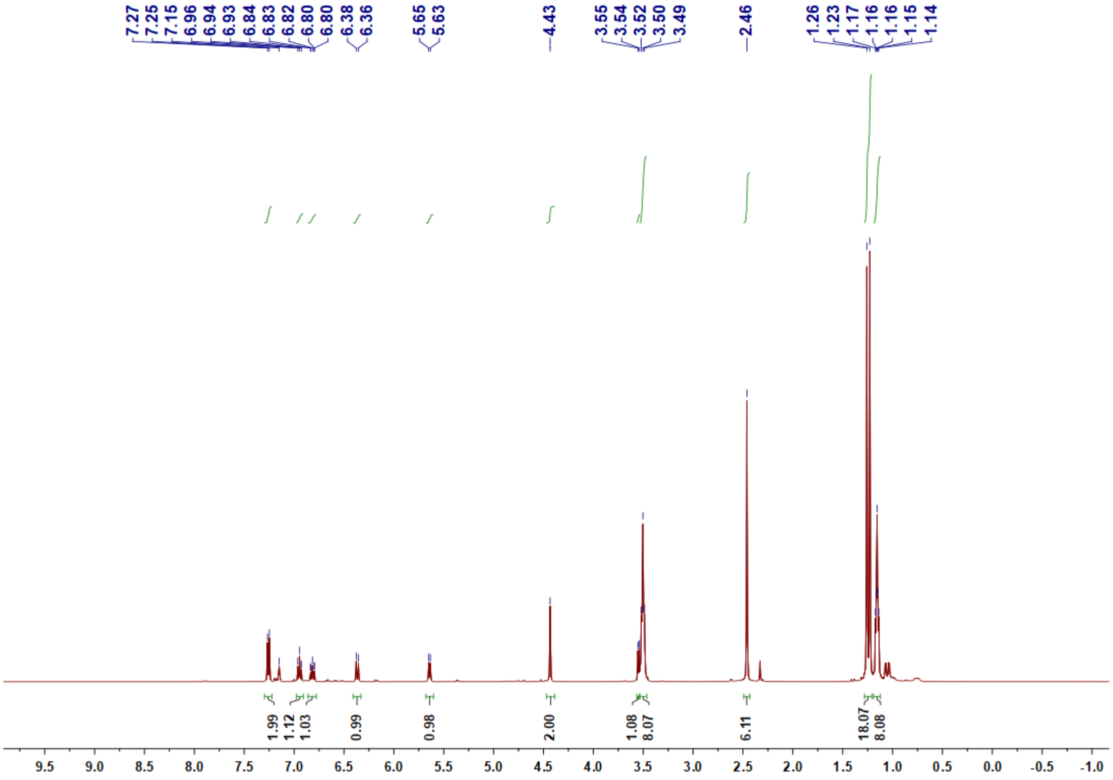

Figure S22. ^{1}H NMR (400 MHz, C_6D_6) spectrum of **Mg-2c**

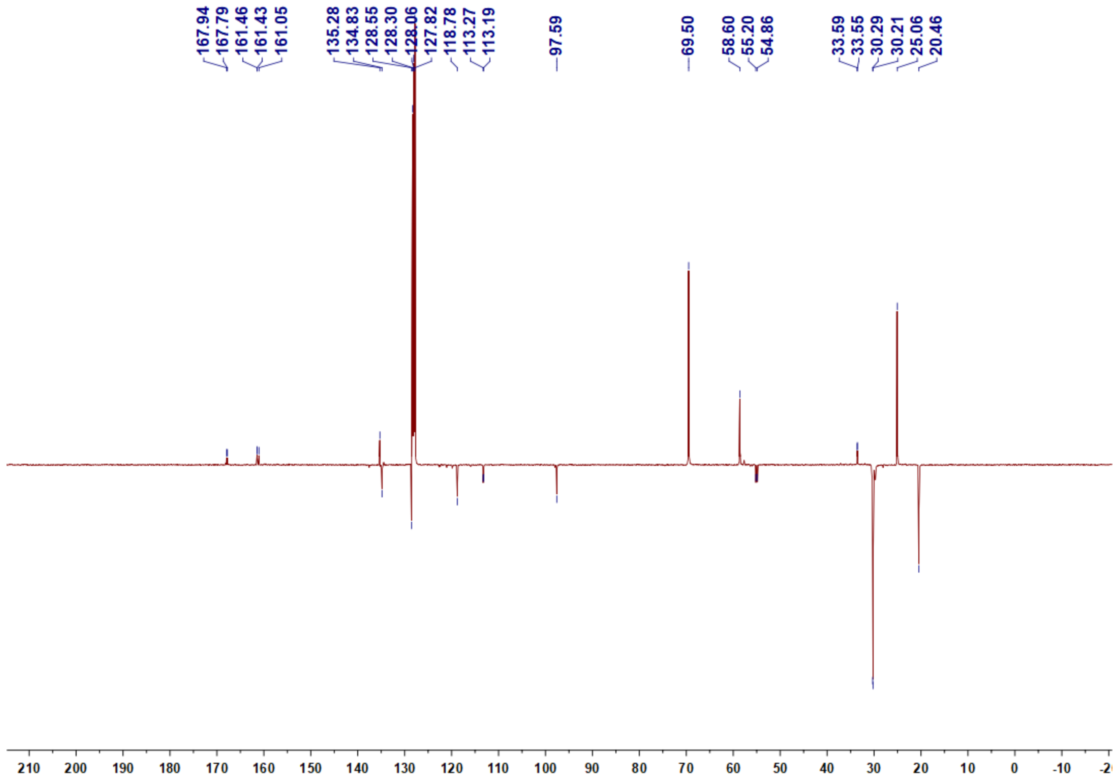

Figure S23. ^{13}C-DEPTQ NMR (101 MHz, C_6D_6) spectrum of **Mg-2c**

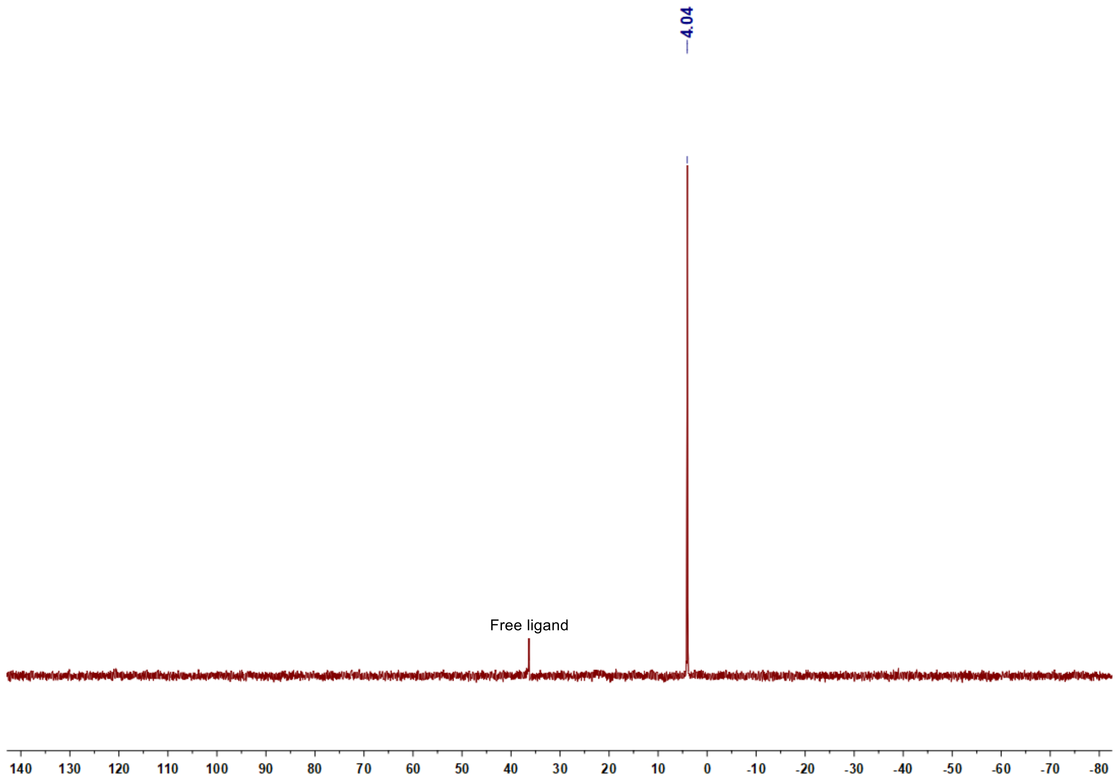

Figure S24. ^{31}P NMR (162 MHz, C_6D_6) spectrum of **Mg-2c**

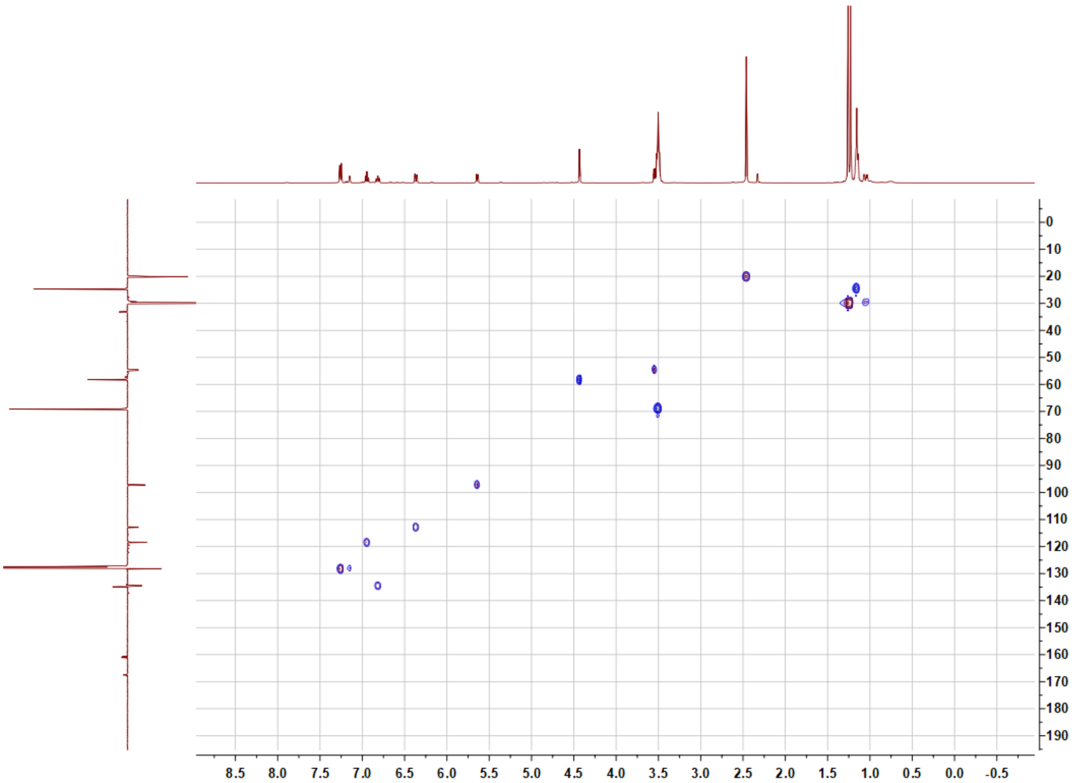

Figure S25. HSQC spectrum of **Mg-2c** in C_6D_6

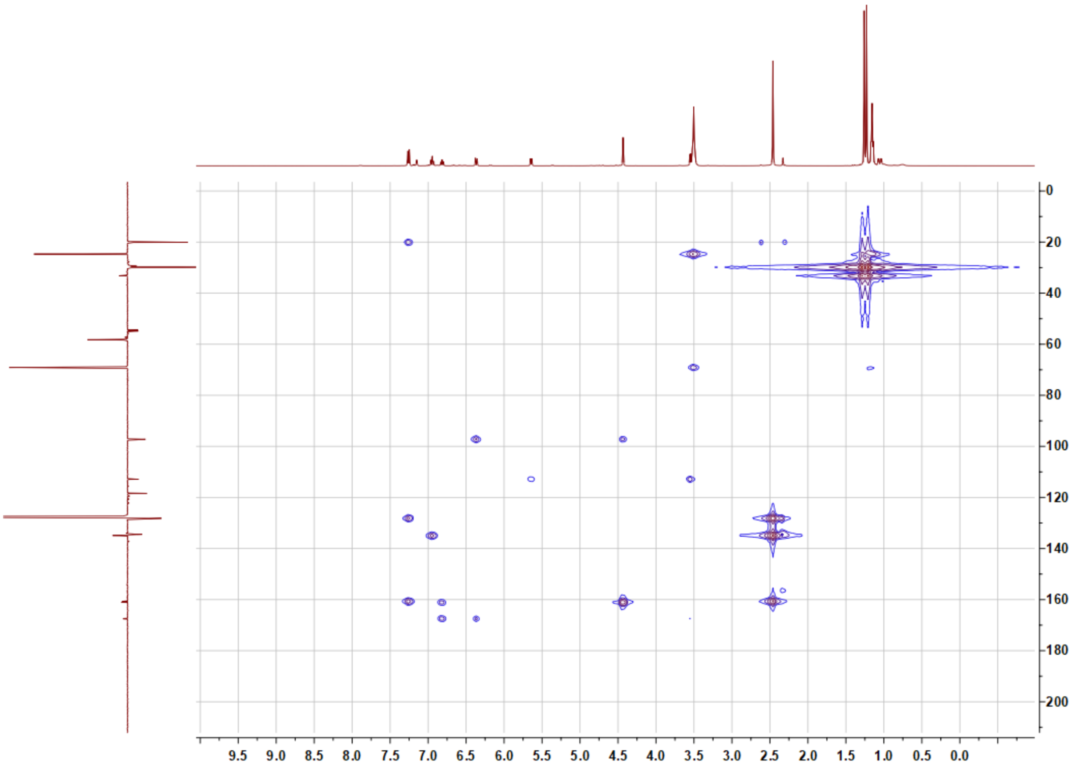

Figure S26. HMBC spectrum of **Mg-2c** in C_6D_6

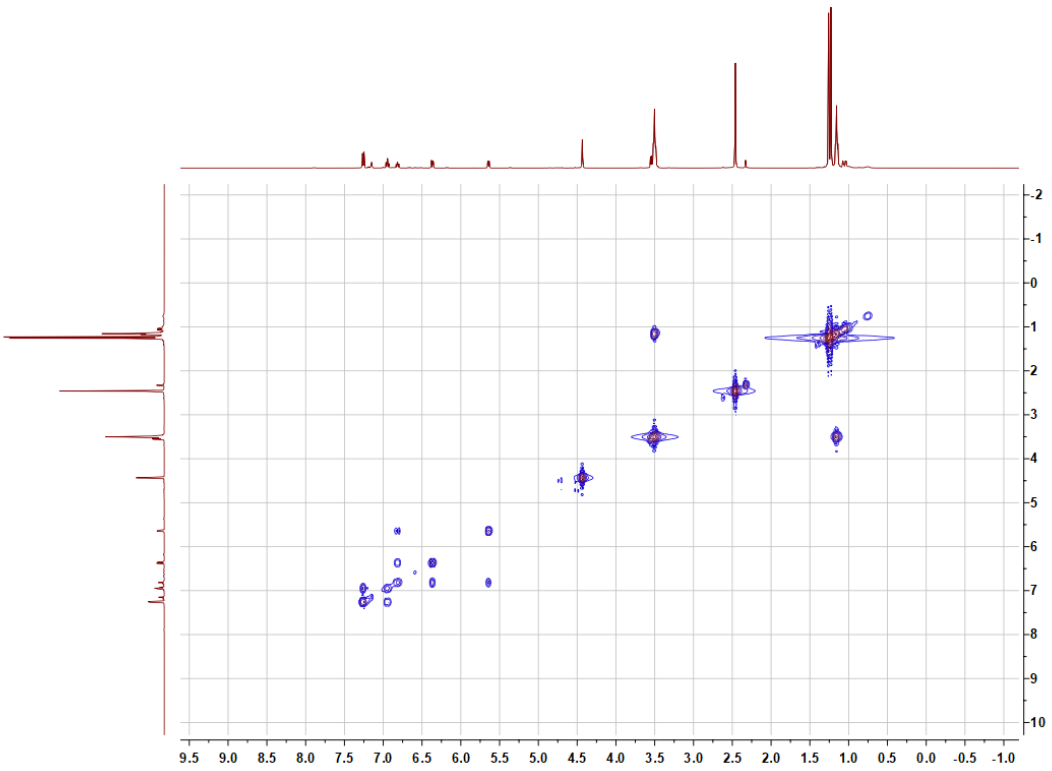

Figure S27. H-H COSY spectrum of **Mg-2c** in C_6D_6

Preparation of ***L2****:*

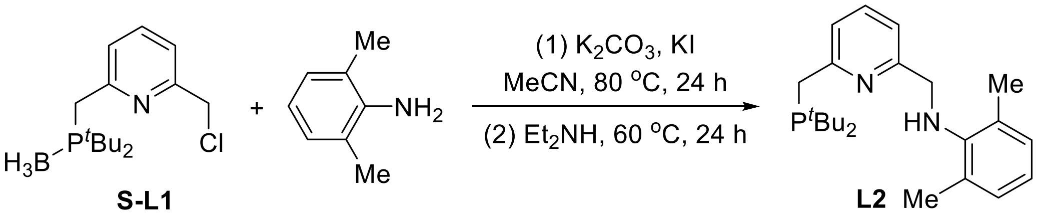

To a 100 mL flask were added K_2CO_3 (1382.1 mg, 10 mmol) and KI (33.2 mg, 0.2 mmol). **S-L1** (598.4 mg, 2 mmol) and 2,6-dimethylaniline (254.5 mg, 2.1 mmol) were dissolved by MeCN (50 mL) in a small vial, and the resulting solution was then added to the flask. The solution was heated at 80 °C for 24 h. After cooling to room temperature, water was added to dissolve the formed solid. The resulting mixture was extracted with Et_2O three times. The combined organic layers were dried over Na_2SO_4, filtered, and concentrated under reduced pressure. The residue was purified by flash silica gel column chromatography to afford the borane-protected ligand. The obtained product was dissolved in degassed Et_2NH under nitrogen and then heated at 60 °C for 24 h. After cooling to room temperature, all volatiles were removed under vacuum. The

residue was passed through a short silica column chromatography in the glovebox to afford the desired **L2** as a colorless oil. Compound **S-L1** was prepared according to the literature procedure.[1]

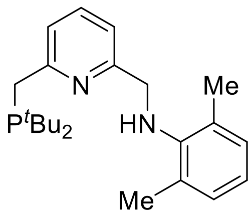

^{1}H NMR (300 MHz, $CDCl_3$) δ 7.52 (t, *J* = 7.7 Hz, 1H), 7.33 (d, *J* = 7.7 Hz, 1H), 6.99 (d, *J* = 7.2 Hz, 3H), 6.80 (t, *J* = 7.4 Hz, 1H), 4.52 (t, *J* = 5.8 Hz, 1H), 4.25 (d, *J* = 6.0 Hz, 2H), 3.09 (d, *J* = 3.3 Hz, 2H), 2.35 (s, 6H), 1.18 (d, *J* = 11.0 Hz, 18H).

^{13}C NMR (75 MHz, $CDCl_3$) δ 161.70 (d, *J* = 14.2 Hz), 157.76 (s), 146.71 (s), 136.56 (s), 129.01 (d, *J* = 10.0 Hz), 128.87 (s), 122.30 (d, *J* = 9.2 Hz), 121.49 (s), 118.73 (d, *J* = 1.6 Hz), 53.59 (s), 31.80 (d, *J* = 24.2 Hz), 29.80 (d, *J* = 13.3 Hz), 28.30 (d, *J* = 1.2 Hz), 19.02 (s).

^{31}P NMR (121 MHz, $CDCl_3$) δ 38.08.

3 Bond activation by magnesium pincer complexes

3.1 Reaction of Mg-2a and Mg-2b with H_2

A toluene solution (0.6 mL) of **Mg-2a** (31.1 mg, 0.05 mmol) was charged with 5 bar of H_2 in a J. Young NMR tube. **Mg-2a** was completely transformed into a dimer complex **Mg-3** (36% yield after recrystallization) after heating at 120 °C for 72 h. Similarly, **Mg-2b** (31.2 mg, 0.05 mmol) can fully convert into the dimer complex **Mg-3** (41% yield after recrystallization) under 5 bar of H_2 after heating at 120 °C for 48 h. These results suggested that the dearomatized complexes **Mg-2a** and **Mg-2b** may undergo the activation of H_2 by MLC. It is noted that, in these two reactions, white precipitates were generated as the byproduct. A broad signal could be observed at 544 cm^{-1} by IR (Figure S28), indicating that MgH_2 was the major byproduct in these reactions.[6]

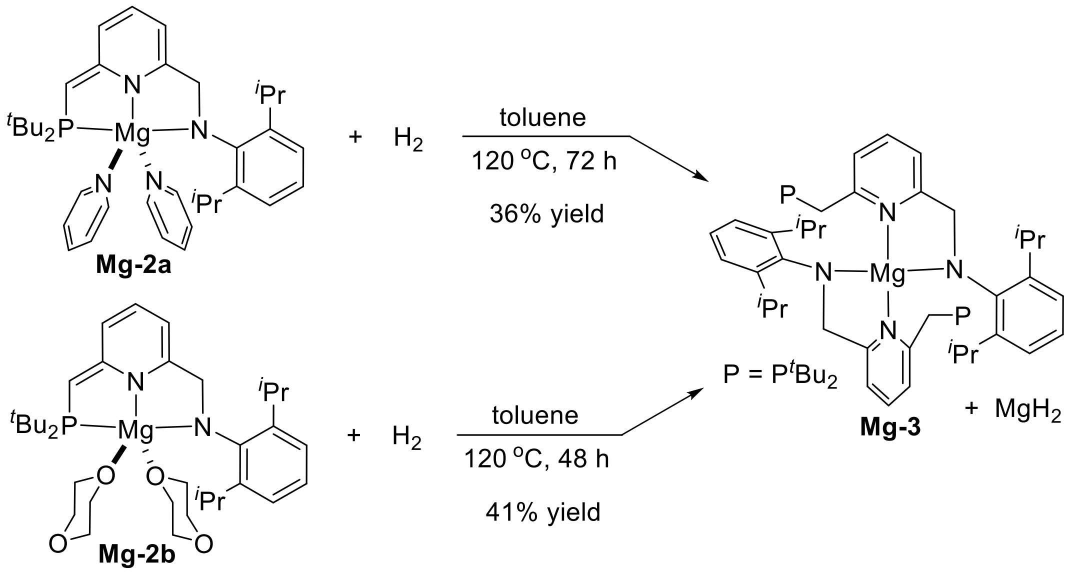

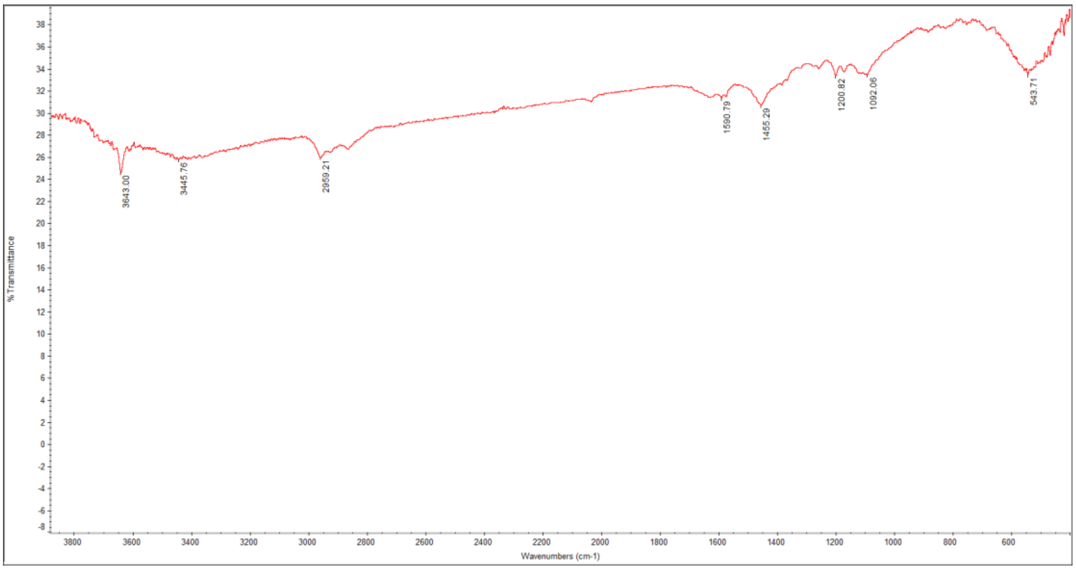

Figure S28. IR spectrum of the precipitate

The conversion of **Mg-2a** into **Mg-3** under different temperatures and reaction times was studied to get a deeper understanding of **Mg-3** formation. The ^{31}P NMR spectra suggested that **Mg-3** was generated as the major product (Figure S29).

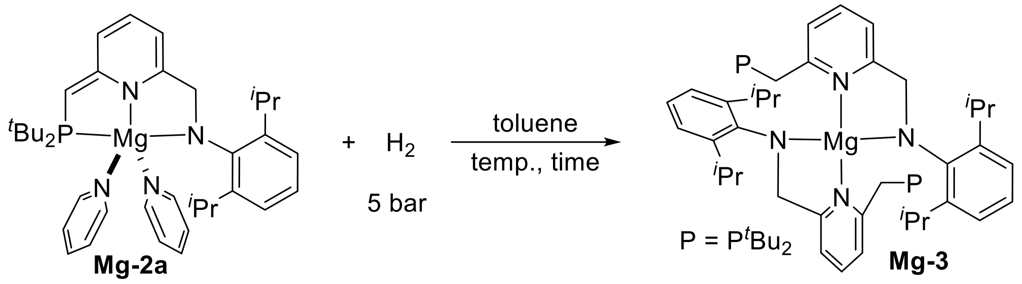

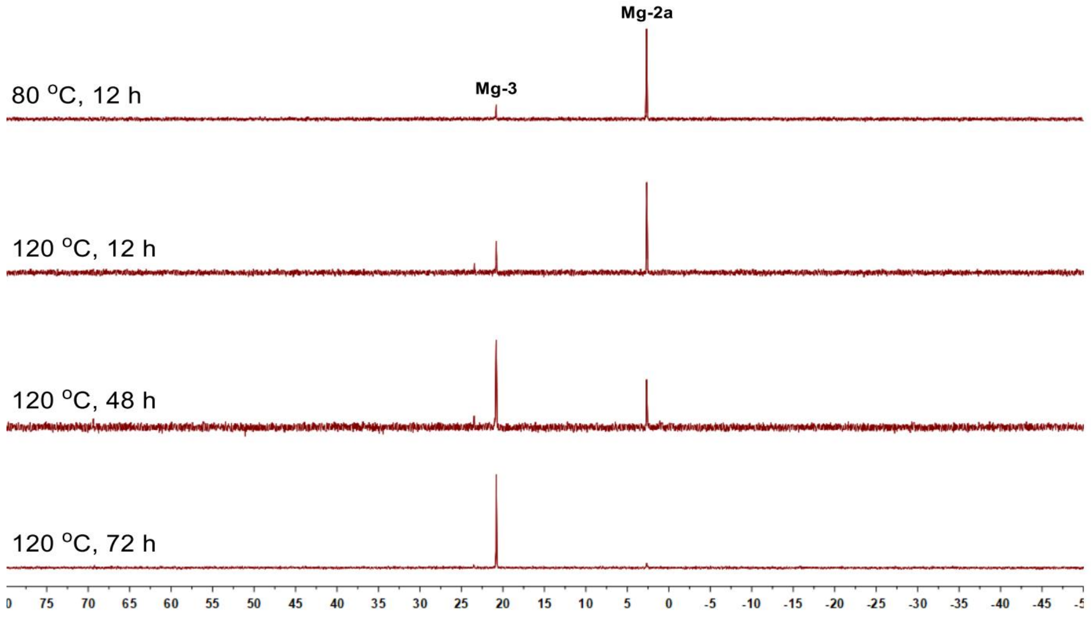

Figure S29. ^{31}P NMR spectra of converting **Mg-2a** to **Mg-3** under different conditions

Characterization of ***Mg-3***

^{1}H NMR (400 MHz, C_6D_6) δ 7.96 – 7.88 (m, 1H, Py***H***), 7.29 – 7.09 (m, 3H, Ar***H***, overlap with residue solvent), 6.93 (t, *J* = 7.8 Hz, 1H, Py***H***), 6.55 (d, *J* = 7.7 Hz, 1H, Py***H***), 4.97 (d, *J* = 22.2 Hz, 1H, NC***H***$_2$), 4.59 (d, *J* = 22.2 Hz, 1H, NC***H***$_2$), 4.15 (br, 1H, C***H***(CH$_3$)$_2$), 3.39 (br, 1H, C***H***(CH$_3$)$_2$), 3.24 (dd, *J* = 18.4, 3.2 Hz, 1H, PC***H***$_2$), 2.30 (d, *J* = 18.4 Hz, 1H, PC***H***$_2$), 1.50 (br, 3H, CH(C***H***$_3$)$_2$), 1.28 (br, 6H, CH(C***H***$_3$)$_2$), 1.14 (d, *J* = 11.1 Hz, 9H, PC(C***H***$_3$)$_3$), 0.72 (d, *J* = 11.4 Hz, 9H, PC(C***H***$_3$)$_3$), 0.54 (br, 3H, CH(C***H***$_3$)$_2$). ^{13}C NMR (101 MHz, C_6D_6) δ 167.45 (d, *J* = 1.8 Hz, Py***C***), 159.18 (s, Py***C***), 158.89 (s, Ar***C***), 154.03 (s, Ar***C***), 137.12 (d, *J* = 2.8 Hz, Py***C***), 129.01 (s, Ar***C***), 122.86 (d, *J* = 28.2 Hz, Py***C***), 122.04 (s, Ar***C***), 120.84 (s, Py***C***), 62.77 (s, N***C***H$_2$), 31.51 (d, *J* = 21.8 Hz, P***C***(CH$_3$)$_3$), 30.68 (d, *J* = 20.0 Hz, P***C***(CH$_3$)$_3$), 29.84 (d, *J* = 13.1 Hz, PC(***C***H$_3$)$_3$), 29.66 (d, *J* = 13.9 Hz, PC(***C***H$_3$)$_3$), 28.88 (d, *J* = 26.6 Hz, P***C***H$_2$), 27.84 (s, ***C***H(CH$_3$)$_2$), 25.89 (s, CH(***C***H$_3$)$_2$), 24.11 (s, CH(***C***H$_3$)$_2$). ^{31}P NMR (162 MHz, C_6D_6) δ 20.81 (s).

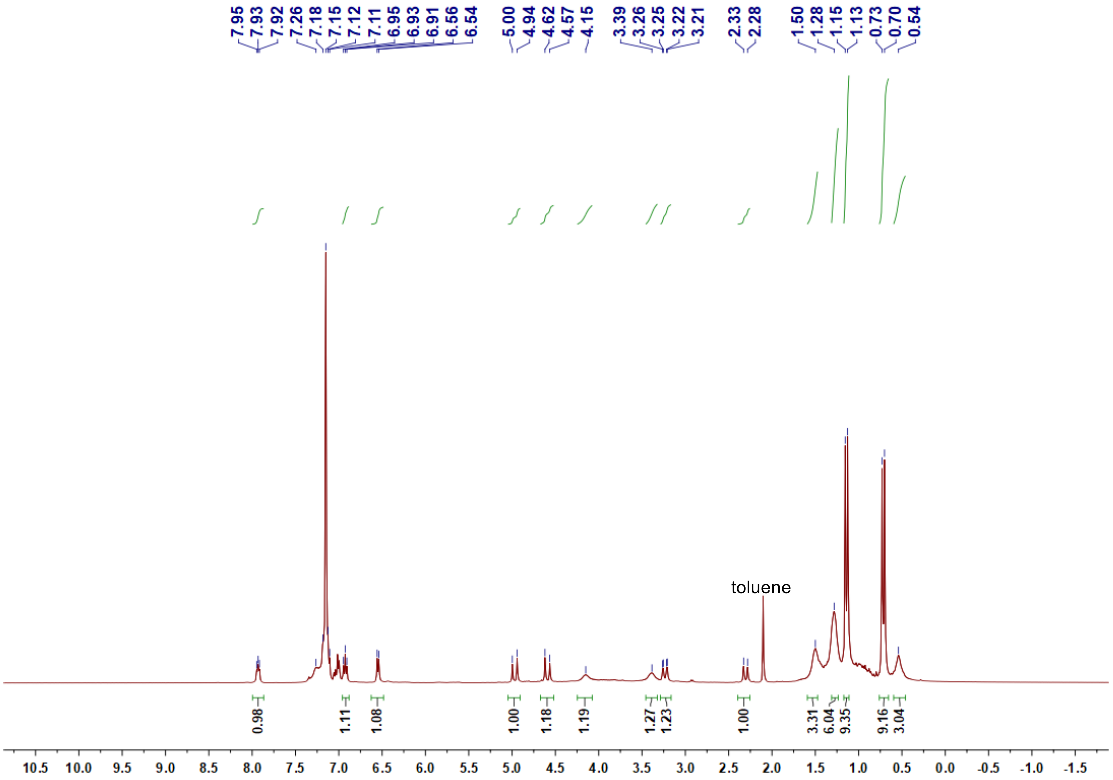

Figure S30. ^{1}H NMR (400 MHz, C_6D_6) spectrum of **Mg-3**

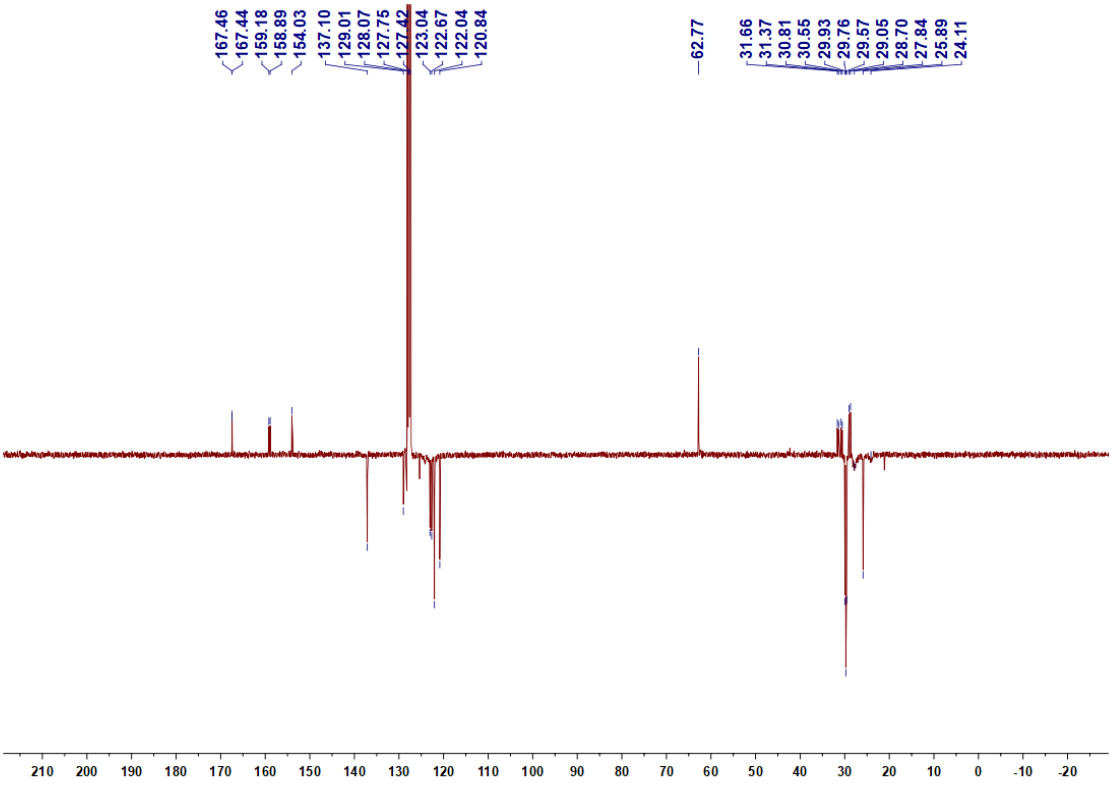

Figure S31. ^{13}C-DEPTQ NMR (101 MHz, C_6D_6) spectrum of **Mg-3**

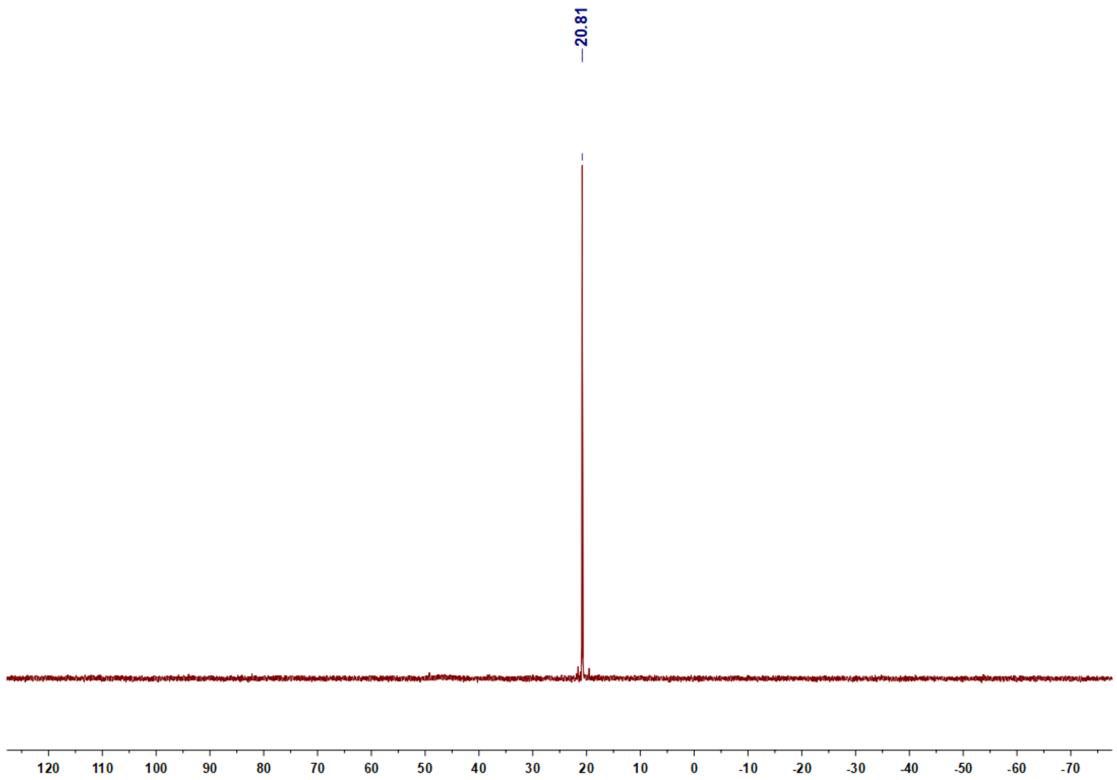

Figure S32. ^{31}P NMR (162 MHz, C_6D_6) spectrum of **Mg-3**

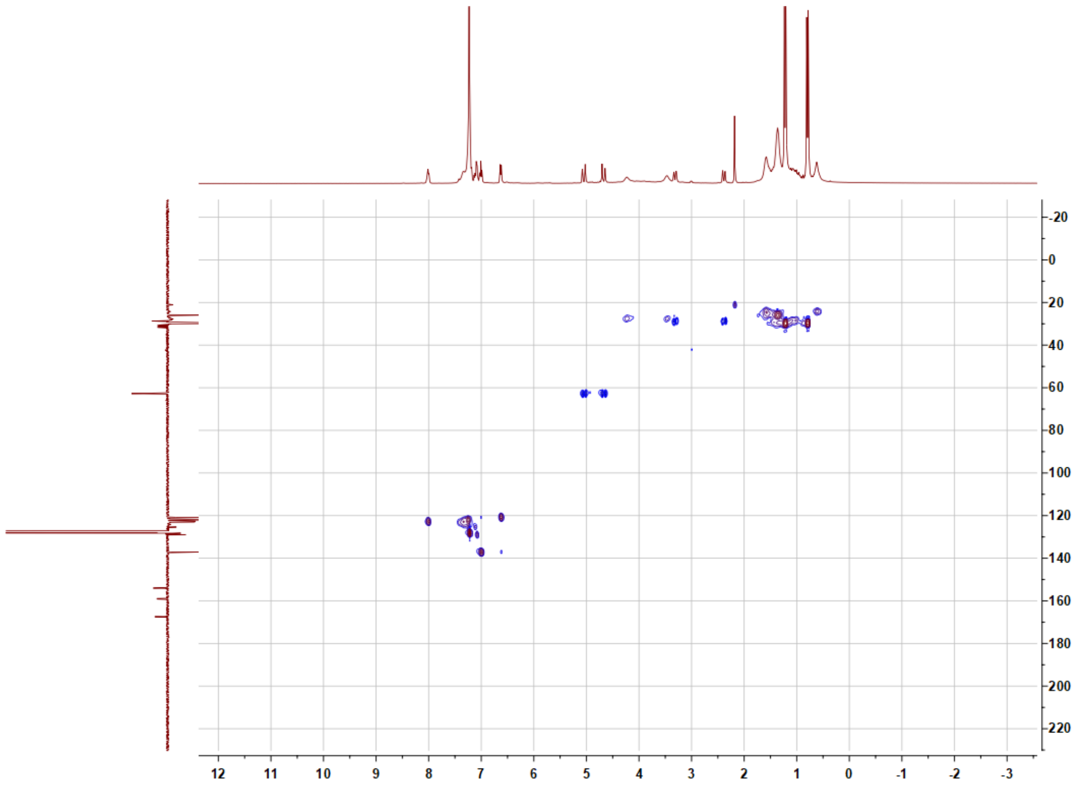

Figure S33. HSQC spectrum of **Mg-3** in C_6D_6

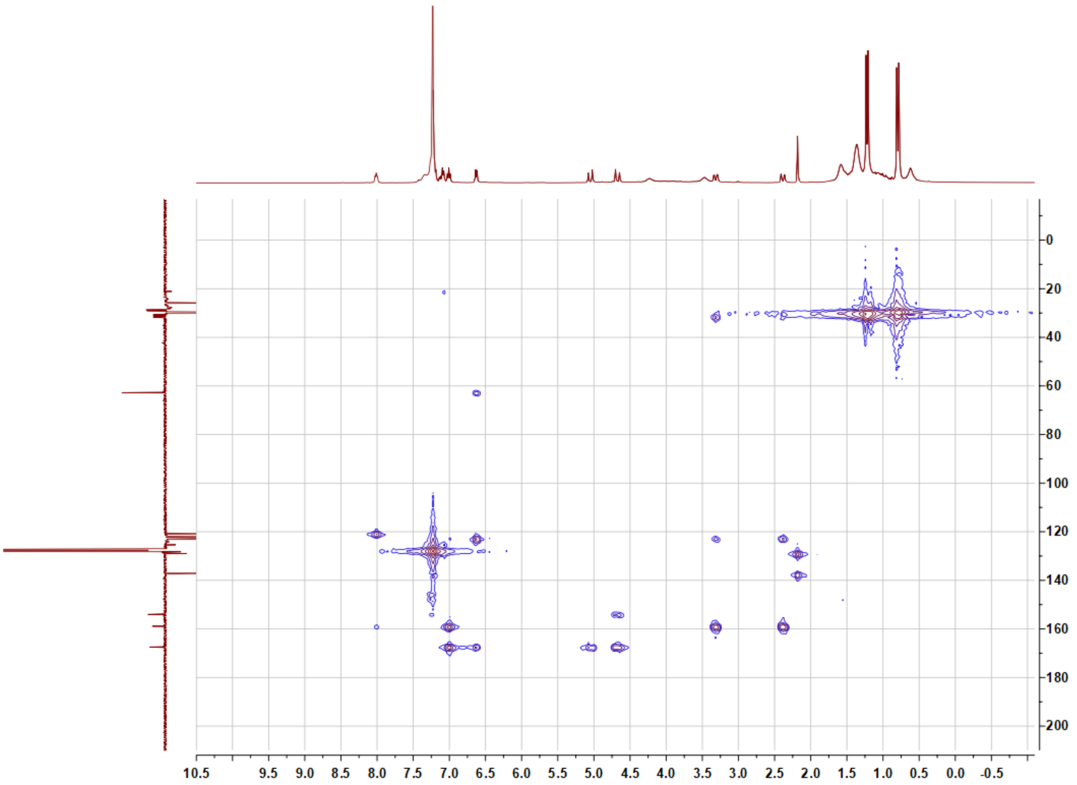

Figure S34. HMBC spectrum of **Mg-3** in C_6D_6

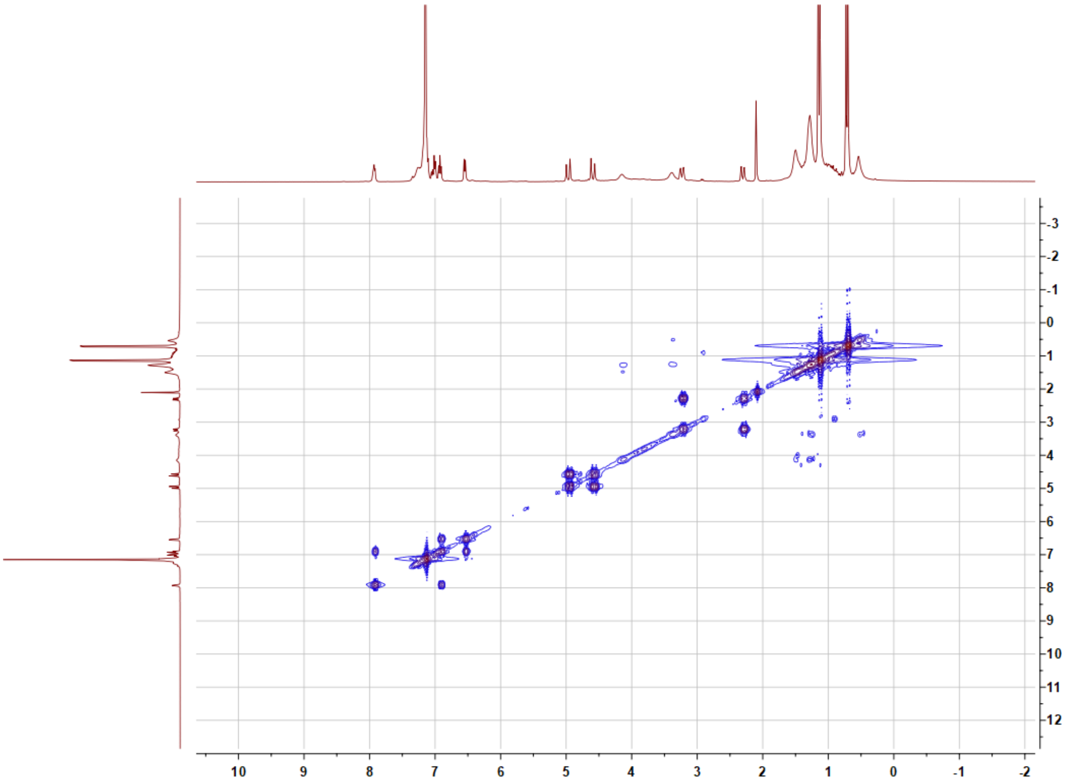

Figure S35. H-H COSY spectrum of **Mg-3** in C_6D_6

The diffraction data from single crystals of **Mg-3** were collected on Rigaku Synergy-R diffractometer dual source equipped with a HyPix ARC 150º detector with CuKα (λ = 1.54184 Å). The data were processed with CrysAlis^{PRO} [2]. The structures were solved with SHELXT[3]. Full matrix least-squares and refined based on F^2 with SHELXL[4]. All structure solution and refinement programs are implemented in Olex-2 GUI[5]. All non-hydrogen atoms were refined with anisotropic displacement coefficients. Hydrogens were placed in calculated positions and refined in riding mode. Supplementary crystallographic data have been deposited at the Cambridge Crystallographic Data Center (CCDC 2237496).

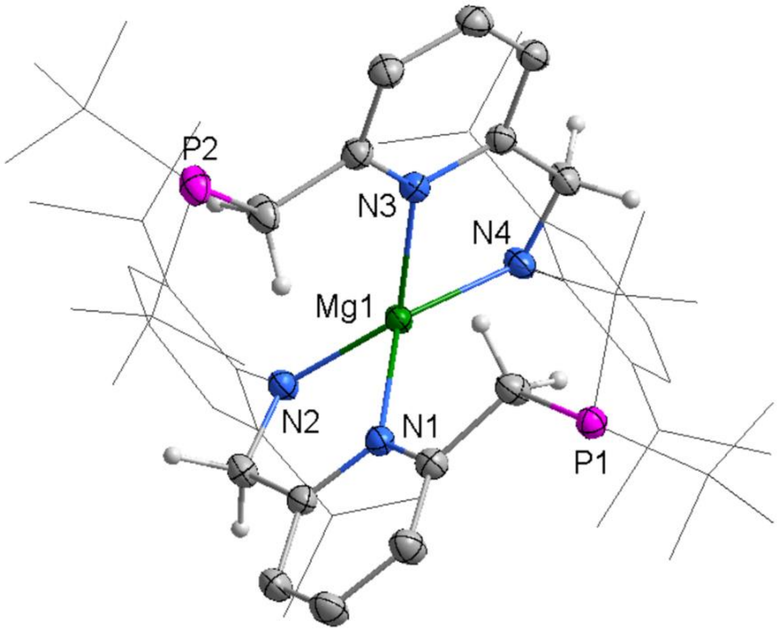

Figure S36. X-ray crystal structure of **Mg-3**

Table S10. Crystal data and structure refinement for Mg-3

Empirical formula	$C_{56}H_{87}MgN_4OP_2$
Formula weight	918.54
Temperature/K	100.01(10)
Crystal system	monoclinic
Space group	$P2_1/n$
a/Å	10.0407(2)
b/Å	26.3592(5)
c/Å	21.0954(4)

α/°	90
β/°	101.365(2)
γ/°	90
Volume/Å^3	5473.73(19)
Z	4
ρ_{calc}g/cm^3	1.115
μ/mm^{-1}	1.130
F(000)	2004.0
Crystal size/mm^3	0.257 × 0.229 × 0.183
Radiation	CuKα (λ = 1.54184)
2Θ range for data collection/°	6.706 to 150.106
Index ranges	-12 ≤ h ≤ 10, -32 ≤ k ≤ 31, -26 ≤ l ≤ 26
Reflections collected	87386
Independent reflections	11105 [R_{int} = 0.0333, R_{sigma} = 0.0225]
Data/restraints/parameters	11105/0/606
Goodness-of-fit on F^2	1.085
Final R indexes [I>=2σ (I)]	R_1 = 0.0374, wR_2 = 0.1013
Final R indexes [all data]	R_1 = 0.0427, wR_2 = 0.1052
Largest diff. peak/hole / e Å^{-3}	0.29/-0.35

Table S11. Bond lengths for Mg-3

Atom	Atom	Length/Å	Atom	Atom	Length/Å
P1	C12	1.8812(14)	C16	C21	1.4182(18)
P1	C8	1.8842(13)	C39	C42	1.538(2)
P1	C1	1.8659(13)	C39	C41	1.533(2)
P2	C28	1.8662(13)	C39	C40	1.533(2)
P2	C39	1.8875(15)	C32	C31	1.3751(18)
P2	C35	1.8872(16)	C10	C8	1.5407(19)
Mg1	N2	1.9673(11)	C2	C3	1.3807(18)
Mg1	N1	2.1616(11)	C2	C1	1.5149(17)
Mg1	N3	2.1633(11)	C6	C5	1.3914(18)
Mg1	N4	1.9680(11)	C44	C43	1.4139(18)
N2	C7	1.4394(16)	C44	C45	1.3942(18)
N2	C16	1.4136(16)	C43	C48	1.4182(18)

N1	C2	1.3612(16)	C25	C21	1.5196(18)
N1	C6	1.3494(15)	C25	C27	1.5345(19)
N3	C33	1.3460(16)	C29	C30	1.3822(18)
N3	C29	1.3639(16)	C22	C17	1.5147(18)
N4	C43	1.4166(16)	C22	C23	1.5276(18)
N4	C34	1.4371(15)	C17	C18	1.3956(18)
C13	C12	1.535(2)	C12	C15	1.529(2)
C26	C25	1.5262(19)	C12	C14	1.543(2)
C49	C51	1.5235(19)	C18	C19	1.3855(19)
C49	C44	1.5193(18)	C8	C11	1.5280(19)
C49	C50	1.5302(19)	C48	C47	1.3954(18)
C24	C22	1.5307(19)	C21	C20	1.3919(18)
C9	C8	1.5343(18)	C35	C37	1.527(2)
C4	C3	1.3922(18)	C35	C36	1.539(2)
C4	C5	1.3741(19)	C31	C30	1.3877(19)
C38	C35	1.537(2)	C20	C19	1.386(2)
C33	C32	1.3934(17)	C47	C46	1.379(2)
C33	C34	1.5070(17)	C46	C45	1.388(2)
C28	C29	1.5117(17)	C55	O1	1.373(2)
C7	C6	1.5042(17)	C55	C56[1]	1.493(3)
C52	C54	1.5314(19)	C55	O1A	1.274(5)
C52	C48	1.5190(19)	C55	C56A[1]	1.601(8)
C52	C53	1.529(2)	O1	C56	1.414(3)
C16	C17	1.4153(18)	O1A	C56A	1.391(9)

[1]1-X,1-Y,2-Z

Table S12. Bond angles for Mg-3

Atom	Atom	Atom	Angle/°	Atom	Atom	Atom	Angle/°
C12	P1	C8	111.09(6)	C44	C43	C48	118.84(11)
C1	P1	C12	102.02(6)	C26	C25	C27	110.52(12)
C1	P1	C8	100.47(6)	C21	C25	C26	110.57(12)
C28	P2	C39	99.53(6)	C21	C25	C27	112.27(11)
C28	P2	C35	103.13(7)	N3	C29	C28	115.17(11)
C35	P2	C39	109.64(7)	N3	C29	C30	121.07(11)

N2	Mg1	N1	82.03(4)	C30	C29	C28	123.75(11)
N2	Mg1	N3	133.73(5)	C17	C22	C24	110.22(11)
N2	Mg1	N4	128.05(5)	C17	C22	C23	112.66(11)
N1	Mg1	N3	104.11(4)	C23	C22	C24	110.80(11)
N4	Mg1	N1	131.46(5)	C16	C17	C22	120.24(11)
N4	Mg1	N3	82.06(4)	C18	C17	C16	119.94(12)
C7	N2	Mg1	116.01(8)	C18	C17	C22	119.80(12)
C16	N2	Mg1	131.52(8)	C13	C12	P1	108.45(10)
C16	N2	C7	112.28(10)	C13	C12	C14	107.84(13)
C2	N1	Mg1	130.61(8)	C15	C12	P1	117.54(10)
C6	N1	Mg1	110.38(8)	C15	C12	C13	109.29(12)
C6	N1	C2	119.00(11)	C15	C12	C14	108.69(12)
C33	N3	Mg1	110.02(8)	C14	C12	P1	104.59(9)
C33	N3	C29	119.15(10)	C19	C18	C17	121.18(12)
C29	N3	Mg1	130.61(8)	C2	C3	C4	119.53(12)
C43	N4	Mg1	131.54(8)	C9	C8	P1	108.98(9)
C43	N4	C34	112.25(10)	C9	C8	C10	108.03(11)
C34	N4	Mg1	115.58(8)	C10	C8	P1	104.54(9)
C51	C49	C50	110.32(12)	C11	C8	P1	117.05(9)
C44	C49	C51	110.35(11)	C11	C8	C9	108.90(11)
C44	C49	C50	113.33(11)	C11	C8	C10	108.96(12)
C5	C4	C3	119.09(12)	C43	C48	C52	121.73(11)
N3	C33	C32	121.81(11)	C47	C48	C52	118.90(12)
N3	C33	C34	118.48(11)	C47	C48	C43	119.34(12)
C32	C33	C34	119.71(11)	C16	C21	C25	121.45(11)
C29	C28	P2	116.53(9)	C20	C21	C16	119.63(12)
N2	C7	C6	113.06(10)	C20	C21	C25	118.83(12)
C48	C52	C54	111.58(11)	C38	C35	P2	106.70(10)
C48	C52	C53	111.70(12)	C38	C35	C36	106.93(14)
C53	C52	C54	109.84(12)	C37	C35	P2	116.46(11)
N2	C16	C17	120.19(11)	C37	C35	C38	109.44(15)
N2	C16	C21	121.28(11)	C37	C35	C36	109.26(13)
C17	C16	C21	118.49(12)	C36	C35	P2	107.63(12)

C42	C39	P2	104.10(10)	C32	C31	C30	119.44(12)
C41	C39	P2	117.93(11)	C19	C20	C21	121.60(12)
C41	C39	C42	108.78(14)	C29	C30	C31	119.52(12)
C41	C39	C40	108.95(12)	C2	C1	P1	116.35(9)
C40	C39	P2	109.01(11)	C46	C47	C48	121.62(13)
C40	C39	C42	107.58(14)	N4	C34	C33	113.06(10)
C31	C32	C33	119.01(12)	C18	C19	C20	119.08(12)
N1	C2	C3	121.30(11)	C47	C46	C45	119.27(12)
N1	C2	C1	115.22(11)	C46	C45	C44	121.18(12)
C3	C2	C1	123.42(11)	C4	C5	C6	119.35(12)
N1	C6	C7	118.31(11)	O1	C55	C56[1]	114.1(2)
N1	C6	C5	121.71(12)	O1	C55	C56A[1]	97.9(3)
C5	C6	C7	119.98(11)	C56[1]	C55	C56A[1]	24.9(2)
C43	C44	C49	120.35(11)	O1A	C55	C56A[1]	117.7(4)
C45	C44	C49	119.90(12)	C55	O1	C56	112.07(19)
C45	C44	C43	119.71(12)	O1	C56	C55[1]	113.5(2)
N4	C43	C48	120.67(11)	C55	O1A	C56A	117.0(5)
C44	C43	N4	120.49(11)				

[1]1-X,1-Y,2-Z

3.2 Reaction of Mg-2a and Mg-2b with D_2

To confirm that **Mg-2a** and **Mg-2b** can reversibly activate H_2, a toluene solution (0.6 mL) of **Mg-2a** (6.1 mg, 0.01 mmol) was pressurized with 5 bar of D_2 and heated at 120 °C in a J. Young NMR tube. After 72 h, **Mg-2a** was consumed, and **Mg-3-D** was obtained with deuterium signals on the phosphine side arm of **Mg-3**, which was confirmed by the ^{2}H NMR. The integration of the ^{1}H NMR confirmed that 91% of deuterium was incorporated into the phosphine side arm.

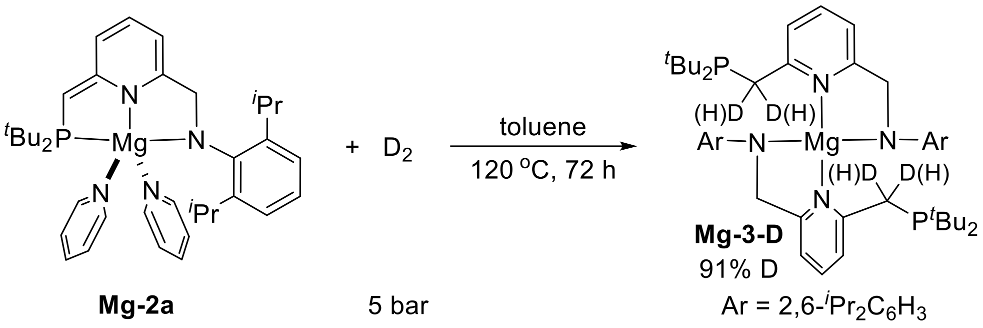

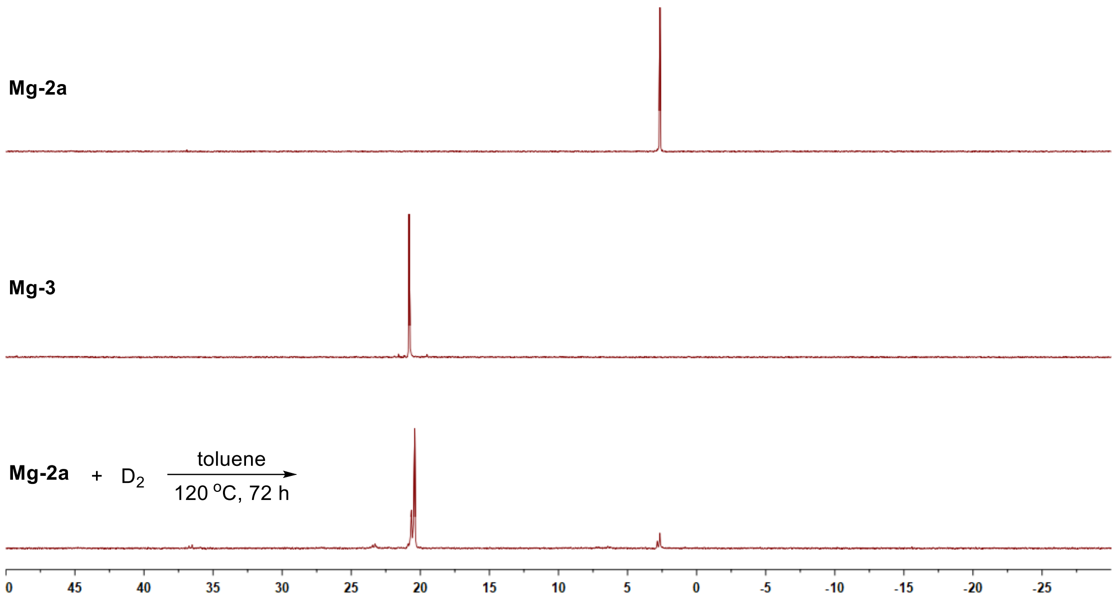

Figure S37. ^{31}P NMR spectra of **Mg-2a**, **Mg-3**, and the reaction of **Mg-2a** with D_2

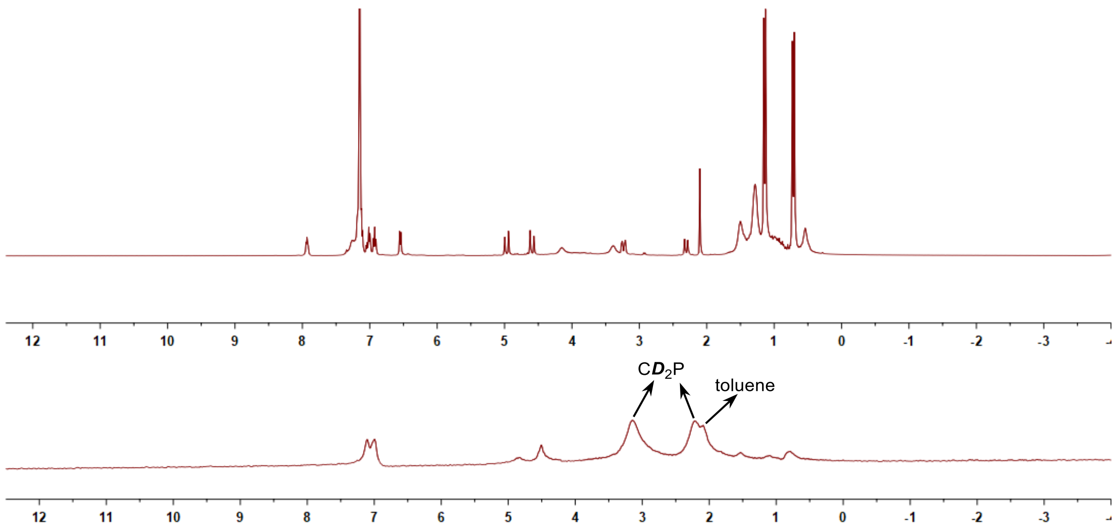

Figure S38. Top: ^{1}H NMR spectrum of **Mg-3**. Bottom: ^{2}H NMR spectrum of the reaction of **Mg-2a** with D_2 (after removing the volatiles and redissolving in toluene)

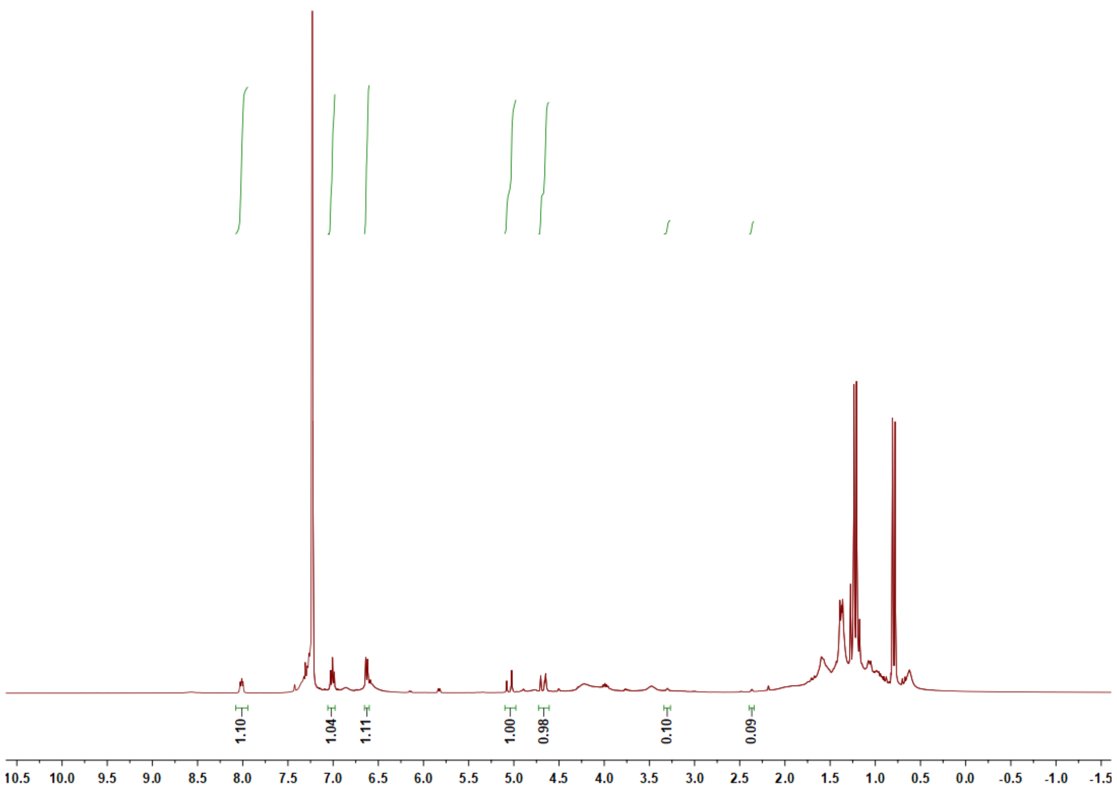

Figure S39. ^{1}H NMR spectrum of **Mg-3-D** in C_6D_6

Likewise, the treatment of **Mg-2b** (6.2 mg, 0.01 mmol) with D_2 (5 bar) under similar conditions resulted in the full consumption of **Mg-2b**. And **Mg-3-D** was obtained with 87% of deuterated ratio of the phosphine side arm. The results certified that the dearomatized complexes **Mg-2a** and **Mg-2b** can reversibly activate D_2 (H_2).

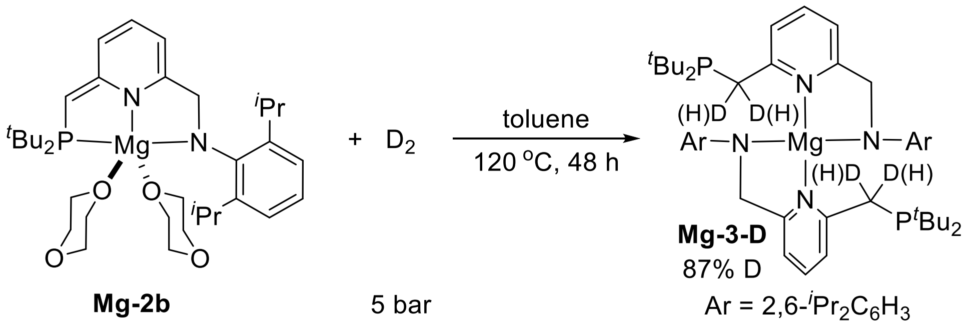

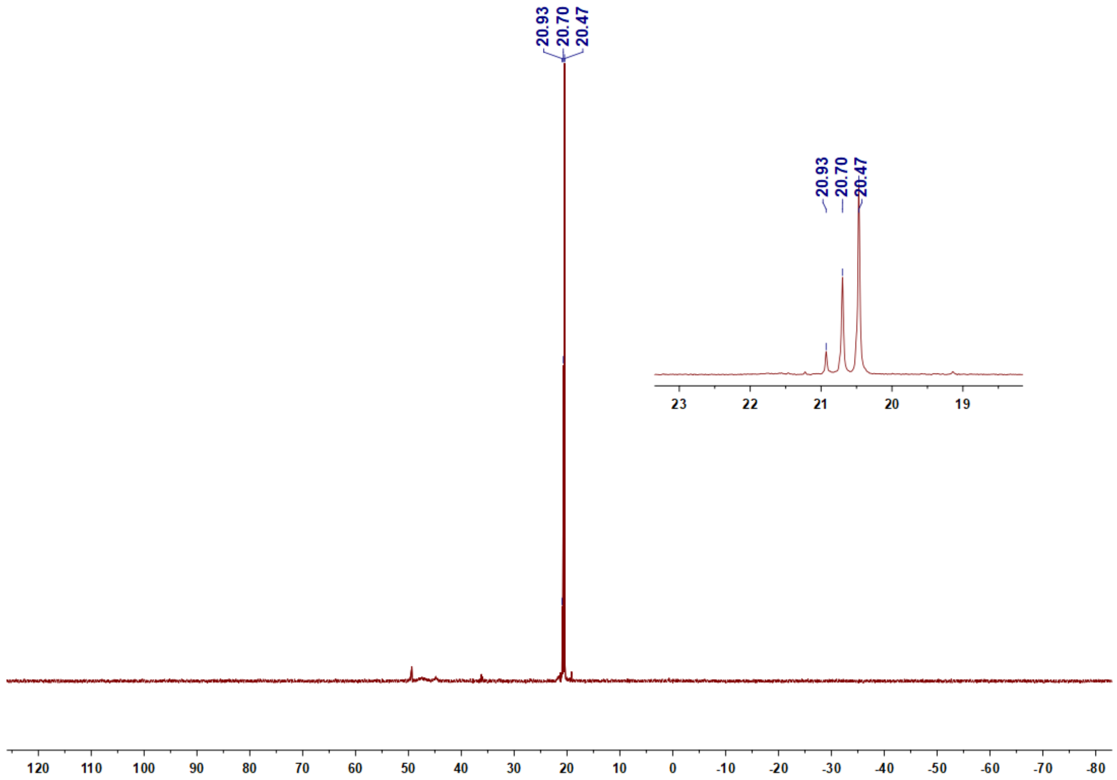

Figure S40. ^{31}P NMR spectrum of the reaction of **Mg-2b** with D_2

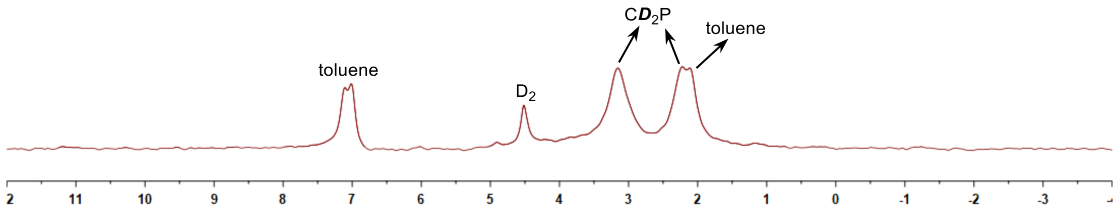

Figure S41. ^{2}H NMR spectrum of the reaction of **Mg-2b** with D_2

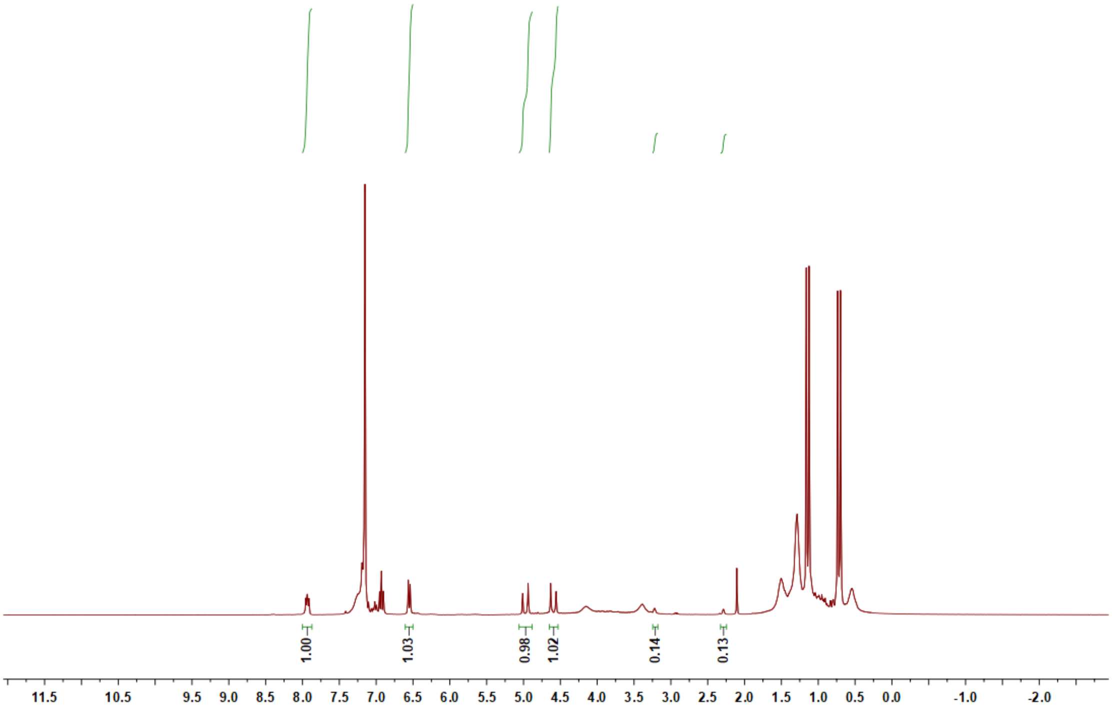

Figure S42. ^{1}H NMR spectrum of the reaction of **Mg-2b** with D_2 (after removing volatiles and redissolving in C_6D_6)

In addition, upon treatment a toluene solution (0.6 mL) of **Mg-2a** (6.1 mg, 0.01 mmol) with 5 bar of D_2 at 60 °C, **Mg-2a-D** was formed with 80% deuterium incorporated into the phosphine side arm of **Mg-2a** after 48 h, further confirming the reversible activation of D_2 (H_2) by the dearomatized complexes.

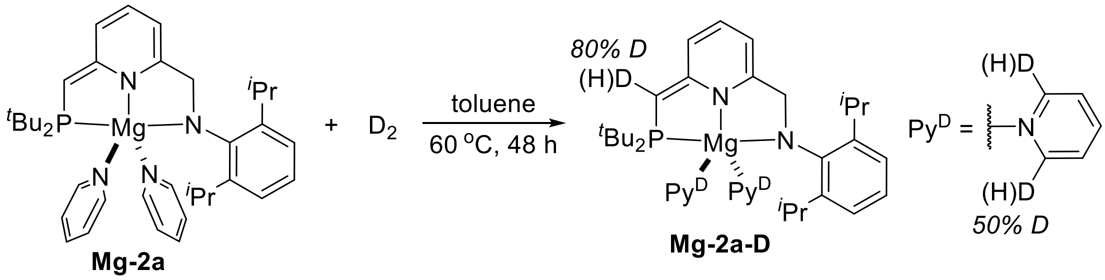

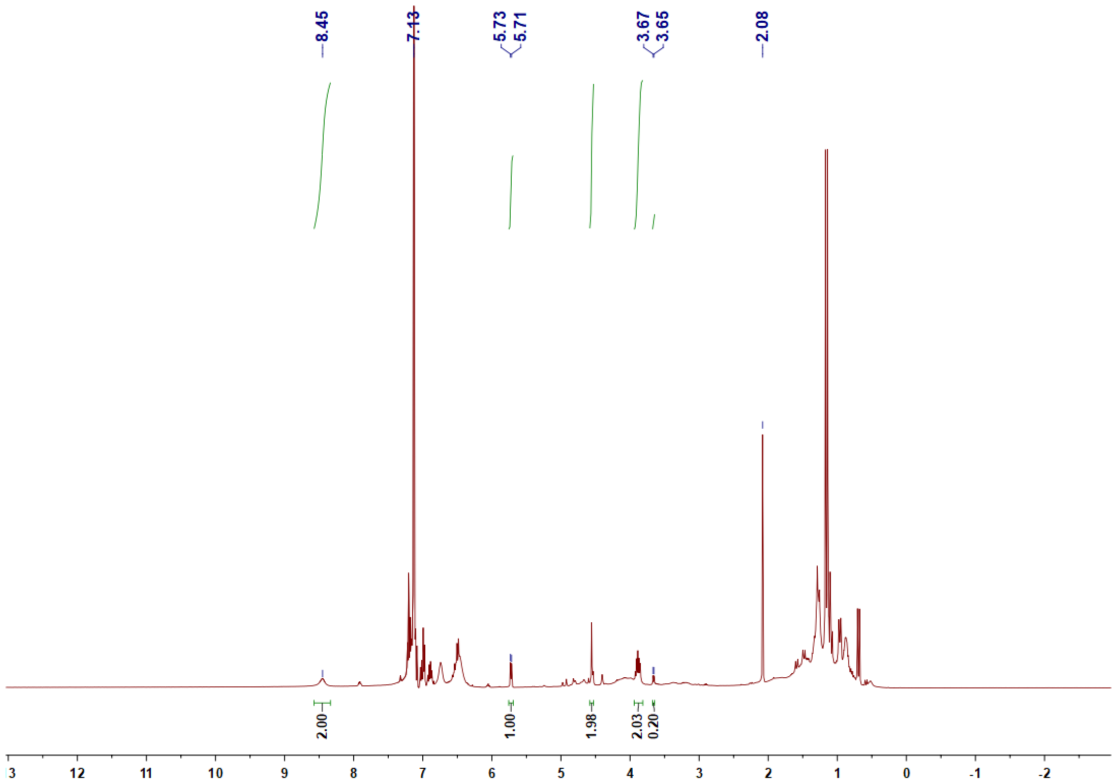

Figure S43. ^{1}H NMR spectrum of the reaction of **Mg-2a** with D_2 at 60 °C (after removing volatiles and redissolving in C_6D_6)

3.3 Reaction of Mg-2b with free ligand L1

A toluene solution (0.6 mL) of **Mg-2b** (6.2 mg, 0.01 mmol) and **L1** (4.3 mg, 0.01 mmol) in a J. Young NMR tube was heated in the presence of H_2 (5 bar) at 75 °C. **Mg-2b** was fully converted into **Mg-3** after 4 h. On the contrary, only a small amount (~7%) of **Mg-3** can be observed in the absence of H_2 under similar conditions. These results suggest that H_2 is required for the formation of **Mg-3**.

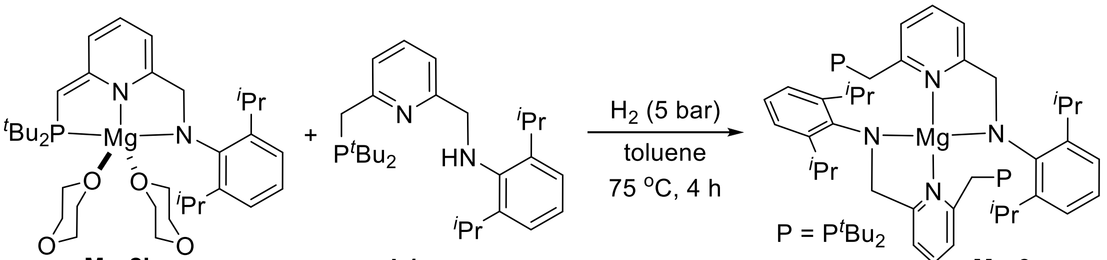

Mg-2b **L1** **Mg-3**

Full conversion

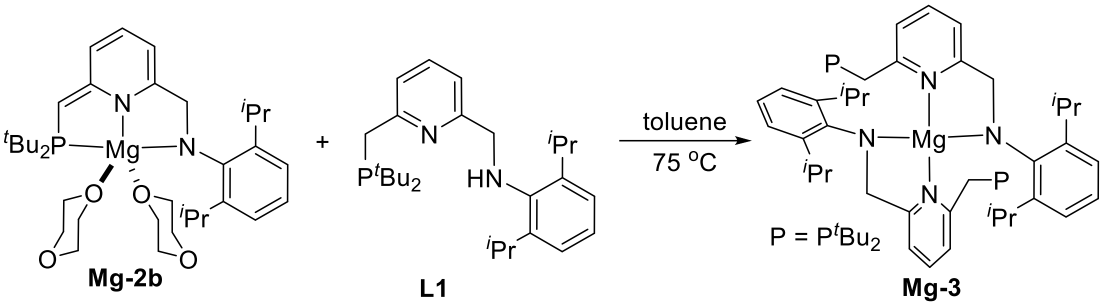

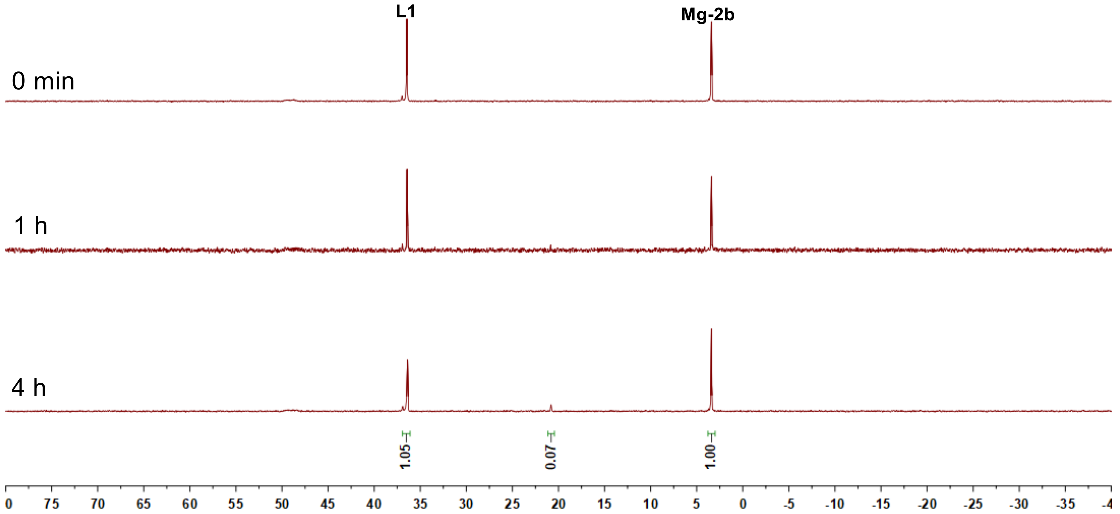

Figure S44. ^{31}P NMR spectrum of the reaction of **Mg-2b** with **L1** in the absence of H_2

3.4 Conversion of Mg-3 into the dearomatized complexes and free ligand L1

The dimer complex **Mg-3** is unstable in the absence of H_2. Upon treatment of **Mg-3** (8.7 mg, 0.01 mmol) with pyridine (4.0 mg, 0.05 mmol) or dioxane (4.4 mg, 0.05 mmol) at 120 °C, the dearomatized complexes **Mg-2a** and **Mg-2b** were gradually regenerated together with the formation of free ligand **L1**.

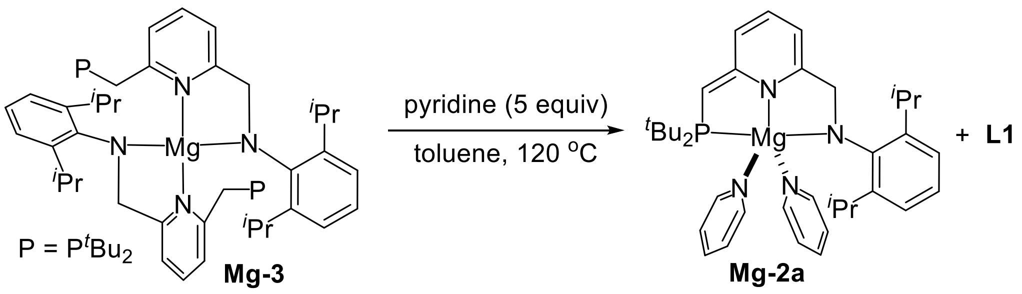

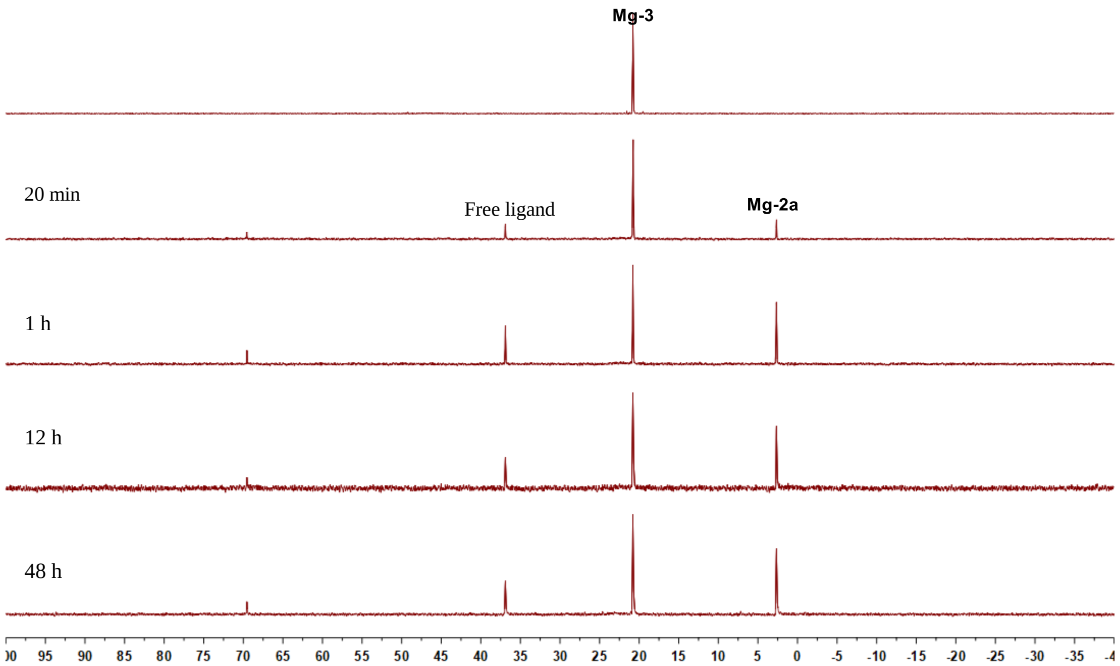

Figure S45. ^{31}P NMR spectrum of converting **Mg-3** to **Mg-2a** and free ligand **L1**

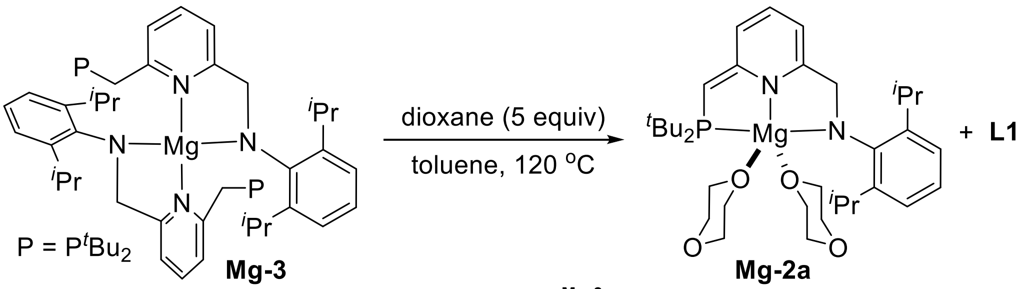

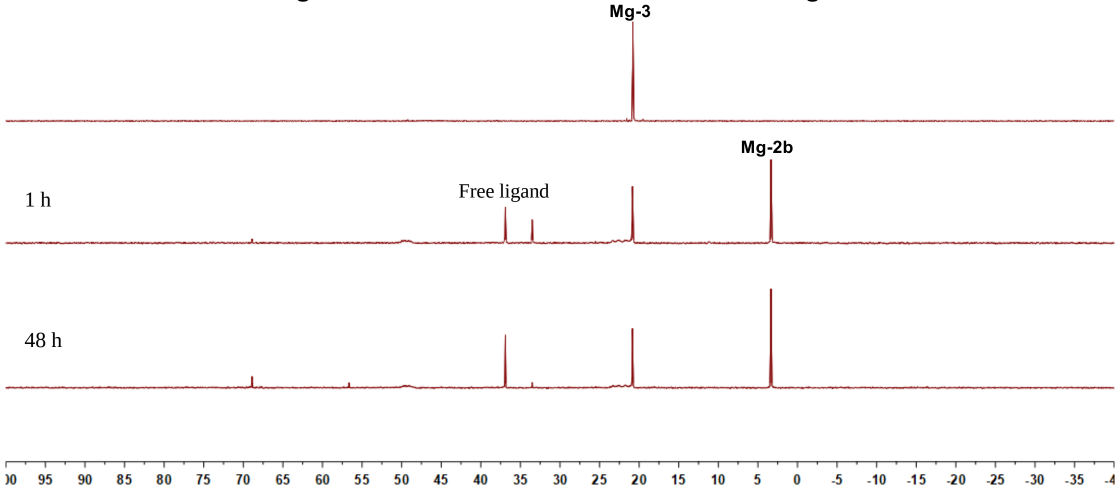

Figure S46. ^{31}P NMR spectrum of converting **Mg-3** to **Mg-2b** and free ligand **L1**

To confirm that the formation of **Mg-3** is reversible, a toluene solution (0.6 mL) of **Mg-3** (8.7 mg, 0.01 mmol) was treated with D_2 (5 bar) in a J. Young NMR tube and

heated at 120 °C. 68% of deuterium was incorporated into the phosphine side arm after 48 h, and the deuterated ratio increased to 87% after 5 days. The result confirmed that the formation of **Mg-3** is reversible.

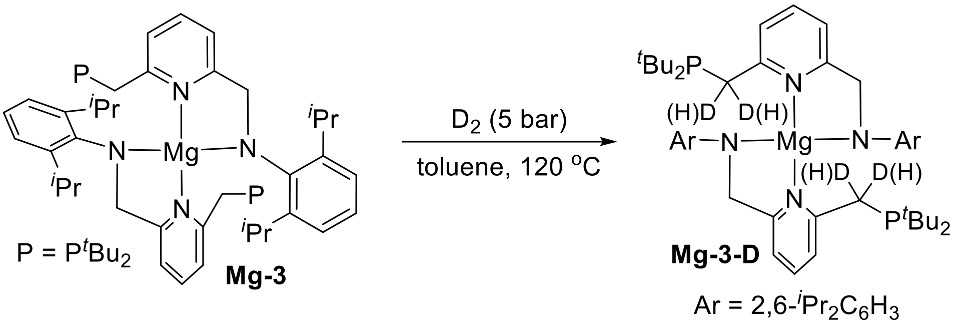

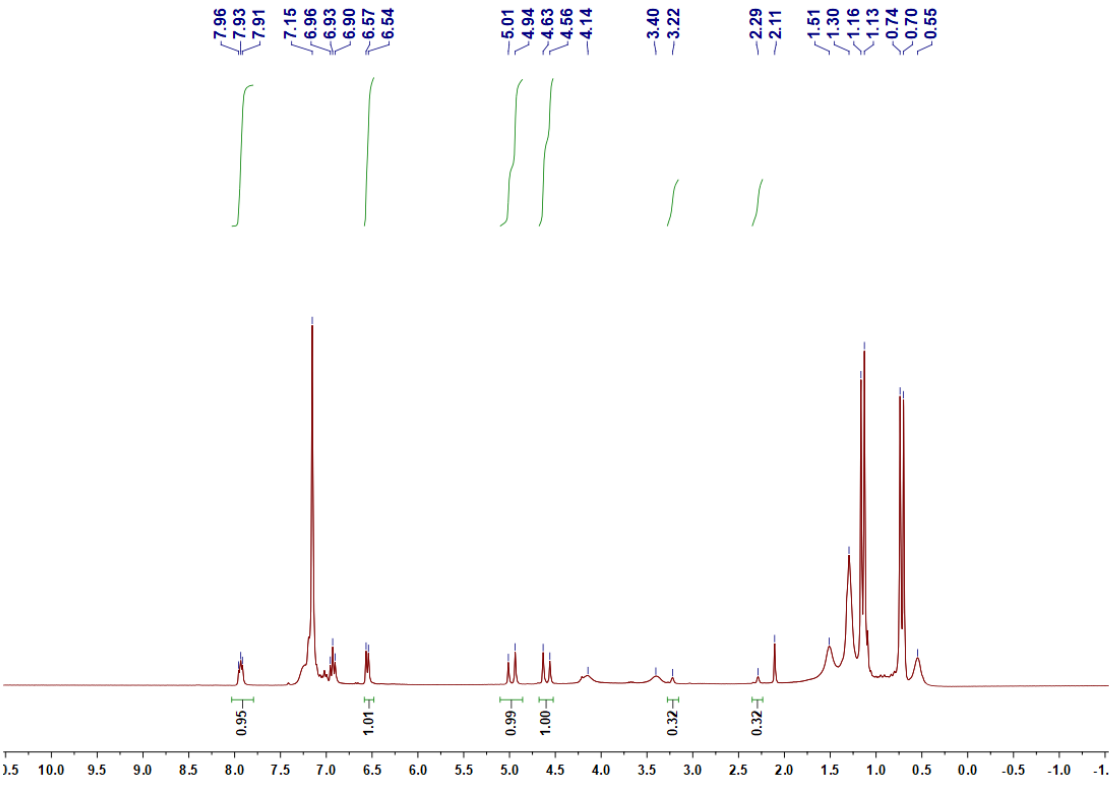

Figure S47. ^{1}H NMR (300 MHz, C_6D_6) spectrum of the reaction of **Mg-3** with D_2 (48 h, 68% D)

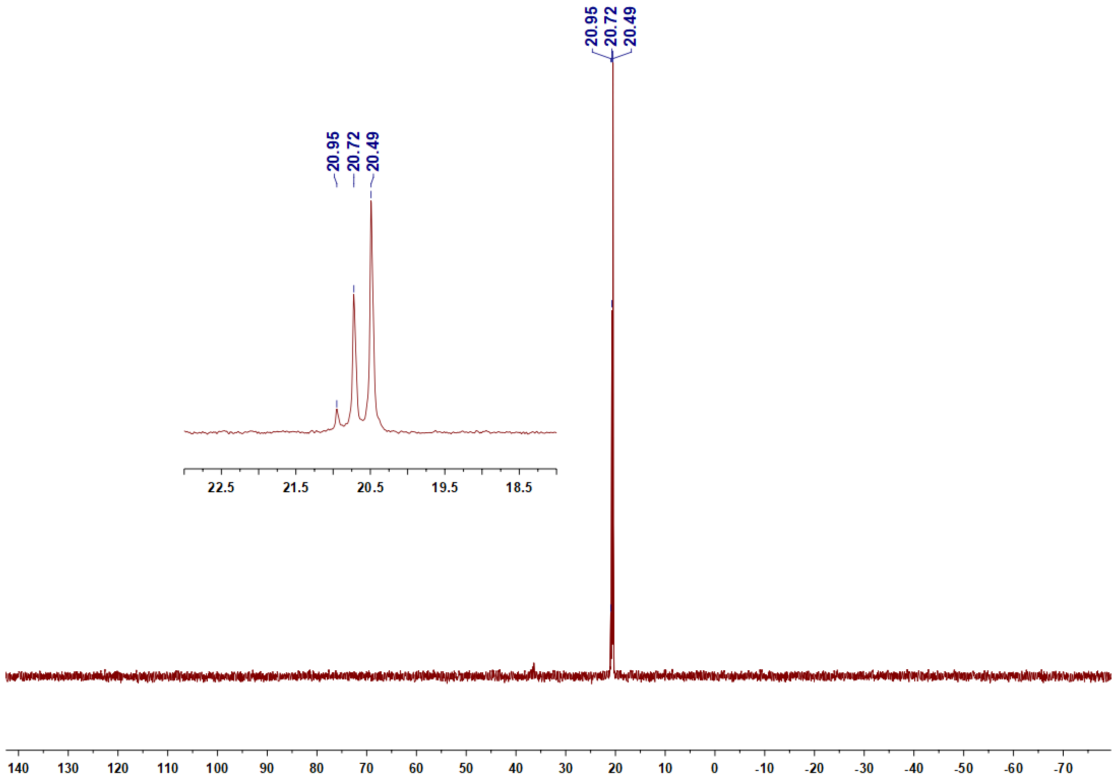

Figure S48. ^{31}H NMR (121 MHz, C_6D_6) spectrum of the reaction of **Mg-3** with D_2 after 48 h

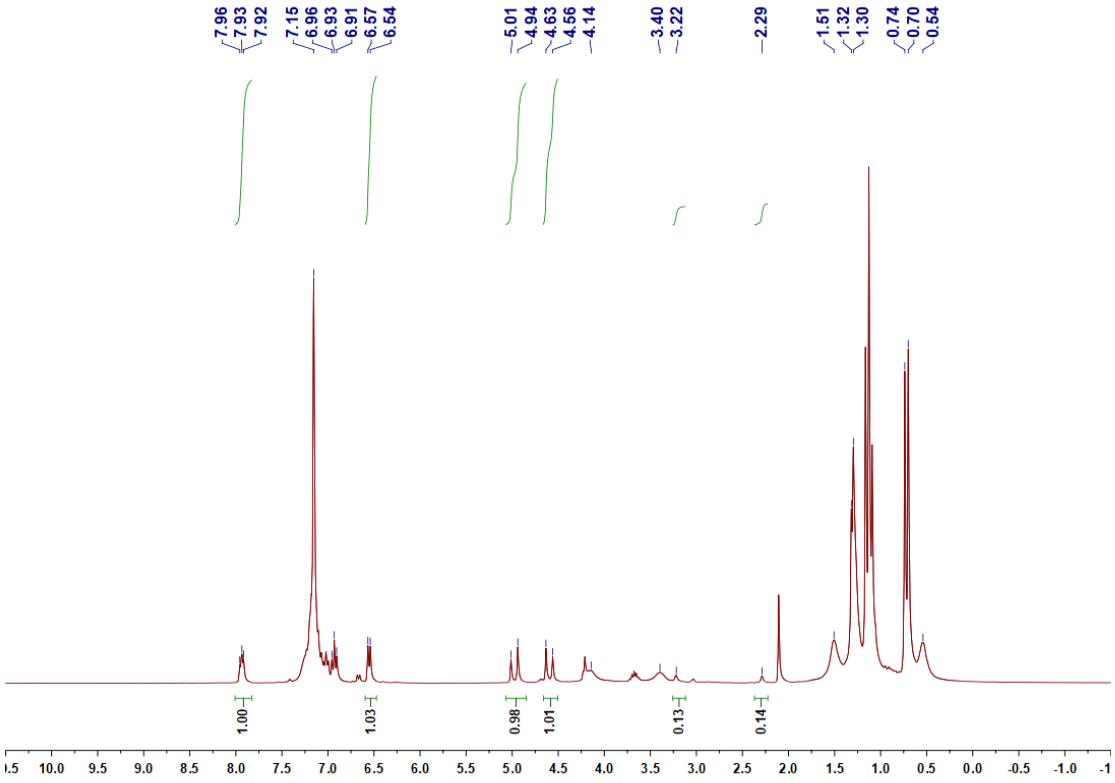

Figure S49. ^{1}H NMR (300 MHz, C_6D_6) spectrum of the reaction of **Mg-3** with D_2 (5 d, 87% D)

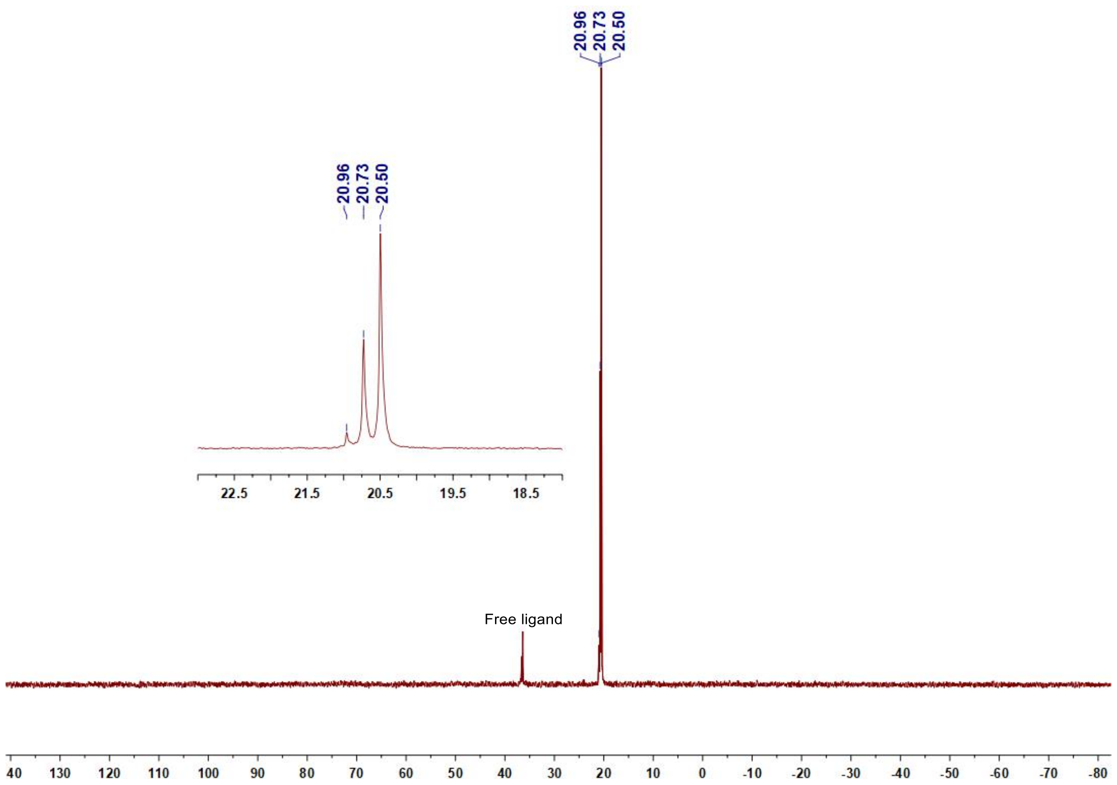

Figure S50. ^{31}H NMR (121 MHz, C_6D_6) spectrum of the reaction of **Mg-3** with D_2 after 5 days

3.5 Reaction of Mg-2a with *N*-benzylaniline

Upon treatment of the benzene solution (0.6 mL) of **Mg-2a** (12.1 mg, 0.02 mmol) with *N*-benzylaniline (3.7 mg, 0.02 mmol) in a J. Young NMR tube, **Mg-2a** was fully consumed after for 18 h at room temperature. **Mg-4** was formed as the major product. However, only free ligand could be observed in the reaction of previously reported Mg(PNP) complex (9.8 mg, 0.02 mmol) with *N*-benzylaniline (3.7 mg, 0.02 mmol), suggesting that **Mg-2a** is a more stable structure compared with Mg(PNP).

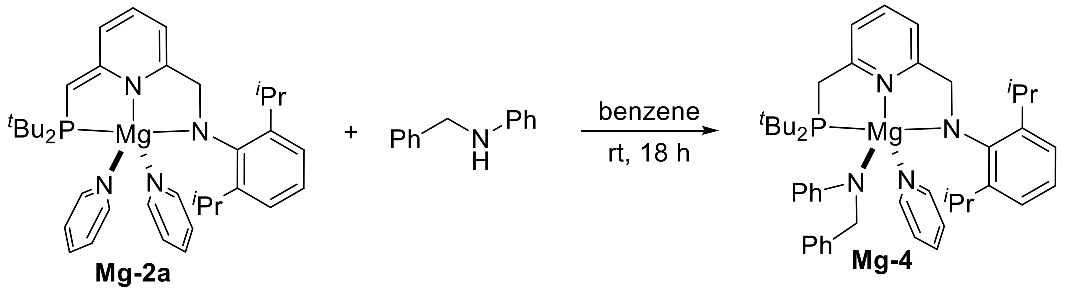

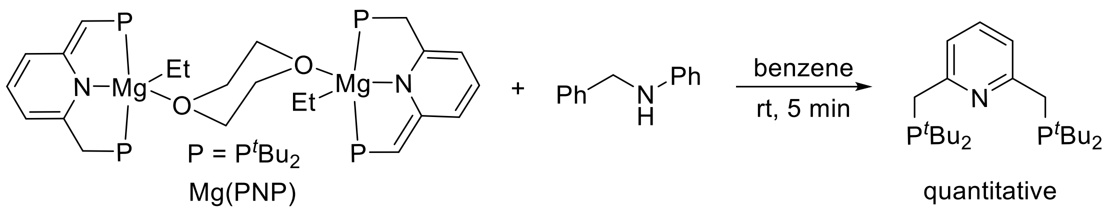

Characterization of ***Mg-4****:*

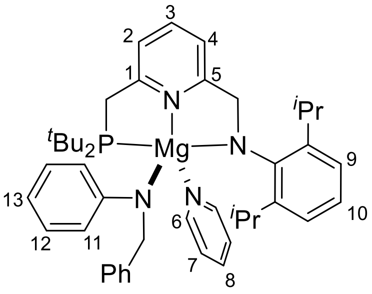

^{1}H NMR (400 MHz, C_6D_6) δ 8.42 (s, 2H, Py***H***^{6}), 7.46 (d, *J* = 7.3 Hz, 3H, Py***H***^{4} and NAr***H***^{9}), 7.32 (d, *J* = 6.9 Hz, 2H, Ph***H***), 7.28 – 7.23 (m, 2H, Ph***H***), 7.18 – 7.13 (m, 2H, NPh***H***^{12}), 7.09 (t, *J* = 7.3 Hz, 2H, NAr***H***^{10} and Ph***H***), 7.03 – 7.00 (m, 1H, Py***H***^{3}), 6.79 (d, *J* = 8.0 Hz, 3H, Py***H***^{8} and NPh***H***^{11}), 6.70 (d, *J* = 7.8 Hz, 1H, Py***H***^{2}), 6.57 (t, *J* = 7.1 Hz, 1H, NPh***H***^{13}), 6.52 – 6.43 (m, 2H, Py***H***^{7}), 5.06 (s, 2H, PyC***H***$_2$N), 4.75 (s, 2H, PhC***H***$_2$N), 4.12 – 4.01 (m, 2H, C***H***$(CH_3)_2$), 2.99 (d, *J* = 2.9 Hz, 2H, PyC***H***$_2$P), 1.31 (s, 12H, CH(C***H***$_3)_2$), 0.95 (d, *J* = 11.2 Hz, 18H, PC(C***H***$_3)_3$).

^{13}C NMR (101 MHz, C_6D_6) δ 166.84 (s, Py***C***^{5}), 160.03 (s, NCH_2Ph***C***), 159.16 (d, *J* = 14.3 Hz, Py***C***^{1}), 155.25 (s, NAr***C***), 149.57 (s, Py***C***^{6}), 147.65 (s, $(CH_3)_2$CHAr***C***), 145.30 (s, NPh***C***), 137.90 (s, Py***C***^{8}), 137.53 (s, Py***C***^{3}), 129.31 l(s, NPh***C***^{12}), 128.30 (s, NAr***C***^{10}), 127.52 (s, NAr***C***^{9}), 125.50 (s, Ph***C***), 124.24 (s, Py***C***^{7}), 123.55 (s, Ph***C***, 2C), 122.23 (s, Py***C***^{4}), 122.02 (s, Ph***C***), 120.33 (s, Py***C***^{2}), 114.66 (s, NPh***C***^{11}), 111.54 (s, NPh***C***^{13}), 62.85 (s, Py***C***H_2N), 53.63 (s, Ph***C***H_2N), 31.57 (d, *J* = 18.5 Hz, P***C***$(CH_3)_3$), 29.83 (s, Py***C***H_2P), 29.69 (d, *J* = 12.5 Hz, PC(***C***$H_3)_3$), 27.88 (s, ***C***$H(CH_3)_2$), 25.56 (s, CH(***C***$H_3)_2$).

^{31}P NMR (162 MHz, C_6D_6) δ 24.87 (s).

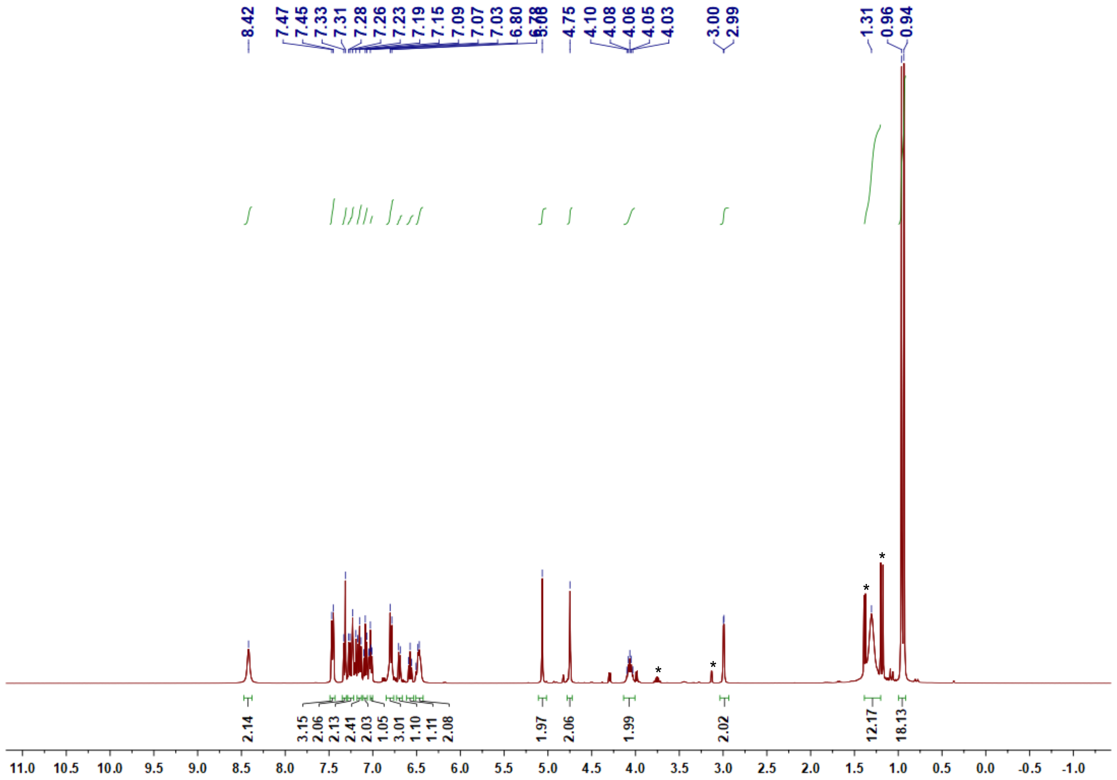

Figure S51. ^{1}H NMR (400 MHz, C_6D_6) spectrum of **Mg-4** (*free ligand)

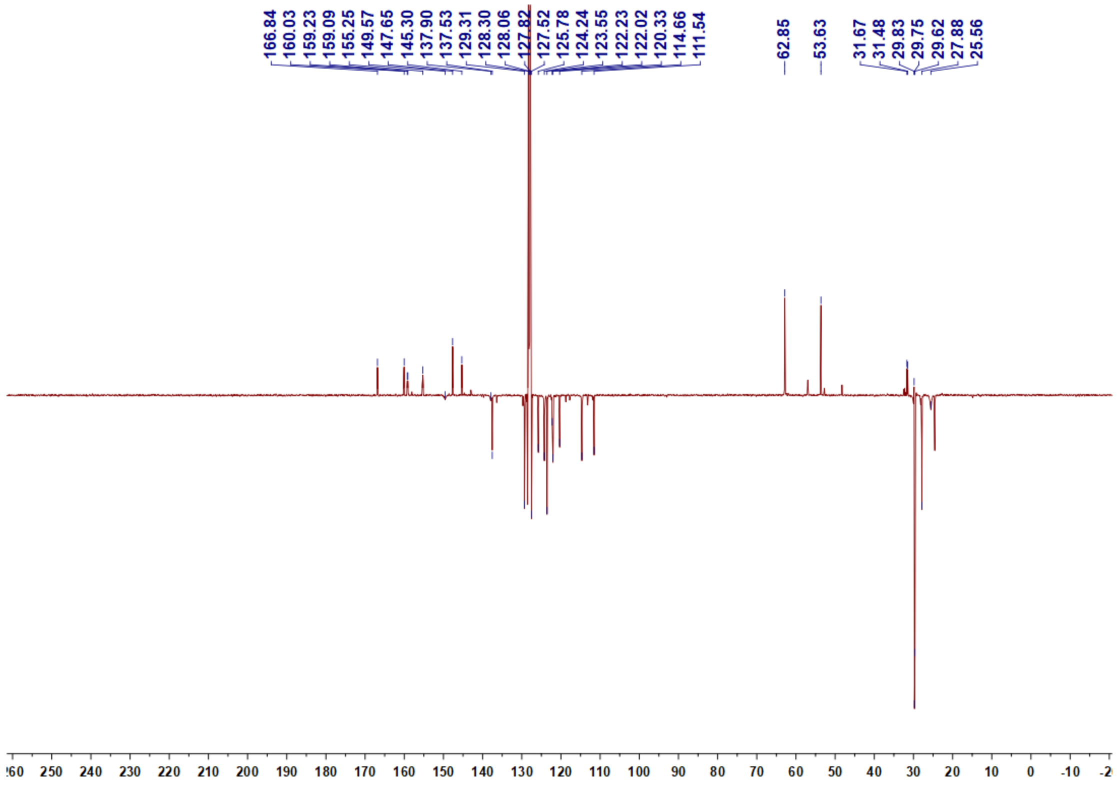

Figure S52. ^{13}C-DEPTQ NMR (101 MHz, C_6D_6) spectrum of **Mg-4**

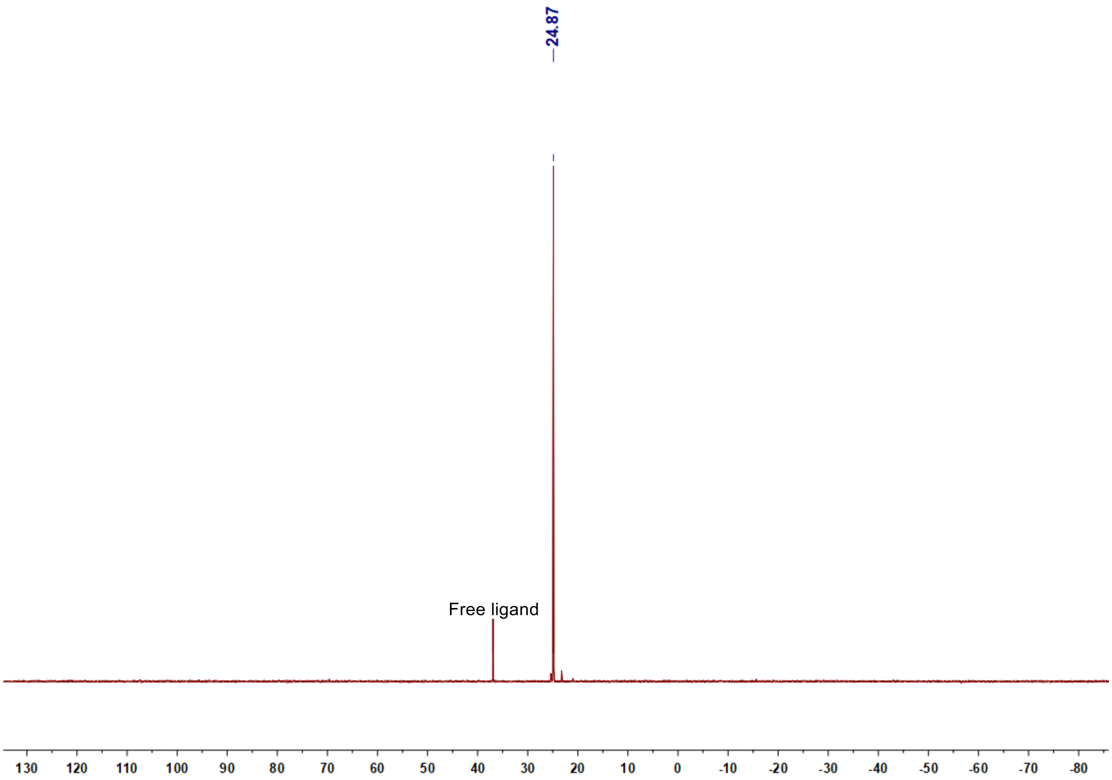

Figure S53. ^{31}P NMR (162 MHz, C_6D_6) spectrum of **Mg-4**

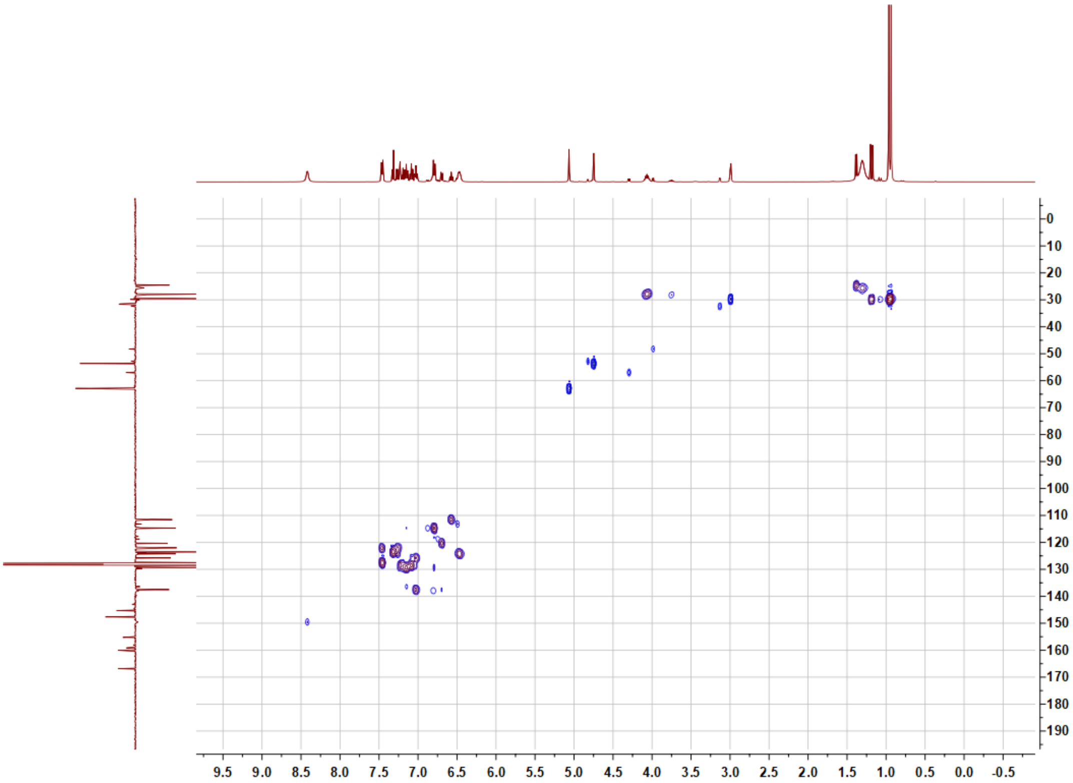

Figure S54. HSQC spectrum of **Mg-4** in C_6D_6

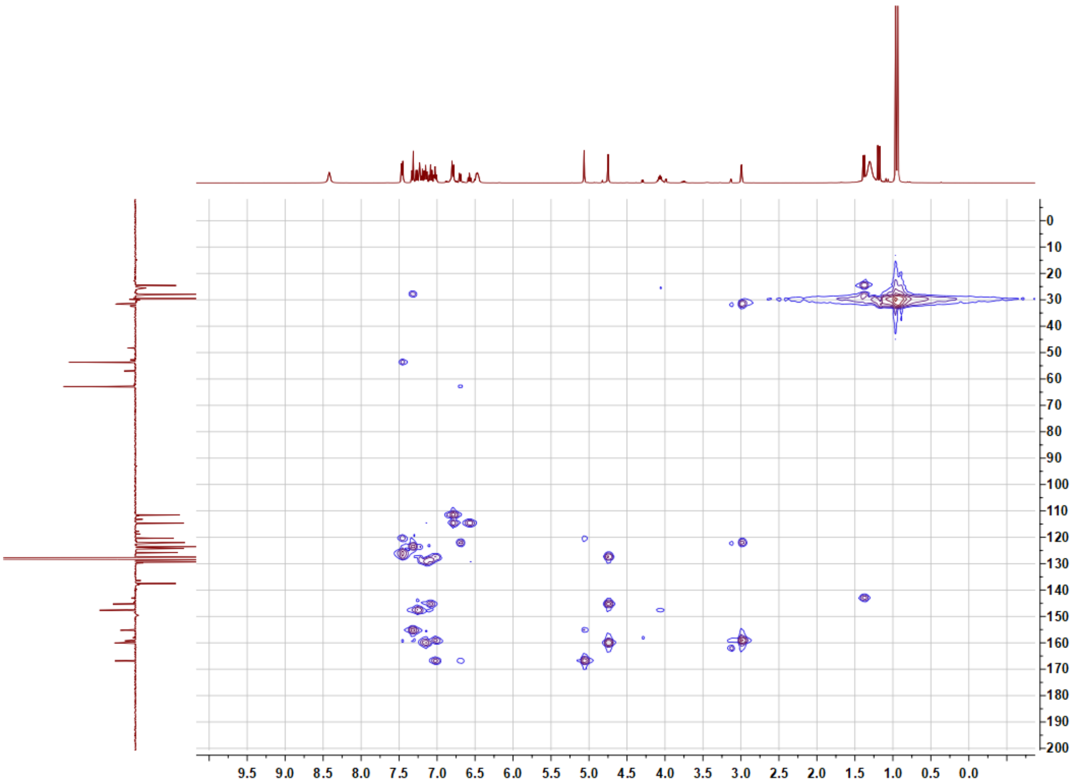

Figure S55. HMBC spectrum of **Mg-4** in C_6D_6

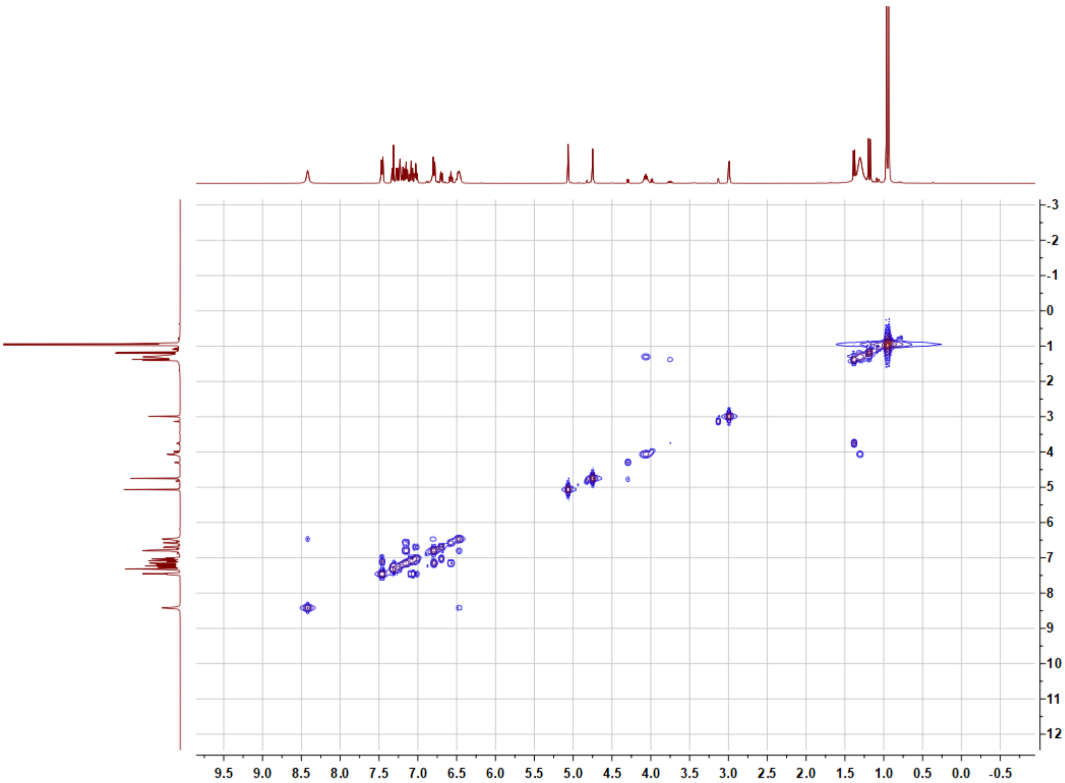

Figure S56. H-H COSY spectrum of **Mg-4** in C_6D_6

4 Catalytic hydrogenation of imines and *N*-heteroarenes

4.1 Condition optimization for the hydrogenation of 1a

Table S13. Catalyst optimization for the hydrogenation of 1a

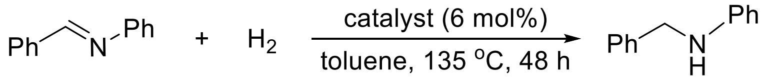

1a **2a**

entry	catalyst	yield (%)
1	**Mg-1a**	9
2	**Mg-2a**	99
3	**Mg-2b**	98
4	**Mg-2c**	99
5	**Mg-3**	63
6	**Mg-4**	81
7	**L1**	no reaction
8	$^{n}Bu_2Mg$	34[a]
9	$MgBr_2$	<3
10	EtMgBr	46[b]
11	NaHMDS	55

Reaction conditions: **1a** (0.3 mmol), catalyst (6 mol%), H_2 (10 bar), toluene (1 mL), 135 °C, 48 h, reaction yields were determined by ^{1}H NMR using 1,3,5-trimethoxybenzene as the internal standard. [a]7% of *N*-(1-phenylpentyl)aniline was detected as the byproduct. [b]5% of *N*-(1-phenylpropyl)aniline was detected as the byproduct.

Table S14. Condition optimization for the hydrogenation of 1a

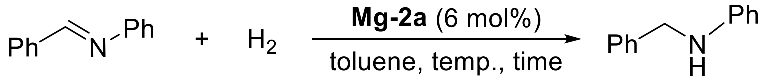

1a **2a**

entry	H_2 (bar)	temp. (°C)	time (h)	yield (%)
1	10	135	48	99
2	7	135	48	97
3	5	135	48	91
4	10	150	48	>99
5	10	120	48	85
6	10	135	60	>99
7	10	135	36	95
8	10	135	24	88

Reaction conditions: **1a** (0.3 mmol), **Mg-2a** (6 mol%), solvent (1 mL), 48 h, reaction yields were determined by ^{1}H NMR using 1,3,5-trimethoxybenzene as the internal standard.

4.2 Condition optimization for the hydrogenation of 3a

Table S15. Condition optimization for the hydrogenation of 3a

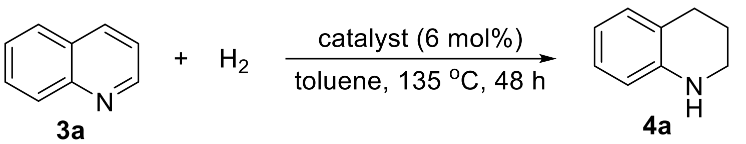

entry	catalyst	yield (%)
1	**Mg-2a**	95
2	**Mg-2b**	96
3	**Mg-3**	39
4	**L1**	no reaction
5	^{n}Bu$_2$Mg	27[a]
6	$MgBr_2$	no reaction
7	EtMgBr	46[b]
8	NaHMDS	<3

Reaction conditions: **3a** (0.3 mmol), catalyst (6 mol%), H_2 (10 bar), toluene (1 mL), 135 °C, 48 h, reaction yields were determined by ^{1}H NMR using 1,3,5-trimethoxybenzene as the internal standard. [a]5% of 2-butyl-1,2,3,4-tetrahydroquinoline was detected as the byproduct. [b]6% of 2-ethyl-1,2,3,4-tetrahydroquinoline was detected as the byproduct.

4.3 Synthesis and characterization of imines and *N*-heteroarenes

Imines **1a**, **1f**, **1k**, and **1p** were commercially available. **1b-1e**, **1g-1j**, **1l-o**, and **1q** were prepared according to the literature procedures.[7] **1r-1aa** were prepared as described below:

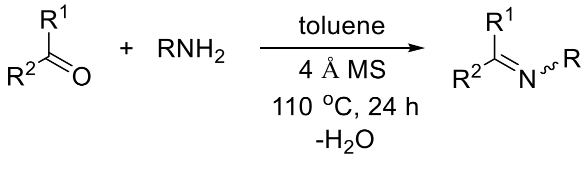

To a pressure tube were added 4 Å molecular sieve (~1 g/mmol), toluene (1 M), ketone (1.0 equiv), and amine (1.0 equiv). The tube was sealed and heated at 110 °C until the full consumption of ketone and amine. The solution was cooled to room temperature and then filtered to provide a clear solution. The desired imine was obtained after the removal of excess solvent. Liquid imines were used without further purification, and solid imines were further purified by recrystallization using the mixed

solvent of EtOAc and hexane.

N-Heteroarenes **3a-3b**, **3d**, **3h**, **3v-3x**, **3z**, and **3ac** were commercially available. **3c**,[8] **3e-3g**,[9] **3j-3s**,[10] **3t**,[11] **3u**,[12] **3aa-3ab**[13] were prepared according to the literature procedures. **3i** and **3y** were prepared by Suzuki coupling reaction using phenylboronic acid and corresponding bromides.

Characterization data of imines:

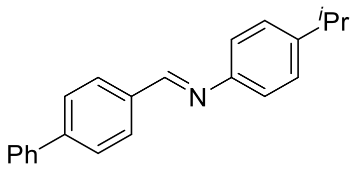

(*E*)-1-([1,1'-Biphenyl]-4-yl)-*N*-(4-isopropylphenyl)methanimine (1l). Purified by recrystallization using a mixed solvent of EtOAc and hexane. Yellowish solid, 345.5 mg, and 77% yield (1.5 mmol scale).

^{1}H NMR (300 MHz, $CDCl_3$) δ 8.57 (s, 1H), 8.02 (d, *J* = 8.3 Hz, 2H), 7.75 (d, *J* = 8.3 Hz, 2H), 7.70 (dd, *J* = 8.3, 1.1 Hz, 2H), 7.52 (t, *J* = 7.4 Hz, 2H), 7.47 – 7.40 (m, 1H), 7.33 (d, *J* = 8.4 Hz, 2H), 7.26 (d, *J* = 8.4 Hz, 2H), 3.08 – 2.91 (m, 1H), 1.34 (d, *J* = 6.9 Hz, 6H).

^{13}C NMR (75 MHz, $CDCl_3$) δ 159.21, 149.92, 147.00, 143.99, 140.46, 135.52, 129.33, 129.02, 127.98, 127.54, 127.31, 127.27, 121.04, 33.86, 24.22.

GC-MS *m/z* calcd. for $C_{22}H_{21}N$ $[M]^+$: 299.2, found: 299.1.

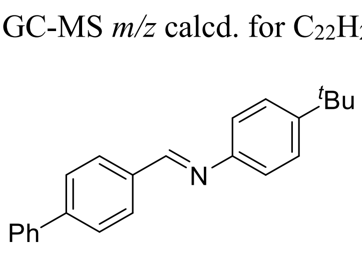

(*E*)-1-([1,1'-Biphenyl]-4-yl)-*N*-(4-(*tert*-butyl)phenyl)methanimine (1m). Purified by recrystallization using a mixed solvent of EtOAc and hexane. Yellowish solid, 380.5 mg, and 81% yield (1.5 mmol scale).

^{1}H NMR (300 MHz, $CDCl_3$) δ 8.58 (s, 1H), 8.03 (d, *J* = 8.3 Hz, 2H), 7.76 (d, *J* = 8.3 Hz, 2H), 7.73 – 7.67 (m, 2H), 7.57 – 7.40 (m, 5H), 7.27 (d, *J* = 8.5 Hz, 2H), 1.42 (s,

9H).

^{13}C NMR (75 MHz, $CDCl_3$) δ 159.29, 149.54, 149.25, 143.99, 140.46, 135.52, 129.33, 129.02, 127.98, 127.54, 127.30, 126.17, 120.74, 34.65, 31.58.

GC-MS *m/z* calcd. for $C_{23}H_{23}N$ $[M]^+$: 313.2, found: 313.2.

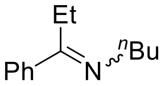

***N*-Butyl-1-phenylpropan-1-imine (1r).** Colorless oil, 359.4 mg, 95% yield, and 2.7/1 *E*/*Z* (2 mmol scale).

^{1}H NMR (300 MHz, $CDCl_3$) *E* isomer: δ 7.78 (dd, *J* = 6.7, 2.8 Hz, 2H), 7.46 – 7.31 (m, 3H), 3.57 (t, *J* = 7.1 Hz, 2H), 2.73 (q, *J* = 7.7 Hz, 2H), 1.84 – 1.70 (m, 2H), 1.57 – 1.42 (m, 2H), 1.13 (t, *J* = 7.7 Hz, 3H), 1.01 (t, *J* = 7.3 Hz, 3H). *Z* isomer: δ 7.46 – 7.31 (m, 3H), 7.13 – 7.05 (m, 2H), 3.20 (t, *J* = 7.1 Hz, 2H), 2.58 (q, *J* = 7.4 Hz, 2H), 1.66 – 1.54 (m, 2H), 1.37 – 1.24 (m, 2H), 1.11 (t, *J* = 7.7 Hz, 3H), 0.88 (t, *J* = 7.3 Hz, 3H).

^{13}C NMR (75 MHz, $CDCl_3$) *E* isomer: δ 169.49, 140.19, 129.13, 128.20, 126.80, 51.08, 33.44, 21.77, 20.81, 14.05, 11.5i9. *Z* isomer: δ 172.59, 138.84, 128.30, 127.78, 126.36, 52.83, 35.17, 33.34, 20.51, 13.91, 10.95.

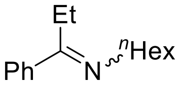

***N*-Hexyl-1-phenylpropan-1-imine (1s).** Colorless oil, 430.0 mg, 99% yield, and 2.7/1 *E*/*Z* (2 mmol scale).

^{1}H NMR (300 MHz, $CDCl_3$) major isomer: δ 7.78 (dd, *J* = 6.5, 3.1 Hz, 2H), 7.45 – 7.33 (m, 3H), 3.56 (t, *J* = 7.1 Hz, 2H), 2.74 (q, *J* = 7.7 Hz, 2H), 1.83 – 1.71 (m, 2H), 1.52 – 1.42 (m, 2H), 1.42 – 1.34 (m, 4H), 1.13 (t, *J* = 7.7 Hz, 3H), 0.94 (t, *J* = 6.9 Hz, 3H).

^{13}C NMR (75 MHz, $CDCl_3$) major isomer: δ 169.59, 140.32, 129.22, 128.31, 126.90, 51.54, 31.92, 31.37, 27.50, 22.79, 21.89, 14.18, 11.70.

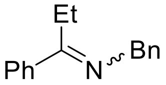

***N*-Benzyl-1-phenylpropan-1-imine (1t).** Yellowish oil, 198.9 mg, 89% yield, and 2/1 *E*/*Z* (1 mmol scale).

^{1}H NMR (300 MHz, $CDCl_3$) *E* isomer: δ 7.95 (dd, *J* = 6.3, 2.3 Hz, 2H), 7.55 – 7.40 (m, 8H), 4.89 (s, 2H), 2.89 (q, *J* = 7.7 Hz, 2H), 1.23 (t, *J* = 7.7 Hz, 3H). *Z* isomer: δ 7.56 – 7.27 (m, 8H), 7.21 (d, *J* = 6.5 Hz, 2H), 4.53 (s, 2H), 2.73 (q, *J* = 7.4 Hz, 2H), 1.24 (t, *J* = 7.4 Hz, 3H).

^{13}C NMR (75 MHz, $CDCl_3$) *E* isomer: δ 170.68, 140.78, 139.80, 129.60, 128.44, 128.35, 127.71, 127.07, 126.60, 54.87, 22.14, 11.67. *Z* isomer: δ 174.07, 140.70, 138.58, 132.91, 128.37, 128.22, 127.61, 126.51, 126.39, 56.82, 35.30, 10.97.

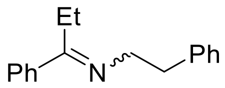

***N*-Phenethyl-1-phenylpropan-1-imine (1u).** Colorless oil, 441.1 mg, 93% yield, and 2/1 *E*/*Z* (2 mmol scale).

^{1}H NMR (300 MHz, $CDCl_3$) *E* isomer: δ 7.83 (dd, *J* = 6.5, 2.8 Hz, 2H), 7.49 – 7.43 (m, 2H), 7.37 (d, *J* = 4.2 Hz, 6H), 3.88 (t, *J* = 7.5 Hz, 2H), 3.17 (t, *J* = 7.5 Hz, 2H), 2.68 (q, *J* = 7.7 Hz, 2H), 1.07 (t, *J* = 7.7 Hz, 3H). *Z* isomer: δ 7.49 – 7.43 (m, 2H), 7.31 – 7.22 (m, 4H), 7.17 (d, *J* = 7.0 Hz, 2H), 6.93 – 6.86 (m, 2H), 3.54 (t, *J* = 7.4 Hz, 2H), 2.99 (t, *J* = 7.4 Hz, 2H), 2.61 (q, *J* = 7.7 Hz, 2H), 1.17 (t, *J* = 7.7 Hz, 3H).

^{13}C NMR (75 MHz, $CDCl_3$) *E* isomer: δ 170.31, 140.64, 139.99, 129.31, 128.97, 128.32, 128.27, 126.84, 126.03, 53.32, 37.81, 21.81, 11.44. *Z* isomer: δ 173.53, 140.29, 138.60, 129.03, 128.25, 128.14, 127.80, 126.18, 125.89, 54.75, 37.59, 35.17, 10.84.

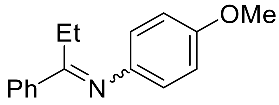

***N*-(4-Methoxyphenyl)-1-phenylpropan-1-imine (1v).** Purified by recrystallization using a mixed solvent of EtOAc and hexane. Yellowish solid, 411.3 mg, 86% yield, and 9/1 *E*/*Z* (2 mmol scale).

^{1}H NMR (300 MHz, $CDCl_3$) *E* isomer: δ 7.93 (dd, *J* = 6.4, 2.8 Hz, 2H), 7.56 – 7.38 (m, 3H), 6.92 (d, *J* = 8.7 Hz, 2H), 6.76 (d, *J* = 8.7 Hz, 2H), 3.82 (s, 3H), 2.70 (q, *J* = 7.6 Hz,

2H), 1.09 (t, J = 7.6 Hz, 3H).

^{13}C NMR (75 MHz, $CDCl_3$) E isomer: δ 7.94, 7.93, 7.92, 7.91, 7.46, 7.45, 7.44, 7.26, 6.93, 6.91, 6.77, 6.74, 3.82, 2.74, 2.71, 2.68, 2.66, 1.12, 1.09, 1.07.

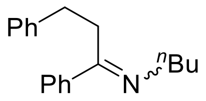

***N*-Butyl-1,3-diphenylpropan-1-imine (1w).** Colorless oil, 771.7 mg, 97% yield, and 2/1 E/Z (3 mmol scale).

^{1}H NMR (400 MHz, $CDCl_3$) E isomer: δ 7.95 – 7.84 (m, 2H), 7.55 – 7.46 (m, 4H), 7.34 – 7.25 (m, 4H), 3.55 (t, J = 7.1 Hz, 2H), 3.16 – 3.09 (m, 2H), 2.92 – 2.85 (m, 2H), 1.81 – 1.70 (m, 2H), 1.57 – 1.46 (m, 2H), 1.06 (t, J = 7.4 Hz, 3H). Z isomer: 7.44 – 7.34 (m, 8H), 7.19 (dd, J = 7.9, 1.2 Hz, 2H), 3.33 (t, J = 7.1 Hz, 2H), 2.98 (s, 4H), 1.70 – 1.63 (m, 2H), 1.42 – 1.31 (m, 2H), 0.96 (t, J = 7.4 Hz, 3H).

^{13}C NMR (101 MHz, $CDCl_3$) E isomer: δ 167.54, 141.10, 140.33, 129.44, 128.67, 128.50, 128.35, 127.06, 126.53, 51.52, 33.44, 33.24, 30.71, 20.91, 14.17. Z isomer: δ 170.79, 141.78, 138.84, 128.54, 128.52, 128.40, 128.12, 126.41, 125.93, 53.06, 43.59, 33.43, 32.64, 20.64, 14.06.

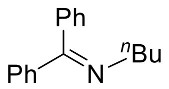

***N*-Butyl-1,1-diphenylmethanimine (1x).** Colorless oil, 441.1 mg, and 93% yield (2 mmol scale).

^{1}H NMR (300 MHz, $CDCl_3$) δ 7.64 (d, J = 6.4 Hz, 2H), 7.52 – 7.39 (m, 3H), 7.41 – 7.29 (m, 3H), 7.19 (d, J = 7.1 Hz, 2H), 3.41 (t, J = 7.0 Hz, 2H), 1.79 – 1.63 (m, 2H), 1.48 – 1.30 (m, 2H), 0.91 (t, J = 7.3 Hz, 3H).

^{13}C NMR (75 MHz, $CDCl_3$) δ 167.75, 140.18, 137.20, 129.78, 128.48, 128.36, 128.27, 128.09, 127.93, 53.72, 33.51, 20.72, 14.06.

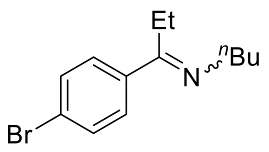

1-(4-Bromophenyl)-*N*-butylpropan-1-imine (1y). Colorless oil, 470.0 mg, 88% yield, and 3.4/1 *E*/*Z* (2 mmol scale).

^{1}H NMR (300 MHz, $CDCl_3$) *E* isomer: δ 7.62 (d, *J* = 8.6 Hz, 2H), 7.48 (d, *J* = 8.6 Hz, 2H), 3.50 (t, *J* = 7.1 Hz, 2H), 2.66 (q, *J* = 7.7 Hz, 2H), 1.78 – 1.65 (m, 2H), 1.52 – 1.37 (m, 2H), 1.08 (t, *J* = 7.1 Hz, 3H), 0.96 (t, *J* = 7.3 Hz, 3H). *Z* isomer: δ 7.52 (d, *J* = 8.5 Hz, 2H), 6.95 (d, *J* = 8.5 Hz, 2H), 3.15 (t, *J* = 7.1 Hz, 2H), 2.50 (q, *J* = 7.5 Hz, 2H), 1.60 – 1.47 (m, 2H), 1.32 – 1.19 (m, 2H), 1.05 (t, *J* = 7.1 Hz, 3H), 0.84 (t, *J* = 7.3 Hz, 3H).

^{13}C NMR (75 MHz, $CDCl_3$) *E* isomer: δ 168.46, 139.03, 131.45, 128.59, 123.69, 51.27, 33.46, 21.66, 20.90, 14.15, 11.63. *Z* isomer: δ 171.40, 137.67, 131.69, 128.25, 122.04, 53.05, 35.15, 33.37, 20.61, 14.02, 10.98.

GC-MS *m/z* calcd. for $C_{13}H_{18}BrN$ $[M]^+$: 267.1, found: 267.1.

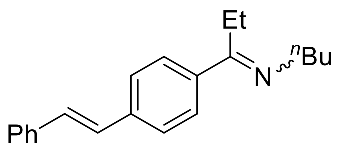

***N*-Butyl-1-(4-((*E*)-styryl)phenyl)propan-1-imine (1z).** Purified by recrystallization using a mixed solvent of EtOAc and hexane. Yellowish solid, 662.5 mg, 65% yield, and 2.9/1 *E*/*Z* (3.5 mmol scale).

^{1}H NMR (400 MHz, $CDCl_3$) *E* isomer: δ 7.86 (d, *J* = 8.3 Hz, 2H), 7.61 (d, *J* = 7.3 Hz, 4H), 7.48 – 7.42 (m, 2H), 7.35 (dd, *J* = 13.4, 6.2 Hz, 1H), 7.22 (s, 1H), 7.17 (d, *J* = 7.9 Hz, 1H), 3.63 (t, *J* = 7.1 Hz, 2H), 2.80 (q, *J* = 7.7 Hz, 2H), 1.89 – 1.78 (m, 2H), 1.62 – 1.50 (m, 2H), 1.21 (t, *J* = 7.7 Hz, 3H), 1.08 (t, *J* = 7.4 Hz, 3H). *Z* isomer: δ 7.60 (d, *J* = 8.1 Hz, 4H), 7.49 – 7.41 (m, 2H), 7.35 (dd, *J* = 13.4, 6.2 Hz, 1H), 7.21 (s, 4H), 3.31 (t, *J* = 7.1 Hz, 2H), 2.65 (q, *J* = 7.4 Hz, 2H), 1.71 – 1.62 (m, 2H), 1.43 – 1.31 (m, 2H), 1.18 (t, *J* = 7.7 Hz, 3H), 0.95 (t, *J* = 7.4 Hz, 3H).

^{13}C NMR (75 MHz, $CDCl_3$) δ *E* isomer: 169.09, 139.32, 138.28, 137.34, 129.30, 128.79,

128.33, 127.82, 127.32, 126.67, 126.49, 51.27, 33.59, 21.73, 20.96, 14.20, 11.79. *Z* isomer: δ 172.42, 138.07, 137.24, 137.05, 129.42, 128.82, 128.09, 127.92, 127.03, 126.67, 126.49, 53.05, 35.23, 33.50, 20.65, 14.07, 11.15.

GC-MS *m/z* calcd. for $C_{21}H_{25}N$ $[M]^+$: 291.2, found: 291.1.

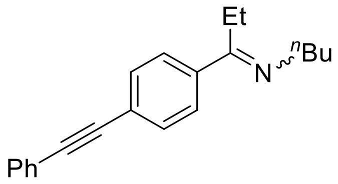

***N*-Butyl-1-(4-(phenylethynyl)phenyl)propan-1-imine (1aa).** Yellowish oil, 777.3 mg, 96% yield, and 5/1 *E*/*Z* (2.8 mmol scale).

^{1}H NMR (300 MHz, $CDCl_3$) *E* isomer: δ 7.81 (d, *J* = 8.3 Hz, 2H), 7.65 – 7.53 (m, 5H), 7.43 – 7.32 (m, 2H), 3.59 (t, *J* = 7.0 Hz, 2H), 2.73 (q, *J* = 7.7 Hz, 2H), 1.88 – 1.72 (m, 2H), 1.57 – 1.44 (m, 2H), 1.14 (t, *J* = 7.7 Hz, 3H), 1.04 (t, *J* = 7.3 Hz, 3H). *Z* isomer: δ 7.42 – 7.35 (m, 5H), 7.30 – 7.25 (m, 1H), 7.21 (d, *J* = 7.4 Hz, 1H), 7.11 (d, *J* = 8.1 Hz, 2H), 3.24 (t, *J* = 7.1 Hz, 2H), 2.59 (q, *J* = 7.5 Hz, 2H), 1.68 – 1.57 (m, 2H), 1.41 – 1.25 (m, 2H), 1.14 (t, *J* = 6.6 Hz, 3H), 0.91 (t, *J* = 7.3 Hz, 3H).

^{13}C NMR (75 MHz, $CDCl_3$) *E* isomer: δ 168.78, 139.72, 131.64, 131.53, 128.37, 128.35, 126.85, 124.10, 123.24, 90.61, 89.41, 51.23, 33.46, 21.64, 20.87, 14.12, 11.62. *Z* isomer: δ 171.93, 138.68, 131.60, 131.53, 128.45, 128.41, 126.58, 123.09, 122.97, 90.10, 88.89, 52.95, 35.00, 33.34, 20.55, 13.97, 10.98.

GC-MS *m/z* calcd. for $C_{21}H_{23}N$ $[M]^+$: 289.2, found: 289.1.

4.4 Procedures of catalytic hydrogenation reactions and characterization of amine products

General procedures:

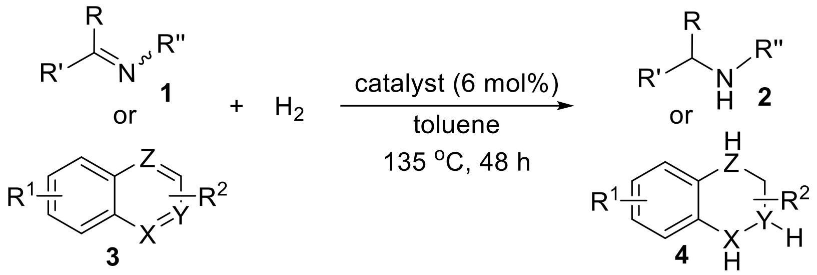

In a N_2 glovebox, substrates **1** or **3** (0.3 mmol) and magnesium complexes were added to a 9 mL narrow-necked tube with a stirring bar and dissolved by toluene (1 mL). The small tube was put into a 90 mL Fischer-Porter tube and 2 mL of toluene was added to the Fischer-Porter tube. The Fischer-Porter tube was closed, taken out of the glovebox, and then charged with 10 bar of H_2. The reaction was heated at 135 °C for 48 h and then cooled to room temperature. The solution was concentrated under reduced pressure. The resulting residue was transferred to an NMR tube to determine the reaction yield using 1,3,5-trimethoxybenzene as the internal standard. The isolated products were purified by column chromatography using a flash silica gel column to obtain the desired products.

Characterization data of selected amine products:

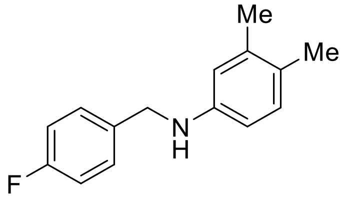

***N*-(4-fluorobenzyl)-3,4-dimethylaniline (2k).** Purified by column chromatography (eluent: PE/EtOAc = 30/1 to 3/1, v/v) using a short flash silica gel column. Yellowish oil, 59.8 mg, and 87% yield.

^{1}H NMR (300 MHz, $CDCl_3$) δ 7.43 – 7.33 (m, 2H), 7.07 (d, *J* = 8.6 Hz, 2H), 6.99 (d, *J* = 8.0 Hz, 1H), 6.52 (s, 1H), 6.44 (d, *J* = 8.0 Hz, 1H), 4.31 (s, 2H), 3.83 (br, 1H), 2.24 (s, 3H), 2.21 (s, 3H).

^{13}C NMR (75 MHz, $CDCl_3$) δ 162.09 (d, *J* = 244.8 Hz), 146.28, 137.46, 135.58 (d, *J* = 3.1 Hz), 130.42, 129.07 (d, *J* = 8.0 Hz), 125.84, 115.46 (d, *J* = 21.3 Hz), 114.88, 110.37, 48.01, 20.14, 18.79.

^{19}F NMR (282 MHz, $CDCl_3$) δ -116.73.

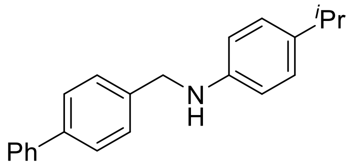

***N*-([1,1'-Biphenyl]-4-ylmethyl)-4-isopropylaniline (2l).** Purified by column chromatography (eluent: PE/EtOAc = 30/1 to 3/1, v/v) using a short flash silica gel column. White solid, 84.0 mg, and 93% yield.

^{1}H NMR (300 MHz, $CDCl_3$) δ 7.71 – 7.59 (m, 4H), 7.55 – 7.45 (m, 4H), 7.42 – 7.35 (m, 1H), 7.12 (d, J = 8.3 Hz, 2H), 6.67 (d, J = 8.3 Hz, 2H), 4.40 (s, 2H), 3.96 (br, 1H), 2.96 – 2.77 (m, 1H), 1.27 (d, J = 6.9 Hz, 6H).

^{13}C NMR (75 MHz, $CDCl_3$) δ 146.34, 141.00, 140.25, 138.92, 138.26, 128.89, 128.07, 127.45, 127.35, 127.26, 127.17, 113.04, 48.46, 33.29, 24.38.

GC-MS *m/z* calcd. for $C_{22}H_{23}N$ $[M]^+$: 301.2, found: 301.1.

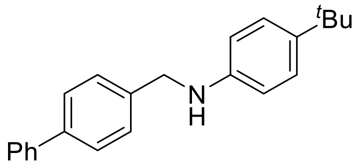

***N*-([1,1'-Biphenyl]-4-ylmethyl)-4-(*tert*-butyl)aniline (2m).** Purified by column chromatography (eluent: PE/EtOAc = 30/1 to 3/1, v/v) using a short flash silica gel column. White solid, 87.0 mg, and 92% yield.

^{1}H NMR (300 MHz, $CDCl_3$) δ 7.73 – 7.61 (m, 4H), 7.55 – 7.47 (m, 4H), 7.45 – 7.37 (m, 1H), 7.29 (d, J = 8.6 Hz, 2H), 6.69 (d, J = 8.6 Hz, 2H), 4.42 (s, 2H), 4.02 (br, 1H), 1.36 (s, 9H).

^{13}C NMR (75 MHz, $CDCl_3$) δ 145.97, 141.01, 140.51, 140.26, 138.93, 128.89, 128.08, 127.46, 127.35, 127.18, 126.17, 112.73, 48.44, 33.99, 31.68.

GC-MS *m/z* calcd. for $C_{23}H_{25}N$ $[M]^+$: 315.2, found: 315.2.

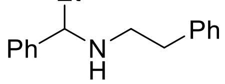

***N*-Phenethyl-1-phenylpropan-1-amine (2u).** Purified by column chromatography

(eluent: PE/EtOAc = 30/1 to 3/1, v/v) using a short flash silica gel column. Colorless oil, 71.0 mg, and 99% yield.

^{1}H NMR (300 MHz, CDCl$_3$) δ 7.42 – 7.15 (m, 10H), 3.62 – 3.46 (m, 1H), 2.89 – 2.68 (m, 4H), 1.87 – 1.59 (m, 2H), 0.82 (t, *J* = 7.4 Hz, 3H).

^{13}C NMR (75 MHz, CDCl$_3$) δ 144.17, 140.24, 128.79, 128.47, 128.35, 127.41, 126.96, 126.15, 65.10, 48.96, 36.53, 31.05, 10.85.

GC-MS *m/z* calcd. for $C_{17}H_{21}N$ [M]$^+$: 239.2, found: 239.2.

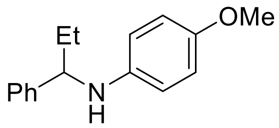

4-Methoxy-*N*-(1-phenylpropyl)aniline (2v). Purified by column chromatography (eluent: PE/EtOAc = 30/1 to 3/1, v/v) using a short flash silica gel column. Colorless oil, 61.5 mg, and 85% yield.

^{1}H NMR (300 MHz, CDCl$_3$) δ 7.41 – 7.31 (m, 4H), 7.30 – 7.21 (m, 1H), 6.73 (d, *J* = 7.5 Hz, 2H), 6.51 (d, *J* = 7.5 Hz, 2H), 4.19 (t, *J* = 6.6 Hz, 1H), 3.72 (s, 3H), 1.97 – 1.71 (m, 2H), 0.98 (t, *J* = 7.3 Hz, 3H).

^{13}C NMR (75 MHz, CDCl$_3$) δ 151.95, 144.29, 141.95, 128.58, 126.95, 126.67, 114.89, 114.58, 60.69, 55.87, 31.82, 10.94.

GC-MS *m/z* calcd. for $C_{16}H_{19}NO$ [M]$^+$: 241.1, found: 241.1.

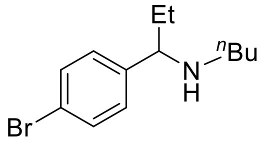

***N*-(1-(4-Bromophenyl)propyl)butan-1-amine (2y).** Purified by column chromatography (eluent: PE/EtOAc = 30/1 to 3/1, v/v) using a short flash silica gel column. Colorless oil, 72.3 mg, and 92% yield.

^{1}H NMR (300 MHz, CDCl$_3$) δ 7.43 (d, *J* = 8.4 Hz, 2H), 7.16 (d, *J* = 8.4 Hz, 2H), 3.44 (dd, *J* = 7.7, 5.9 Hz, 1H), 2.51 – 2.29 (m, 2H), 1.78 – 1.63 (m, 1H), 1.63 – 1.46 (m, 1H), 1.47 – 1.20 (m, 5H), 0.86 (t, *J* = 7.2 Hz, 3H), 0.78 (t, *J* = 7.4 Hz, 3H).

^{13}C NMR (75 MHz, $CDCl_3$) δ 143.66, 131.42, 129.20, 120.49, 64.80, 47.62, 32.52, 31.14, 20.57, 14.10, 10.78.

GC-MS *m/z* calcd. for $C_{13}H_{20}BrN$ $[M]^+$: 269.1, found: 269.1.

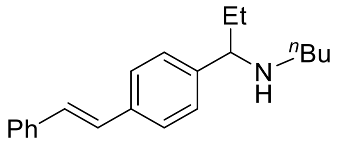

(*E*)-*N*-(1-(4-Styrylphenyl)propyl)butan-1-amine (2z). Purified by column chromatography (eluent: PE/EtOAc = 30/1 to 3/1, v/v) using a short flash silica gel column. Colorless oil, 86.2 mg, and 98% yield.

^1H NMR (300 MHz, $CDCl_3$) δ 7.53 (t, *J* = 8.4 Hz, 4H), 7.39 (t, *J* = 7.5 Hz, 2H), 7.34 – 7.27 (m, 3H), 7.14 (s, 2H), 3.53 (dd, *J* = 7.6, 6.0 Hz, 1H), 2.57 – 2.40 (m, 2H), 1.88 – 1.74 (m, 1H), 1.74 – 1.63 (m, 1H), 1.60 (s, 1H), 1.54 – 1.42 (m, 2H), 1.41 – 1.24 (m, 2H), 0.91 (t, *J* = 7.4 Hz, 3H), 0.86 (t, *J* = 7.5 Hz, 3H).

^{13}C NMR (75 MHz, $CDCl_3$) δ 144.16, 137.62, 136.10, 128.77, 128.71, 128.26, 127.79, 127.59, 126.57, 126.56, 65.10, 47.66, 32.54, 31.04, 20.61, 14.13, 10.93.

GC-MS *m/z* calcd. for $C_{21}H_{27}N$ $[M]^+$: 293.2, found: 293.1.

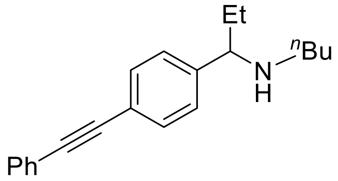

***N*-(1-(4-(Phenylethynyl)phenyl)propyl)butan-1-amine (2aa).** Purified by column chromatography (eluent: PE/EtOAc = 30/1 to 3/1, v/v) using a short flash silica gel column. Yellowish oil, 83.0 mg, and 95% yield.

^1H NMR (300 MHz, $CDCl_3$) δ 7.61 – 7.49 (m, 4H), 7.41 – 7.34 (m, 3H), 7.30 (d, *J* = 7.8 Hz, 2H), 3.52 (dd, *J* = 7.7, 5.9 Hz, 1H), 2.55 – 2.36 (m, 2H), 1.86 – 1.58 (m, 2H), 1.52 – 1.25 (m, 5H), 0.90 (t, *J* = 7.2 Hz, 3H), 0.83 (t, *J* = 7.4 Hz, 3H).

^{13}C NMR (75 MHz, $CDCl_3$) δ 145.10, 131.69, 131.68, 128.44, 128.24, 127.50, 123.57, 121.72, 89.61, 89.08, 65.16, 47.65, 32.55, 31.06, 20.59, 14.12, 10.84.

GC-MS *m/z* calcd. for $C_{21}H_{25}N$ $[M]^+$: 291.2, found: 291.2.

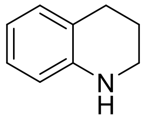

1,2,3,4-Tetrahydroquinoline (4a). Purified by column chromatography (eluent: PE/EtOAc = 50/1 to 20/1, v/v) using a short flash silica gel column. Yellowish oil, 36.7 mg, and 92% yield.

^{1}H NMR (300 MHz, $CDCl_3$) δ 7.07 – 6.98 (m, 2H), 6.67 (td, *J* = 7.4, 1.1 Hz, 1H), 6.52 (d, *J* = 7.9 Hz, 1H), 3.70 (br, 1H), 3.40 – 3.25 (m, 2H), 2.83 (t, *J* = 6.4 Hz, 2H), 2.07 – 1.93 (m, 2H).

^{13}C NMR (75 MHz, $CDCl_3$) δ 144.84, 129.55, 126.77, 121.47, 116.96, 114.24, 42.03, 27.04, 22.24.

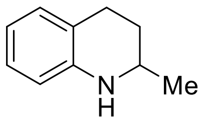

2-Methyl-1,2,3,4-tetrahydroquinoline (4b). Purified by column chromatography (eluent: PE/EtOAc = 50/1 to 20/1, v/v) using a short flash silica gel column. Yellowish oil, 35.7 mg, and 81% yield.

^{1}H NMR (400 MHz, $CDCl_3$) δ 7.03 – 6.92 (m, 2H), 6.62 (t, *J* = 7.3 Hz, 1H), 6.49 (d, *J* = 8.1 Hz, 1H), 3.48 – 3.36 (m, 2H), 2.86 (ddd, *J* = 17.0, 11.5, 5.6 Hz, 1H), 2.74 (ddd, *J* = 16.4, 5.6, 3.8 Hz, 1H), 1.99 – 1.89 (m, 1H), 1.61 (dddd, *J* = 12.8, 11.5, 10.0, 5.6 Hz, 1H), 1.23 (d, *J* = 6.3 Hz, 3H).

^{13}C NMR (101 MHz, $CDCl_3$) δ 144.87, 129.39, 126.81, 121.25, 117.12, 114.14, 47.29, 30.25, 26.72, 22.73.

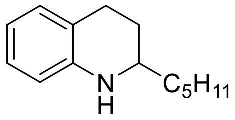

2-Pentyl-1,2,3,4-tetrahydroquinoline (4c). Purified by column chromatography (eluent: PE/EtOAc = 50/1 to 20/1, v/v) using a short flash silica gel column. Colorless oil, 53.0 mg, and 87% yield.

^{1}H NMR (300 MHz, $CDCl_3$) δ 7.01 (t, *J* = 7.0 Hz, 2H), 6.65 (td, *J* = 7.4, 1.0 Hz, 1H), 6.52 (d, *J* = 8.1 Hz, 1H), 3.79 (br, 1H), 3.28 (dtd, *J* = 9.4, 6.1, 2.9 Hz, 1H), 2.95 – 2.69

(m, 2H), 2.07 – 1.94 (m, 1H), 1.74 – 1.59 (m, 1H), 1.59 – 1.29 (m, 8H), 0.97 (t, *J* = 6.7 Hz, 3H).

^{13}C NMR (75 MHz, $CDCl_3$) δ 144.84, 129.33, 126.78, 121.46, 116.96, 114.12, 51.69, 36.79, 32.07, 28.22, 26.54, 25.50, 22.76, 14.17.

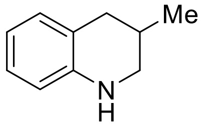

3-Methyl-1,2,3,4-tetrahydroquinoline (4d). Purified by column chromatography (eluent: PE/EtOAc = 50/1 to 20/1, v/v) using a short flash silica gel column. Colorless oil, 41.5 mg, and 94% yield.

^{1}H NMR (300 MHz, $CDCl_3$) δ 7.05 – 6.93 (m, 2H), 6.65 (td, *J* = 7.4, 1.1 Hz, 1H), 6.52 (d, *J* = 7.9 Hz, 1H), 3.61 (br, 1H), 3.30 (ddd, *J* = 11.0, 3.7, 2.0 Hz, 1H), 2.93 (dd, *J* = 11.0, 9.7 Hz, 1H), 2.82 (ddd, *J* = 16.0, 4.8, 1.7 Hz, 1H), 2.47 (dd, *J* = 16.0, 10.2 Hz, 1H), 2.19 – 1.98 (m, 1H), 1.09 (d, *J* = 6.6 Hz, 3H).

^{13}C NMR (75 MHz, $CDCl_3$) δ 144.37, 129.62, 126.78, 121.20, 117.02, 113.97, 48.94, 35.57, 27.27, 19.14.

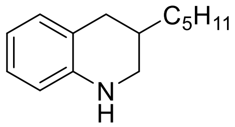

3-Pentyl-1,2,3,4-tetrahydroquinoline (4e). Purified by column chromatography (eluent: PE/EtOAc = 50/1 to 20/1, v/v) using a short flash silica gel column. Colorless oil, 55.5 mg, and 91% yield.

^{1}H NMR (300 MHz, $CDCl_3$) δ 7.04 (t, *J* = 7.6 Hz, 2H), 6.69 (t, *J* = 7.3 Hz, 1H), 6.55 (d, *J* = 7.8 Hz, 1H), 3.70 (br, 1H), 3.38 (ddd, *J* = 11.0, 3.4, 2.0 Hz, 1H), 3.05 – 2.93 (m, 1H), 2.89 (dd, *J* = 16.0, 4.2 Hz, 1H), 2.51 (dd, *J* = 16.0, 10.2 Hz, 1H), 2.08 – 1.90 (m, 1H), 1.56 – 1.32 (m, 8H), 1.00 (t, *J* = 6.7 Hz, 3H).

^{13}C NMR (75 MHz, $CDCl_3$) δ 144.67, 129.67, 126.75, 121.21, 116.98, 113.92, 47.44, 33.86, 33.84, 32.31, 32.14, 26.71, 22.76, 14.20.

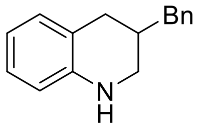

3-Benzyl-1,2,3,4-tetrahydroquinoline (4f). Purified by column chromatography (eluent: PE/EtOAc = 50/1 to 20/1, v/v) using a short flash silica gel column. Colorless oil, 60.9 mg, and 91% yield.

^{1}H NMR (300 MHz, $CDCl_3$) δ 7.37 (t, J = 7.1 Hz, 2H), 7.27 (t, J = 8.4 Hz, 3H), 7.01 (dd, J = 17.6, 7.6 Hz, 2H), 6.66 (t, J = 7.3 Hz, 1H), 6.53 (d, J = 7.9 Hz, 1H), 3.57 (br, 1H), 3.33 (ddd, J = 11.0, 3.3, 1.8 Hz, 1H), 3.02 (dd, J = 11.0, 8.8 Hz, 1H), 2.85 (dd, J = 16.0, 4.4 Hz, 1H), 2.79 – 2.65 (m, 2H), 2.58 (dd, J = 16.0, 9.2 Hz, 1H), 2.41 – 2.20 (m, 1H).

^{13}C NMR (75 MHz, $CDCl_3$) δ 144.50, 140.33, 129.81, 129.19, 128.45, 126.90, 126.16, 120.64, 117.13, 113.95, 46.68, 40.02, 34.21, 33.43.

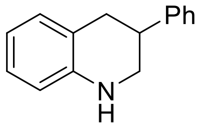

3-Phenyl-1,2,3,4-tetrahydroquinoline (4g). Purified by column chromatography (eluent: PE/EtOAc = 50/1 to 20/1, v/v) using a short flash silica gel column. Colorless oil, 54.6 mg, and 87% yield.

^{1}H NMR (300 MHz, $CDCl_3$) δ 7.46 – 7.36 (m, 2H), 7.30 (t, J = 6.3 Hz, 3H), 7.07 (t, J = 7.2 Hz, 2H), 6.70 (t, J = 7.4 Hz, 1H), 6.60 (d, J = 8.0 Hz, 1H), 3.71 (br, 1H), 3.55 – 3.45 (m, 1H), 3.38 (t, J = 10.6 Hz, 1H), 3.20 (ddd, J = 13.5, 8.0, 3.8 Hz, 1H), 3.12 – 2.94 (m, 2H).

^{13}C NMR (75 MHz, $CDCl_3$) δ 144.11, 143.96, 129.64, 128.72, 127.32, 127.08, 126.76, 121.46, 117.23, 114.18, 48.44, 38.77, 34.73.

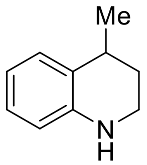

4-Methyl-1,2,3,4-tetrahydroquinoline (4h). Purified by column chromatography (eluent: PE/EtOAc = 50/1 to 20/1, v/v) using a short flash silica gel column. Yellowish oil, 42.4 mg, and 96% yield.

^{1}H NMR (300 MHz, $CDCl_3$) δ 7.09 (d, *J* = 7.5 Hz, 1H), 7.00 (t, *J* = 7.5 Hz, 1H), 6.67 (t, *J* = 7.4 Hz, 1H), 6.51 (d, *J* = 7.9 Hz, 1H), 3.48 (br, 1H), 3.41 – 3.22 (m, 2H), 3.02 – 2.85 (m, 1H), 2.02 (ddd, *J* = 13.0, 8.9, 4.7 Hz, 1H), 1.71 (dtd, *J* = 10.1, 6.3, 3.8 Hz, 1H), 1.33 (d, *J* = 7.0 Hz, 3H).

^{13}C NMR (75 MHz, $CDCl_3$) δ 144.27, 128.54, 126.82, 126.74, 117.09, 114.31, 39.10, 30.31, 29.95, 22.75.

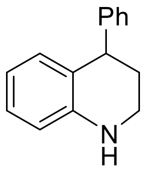

4-Phenyl-1,2,3,4-tetrahydroquinoline (4i). Purified by column chromatography (eluent: PE/EtOAc = 50/1 to 20/1, v/v) using a short flash silica gel column. White solid, 55.2 mg, and 88% yield.

^{1}H NMR (300 MHz, $CDCl_3$) δ 7.33 (t, *J* = 7.3 Hz, 2H), 7.28 – 7.21 (m, 1H), 7.18 (d, *J* = 7.2 Hz, 2H), 7.05 (t, *J* = 7.2 Hz, 1H), 6.79 (d, *J* = 7.3 Hz, 1H), 6.60 (t, *J* = 7.9 Hz, 2H), 4.18 (t, *J* = 6.0 Hz, 1H), 3.56 (br, 1H), 3.40 – 3.19 (m, 2H), 2.25 (ddd, *J* = 13.0, 9.3, 4.6 Hz, 1H), 2.09 (dtd, *J* = 10.6, 6.8, 3.8 Hz, 1H).

^{13}C NMR (75 MHz, $CDCl_3$) δ 146.77, 145.06, 130.55, 128.79, 128.39, 127.41, 126.22, 123.50, 117.14, 114.31, 42.93, 39.29, 31.21.

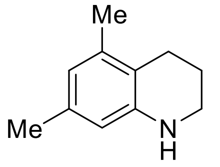

5,7-Dimethyl-1,2,3,4-tetrahydroquinoline (4j). Purified by column chromatography (eluent: PE/EtOAc = 50/1 to 20/1, v/v) using a short flash silica gel column. Colorless oil, 46.9 mg, and 97% yield.

^{1}H NMR (400 MHz, $CDCl_3$) δ 6.42 (s, 1H), 6.25 (s, 1H), 3.56 (br, 1H), 3.36 – 3.21 (m, 2H), 2.65 (t, *J* = 6.6 Hz, 2H), 2.24 (s, 3H), 2.19 (s, 3H), 2.07 – 1.97 (m, 2H).

^{13}C NMR (101 MHz, $CDCl_3$) δ 144.92, 137.15, 135.84, 120.12, 117.37, 113.08, 41.74, 23.88, 22.81, 21.05, 19.33.

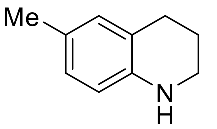

6-Methyl-1,2,3,4-tetrahydroquinoline (4k). Purified by column chromatography (eluent: PE/EtOAc = 50/1 to 20/1, v/v) using a short flash silica gel column. Yellowish oil, 41.0 mg, and 93% yield.

^{1}H NMR (300 MHz, $CDCl_3$) δ 6.88 – 6.75 (m, 2H), 6.48 – 6.42 (m, 1H), 3.51 (s, 1H), 3.34 – 3.27 (m, 2H), 2.78 (t, *J* = 6.4 Hz, 2H), 2.26 (s, 3H), 2.04 – 1.89 (m, 2H).

^{13}C NMR (75 MHz, $CDCl_3$) δ 142.47, 130.15, 127.32, 126.32, 121.68, 114.56, 42.26, 27.00, 22.52, 20.49.

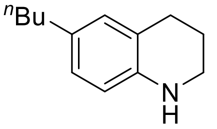

6-Butyl-1,2,3,4-tetrahydroquinoline (4l). Purified by column chromatography (eluent: PE/EtOAc = 50/1 to 20/1, v/v) using a short flash silica gel column. Colorless oil, 52.2 mg, and 92% yield.

^{1}H NMR (400 MHz, $CDCl_3$) δ 6.82 (d, *J* = 7.4 Hz, 2H), 6.45 (d, *J* = 8.0 Hz, 1H), 3.49 (br, 1H), 3.34 – 3.24 (m, 2H), 2.78 (t, *J* = 6.5 Hz, 2H), 2.55 – 2.45 (m, 2H), 2.01 – 1.91 (m, 2H), 1.65 – 1.52 (m, 2H), 1.39 (dq, *J* = 14.5, 7.3 Hz, 2H), 0.96 (t, *J* = 7.3 Hz, 3H).

^{13}C NMR (101 MHz, $CDCl_3$) δ 142.67, 131.71, 129.54, 126.72, 121.56, 114.49, 42.27, 34.92, 34.23, 27.07, 22.54 (2C), 14.12.

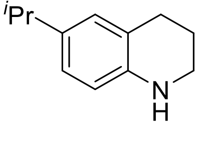

6-Isopropyl-1,2,3,4-tetrahydroquinoline (4m). Purified by column chromatography (eluent: PE/EtOAc = 50/1 to 20/1, v/v) using a short flash silica gel column. Yellowish oil, 50.4 mg, and 96% yield.

^{1}H NMR (300 MHz, $CDCl_3$) δ 6.91 – 6.83 (m, 2H), 6.47 (d, *J* = 7.9 Hz, 1H), 3.49 (br, 1H), 3.33 – 3.25 (m, 2H), 2.90 – 2.69 (m, 3H), 2.05 – 1.91 (m, 2H), 1.24 (d, *J* = 6.9 Hz, 6H).

^{13}C NMR (75 MHz, $CDCl_3$) δ 142.84, 137.76, 127.53, 124.71, 121.49, 114.53, 42.25, 33.32, 27.1k5, 24.41, 22.54.

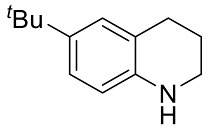

6-(*tert*-Butyl)-1,2,3,4-tetrahydroquinoline (4n). Purified by column chromatography (eluent: PE/EtOAc = 50/1 to 20/1, v/v) using a short flash silica gel column. Yellowish oil, 52.2 mg, and 92% yield.

^{1}H NMR (400 MHz, $CDCl_3$) δ 7.08 – 7.00 (m, 2H), 6.49 (d, *J* = 8.3 Hz, 1H), 3.50 (br, 1H), 3.35 – 3.29 (m, 2H), 2.81 (t, *J* = 6.4 Hz, 2H), 2.04 – 1.91 (m, 2H), 1.32 (s, 9H).

^{13}C NMR (101 MHz, $CDCl_3$) δ 142.51, 139.98, 126.42, 123.73, 121.05, 114.27, 42.23, 33.89, 31.71, 27.30, 22.56.

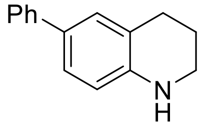

6-Phenyl-1,2,3,4-tetrahydroquinoline (4o). Purified by column chromatography (eluent: PE/EtOAc = 50/1 to 20/1, v/v) using a flash silica gel column. Colorless oil, 52.7 mg, and 84% yield.

^{1}H NMR (300 MHz, $CDCl_3$) δ 7.63 – 7.56 (m, 2H), 7.44 (t, *J* = 7.6 Hz, 2H), 7.35 – 7.24 (m, 3H), 6.58 (d, *J* = 8.5 Hz, 1H), 3.70 (s, 1H), 3.40 – 3.29 (m, 2H), 2.88 (t, *J* = 6.4 Hz, 2H), 2.07 – 1.95 (m, 2H).

^{13}C NMR (75 MHz, $CDCl_3$) δ 144.33, 141.59, 129.98, 128.68, 128.25, 126.34, 125.98,

125.59, 121.66, 114.54, 42.10, 27.23, 22.25.

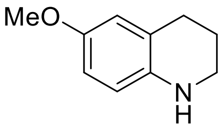

6-Methoxy-1,2,3,4-tetrahydroquinoline (4p). Purified by column chromatography (eluent: PE/EtOAc = 30/1 to 10/1, v/v) using a flash silica gel column. Colorless oil, 46.0 mg, and 94% yield.

^{1}H NMR (300 MHz, $CDCl_3$) δ 6.66 – 6.55 (m, 2H), 6.46 (d, *J* = 8.4 Hz, 1H), 3.74 (s, 3H), 3.41 (s, 1H), 3.31 – 3.21 (m, 2H), 2.77 (t, *J* = 6.5 Hz, 2H), 2.01 – 1.85 (m, 2H).

^{13}C NMR (75 MHz, $CDCl_3$) δ 151.91, 138.93, 122.96, 115.66, 114.96, 112.98, 55.88, 42.42, 27.25, 22.52.

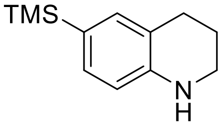

6-(Trimethylsilyl)-1,2,3,4-tetrahydroquinoline (4q). Purified by column chromatography (eluent: PE/EtOAc = 50/1 to 20/1, v/v) using a short flash silica gel column. Colorless oil, 49.2 mg, and 80% yield.

^{1}H NMR (300 MHz, $CDCl_3$) δ 7.19 – 7.07 (m, 2H), 6.50 (d, *J* = 7.8 Hz, 1H), 3.72 (br, 1H), 3.38 – 3.28 (m, 2H), 2.80 (t, *J* = 6.4 Hz, 2H), 2.08 – 1.86 (m, 2H), 0.25 (s, 9H).

^{13}C NMR (75 MHz, $CDCl_3$) δ 145.65, 134.86, 132.10, 126.41, 120.83, 113.84, 42.02, 27.11, 22.28, -0.66.

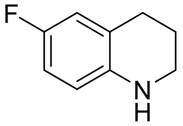

6-Fluoro-1,2,3,4-tetrahydroquinoline (4r). Purified by column chromatography (eluent: PE/EtOAc = 50/1 to 20/1, v/v) using a flash silica gel column. Colorless oil, 35.4 mg, and 78% yield.

^{1}H NMR (300 MHz, $CDCl_3$) δ 6.74 – 6.65 (m, 2H), 6.41 (dd, *J* = 9.5, 4.9 Hz, 1H), 3.40

(br, 1H), 3.33 – 3.20 (m, 2H), 2.75 (t, *J* = 6.4 Hz, 2H), 2.01 – 1.86 (m, 2H).

^{13}C NMR (75 MHz, $CDCl_3$) δ 155.58 (d, *J* = 234.6 Hz), 141.00 (d, *J* = 1.5 Hz), 122.92 (d, *J* = 6.7 Hz), 115.71 (d, *J* = 21.6 Hz), 115.05 (d, *J* = 7.6 Hz), 113.29 (d, *J* = 22.4 Hz). 42.18, 27.12 (d, *J* = 1.1 Hz), 22.09.

^{19}F NMR (282 MHz, $CDCl_3$) δ -129.30.

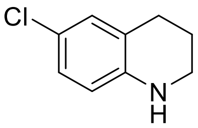

6-Chloro-1,2,3,4-tetrahydroquinoline (4s). Purified by column chromatography (eluent: PE/EtOAc = 50/1 to 20/1, v/v) using a short flash silica gel column. Colorless oil, 40.6 mg, and 81% yield.

^{1}H NMR (300 MHz, $CDCl_3$) δ 6.96 – 6.84 (m, 2H), 6.38 (d, *J* = 8.9 Hz, 1H), 3.42 (br, 1H), 3.32 – 3.23 (m, 2H), 2.73 (t, *J* = 6.4 Hz, 2H), 1.99 – 1.83 (m, 2H).

^{13}C NMR (75 MHz, $CDCl_3$) δ 143.39, 129.13, 126.61, 122.99, 121.28, 115.21, 41.96, 26.98, 21.85.

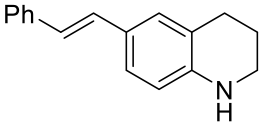

(*E*)-6-Styryl-1,2,3,4-tetrahydroquinoline (4t). Purified by column chromatography (eluent: PE/EtOAc = 100/1 to 30/1, v/v) using a flash silica gel column. White solid, 59.3 mg, and 84% yield.

^{1}H NMR (300 MHz, $CDCl_3$) δ 7.50 (d, *J* = 7.4 Hz, 2H), 7.36 (t, *J* = 7.6 Hz, 2H), 7.28 – 7.15 (m, 3H), 7.04 (d, *J* = 16.3 Hz, 1H), 6.91 (d, *J* = 16.3 Hz, 1H), 6.47 (d, *J* = 7.9 Hz, 1H), 3.74 (br, 1H), 3.40 – 3.29 (m, 2H), 2.81 (t, *J* = 6.4 Hz, 2H), 2.04 – 1.89 (m, 2H).

^{13}C NMR (75 MHz, $CDCl_3$) δ 144.74, 138.36, 129.18, 128.67, 127.95, 126.69, 126.38, 126.07, 125.54, 124.07, 121.36, 114.24, 42.07, 27.15, 22.17.

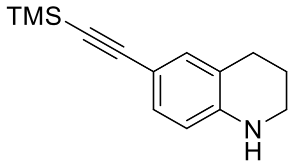

6-((Trimethylsilyl)ethynyl)-1,2,3,4-tetrahydroquinoline (4u). Purified by column chromatography (eluent: PE/EtOAc = 50/1 to 20/1, v/v) using a flash silica gel column. Colorless oil, 61.2 mg, and 89% yield.

^{1}H NMR (300 MHz, $CDCl_3$) δ 7.14 – 6.99 (m, 2H), 6.33 (d, *J* = 8.8 Hz, 1H), 3.98 (s, 1H), 3.34 – 3.24 (m, 2H), 2.70 (t, *J* = 6.3 Hz, 2H), 1.97 – 1.82 (m, 2H), 0.22 (s, 9H).

^{13}C NMR (75 MHz, $CDCl_3$) δ 145.23, 133.41, 130.98, 120.87, 113.50, 110.41, 106.89, 90.72, 41.93, 26.87, 21.85, 0.36.

GC-MS *m/z* calcd. for $C_{14}H_{19}NSi$ $[M]^+$: 229.1, found: 229.1.

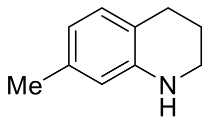

7-Methyl-1,2,3,4-tetrahydroquinoline (4v). Purified by column chromatography (eluent: PE/EtOAc = 50/1 to 20/1, v/v) using a short flash silica gel column. Yellowish oil, 40.2 mg, and 91% yield.

^{1}H NMR (300 MHz, $CDCl_3$) δ 6.90 (d, *J* = 7.6 Hz, 1H), 6.50 (d, *J* = 7.6 Hz, 1H), 6.36 (s, 1H), 3.61 (s, 1H), 3.37 – 3.26 (m, 2H), 2.79 (t, *J* = 6.4 Hz, 2H), 2.29 (s, 3H), 2.05 – 1.91 (m, 2H).

^{13}C NMR (75 MHz, $CDCl_3$) δ 144.64, 136.39, 129.44, 118.61, 117.99, 114.86, 42.09, 26.67, 22.47, 21.19.

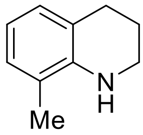

8-Methyl-1,2,3,4-tetrahydroquinoline (4w). Purified by column chromatography (eluent: PE/EtOAc = 50/1 to 20/1, v/v) using a short flash silica gel column. Colorless oil, 33.5 mg, and 76% yield.

^{1}H NMR (400 MHz, $CDCl_3$) δ 6.89 (dd, *J* = 10.2, 7.9 Hz, 2H), 6.58 (t, *J* = 7.4 Hz, 1H),

3.49 – 3.29 (m, 3H), 2.82 (t, J = 6.4 Hz, 2H), 2.10 (s, 3H), 2.03 – 1.91 (m, 2H).

^{13}C NMR (101 MHz, $CDCl_3$) δ 142.77, 127.96, 127.48, 121.33, 121.02, 116.55, 42.46, 27.40, 22.27, 17.26.

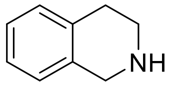

1,2,3,4-Tetrahydroisoquinoline (4x). Purified by column chromatography (eluent: EtOAc/MeOH = 1/1, v/v) using a flash silica gel column. Yellowish oil, 35.1 mg, and 88% yield.

^{1}H NMR (400 MHz, $CDCl_3$) δ 7.16 – 7.11 (m, 2H), 7.11 – 7.07 (m, 1H), 7.05 – 6.97 (m, 1H), 4.01 (s, 2H), 3.14 (t, J = 6.0 Hz, 2H), 2.80 (t, J = 6.0 Hz, 2H), 2.69 (s, 1H).

^{13}C NMR (101 MHz, $CDCl_3$) δ 135.71, 134.69, 129.32, 126.26, 126.10, 125.79, 48.15, 43.79, 29.07.

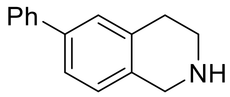

6-Phenyl-1,2,3,4-tetrahydroisoquinoline (4y). Purified by column chromatography (eluent: EtOAc/MeOH = 1/1, v/v) using a flash silica gel column. White solid, 53.3 mg, and 85% yield.

^{1}H NMR (300 MHz, $CDCl_3$) δ 7.61 (d, J = 7.4 Hz, 2H), 7.46 (t, J = 7.5 Hz, 2H), 7.42 – 7.31 (m, 3H), 7.10 (d, J = 7.9 Hz, 1H), 4.07 (s, 2H), 3.19 (t, J = 5.3 Hz, 2H), 2.89 (t, J = 5.3 Hz, 2H), 2.11 (br, 1H).

^{13}C NMR (75 MHz, $CDCl_3$) δ 141.13, 139.10, 135.19, 135.10, 128.74, 128.00, 127.10, 127.02, 126.72, 124.65, 48.13, 43.92, 29.39.

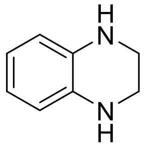

1,2,3,4-Tetrahydroquinoxaline (4z). Purified by column chromatography (eluent: PE/EtOAc = 50/1 to 5/1, v/v) using a flash silica gel column. Yellowish oil, 31.0 mg,

and 77% yield.

^{1}H NMR (300 MHz, $CDCl_3$) δ 6.66 – 6.58 (m, 2H), 6.56 – 6.47 (m, 2H), 3.59 (s, 2H), 3.41 (s, 4H).

^{13}C NMR (75 MHz, $CDCl_3$) δ 133.71, 118.72, 114.72, 41.37.

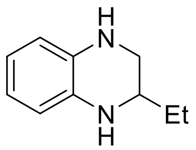

2-Ethyl-1,2,3,4-tetrahydroquinoxaline (4aa). Purified by column chromatography (eluent: PE/EtOAc = 50/1 to 5/1, v/v) using a flash silica gel column. Colorless oil, 39.4 mg, and 81% yield.

^{1}H NMR (300 MHz, $CDCl_3$) δ 6.65 – 6.57 (m, 2H), 6.56 – 6.48 (m, 2H), 3.54 (s, 2H), 3.38 (dd, *J* = 10.6, 2.4 Hz, 1H), 3.34 – 3.24 (m, 1H), 3.07 (dd, *J* = 10.6, 8.0 Hz, 1H), 1.60 – 1.45 (m, 2H), 1.02 (t, *J* = 7.5 Hz, 3H).

^{13}C NMR (75 MHz, $CDCl_3$) δ 133.60, 133.48, 118.82, 118.65, 114.52, 114.51, 51.78, 46.33, 27.20, 10.11.

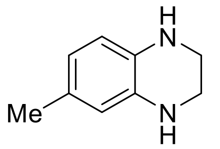

6-Methyl-1,2,3,4-tetrahydroquinoxaline (4ab). Purified by column chromatography (eluent: PE/EtOAc = 50/1 to 5/1, v/v) using a flash silica gel column. Colorless oil, 32.9 mg, and 74% yield.

^{1}H NMR (300 MHz, $CDCl_3$) δ 6.48 – 6.37 (m, 2H), 6.34 (s, 1H), 3.40 (s, 6H), 2.19 (s, 3H).

^{13}C NMR (75 MHz, $CDCl_3$) δ 133.83, 131.17, 128.49, 119.21, 115.54, 115.12, 41.66, 20.75.

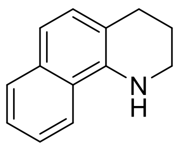

1,2,3,4-Tetrahydrobenzo[*h*]quinoline (4ac). Purified by column chromatography (eluent: PE/EtOAc = 50/1 to 20/1, v/v) using a flash silica gel column. Yellowish oil, 40.0 mg, and 82% yield.

^{1}H NMR (300 MHz, $CDCl_3$) δ 7.84 – 7.78 (m, 1H), 7.76 – 7.68 (m, 1H), 7.50 – 7.41 (m, 2H), 7.25 (d, J = 8.2 Hz, 1H), 7.19 (d, J = 8.3 Hz, 1H), 4.12 (br, 1H), 3.54 – 3.35 (m, 2H), 2.97 (t, J = 6.4 Hz, 2H), 2.15 – 2.00 (m, 2H).

^{13}C NMR (75 MHz, $CDCl_3$) δ 139.09, 133.12, 128.66, 128.58, 124.98, 124.76, 123.32, 119.53, 116.95, 115.83, 42.46, 27.53, 22.19.

5 Mechanistic studies

To get a deeper understanding of the mechanism, the monitor experiment by using **Mg-2a** (10 mol%) as a catalyst in the catalytic hydrogenation of **1a** (0.1 mmol) was conducted. **Mg-4** could be observed as the major magnesium species by ^{31}P NMR (Figure S57). The generation of **Mg-4** was also confirmed by ^{1}H NMR spectrum (Figure S58). These results suggest that **Mg-4** is a key intermediate in the hydrogenation reaction.

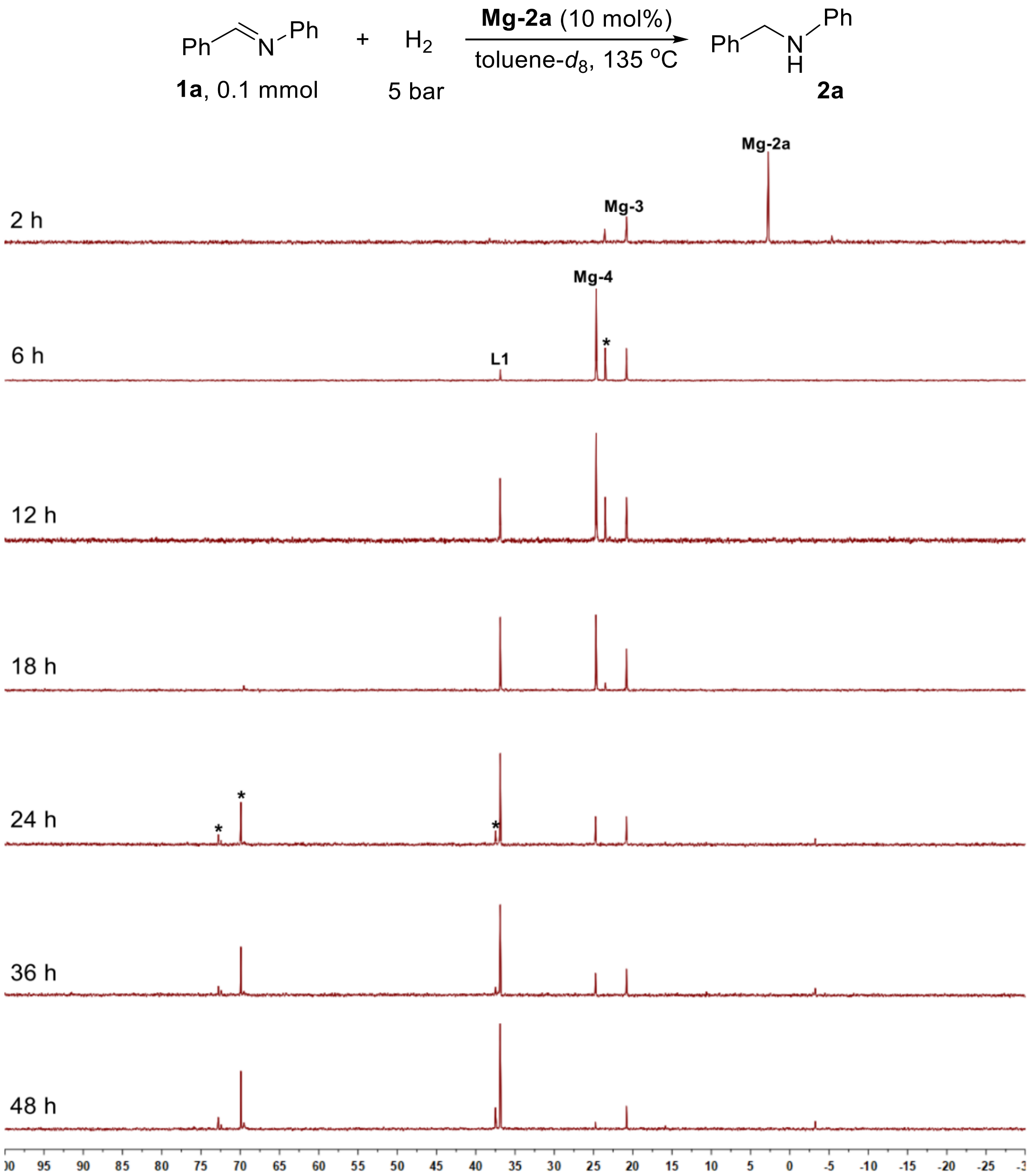

Figure S57. ^{31}P NMR spectra of monitor reaction for **Mg-2a** catalyzed hydrogenation of **1a** (*unidentified impurity)

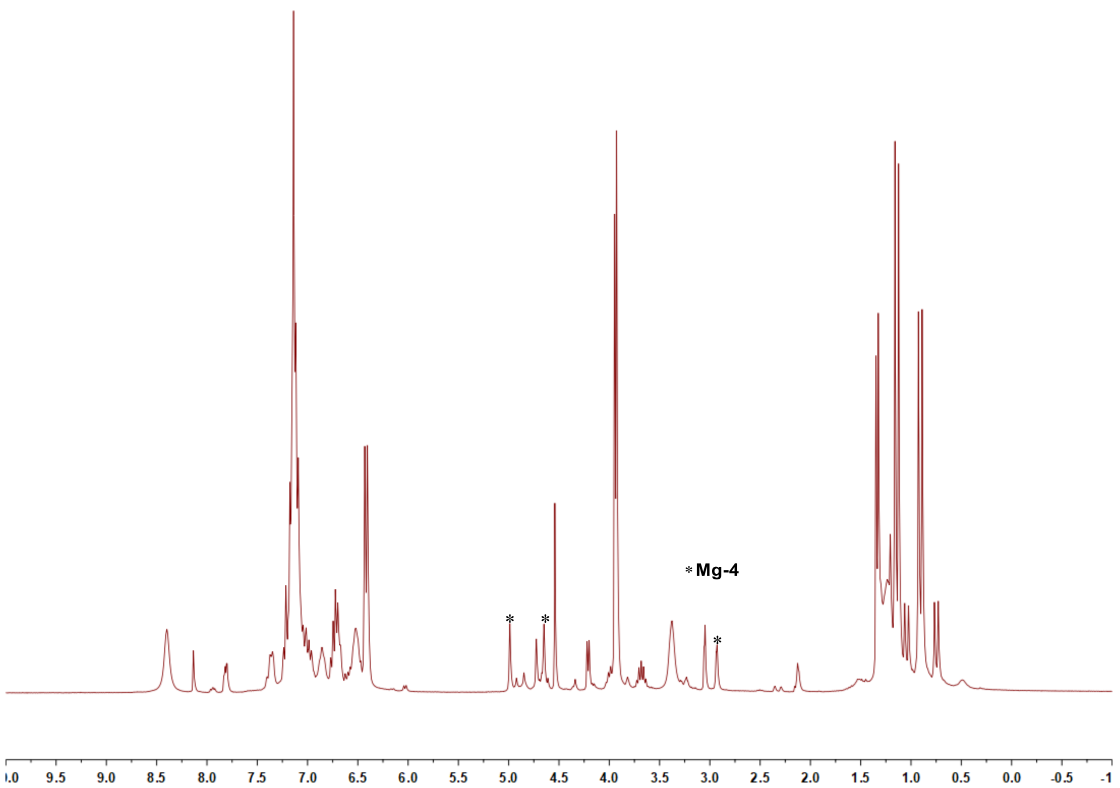

Figure S58. ^{1}H NMR spectrum of **Mg-2a** catalyzed hydrogenation of **1a** at 12 h

The experiment of **Mg-3** (0.01 mmol) catalyzed hydrogenation of **1a** (0.05 mmol) under 5 bar of H_2 in the presence of a catalytic amount of pyridine (0.015 mmol) was also performed. The decomposition of **Mg-3** to generate the free ligand (**L1**) was observed (Figure S59). The dearomatized complex **Mg-2a** was not observed, but a small amount of **Mg-4** could be observed. **Mg-4** gradually disappeared at 24 h, at this stage, the yield of **2a** did not increase obviously (Figure S60), suggesting that the formation of **Mg-4** is correlative to amine **2a** formation. These results reveal that **Mg-3** catalyzed hydrogenation of **1a** undergoes the intermediate **Mg-4**, and **Mg-3** shows lower activity than **Mg-2** in the catalytic hydrogenation of **1a**.

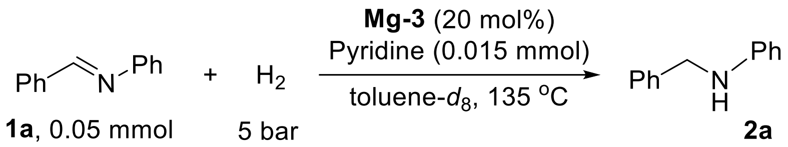

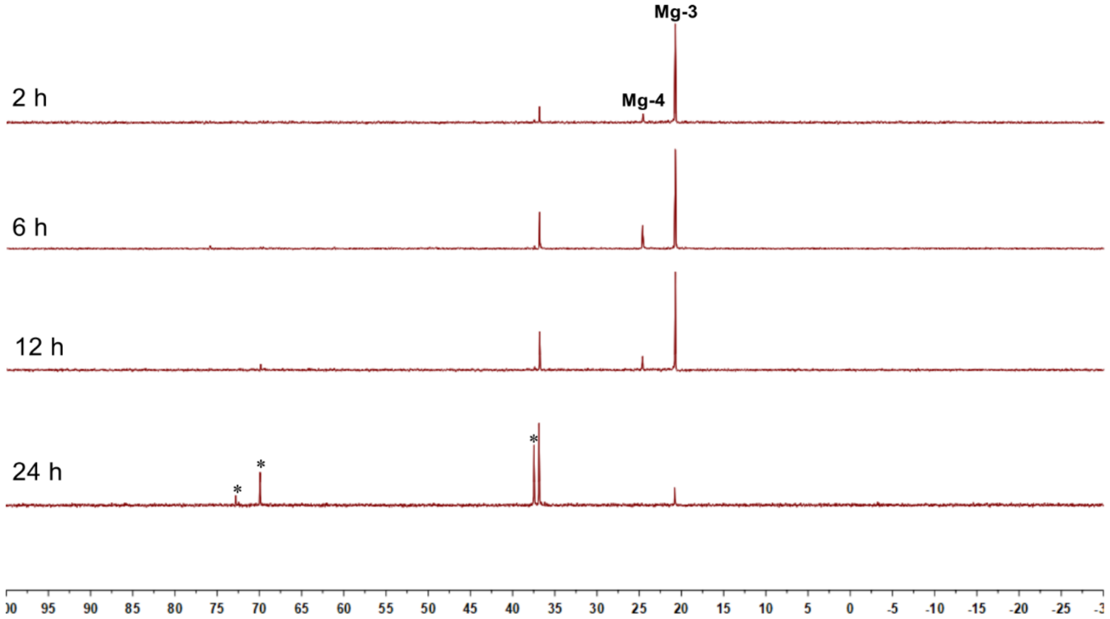

Figure S59. ^{31}P NMR spectra of **Mg-3** catalyzed hydrogenation of **1a** at different times

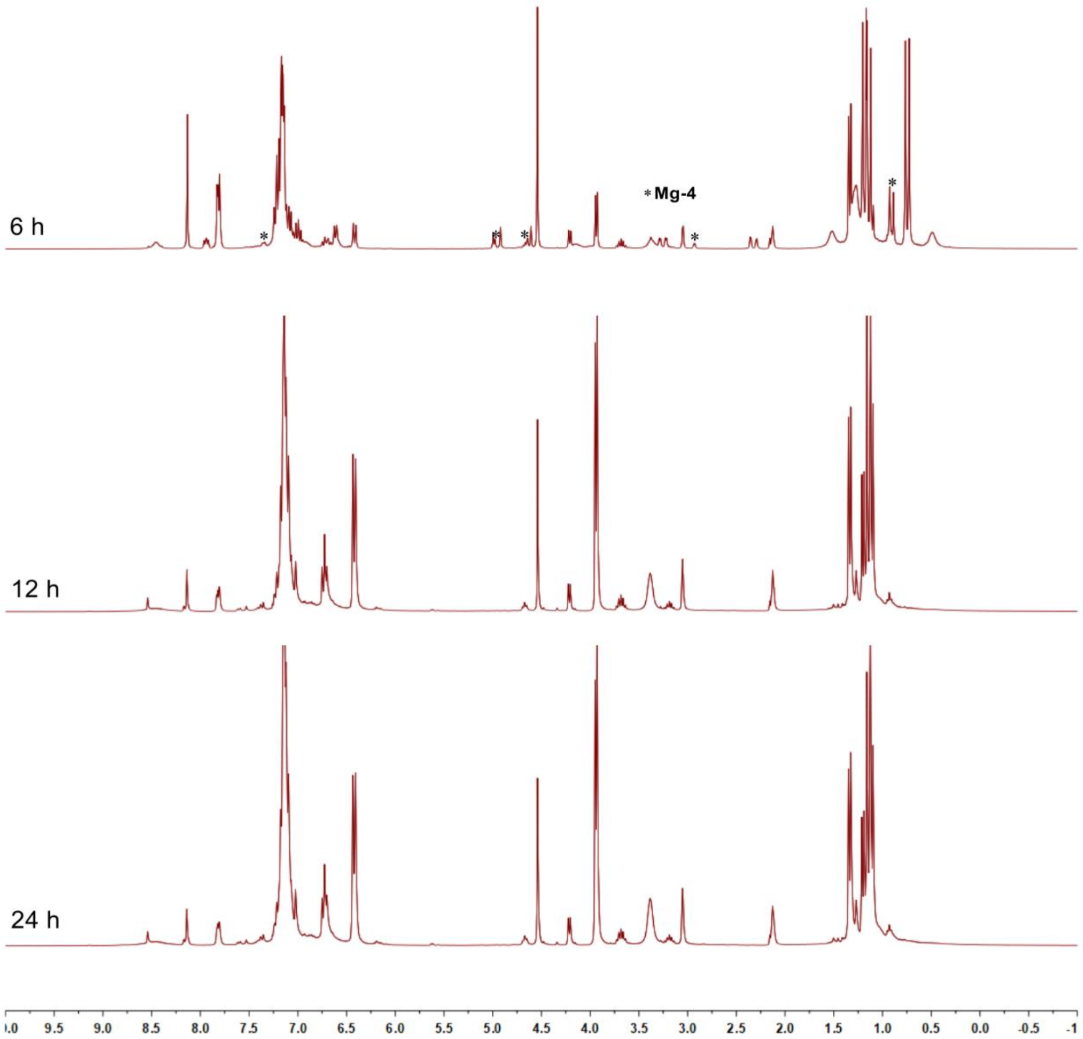

Figure S60. ^{1}H NMR spectra of **Mg-3** catalyzed hydrogenation of **1a** at different times

6 Computational details

DFT calculations were performed with Gaussian 16 (C.01 revision)[14] using Truhlar's M06-L functional,[15] the triple-ξ def2-TZVP basis set,[16] W06 density fitting,[17] and Grimme's D3(0) empirical dispersion correction.[18] Frequency calculations at this level of theory were run to confirm stationary points and transition states, as well as to obtain thermodynamic corrections. Single point energies of the M06-L optimized structures were computed with ORCA (4.2.1)[19] using the range-separated meta-GGA hybrid functional ωB97M-V of the Head-Gordon group[20] including dispersion correction,[21,22] together with the triple-ξ def2-TZVPP basis set[16] and the corresponding auxiliary basis sets, def2/J[17] and def2-TZVPP/C[23] for RIJCOSX density fitting. The functional and

basis set selections are based on recent benchmark studies.[24] Gibbs free energies were computed by adding the free energy correction term from the frequency calculation to the single point energy, according to

$$\mathbf{G^{o\omega B97M\text{-}V} = E^{\omega B97M\text{-}V} + corr^{\mathit{M06\text{-}L}}_{\mathit{freq}}}$$

where $\mathbf{E^{\omega B97M\text{-}V}}$ is the single point energy, and where $\mathbf{corr^{\mathit{M06\text{-}L}}_{\mathit{freq}}}$ is the thermal correction to the Gibbs free energy from the frequency calculation (at T = 298.15 K and P = 1 atm).

A mechanism for **Mg-2a** catalyzed hydrogenation of imine **1a** to generate amine **2a** via an MLC pathway was computed. In this study, intermediate **A** is standardized to 0.0 kcal/mol:

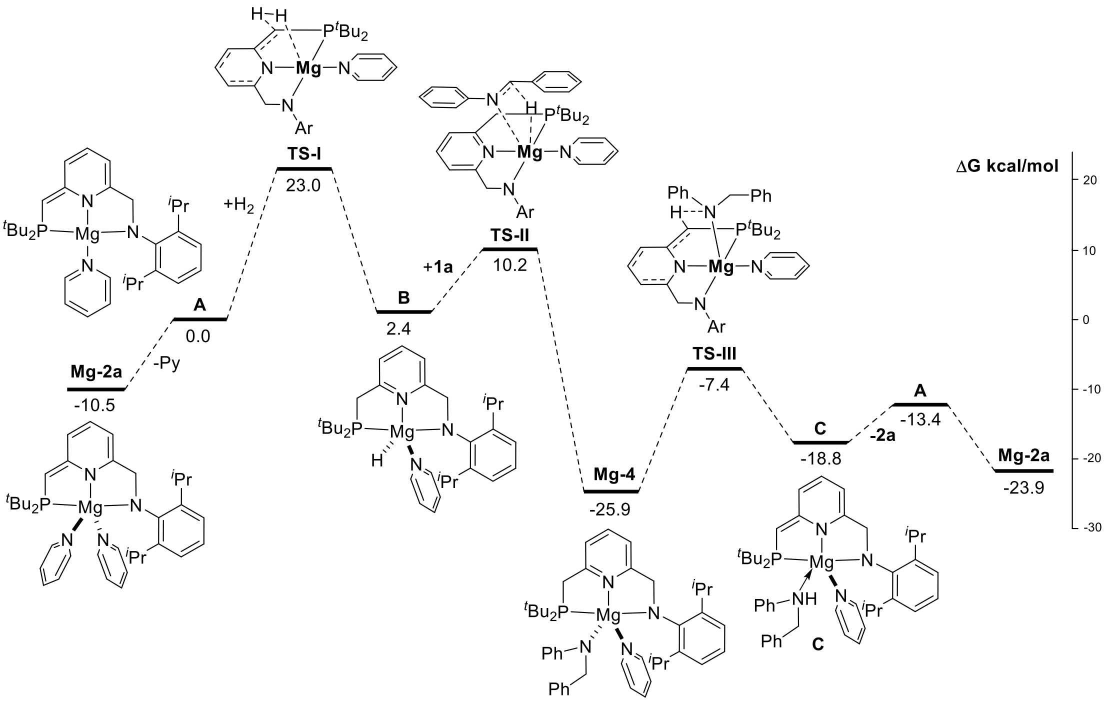

Figure S61. DFT studies for the hydrogenation of imines

In addition, the dissociation of one dioxane from **Mg-2b** to generate intermediate **A′**, which subsequently activated H_2 to generate intermediate **B′**, was calculated under the same conditions. The transition state **TS-IV** with a free energy barrier of 20.1 kcal/mol is required to overcome.

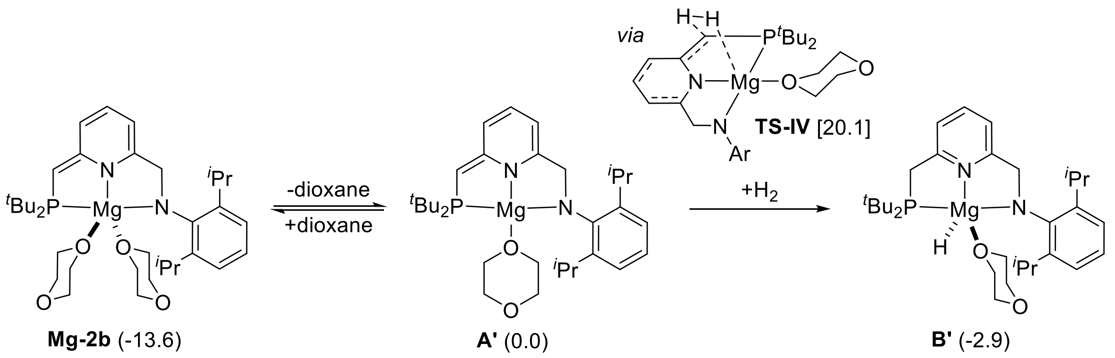

Table S16. Energies of computed structures in energy diagrams

Structure	$E^{\omega B97M\text{-}V}$	$G^{o\omega B97M\text{-}V}$	G
Energy Unit	Hartree	Hartree	kcal
**Mg-2a**	-2201.091423	-2200.360818	-1380747.259
**A**	-1952.774032	-1952.128154	-1224978.911
**TS-I**	-1953.916154	-1953.254527	-1225685.721
**B**	-1953.951823	-1953.287295	-1225706.283
**TS-II**	-2510.698289	-2509.842928	-1574950.215
**Mg-4**	-2510.764461	-2509.90045	-1574986.311
**TS-III**	-2510.729475	-2509.871026	-1574967.848
**C**	-2510.753469	-2509.889132	-1574979.209
**1a**	-556.7330534	-556.5680584	-349251.7296
**2a**	-557.9399847	-557.7524877	-349994.9702
**H_2**	-1.161490729	-1.165987729	-731.6683465
**Pyridine**	-248.2773742	-248.2247712	-155763.3956
**Mg-2b**	-2319.85147	-2319.054056	-1455228.391
**A′**	-2012.146364	-2011.466222	-1262214.111
**B′**	-2013.335466	-2012.636897	-1262948.721
**TS-IV**	-2013.295011	-2012.60011	-1262925.637
**dioxane**	-307.6603313	-307.5661613	-193000.6801

Cartesian Coordinates

Mg-2a

P 5.5977570000 3.6442730000 12.7954160000
Mg 7.3723600000 5.1126090000 14.0809830000
N 6.3113860000 6.5226000000 12.9968140000
N 7.5783830000 6.6951480000 15.3067880000
N 8.3727370000 3.6929170000 15.4304320000
N 9.2020940000 5.2523850000 12.8079810000
C 5.3834680000 4.8600770000 11.5533750000
H 4.8037050000 4.6469760000 10.6623930000

C 5.6481150000 6.2101070000 11.8341300000
C 5.2642040000 7.2905210000 10.9954780000
H 4.7689890000 7.0780080000 10.0568710000
C 5.4975500000 8.5822410000 11.3920210000
H 5.1835590000 9.3996550000 10.7525950000
C 6.1241260000 8.8614620000 12.6124340000
H 6.2991250000 9.8746050000 12.9471340000
C 6.5232360000 7.7827060000 13.3823200000
C 7.2793180000 7.9565910000 14.6809160000
H 6.6752930000 8.6095860000 15.3358150000
H 8.1599650000 8.5814800000 14.4393370000
C 3.9170940000 3.3940730000 13.6518610000
C 3.4886040000 4.8090160000 14.0460900000
H 3.2784120000 5.4254160000 13.1736950000
H 2.5874080000 4.7640400000 14.6628210000
H 4.2623850000 5.3199580000 14.6255080000
C 4.0685190000 2.5858950000 14.9367550000
H 4.7916760000 3.0561940000 15.6074970000
H 3.1158980000 2.5503830000 15.4724960000
H 4.3811960000 1.5567010000 14.7689020000
C 2.8521640000 2.7876740000 12.7500970000
H 3.0443310000 1.7388190000 12.5240910000
H 1.8727040000 2.8378210000 13.2337840000
H 2.7773420000 3.3319250000 11.8072920000
C 6.0990020000 2.0744790000 11.8786310000
C 7.5945570000 2.2447440000 11.6078100000
H 8.1618100000 2.2670110000 12.5412610000
H 7.9706890000 1.4114210000 11.0088600000
H 7.7946080000 3.1705820000 11.0660270000
C 5.9032620000 0.8230350000 12.7263280000
H 4.8518150000 0.5847340000 12.8799070000
H 6.3575660000 -0.0358880000 12.2261640000
H 6.3763380000 0.9131960000 13.7058580000
C 5.3825570000 1.9028360000 10.5426970000
H 5.5815850000 2.7418380000 9.8782630000
H 5.7409430000 0.9968150000 10.0469570000
H 4.3039660000 1.8084940000 10.6543230000
C 8.0685440000 6.7074860000 16.6047820000
C 9.3390560000 7.2414530000 16.9489250000
C 9.8005310000 7.1534440000 18.2580690000
H 10.7679350000 7.5773930000 18.5079100000
C 9.0673590000 6.5128160000 19.2404600000
H 9.4420340000 6.4497530000 20.2541250000
C 7.8451500000 5.9483180000 18.9077640000

H 7.2768470000 5.4410260000 19.6776790000
C 7.3283630000 6.0381780000 17.6213900000
C 10.2314570000 7.8501820000 15.8876330000
H 9.8118010000 7.5503390000 14.9224010000
C 10.2432920000 9.3745440000 15.9679470000
H 10.7030090000 9.7020750000 16.9026190000
H 10.8148180000 9.8139990000 15.1485610000
H 9.2370660000 9.7894690000 15.9387090000
C 11.6628180000 7.3226460000 15.9334590000
H 11.7035800000 6.2329790000 15.8877840000
H 12.2393470000 7.7070520000 15.0904950000
H 12.1817230000 7.6272570000 16.8431450000
C 5.9606120000 5.4686910000 17.3017100000
H 6.0296670000 4.9603650000 16.3274370000
C 5.4552660000 4.4435790000 18.3038530000
H 5.2446780000 4.8948360000 19.2746780000
H 4.5227150000 4.0010530000 17.9521850000
H 6.1716430000 3.6367120000 18.4658240000
C 4.9349670000 6.5875240000 17.1336870000
H 5.2140410000 7.2595400000 16.3247200000
H 3.9454530000 6.1842180000 16.9126220000
H 4.8581260000 7.1725010000 18.0515910000
C 9.4918140000 4.1612000000 15.9969350000
H 9.9265920000 5.0335780000 15.5231210000
C 10.0589300000 3.5993690000 17.1252590000
H 10.9493640000 4.0367520000 17.5544180000
C 9.4400140000 2.5011170000 17.6963130000
H 9.8463190000 2.0436500000 18.5891640000
C 8.2874940000 1.9995930000 17.1112680000
H 7.7725460000 1.1437460000 17.5246690000
C 7.7878450000 2.6272080000 15.9850960000
H 6.8776050000 2.2861100000 15.5018340000
C 10.2065780000 4.3682290000 12.8150900000
H 10.0199150000 3.4415460000 13.3451950000
C 11.4232770000 4.5961610000 12.2002560000
H 12.1983730000 3.8439030000 12.2441650000
C 11.6178320000 5.7962040000 11.5367400000
H 12.5593110000 6.0101130000 11.0474980000
C 10.5809910000 6.7129440000 11.5082800000
H 10.6793010000 7.6599630000 10.9966840000
C 9.3956980000 6.4038540000 12.1510720000
H 8.5642160000 7.0981940000 12.1474470000

A

P 5.4512010000 3.6556280000 12.8687810000
Mg 7.0247780000 5.1219890000 14.3060310000
N 6.3524380000 6.5206600000 12.9881370000
N 7.6229200000 6.7216690000 15.3056670000
N 8.3071600000 3.7391480000 15.3632270000
C 5.2439270000 4.8754280000 11.6411490000
H 4.6449400000 4.6750720000 10.7598880000
C 5.6274830000 6.2103240000 11.8621460000
C 5.2987020000 7.2783750000 10.9887500000
H 4.7478040000 7.0640070000 10.0823790000
C 5.6584400000 8.5620620000 11.3079060000
H 5.3907710000 9.3715150000 10.6383330000
C 6.3502900000 8.8444670000 12.4904930000
H 6.6211730000 9.8540290000 12.7665500000
C 6.6784900000 7.7792340000 13.3063600000
C 7.4241460000 7.9685840000 14.6093740000
H 6.8427800000 8.6836290000 15.2176620000
H 8.3442310000 8.5217900000 14.3629720000
C 3.7691740000 3.3072930000 13.6744680000
C 3.2511120000 4.6926140000 14.0630960000
H 3.0544000000 5.3109260000 13.1890700000
H 2.3277000000 4.5969630000 14.6393220000
H 3.9708540000 5.2329960000 14.6855680000
C 3.9379540000 2.4984110000 14.9573660000
H 4.6283850000 2.9958050000 15.6441950000
H 2.9802760000 2.4125290000 15.4775510000
H 4.3028490000 1.4874150000 14.7865450000
C 2.7668860000 2.6425060000 12.7421630000
H 3.0336180000 1.6104500000 12.5148080000
H 1.7750920000 2.6244980000 13.2017640000
H 2.6796870000 3.1870630000 11.8008880000
C 6.0924720000 2.1205350000 11.9794640000
C 7.5778270000 2.4064520000 11.7462990000
H 8.1284230000 2.4654870000 12.6876250000
H 8.0278700000 1.6091460000 11.1503030000
H 7.7161290000 3.3455530000 11.2080090000
C 5.9607040000 0.8534540000 12.8149940000
H 4.9237480000 0.5433700000 12.9379030000
H 6.4846740000 0.0310030000 12.3220490000
H 6.4010030000 0.9656990000 13.8071810000
C 5.4226150000 1.9122630000 10.6246600000
H 5.5616640000 2.7787320000 9.9803440000
H 5.8698330000 1.0507610000 10.1224960000
H 4.3543600000 1.7213950000 10.7104490000

C 8.0864550000 6.7185220000 16.6129630000
C 9.3513230000 7.2350250000 17.0009020000
C 9.7731060000 7.1230160000 18.3217640000
H 10.7341300000 7.5388020000 18.6047190000
C 9.0145490000 6.4688480000 19.2737700000
H 9.3634580000 6.3837770000 20.2946430000
C 7.7987560000 5.9190790000 18.8991250000
H 7.2068220000 5.4057470000 19.6460720000
C 7.3136540000 6.0407220000 17.6032350000
C 10.2811860000 7.8630040000 15.9847030000
H 9.9264790000 7.5502390000 14.9986940000
C 10.2453400000 9.3878960000 16.0583750000
H 10.6401510000 9.7287880000 17.0174020000
H 10.8517540000 9.8404660000 15.2724080000
H 9.2320060000 9.7759080000 15.9690490000
C 11.7210840000 7.3751920000 16.1123160000
H 11.7876960000 6.2869130000 16.0852920000
H 12.3307880000 7.7642020000 15.2963990000
H 12.1847740000 7.7055340000 17.0421240000
C 5.9251860000 5.5238850000 17.2698150000
H 5.9654550000 4.9682290000 16.3148620000
C 5.3579360000 4.5347000000 18.2756830000
H 5.1615520000 5.0077890000 19.2382070000
H 4.4075920000 4.1383440000 17.9172180000
H 6.0311450000 3.6946340000 18.4516940000
C 4.9525030000 6.6830640000 17.0640650000
H 5.2755600000 7.3331780000 16.2533570000
H 3.9485170000 6.3227520000 16.8354460000
H 4.8910450000 7.2813590000 17.9737740000
C 9.5036700000 4.1672040000 15.7851380000
H 9.8810980000 5.0620120000 15.3034350000
C 10.2106430000 3.5344230000 16.7904160000
H 11.1651790000 3.9301340000 17.1069140000
C 9.6550970000 2.4194060000 17.3929080000
H 10.1751570000 1.9125920000 18.1954020000
C 8.4193840000 1.9657540000 16.9586750000
H 7.9483230000 1.0999360000 17.4021750000
C 7.7833640000 2.6539500000 15.9441410000
H 6.8072270000 2.3485750000 15.5777990000

TS-I

P -2.7717710000 -0.3848880000 -0.3185930000
Mg -0.1812350000 0.1863420000 -0.8412030000
N -1.0164090000 2.0256060000 -0.3783500000

N 1.3547500000 1.0653170000 0.1313960000
N 0.8367200000 -1.6987640000 -1.0927180000
C -3.0134550000 1.1520760000 -1.2425130000
H -4.0199110000 1.3561360000 -1.6035030000
C -2.3018480000 2.2957960000 -0.7064610000
C -2.7723660000 3.6060440000 -0.5827810000
H -3.7909780000 3.8449780000 -0.8594300000
C -1.9215970000 4.5727560000 -0.0769200000
H -2.2814700000 5.5864510000 0.0520640000
C -0.6133040000 4.2573410000 0.2733620000
H 0.0592530000 5.0054870000 0.6712910000
C -0.1820720000 2.9518130000 0.0871000000
C 1.2284400000 2.4829140000 0.3397140000
H 1.5097800000 2.7861180000 1.3645270000
H 1.8740460000 3.1132680000 -0.2993320000
C -3.5135280000 -0.1453500000 1.4038990000
C -2.5071540000 0.7136990000 2.1776050000
H -2.4825550000 1.7423060000 1.8222830000
H -2.7911530000 0.7399520000 3.2325650000
H -1.4933110000 0.3168070000 2.1147560000
C -3.6243920000 -1.4920010000 2.1124590000
H -2.6793960000 -2.0407880000 2.0860570000
H -3.8791920000 -1.3354390000 3.1631560000
H -4.3988440000 -2.1274220000 1.6846110000
C -4.8552650000 0.5798030000 1.4137000000
H -5.6614140000 -0.0065060000 0.9805770000
H -5.1383560000 0.8090160000 2.4441390000
H -4.7962900000 1.5253650000 0.8738840000
C -3.7629840000 -1.6500110000 -1.3052280000
C -3.4027320000 -1.4572010000 -2.7798760000
H -2.3245830000 -1.4774600000 -2.9430910000
H -3.8452410000 -2.2690720000 -3.3612820000
H -3.7778830000 -0.5167700000 -3.1783160000
C -3.3159710000 -3.0589320000 -0.9112190000
H -3.4754940000 -3.2842970000 0.1410430000
H -3.8773450000 -3.7953100000 -1.4908630000
H -2.2594810000 -3.2094180000 -1.1328080000
C -5.2742560000 -1.5277290000 -1.1490120000
H -5.6287450000 -0.5184470000 -1.3585380000
H -5.7688210000 -2.1999790000 -1.8546800000
H -5.6119950000 -1.8064520000 -0.1517390000
C 2.5460760000 0.4454490000 0.5022070000
C 3.7804840000 0.6800010000 -0.1559540000
C 4.9113700000 -0.0457110000 0.2073760000

H 5.8513540000 0.1537790000 -0.2961770000
C 4.8585630000 -1.0326130000 1.1733520000
H 5.7460760000 -1.5939970000 1.4362150000
C 3.6519570000 -1.2909260000 1.8052480000
H 3.6129140000 -2.0613770000 2.5657750000
C 2.5055810000 -0.5660550000 1.5044560000
C 3.8823630000 1.6860760000 -1.2818770000
H 2.8594330000 1.9025830000 -1.6033660000
C 4.5349860000 2.9838300000 -0.8102810000
H 5.5747070000 2.8054310000 -0.5286940000
H 4.5316570000 3.7410890000 -1.5957620000
H 4.0311800000 3.3988370000 0.0615590000
C 4.6315030000 1.1468370000 -2.4963920000
H 4.2151170000 0.2023660000 -2.8483600000
H 4.5859890000 1.8564890000 -3.3228940000
H 5.6866450000 0.9750650000 -2.2807140000
C 1.2287930000 -0.7999300000 2.2870630000
H 0.3909410000 -0.7920280000 1.5746240000
C 1.1731660000 -2.1232090000 3.0334200000
H 1.8761250000 -2.1482010000 3.8676390000
H 0.1775770000 -2.2757660000 3.4527280000
H 1.4019220000 -2.9729270000 2.3897260000
C 0.9879240000 0.3518610000 3.2618320000
H 0.8697190000 1.2966740000 2.7354110000
H 0.0919060000 0.1836590000 3.8616160000
H 1.8312680000 0.4488210000 3.9472370000
C 2.0130880000 -1.6099380000 -1.7239780000
H 2.1919720000 -0.6855480000 -2.2636320000
C 2.9652780000 -2.6096350000 -1.6814340000
H 3.9102290000 -2.4763700000 -2.1887940000
C 2.6873280000 -3.7558650000 -0.9567870000
H 3.4151850000 -4.5541310000 -0.8912250000
C 1.4676410000 -3.8610740000 -0.3079480000
H 1.2127650000 -4.7361740000 0.2732910000
C 0.5775870000 -2.8072640000 -0.3951970000
H -0.3751800000 -2.8267450000 0.1264420000
H -1.0579090000 0.4940090000 -2.5493300000
H -1.9934600000 0.7809230000 -2.1832440000

B

P 6.3182850000 2.4445650000 11.8979670000
Mg 8.3442080000 4.1476980000 12.8168790000
N 7.3189600000 5.1778170000 11.1964140000
N 8.1555830000 6.0347520000 13.5333740000

N 8.6161160000 3.2579300000 14.8128200000
H 9.6563850000 3.3778380000 11.9179690000
C 6.6306380000 3.1022420000 10.1878690000
H 5.9005400000 2.7438350000 9.4596180000
C 6.7452880000 4.5963200000 10.1360170000
C 6.2725140000 5.3513220000 9.0760620000
H 5.8024990000 4.8677090000 8.2296390000
C 6.4018710000 6.7328910000 9.1328910000
H 6.0336200000 7.3459030000 8.3199480000
C 6.9845390000 7.3196920000 10.2398780000
H 7.0836790000 8.3945040000 10.3200120000
C 7.4334370000 6.5051940000 11.2738730000
C 8.0628890000 7.0464450000 12.5263870000
H 7.4647950000 7.9187380000 12.8474130000
H 9.0237380000 7.5010420000 12.2054680000
C 4.4704600000 2.7150950000 12.1610610000
C 4.2093610000 4.2020550000 11.9065030000
H 4.2537720000 4.4555860000 10.8484180000
H 3.2082370000 4.4567370000 12.2613440000
H 4.9197910000 4.8387770000 12.4378160000
C 4.1602440000 2.4494630000 13.6352370000
H 4.7770280000 3.0764700000 14.2811910000
H 3.1162940000 2.6975850000 13.8408010000
H 4.3112140000 1.4120670000 13.9263600000
C 3.5475250000 1.8925060000 11.2719460000
H 3.5949400000 0.8272020000 11.4924990000
H 2.5123910000 2.2067590000 11.4273570000
H 3.7678040000 2.0295260000 10.2125320000
C 6.6976140000 0.6132910000 11.6501700000
C 8.2205450000 0.4965380000 11.7583630000
H 8.5833490000 0.8204430000 12.7337880000
H 8.5137930000 -0.5458100000 11.6120730000
H 8.7454890000 1.1026020000 11.0208840000
C 6.0791520000 -0.2060720000 12.7795510000
H 4.9938510000 -0.2666350000 12.7104870000
H 6.4630090000 -1.2273890000 12.7357220000
H 6.3373200000 0.1937000000 13.7614430000
C 6.2566220000 0.0439030000 10.3042780000
H 6.7657380000 0.5267810000 9.4713730000
H 6.5147990000 -1.0167580000 10.2606790000
H 5.1847100000 0.1234280000 10.1367080000
C 8.3872320000 6.4450620000 14.8446940000
C 9.6019090000 7.0416410000 15.2652090000
C 9.7849190000 7.3733780000 16.6044970000

H 10.7136840000 7.8430090000 16.9107750000
C 8.8221300000 7.0941100000 17.5557100000
H 8.9839800000 7.3513900000 18.5947340000
C 7.6456990000 6.4755580000 17.1620270000
H 6.8949220000 6.2548940000 17.9107680000
C 7.4031550000 6.1575780000 15.8312070000
C 10.7235820000 7.2926160000 14.2806100000
H 10.5000900000 6.6988820000 13.3895200000
C 10.7982470000 8.7659930000 13.8855700000
H 11.0661000000 9.3780390000 14.7492770000
H 11.5526660000 8.9349880000 13.1156000000
H 9.8446200000 9.1364250000 13.5116010000
C 12.0814300000 6.8300380000 14.7988090000
H 12.0624590000 5.7878750000 15.1182560000
H 12.8414630000 6.9235930000 14.0228110000
H 12.4160670000 7.4250550000 15.6495100000
C 6.0750600000 5.5575940000 15.4148190000
H 6.2883590000 4.7768460000 14.6698920000
C 5.2929270000 4.9204760000 16.5527890000
H 4.9453170000 5.6647880000 17.2709770000
H 4.4043540000 4.4166830000 16.1702460000
H 5.8857450000 4.1888630000 17.1028920000
C 5.2059650000 6.6001760000 14.7142040000
H 5.6960470000 6.9889890000 13.8236100000
H 4.2449690000 6.1755620000 14.4158060000
H 5.0027030000 7.4392470000 15.3816440000
C 9.7747510000 3.6725950000 15.3339860000
H 10.4265640000 4.2141550000 14.6546270000
C 10.1334960000 3.4393380000 16.6481800000
H 11.0724870000 3.8180390000 17.0269010000
C 9.2603530000 2.7298680000 17.4551000000
H 9.5061010000 2.5316260000 18.4903940000
C 8.0646970000 2.2810660000 16.9178160000
H 7.3539270000 1.7243670000 17.5128100000
C 7.7813470000 2.5762230000 15.5963990000
H 6.8436470000 2.2717550000 15.1394010000
H 7.5990050000 2.6700970000 9.9121410000

TS-II

P 6.2031210000 2.1771230000 11.7793290000
Mg 8.2129330000 3.8847990000 12.6332920000
N 7.3669660000 4.7834300000 10.8702330000
N 8.2419100000 5.8238470000 13.1303110000
N 8.5270740000 3.1597350000 14.6387590000

H 9.6434700000 2.8626270000 12.0019600000
C 6.5521740000 2.6741610000 10.0255260000
H 5.7948670000 2.3073280000 9.3300390000
C 6.7596640000 4.1505300000 9.8555130000
C 6.3311940000 4.8372930000 8.7344040000
H 5.8312250000 4.3093790000 7.9328140000
C 6.5385180000 6.2099500000 8.6718910000
H 6.2043610000 6.7709930000 7.8084990000
C 7.1622570000 6.8508520000 9.7228010000
H 7.3373160000 7.9185250000 9.7086200000
C 7.5653920000 6.1061140000 10.8257690000
C 8.2345380000 6.7243650000 12.0157940000
H 7.7117990000 7.6708260000 12.2371750000
H 9.2318430000 7.0501170000 11.6526500000
C 4.3786400000 2.5709750000 12.0221030000
C 4.1981700000 4.0424980000 11.6401820000
H 4.2572070000 4.2024470000 10.5646410000
H 3.2120340000 4.3786370000 11.9672500000
H 4.9399750000 4.6848960000 12.1190460000
C 4.0659160000 2.4440450000 13.5140900000
H 4.7362870000 3.0666830000 14.1087040000
H 3.0467050000 2.7875800000 13.7048660000
H 4.1385290000 1.4227140000 13.8817870000
C 3.4114820000 1.7254150000 11.2042250000
H 3.4061050000 0.6807770000 11.5113190000
H 2.3949180000 2.1037070000 11.3354780000
H 3.6315280000 1.7647760000 10.1367130000
C 6.4852790000 0.3151790000 11.6939030000
C 7.9982890000 0.1376950000 11.8340840000
H 8.3586330000 0.5329120000 12.7846710000
H 8.2506990000 -0.9244340000 11.7874090000
H 8.5572200000 0.6464530000 11.0467600000
C 5.8209590000 -0.3763790000 12.8812660000
H 4.7347190000 -0.3862550000 12.8079320000
H 6.1511750000 -1.4162300000 12.9226280000
H 6.0954290000 0.0870430000 13.8298600000
C 6.0255010000 -0.3416700000 10.3952310000
H 6.5638900000 0.0419980000 9.5298560000
H 6.2269160000 -1.4141250000 10.4437760000
H 4.9603890000 -0.2203240000 10.2108890000
C 8.4446130000 6.3654260000 14.4010010000
C 9.6560220000 6.9869930000 14.7903780000
C 9.8059200000 7.4568360000 16.0917690000
H 10.7357520000 7.9398170000 16.3722080000

C 8.8137530000 7.2926020000 17.0390200000
H 8.9512400000 7.6548300000 18.0498640000
C 7.6397680000 6.6507960000 16.6787520000
H 6.8654390000 6.5200640000 17.4241530000
C 7.4298590000 6.1956890000 15.3824390000
C 10.8176630000 7.0983970000 13.8292170000
H 10.6213050000 6.4080080000 13.0045730000
C 10.9518720000 8.5143970000 13.2743950000
H 11.1755540000 9.2204880000 14.0770500000
H 11.7587620000 8.5796410000 12.5427920000
H 10.0357280000 8.8527060000 12.7910750000
C 12.1364180000 6.6550220000 14.4529170000
H 12.0467490000 5.6761240000 14.9262500000
H 12.9101300000 6.5741160000 13.6884750000
H 12.4911780000 7.3569250000 15.2096840000
C 6.0975600000 5.5821520000 15.0003810000
H 6.3002270000 4.7324670000 14.3313740000
C 5.2934740000 5.0545520000 16.1783650000
H 4.9540810000 5.8603650000 16.8305500000
H 4.3980640000 4.5381600000 15.8304310000
H 5.8696690000 4.3590870000 16.7898880000
C 5.2620230000 6.5799920000 14.2012940000
H 5.7731020000 6.8775330000 13.2865550000
H 4.2918550000 6.1583270000 13.9297300000
H 5.0771270000 7.4794350000 14.7905820000
C 9.6758310000 3.6455180000 15.1242090000
H 10.2681050000 4.2436510000 14.4351260000
C 10.0918940000 3.4158830000 16.4227950000
H 11.0237750000 3.8391340000 16.7698970000
C 9.2917460000 2.6462120000 17.2478010000
H 9.5861840000 2.4499930000 18.2706960000
C 8.1042900000 2.1323470000 16.7481170000
H 7.4495110000 1.5291100000 17.3613570000
C 7.7605870000 2.4190580000 15.4411480000
H 6.8301410000 2.0604460000 15.0089850000
H 7.4838100000 2.1538760000 9.7751190000
N 11.6482330000 4.2545050000 11.7964200000
C 11.2976330000 2.9900660000 11.8858930000
C 11.2861990000 4.9569550000 10.6770700000
C 11.7778660000 6.2698450000 10.5369750000
H 12.4544630000 6.6334370000 11.3010230000
C 11.4182010000 7.0662100000 9.4703240000
H 11.8157660000 8.0715850000 9.3959890000
C 10.5493340000 6.5871920000 8.4921690000

H 10.2667030000 7.2113590000 7.6541940000
C 10.0570240000 5.2960080000 8.6053840000
H 9.3802890000 4.9106060000 7.8505650000
C 10.4088170000 4.4889610000 9.6763580000
H 9.9981550000 3.4895050000 9.7461130000
H 11.1828070000 2.3667450000 10.9880840000
C 11.7423800000 2.2310600000 13.0770330000
C 11.2953340000 0.9310690000 13.3043930000
C 12.6412500000 2.7943800000 13.9816670000
C 11.7004140000 0.2245020000 14.4242140000
C 13.0573820000 2.0847670000 15.0968270000
C 12.5801590000 0.8026050000 15.3305070000
H 10.6160780000 0.4812030000 12.5906540000
H 13.0018830000 3.7946180000 13.7797490000
H 11.3351640000 -0.7816200000 14.5896190000
H 13.7609170000 2.5341320000 15.7875170000
H 12.9021690000 0.2503760000 16.2043490000

Mg-4

P 7.1712350000 1.7941780000 12.7845490000
Mg 8.4298900000 4.0346140000 13.9612240000
N 6.3925480000 4.6425160000 13.3068520000
N 7.9238300000 5.3825760000 15.3793090000
N 9.8875740000 2.7176670000 14.9835500000
C 5.8929900000 2.7420680000 11.8525940000
H 4.9891660000 2.1501400000 11.6876800000
C 5.5334800000 4.0748160000 12.4461510000
C 4.3515110000 4.6982800000 12.0841420000
H 3.6702270000 4.2044450000 11.4037200000
C 4.0709100000 5.9572250000 12.5917080000
H 3.1576870000 6.4677690000 12.3139500000
C 4.9639950000 6.5410690000 13.4657100000
H 4.7798930000 7.5180950000 13.8923690000
C 6.1106210000 5.8450200000 13.8267400000
C 7.0904280000 6.4066240000 14.8095980000
H 6.5158430000 6.9507540000 15.5767990000
H 7.6246140000 7.2077560000 14.2567970000
C 6.1681380000 0.8309310000 14.0559260000
C 5.2737080000 1.8548660000 14.7583750000
H 4.4661110000 2.2105660000 14.1180280000
H 4.8154830000 1.3911830000 15.6347870000
H 5.8329780000 2.7266570000 15.1035270000
C 7.1450780000 0.2724390000 15.0933080000
H 7.7160060000 1.0718280000 15.5696590000

H 6.5910950000 -0.2471380000 15.8781830000
H 7.8554550000 -0.4362640000 14.6699260000
C 5.2912120000 -0.2852730000 13.5049800000
H 5.8713430000 -1.1120570000 13.0984740000
H 4.6734470000 -0.6920790000 14.3091380000
H 4.6124660000 0.0687310000 12.7282110000
C 7.8160560000 0.6490440000 11.4262230000
C 8.7796110000 1.5020500000 10.5939780000
H 9.5394210000 2.0012220000 11.1979330000
H 9.2871950000 0.8701950000 9.8620160000
H 8.2581520000 2.2800310000 10.0359160000
C 8.5879280000 -0.5171820000 12.0398910000
H 7.9349090000 -1.2142660000 12.5632170000
H 9.0857380000 -1.0770890000 11.2456410000
H 9.3569130000 -0.1918410000 12.7391330000
C 6.7452720000 0.0926280000 10.4891530000
H 6.1940300000 0.8791080000 9.9760750000
H 7.2284330000 -0.5089630000 9.7157640000
H 6.0284000000 -0.5491300000 10.9946620000
C 8.4453730000 5.6845400000 16.6373580000
C 9.3385370000 6.7603220000 16.8670430000
C 9.8077230000 7.0049240000 18.1548330000
H 10.4824800000 7.8382030000 18.3191530000
C 9.4560600000 6.1991320000 19.2212920000
H 9.8363770000 6.4031210000 20.2139280000
C 8.6148380000 5.1195950000 18.9999250000
H 8.3423510000 4.4823570000 19.8328590000
C 8.0989360000 4.8539220000 17.7379760000
C 9.8270870000 7.6265010000 15.7261300000
H 9.5713400000 7.1031430000 14.8007330000
C 9.1482270000 8.9939370000 15.7087590000
H 9.4329440000 9.5753260000 16.5880570000
H 9.4394310000 9.5654360000 14.8254560000
H 8.0623430000 8.9129540000 15.7116510000
C 11.3419390000 7.8054340000 15.7384200000
H 11.8615570000 6.8487540000 15.7970260000
H 11.6761260000 8.3122510000 14.8317350000
H 11.6723240000 8.4101550000 16.5839870000
C 7.1393320000 3.7026300000 17.5249030000
H 7.3662920000 3.2812450000 16.5368740000
C 7.2673300000 2.5761440000 18.5370240000
H 6.9494850000 2.8806380000 19.5352640000
H 6.6369540000 1.7343820000 18.2461030000
H 8.2930130000 2.2120560000 18.6162510000

C 5.7033790000 4.2181420000 17.4725270000
H 5.5753330000 4.9347880000 16.6620740000
H 4.9891950000 3.4063220000 17.3247600000
H 5.4454650000 4.7183420000 18.4073440000
C 10.3095400000 2.8077480000 16.2501400000
H 9.8564200000 3.5842850000 16.8544710000
C 11.2751360000 1.9684210000 16.7786600000
H 11.5730930000 2.0847790000 17.8114960000
C 11.8389050000 0.9990400000 15.9668850000
H 12.5965050000 0.3281650000 16.3515380000
C 11.4297860000 0.9168160000 14.6466380000
H 11.8624690000 0.2010020000 13.9615730000
C 10.4620580000 1.7971260000 14.2016220000
H 10.1315950000 1.7908240000 13.1696090000
H 6.3149150000 2.9143740000 10.8574470000
H 8.0321880000 4.2986780000 10.8658560000
N 9.7741480000 4.8463070000 12.6498210000
C 11.0069480000 5.1749670000 13.3277220000
H 7.2577810000 8.2985480000 9.5135720000
C 9.2127070000 5.7919290000 11.8302650000
C 9.4835750000 7.1805380000 11.8317410000
H 10.2233460000 7.5898410000 12.5084730000
C 8.7870020000 8.0525600000 11.0107580000
H 9.0188880000 9.1106410000 11.0596420000
C 7.7951650000 7.6069010000 10.1476650000
C 7.5261960000 6.2420470000 10.1095070000
H 6.7740100000 5.8558660000 9.4293400000
C 8.2232900000 5.3660700000 10.9142200000
H 11.4061050000 6.1495650000 13.0210650000
H 10.8670630000 5.2716890000 14.4183590000
C 12.1023740000 4.1613350000 13.1042260000
C 13.0805320000 3.9537510000 14.0732630000
C 12.1666530000 3.4187470000 11.9280400000
C 14.0874980000 3.0203550000 13.8830260000
C 13.1713290000 2.4827320000 11.7333280000
C 14.1336140000 2.2750690000 12.7126060000
H 13.0373100000 4.5243000000 14.9960890000
H 11.4022830000 3.5843580000 11.1775770000
H 14.8344590000 2.8672590000 14.6527320000
H 13.2077090000 1.9152000000 10.8108740000
H 14.9171700000 1.5430200000 12.5625550000

TS-III

P 2.9859160000 -0.8507560000 0.3505470000

Mg 0.2532090000 -0.2028320000 -0.1927420000
N 1.3780410000 0.5414750000 -1.7851790000
N -0.9725780000 -0.6082750000 -1.7264690000
N -0.7352210000 -1.2668400000 1.3973480000
C 3.1584910000 0.8502770000 -0.2630660000
C 2.5879650000 1.1076830000 -1.5736030000
C 3.1420790000 1.9141110000 -2.5715150000
H 4.0980600000 2.3942500000 -2.4099410000
C 2.4552180000 2.0713810000 -3.7620340000
H 2.8799100000 2.6851020000 -4.5472420000
C 1.2298450000 1.4483010000 -3.9609320000
H 0.6814570000 1.5617220000 -4.8865680000
C 0.7087060000 0.6868880000 -2.9239010000
C -0.6404190000 0.0183010000 -2.9777080000
H -0.6240250000 -0.7034930000 -3.8156170000
H -1.3540670000 0.7918300000 -3.3214090000
C 3.6938130000 -2.1479310000 -0.8630330000
C 3.0330590000 -1.9362040000 -2.2264000000
H 3.3553870000 -1.0176470000 -2.7128010000
H 3.3093530000 -2.7683490000 -2.8789160000
H 1.9440670000 -1.9210140000 -2.1577170000
C 3.2407750000 -3.5293580000 -0.3807320000
H 2.1618920000 -3.5487860000 -0.2103880000
H 3.4618580000 -4.2739060000 -1.1497080000
H 3.7289140000 -3.8565810000 0.5327580000
C 5.2023800000 -2.1165080000 -1.0636540000
H 5.7548710000 -2.4186930000 -0.1755140000
H 5.4812940000 -2.8083060000 -1.8629490000
H 5.5461740000 -1.1243040000 -1.3603450000
C 4.1272950000 -0.8086060000 1.8743630000
C 3.4230680000 -0.0047200000 2.9639790000
H 2.4920820000 -0.4768030000 3.2809910000
H 4.0706900000 0.0596700000 3.8417130000
H 3.2050350000 1.0126720000 2.6420860000
C 4.3545210000 -2.2156140000 2.4214940000
H 5.0098270000 -2.8113290000 1.7896610000
H 4.8321130000 -2.1463590000 3.4015240000
H 3.4196510000 -2.7633760000 2.5517400000
C 5.4819920000 -0.1416800000 1.6319330000
H 5.3789460000 0.9212450000 1.4239780000
H 6.0851210000 -0.2289910000 2.5391820000
H 6.0485280000 -0.5890170000 0.8212030000
C -2.1450020000 -1.3652990000 -1.6902400000
C -3.4324780000 -0.7838830000 -1.8102060000

C -4.5680030000 -1.5823330000 -1.7216710000
H -5.5453070000 -1.1225070000 -1.8277240000
C -4.4787410000 -2.9389310000 -1.4712020000
H -5.3718370000 -3.5465490000 -1.4018430000
C -3.2280380000 -3.5072510000 -1.2950570000
H -3.1583320000 -4.5673020000 -1.0833940000
C -2.0642640000 -2.7545340000 -1.4021410000
C -3.6037300000 0.7134360000 -1.9465170000
H -2.6206480000 1.1576170000 -1.7767640000
C -4.0899020000 1.1154840000 -3.3348580000
H -5.0890590000 0.7176770000 -3.5232640000
H -4.1438170000 2.2005010000 -3.4383840000
H -3.4364490000 0.7338650000 -4.1187460000
C -4.5326100000 1.2788320000 -0.8763560000
H -4.2306080000 0.9648330000 0.1241610000
H -4.5290030000 2.3704030000 -0.8962380000
H -5.5639430000 0.9543210000 -1.0210950000
C -0.7158650000 -3.4309590000 -1.2653000000
H -0.0250700000 -2.7111670000 -0.8062640000
C -0.7304180000 -4.6672830000 -0.3795170000
H -1.2738270000 -5.4941790000 -0.8387330000
H 0.2855990000 -5.0211930000 -0.1999570000
H -1.1942690000 -4.4680170000 0.5875440000
C -0.1463510000 -3.7604250000 -2.6422450000
H -0.0607810000 -2.8637010000 -3.2551590000
H 0.8438310000 -4.2139630000 -2.5660530000
H -0.7975240000 -4.4619460000 -3.1665480000
C -2.0573960000 -1.0761280000 1.4821510000
H -2.4933760000 -0.4149310000 0.7425010000
C -2.8412320000 -1.6816670000 2.4468730000
H -3.9056340000 -1.4937090000 2.4652670000
C -2.2309160000 -2.5157540000 3.3669790000
H -2.8135420000 -3.0044280000 4.1373670000
C -0.8616200000 -2.7189790000 3.2861670000
H -0.3465670000 -3.3661100000 3.9821040000
C -0.1544010000 -2.0803860000 2.2852400000
H 0.9171870000 -2.2129030000 2.1656140000
H 4.1314240000 1.3143350000 -0.1250150000
H 1.9934220000 3.9367190000 1.3533870000
N 0.7187090000 1.6433950000 0.7722330000
C 0.5631470000 1.6560340000 2.2204510000
H 0.2822200000 6.2629290000 -1.8064040000
C 0.5568190000 2.9047730000 0.1352940000
C -0.2955340000 3.0357770000 -0.9631700000

H -0.8867480000 2.1747570000 -1.2612620000
C -0.3941870000 4.2282160000 -1.6574760000
H -1.0639720000 4.2966630000 -2.5065720000
C 0.3578140000 5.3281190000 -1.2669930000
C 1.2062040000 5.2153710000 -0.1753670000
H 1.8030510000 6.0640750000 0.1350910000
C 1.3091520000 4.0186050000 0.5164360000
H 1.0043690000 0.7313560000 2.5954600000
H 1.1436020000 2.4614110000 2.6953530000
C -0.8466580000 1.7096790000 2.7567390000
C -1.9371590000 2.1638860000 2.0215700000
C -1.0697780000 1.2674390000 4.0614570000
C -3.2086610000 2.1868590000 2.5816730000
C -2.3368160000 1.2783880000 4.6181120000
C -3.4155540000 1.7435590000 3.8776100000
H -1.7978800000 2.5002820000 1.0024250000
H -0.2297200000 0.9000540000 4.6428910000
H -4.0413280000 2.5559570000 1.9964290000
H -2.4847960000 0.9218490000 5.6300030000
H -4.4079530000 1.7606510000 4.3094390000
H 1.9910630000 1.3648100000 0.4846720000

C
P 7.1256260000 1.7666520000 12.8490730000
Mg 8.2398640000 3.9323650000 14.1011980000
N 6.4391780000 4.6525450000 13.3254690000
N 7.9485210000 5.4351200000 15.3873640000
N 9.9248570000 2.8977110000 15.1283190000
C 6.0302040000 2.7522330000 11.9155820000
C 5.7012960000 4.0560230000 12.3330680000
C 4.6327570000 4.7967500000 11.7675560000
H 4.0414520000 4.3435220000 10.9822250000
C 4.3543530000 6.0606300000 12.2206980000
H 3.5304030000 6.6158940000 11.7866530000
C 5.1131520000 6.6314620000 13.2444310000
H 4.9033130000 7.6197500000 13.6298110000
C 6.1490840000 5.8805860000 13.7696430000
C 7.0136680000 6.4161910000 14.8853330000
H 6.3394770000 6.7726470000 15.6815760000
H 7.4773680000 7.3446930000 14.4987540000
C 6.0880860000 0.7087170000 14.0477350000
C 5.1783100000 1.7195440000 14.7461660000
H 4.4451060000 2.1420630000 14.0608950000
H 4.6440400000 1.2331160000 15.5665460000

H 5.7411900000 2.5555970000 15.1669670000
C 6.9859800000 0.0747700000 15.1060510000
H 7.5637600000 0.8388890000 15.6334530000
H 6.3825080000 -0.4444360000 15.8557430000
H 7.6883170000 -0.6514370000 14.6986590000
C 5.2076500000 -0.3319470000 13.3708260000
H 5.7746320000 -1.1517880000 12.9318290000
H 4.5194740000 -0.7719580000 14.0980110000
H 4.6005430000 0.1211940000 12.5854260000
C 7.9371120000 0.6150180000 11.5750210000
C 9.0768880000 1.4321480000 10.9704100000
H 9.8302250000 1.6752070000 11.7231590000
H 9.5783220000 0.8729580000 10.1755090000
H 8.7024240000 2.3618980000 10.5340740000
C 8.5356120000 -0.6515070000 12.1807690000
H 7.7790040000 -1.3251060000 12.5774430000
H 9.0785050000 -1.1997890000 11.4067250000
H 9.2496630000 -0.4457170000 12.9789520000
C 6.9826540000 0.2185190000 10.4516710000
H 6.6174360000 1.0931690000 9.9170170000
H 7.5079450000 -0.4147610000 9.7318940000
H 6.1216380000 -0.3413860000 10.8117420000
C 8.4364410000 5.6733630000 16.6703410000
C 9.3122210000 6.7489750000 16.9637410000
C 9.8114390000 6.9160810000 18.2515710000
H 10.4735800000 7.7510990000 18.4576770000
C 9.5052600000 6.0243790000 19.2627380000
H 9.9023660000 6.1637840000 20.2600010000
C 8.6878630000 4.9415790000 18.9777630000
H 8.4572850000 4.2364550000 19.7671070000
C 8.1452990000 4.7507140000 17.7125840000
C 9.7901820000 7.6614870000 15.8592040000
H 9.4586940000 7.1939250000 14.9302430000
C 9.1791290000 9.0554840000 15.9408870000
H 9.5111430000 9.5734760000 16.8429920000
H 9.4721270000 9.6673010000 15.0850500000
H 8.0911200000 9.0154340000 15.9683130000
C 11.3123640000 7.7489480000 15.8041820000
H 11.7721990000 6.7586220000 15.7785310000
H 11.6404980000 8.2928330000 14.9158820000
H 11.7229110000 8.2698320000 16.6702000000
C 7.2114730000 3.5885270000 17.4539790000
H 7.4071680000 3.2304460000 16.4337820000
C 7.4133780000 2.3990970000 18.3780050000

H 7.1386500000 2.6254580000 19.4091210000
H 6.7870240000 1.5649770000 18.0598970000
H 8.4509870000 2.0591150000 18.3794100000
C 5.7620460000 4.0661520000 17.4860330000
H 5.5788890000 4.8129210000 16.7144440000
H 5.0652650000 3.2419800000 17.3304000000
H 5.5341040000 4.5169970000 18.4531810000
C 10.6278710000 3.4847690000 16.1075240000
H 10.4309940000 4.5343760000 16.2810580000
C 11.5591510000 2.8064540000 16.8751330000
H 12.0742670000 3.3309110000 17.6682930000
C 11.8077030000 1.4728990000 16.6038520000
H 12.5304620000 0.9154150000 17.1859810000
C 11.1184300000 0.8674190000 15.5654120000
H 11.2858220000 -0.1675090000 15.3001950000
C 10.1872440000 1.6115880000 14.8674470000
H 9.6022390000 1.1741370000 14.0680520000
H 5.3566890000 2.2949270000 11.1991680000
H 7.8514140000 4.4858870000 10.4654230000
N 9.6178370000 4.7571700000 12.4071500000
C 10.9703650000 5.0906760000 12.9021160000
H 6.8658690000 8.6349090000 10.0734010000
C 8.9209540000 5.8245400000 11.7552500000
C 9.0936050000 7.1467290000 12.1482800000
H 9.7884410000 7.3995610000 12.9374360000
C 8.3534940000 8.1509050000 11.5399470000
H 8.4891330000 9.1750740000 11.8634480000
C 7.4471290000 7.8501570000 10.5382880000
C 7.2845670000 6.5296050000 10.1421820000
H 6.5726530000 6.2758680000 9.3678700000
C 8.0176620000 5.5224530000 10.7414150000
H 11.3399040000 5.9658420000 12.3546520000
H 10.8970470000 5.3989330000 13.9500600000
C 11.9605400000 3.9728490000 12.7595360000
C 12.8858080000 3.7297930000 13.7704950000
C 12.0286040000 3.2008000000 11.6028620000
C 13.8251870000 2.7193040000 13.6495490000
C 12.9604530000 2.1814930000 11.4811250000
C 13.8575260000 1.9319700000 12.5079990000
H 12.8628070000 4.3416720000 14.6644610000
H 11.3526530000 3.3962340000 10.7770210000
H 14.5293730000 2.5424730000 14.4528030000
H 12.9875520000 1.5863540000 10.5773280000
H 14.5841230000 1.1355940000 12.4146830000

H 9.6664030000 3.9656610000 11.7727700000

1a

C -0.8898820000 0.0961360000 -0.6206340000
H -1.8442770000 0.4910970000 -0.2397720000
N 0.0036010000 -0.3682340000 0.1609230000
C -0.2413220000 -0.4637180000 1.5295070000
C -1.4904490000 -0.8012470000 2.0607840000
C 0.8299820000 -0.2628210000 2.4033350000
C -1.6627590000 -0.9075340000 3.4300350000
C 0.6455570000 -0.3512670000 3.7706840000
C -0.6005460000 -0.6741760000 4.2915710000
H -2.3135790000 -1.0129540000 1.3894540000
H 1.7982880000 -0.0248070000 1.9826930000
H -2.6314120000 -1.1834340000 3.8274870000
H 1.4818630000 -0.1786320000 4.4360370000
H -0.7387020000 -0.7598200000 5.3612710000
C -0.7198860000 0.1610980000 -2.0635530000
C 0.4434150000 -0.3031150000 -2.6851100000
C -1.7399400000 0.6971240000 -2.8506810000
C 0.5753880000 -0.2291980000 -4.0572220000
C -1.6064440000 0.7688210000 -4.2269060000
C -0.4482660000 0.3057410000 -4.8324650000
H 1.2282950000 -0.7159230000 -2.0646000000
H -2.6421490000 1.0579160000 -2.3696280000
H 1.4787330000 -0.5896080000 -4.5324610000
H -2.4041840000 1.1859220000 -4.8275490000
H -0.3404590000 0.3606340000 -5.9081210000

2a

C -1.2880110000 0.2077380000 -0.6549160000
H -1.4620430000 1.2324430000 -0.3062640000
N -0.2279220000 -0.3646010000 0.1419550000
C -0.3462850000 -0.5091900000 1.5118550000
C -1.3351550000 0.1510880000 2.2478790000
C 0.5715750000 -1.3121840000 2.2018270000
C -1.3833700000 0.0209120000 3.6275670000
C 0.5068800000 -1.4380760000 3.5746570000
C -0.4695210000 -0.7697290000 4.3040240000
H -2.0680270000 0.7659720000 1.7435550000
H 1.3406460000 -1.8318280000 1.6414720000
H -2.1565120000 0.5438000000 4.1766650000
H 1.2281210000 -2.0660080000 4.0826280000
H -0.5173460000 -0.8697760000 5.3795940000

C -0.9281460000 0.2324770000 -2.1110710000
C 0.3381240000 0.6412990000 -2.5249130000
C -1.8608790000 -0.1304940000 -3.0765420000
C 0.6600630000 0.6858340000 -3.8715810000
C -1.5425790000 -0.0810940000 -4.4252440000
C -0.2800900000 0.3263290000 -4.8265430000
H 1.0699420000 0.9261960000 -1.7783320000
H -2.8459390000 -0.4579840000 -2.7643690000
H 1.6478790000 1.0060740000 -4.1775810000
H -2.2806210000 -0.3686030000 -5.1630290000
H -0.0281000000 0.3611530000 -5.8784830000
H 0.2744490000 -1.1076520000 -0.3163370000
H -2.2432910000 -0.3196020000 -0.5176800000

H_2
H 1.2213590000 0.0262130000 0.0242980000
H 1.9654290000 0.0262130000 0.0242980000

Pyridine
C -0.6935160000 1.1330160000 0.0455610000
C -1.3576630000 -0.0836060000 0.0008960000
C -0.6044540000 -1.2455250000 -0.0213050000
C 0.7762350000 -1.1402910000 0.0019600000
C 1.3413070000 0.1253540000 0.0463530000
N 0.6317540000 1.2509010000 0.0682830000
H -1.2554740000 2.0623430000 0.0640020000
H -2.4390620000 -0.1162220000 -0.0159320000
H -1.0846760000 -2.2153460000 -0.0561480000
H 1.4057550000 -2.0201950000 -0.0140090000
H 2.4210680000 0.2416470000 0.0654350000

Mg-2b
P 3.5824720000 9.2591650000 13.3416880000
Mg 6.0341550000 9.8906850000 12.6067820000
O 6.4566500000 8.4943000000 11.0541120000
O 7.1027240000 8.5562960000 8.3118500000
N 4.8562360000 11.0321950000 11.3483900000
N 7.2945740000 11.4224910000 12.3692650000
C 2.8552810000 9.8165890000 11.8496010000
H 1.7995740000 9.6754620000 11.6452640000
C 3.5437140000 10.7515930000 11.0509500000
C 2.9747990000 11.4275270000 9.9407720000
H 1.9511780000 11.2137350000 9.6615890000
C 3.7116460000 12.3599490000 9.2535940000

H 3.2604850000 12.8822050000 8.4176010000
C 5.0263140000 12.6604390000 9.6262980000
H 5.6065150000 13.4158440000 9.1147830000
C 5.5589080000 11.9605750000 10.6953280000
C 6.9714240000 12.1926540000 11.1886760000
H 7.0843900000 13.2759770000 11.3610180000
H 7.6433540000 12.0075830000 10.3245910000
C 2.9940170000 7.4871950000 13.5992800000
C 3.7419730000 6.6399850000 12.5697060000
H 4.8238780000 6.7773280000 12.6317000000
H 3.5282270000 5.5808470000 12.7328390000
H 3.4274320000 6.8904340000 11.5580350000
C 1.5002190000 7.2899770000 13.3556470000
H 1.2125880000 7.6389060000 12.3645300000
H 1.2580740000 6.2252700000 13.4087730000
H 0.8800810000 7.7991820000 14.0882830000
C 3.3655810000 6.9938420000 14.9938510000
H 2.7727330000 7.4699710000 15.7730700000
H 3.1883160000 5.9182590000 15.0677840000
H 4.4197320000 7.1707260000 15.2194690000
C 2.9581140000 10.3424200000 14.7807460000
C 1.5081180000 10.1114210000 15.1805210000
H 1.3598270000 9.1571390000 15.6852690000
H 1.1836570000 10.8924750000 15.8736470000
H 0.8441080000 10.1490730000 14.3157960000
C 3.0957380000 11.7757680000 14.2646210000
H 2.3827340000 11.9860600000 13.4689620000
H 2.9193780000 12.4805370000 15.0814950000
H 4.0911300000 11.9753690000 13.8617740000
C 3.8756010000 10.1834750000 15.9906640000
H 4.9158040000 10.3891560000 15.7266960000
H 3.5960270000 10.8959060000 16.7712870000
H 3.8366570000 9.1898400000 16.4327170000
C 7.7992620000 8.2841970000 10.5954030000
H 8.4526320000 8.6681370000 11.3814150000
H 7.9629480000 7.2055500000 10.4778260000
C 8.0235990000 8.9975780000 9.2850910000
H 9.0268090000 8.7967630000 8.9077080000
H 7.9213740000 10.0823290000 9.4339710000
C 5.7846310000 8.8442870000 8.7515060000
H 5.6544990000 9.9281740000 8.8745690000
H 5.1029870000 8.5041440000 7.9726230000
C 5.4893460000 8.1355120000 10.0445090000
H 5.5337100000 7.0486940000 9.9109750000

H 4.5135880000 8.4137690000 10.4465670000
C 8.3767330000 11.9020000000 13.1169550000
C 8.1454880000 12.5045500000 14.3844920000
C 9.2199570000 12.9265620000 15.1598290000
H 9.0336210000 13.3818590000 16.1249640000
C 10.5267090000 12.8034260000 14.7161770000
H 11.3483730000 13.1520260000 15.3284430000
C 10.7625580000 12.2338990000 13.4798510000
H 11.7828670000 12.1315620000 13.1244930000
C 9.7184620000 11.7733440000 12.6833060000
C 6.7288320000 12.7300450000 14.8701140000
H 6.1298340000 11.8767130000 14.5258990000
C 6.5841150000 12.8097300000 16.3813990000
H 7.0424980000 13.7112460000 16.7898770000
H 5.5291800000 12.8368690000 16.6555480000
H 7.0390480000 11.9554700000 16.8873500000
C 6.1209480000 13.9665680000 14.2133470000
H 6.0756510000 13.8592240000 13.1314620000
H 5.1075770000 14.1463730000 14.5744480000
H 6.7180410000 14.8512610000 14.4401480000
C 10.0400440000 11.1146250000 11.3634770000
H 9.0999150000 10.6957530000 11.0062940000
C 10.5370690000 12.1170110000 10.3277180000
H 11.4938960000 12.5482590000 10.6280220000
H 10.6810710000 11.6420090000 9.3552120000
H 9.8343700000 12.9395620000 10.1991860000
C 11.0307710000 9.9662930000 11.5171550000
H 10.6889480000 9.2383870000 12.2561240000
H 11.1777780000 9.4417170000 10.5710090000
H 12.0103700000 10.3163380000 11.8448470000
C 7.6201230000 9.1888630000 15.1499490000
O 7.1649560000 8.6395360000 13.8987460000
H 7.3625810000 10.2461570000 15.1384410000
H 7.0774820000 8.6912610000 15.9620380000
O 9.4176310000 7.6005360000 15.2108500000
C 9.1039830000 8.9829540000 15.2757670000
H 9.4661450000 9.3519850000 16.2344930000
H 9.6190860000 9.5377920000 14.4786890000
C 8.9627110000 7.0461360000 13.9962880000
H 9.4926960000 7.5041080000 13.1479910000
H 9.2073710000 5.9840330000 14.0157560000
C 7.4766240000 7.2431930000 13.8276370000
H 6.9234460000 6.7203980000 14.6161170000
H 7.1232000000 6.8907810000 12.8595750000

A′

P 3.7635360000 8.9791150000 13.2677880000
Mg 5.9928540000 10.3127020000 12.9395150000
N 4.8155620000 11.1188820000 11.4478210000
N 7.2066000000 11.7532790000 12.4011760000
C 2.9765730000 9.6341410000 11.8639750000
H 1.9607540000 9.3569930000 11.6116570000
C 3.5655000000 10.6591320000 11.1057410000
C 2.9239420000 11.2637690000 9.9942380000
H 1.9449600000 10.9077600000 9.7014360000
C 3.5368010000 12.2818200000 9.3135460000
H 3.0346700000 12.7384420000 8.4684150000
C 4.7984310000 12.7422010000 9.7028640000
H 5.2924510000 13.5572780000 9.1927430000
C 5.3986670000 12.1254810000 10.7820230000
C 6.7395640000 12.5660300000 11.3041590000
H 6.6524810000 13.6332740000 11.5812950000
H 7.4494570000 12.5788080000 10.4568250000
C 3.3577960000 7.1332670000 13.2473450000
C 4.2721880000 6.5268560000 12.1812900000
H 5.3268070000 6.5854760000 12.4558000000
H 4.0319590000 5.4708610000 12.0385660000
H 4.1470710000 7.0349270000 11.2248240000
C 1.9137190000 6.8666400000 12.8259260000
H 1.7135010000 7.2491330000 11.8272980000
H 1.7381820000 5.7883120000 12.8044590000
H 1.1876840000 7.3014340000 13.5094740000
C 3.6151210000 6.4499060000 14.5855130000
H 2.9051460000 6.7622580000 15.3501070000
H 3.5051880000 5.3689510000 14.4711760000
H 4.6187870000 6.6341460000 14.9673330000
C 3.0373400000 9.7900340000 14.8330250000
C 1.5861240000 9.4270770000 15.1102040000
H 1.4703460000 8.3911460000 15.4274100000
H 1.1837420000 10.0543220000 15.9102110000
H 0.9646800000 9.5858040000 14.2276830000
C 3.1174240000 11.2899330000 14.5439910000
H 2.4906950000 11.5723120000 13.6997110000
H 2.8024780000 11.8574760000 15.4228110000
H 4.1375070000 11.6181100000 14.3089070000
C 3.9152850000 9.4976040000 16.0464790000
H 4.9549880000 9.7826080000 15.8570940000
H 3.5780300000 10.0793700000 16.9082310000
H 3.9062360000 8.4495020000 16.3406530000

C 8.3862440000 12.2296130000 12.9895140000
C 8.3265150000 13.1602810000 14.0518380000
C 9.4897500000 13.4747030000 14.7489440000
H 9.4419550000 14.1632230000 15.5855160000
C 10.7079380000 12.9156270000 14.3987730000
H 11.6017710000 13.1621780000 14.9576860000
C 10.7797320000 12.0644690000 13.3080170000
H 11.7411110000 11.6574130000 13.0145220000
C 9.6418440000 11.7316310000 12.5789100000
C 6.9984230000 13.7525080000 14.4810110000
H 6.2980940000 13.6074210000 13.6560740000
C 6.4173700000 13.0109510000 15.6823660000
H 7.1051310000 13.0239380000 16.5309470000
H 5.4758090000 13.4563360000 16.0073190000
H 6.2104910000 11.9629560000 15.4452090000
C 7.0873020000 15.2452740000 14.7665220000
H 7.5157710000 15.7856010000 13.9230560000
H 6.0982510000 15.6590410000 14.9636100000
H 7.7039270000 15.4590510000 15.6406970000
C 9.7351190000 10.8295650000 11.3653880000
H 8.8385440000 11.0143320000 10.7712940000
C 10.9486430000 11.1305420000 10.4971500000
H 11.8835640000 10.8754260000 10.9983020000
H 10.9137390000 10.5507820000 9.5746490000
H 10.9976030000 12.1853570000 10.2291940000
C 9.7052210000 9.3547420000 11.7492870000
H 8.8093110000 9.1221030000 12.3282450000
H 9.7135090000 8.7129980000 10.8668310000
H 10.5731270000 9.0870680000 12.3575670000
C 8.0772690000 9.9383970000 15.4115340000
O 7.2257260000 9.3330950000 14.4080740000
H 8.2331030000 10.9710820000 15.1126590000
H 7.5387100000 9.9093760000 16.3656650000
O 9.1762070000 7.8294290000 15.8086640000
C 9.3823780000 9.1991660000 15.5095470000
H 9.9850540000 9.6186130000 16.3143230000
H 9.9415310000 9.3084720000 14.5720410000
C 8.3722930000 7.2400210000 14.8105720000
H 8.8860230000 7.2688790000 13.8398120000
H 8.2248400000 6.1961260000 15.0876680000
C 7.0411400000 7.9385200000 14.7074340000
H 6.4868880000 7.8569420000 15.6496810000
H 6.4262740000 7.5265890000 13.9076870000

B′
P -2.4932980000 -0.6474020000 -0.6487310000
Mg 0.2014310000 0.1148380000 -1.3100760000
N -0.9831830000 1.8989020000 -0.9038780000
N 1.4148060000 1.1846710000 -0.1033540000
C -2.9691960000 0.8209160000 -1.6887910000
H -4.0469820000 0.9635920000 -1.7795760000
C -2.2886680000 2.0511770000 -1.1859460000
C -2.9323750000 3.2554470000 -0.9655520000
H -3.9843890000 3.3590190000 -1.1970700000
C -2.2041110000 4.3144480000 -0.4353970000
H -2.6854050000 5.2671700000 -0.2545480000
C -0.8737710000 4.1328180000 -0.1142640000
H -0.2893490000 4.9261750000 0.3332800000
C -0.2864360000 2.8933020000 -0.3509570000
C 1.1234760000 2.5757320000 0.0406120000
H 1.2594220000 2.9648390000 1.0656720000
H 1.7900740000 3.2270900000 -0.5647180000
C -3.2761230000 -0.2716960000 1.0347250000
C -2.2829890000 0.6194670000 1.7932400000
H -2.2411570000 1.6321440000 1.3963950000
H -2.5909230000 0.6951560000 2.8389220000
H -1.2693190000 0.2127370000 1.7706830000
C -3.4096720000 -1.5731560000 1.8224380000
H -2.4699160000 -2.1276460000 1.8532440000
H -3.6851350000 -1.3443680000 2.8540120000
H -4.1795230000 -2.2297690000 1.4212080000
C -4.6232070000 0.4395780000 0.9739500000
H -5.3845600000 -0.1377750000 0.4537640000
H -4.9882570000 0.6209910000 1.9879380000
H -4.5437910000 1.4121510000 0.4879540000
C -3.4112790000 -2.0467890000 -1.5162480000
C -2.9961810000 -1.9701800000 -2.9905470000
H -1.9217140000 -1.8123720000 -3.1135640000
H -3.2643040000 -2.9049960000 -3.4866470000
H -3.5101850000 -1.1678200000 -3.5179280000
C -2.8948670000 -3.3759360000 -0.9610190000
H -3.1718010000 -3.5436770000 0.0768950000
H -3.3085960000 -4.2004360000 -1.5454120000
H -1.8086060000 -3.4370580000 -1.0302370000
C -4.9312620000 -2.0020650000 -1.4241120000
H -5.3367990000 -1.0520510000 -1.7735900000
H -5.3592190000 -2.7852310000 -2.0549090000
H -5.2945550000 -2.1739220000 -0.4117720000

C 2.6419080000 0.7817180000 0.4387560000
C 3.7260340000 0.4726540000 -0.4169450000
C 4.8910810000 -0.0750730000 0.1128600000
H 5.7086590000 -0.3259400000 -0.5555740000
C 5.0412250000 -0.2736190000 1.4739530000
H 5.9572540000 -0.6923270000 1.8705690000
C 4.0101310000 0.0899150000 2.3243870000
H 4.1321340000 -0.0443350000 3.3942420000
C 2.8152790000 0.6087840000 1.8372190000
C 3.6698940000 0.7938600000 -1.8960340000
H 2.7149240000 1.2924050000 -2.0784840000
C 4.7821450000 1.7616060000 -2.2876050000
H 5.7687560000 1.3119410000 -2.1624990000
H 4.6889560000 2.0571110000 -3.3332130000
H 4.7562060000 2.6649680000 -1.6782570000
C 3.7059500000 -0.4515230000 -2.7704710000
H 2.8369760000 -1.0830570000 -2.5869630000
H 3.6945930000 -0.1898050000 -3.8289230000
H 4.6039630000 -1.0448820000 -2.5830210000
C 1.7246480000 0.9924840000 2.8172520000
H 0.8230940000 1.1842210000 2.2271970000
C 1.3929560000 -0.1057370000 3.8231460000
H 2.2416660000 -0.3292170000 4.4708810000
H 0.5690590000 0.2016820000 4.4690080000
H 1.1002550000 -1.0385140000 3.3417000000
C 2.0941060000 2.2683680000 3.5725350000
H 2.3498360000 3.0833200000 2.8963100000
H 1.2747110000 2.6025290000 4.2109820000
H 2.9617310000 2.0979130000 4.2129270000
H -0.0017920000 -0.2761720000 -3.0165760000
H -2.5703470000 0.5864070000 -2.6802730000
C 2.1621370000 -2.8170530000 1.0853930000
O 2.3321110000 -3.9781400000 0.2890500000
H 2.1266730000 -3.1429240000 2.1249680000
H 3.0168460000 -2.1385650000 0.9626030000
C 0.8931790000 -2.1020180000 0.7015820000
C 2.4029040000 -3.6181320000 -1.0783030000
O 0.9351190000 -1.7632350000 -0.6877030000
C 1.1385610000 -2.9191140000 -1.5065910000
H 0.7743000000 -1.1557440000 1.2329820000
H 0.0119260000 -2.7329980000 0.8809190000
H 0.2782390000 -3.5895030000 -1.3923870000
H 1.1674640000 -2.5587440000 -2.5354160000
H 3.2676480000 -2.9623560000 -1.2495790000

H 2.5461790000 -4.5379630000 -1.6451290000

TS-IV

P -2.7547990000 -0.4012690000 -0.1057440000
Mg -0.1887480000 -0.0725720000 -0.7569260000
N -1.0253050000 1.8033200000 -0.9593240000
N 1.2884730000 1.0455970000 0.0075840000
C -3.0111960000 0.6963550000 -1.5222730000
H -4.0134300000 0.7603020000 -1.9414500000
C -2.2975350000 1.9529500000 -1.3984860000
C -2.7495250000 3.2328360000 -1.7313560000
H -3.7532490000 3.3733370000 -2.1107350000
C -1.9033710000 4.3088570000 -1.5282330000
H -2.2512810000 5.3100240000 -1.7525760000
C -0.6201090000 4.1249200000 -1.0236300000
H 0.0399960000 4.9630900000 -0.8448900000
C -0.2020660000 2.8282450000 -0.7608480000
C 1.1734650000 2.4588290000 -0.2700080000
H 1.3913660000 3.0755150000 0.6201970000
H 1.8819060000 2.8404450000 -1.0266620000
C -3.5358090000 0.4186160000 1.4059870000
C -2.5991050000 1.5681180000 1.7959270000
H -2.6656950000 2.4047370000 1.1030670000
H -2.8795260000 1.9409060000 2.7840580000
H -1.5550680000 1.2554480000 1.8381310000
C -3.5754370000 -0.5723870000 2.5654990000
H -2.5980220000 -1.0280820000 2.7393840000
H -3.8615570000 -0.0532230000 3.4830670000
H -4.2995430000 -1.3710170000 2.4089270000
C -4.9201030000 1.0063820000 1.1522650000
H -5.6811820000 0.2498250000 0.9796510000
H -5.2335400000 1.5892070000 2.0219980000
H -4.9109890000 1.6803010000 0.2949390000
C -3.6506670000 -1.9967950000 -0.5455850000
C -3.2865130000 -2.3323180000 -1.9925690000
H -2.2070660000 -2.3471540000 -2.1447420000
H -3.6729060000 -3.3242850000 -2.2369320000
H -3.7081380000 -1.6226140000 -2.7017570000
C -3.0958840000 -3.1178060000 0.3372320000
H -3.3100950000 -2.9744810000 1.3939440000
H -3.5386890000 -4.0716410000 0.0412570000
H -2.0138680000 -3.2040590000 0.2258300000
C -5.1683560000 -1.9408870000 -0.4236160000
H -5.5948840000 -1.1162630000 -0.9949130000

H -5.6012330000 -2.8660110000 -0.8125500000
H -5.4973150000 -1.8442910000 0.6098860000
C 2.4585100000 0.6424030000 0.6556440000
C 3.7379070000 0.7110020000 0.0478950000
C 4.8608220000 0.2662530000 0.7397700000
H 5.8338620000 0.3361590000 0.2655790000
C 4.7616240000 -0.2905340000 2.0003280000
H 5.6446270000 -0.6407370000 2.5190580000
C 3.5124870000 -0.3983210000 2.5894930000
H 3.4346280000 -0.8325920000 3.5787480000
C 2.3672490000 0.0684130000 1.9541720000
C 3.9105220000 1.2074850000 -1.3710130000
H 2.9114930000 1.2535060000 -1.8139290000
C 4.5362130000 2.5997830000 -1.4098620000
H 5.5544780000 2.5696380000 -1.0170270000
H 4.5891840000 2.9842090000 -2.4294780000
H 3.9794570000 3.3161800000 -0.8073260000
C 4.7411280000 0.2486060000 -2.2195250000
H 4.3729200000 -0.7757050000 -2.1547300000
H 4.7258490000 0.5467880000 -3.2683570000
H 5.7855480000 0.2334890000 -1.9060280000
C 1.0371270000 0.0345270000 2.6821620000
H 0.2504290000 -0.1783660000 1.9438840000
C 0.9402560000 -1.0263050000 3.7683920000
H 1.5855400000 -0.7994730000 4.6179850000
H -0.0784780000 -1.0833830000 4.1521190000
H 1.2180900000 -2.0184500000 3.4083340000
C 0.7275800000 1.4110690000 3.2659250000
H 0.6684780000 2.1668510000 2.4840870000
H -0.2188840000 1.4099760000 3.8093890000
H 1.5118740000 1.7096990000 3.9632800000
H -1.0352510000 -0.3260840000 -2.5164130000
H -1.9682090000 0.0338550000 -2.2758570000
C 2.6721290000 -2.8615280000 -0.1279780000
O 2.8071860000 -3.5774060000 -1.3449600000
H 3.2070160000 -3.4232770000 0.6371450000
H 3.1366070000 -1.8699820000 -0.2094930000
C 1.2200430000 -2.7130980000 0.2433670000
C 2.1063790000 -2.9229760000 -2.3826410000
O 0.5070130000 -2.0504270000 -0.8180560000
C 0.6466040000 -2.7862480000 -2.0473040000
H 1.0927440000 -2.1029680000 1.1360860000
H 0.7518200000 -3.6902980000 0.4049680000
H 0.1769710000 -3.7667390000 -1.9107010000

H 0.0977830000 -2.2210060000 -2.8016250000
H 2.5289160000 -1.9228520000 -2.5597510000
H 2.2415230000 -3.5170020000 -3.2862900000

dioxane
C 0.6645790000 1.1996380000 -0.2489610000
C 1.3724620000 -0.0403870000 0.2389360000
O 0.6986700000 -1.2053710000 -0.2016570000
C -0.6457970000 -1.1908840000 0.2429000000
C -1.3536800000 0.0491410000 -0.2449970000
O -0.6798880000 1.2141240000 0.1955960000
H 0.6951330000 1.2360910000 -1.3476140000
H 1.1342780000 2.1042420000 0.1378250000
H 1.4225980000 -0.0307060000 1.3374370000
H 2.3890250000 -0.0975420000 -0.1507330000
H -0.6763510000 -1.2273380000 1.3415540000
H -1.1154950000 -2.0954890000 -0.1438860000
H -2.3702420000 0.1062950000 0.1446730000
H -1.4038160000 0.0394600000 -1.3434980000

7 NMR spectra

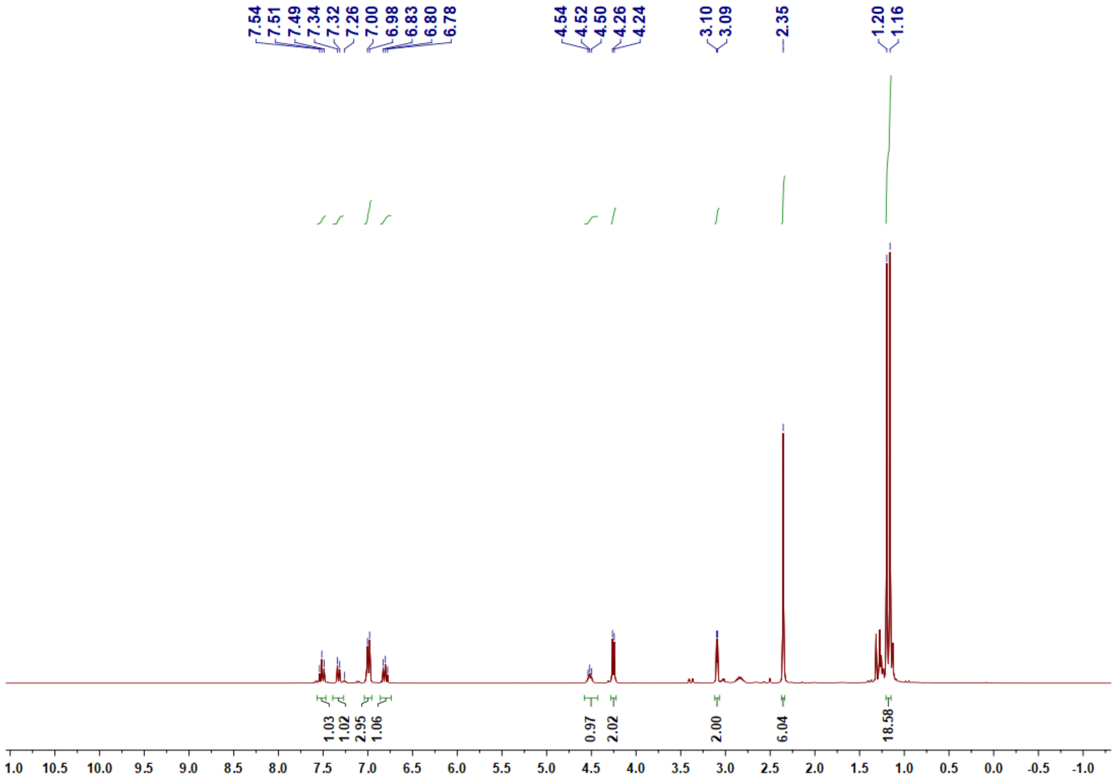

Figure S62. ^{1}H NMR (300 MHz, $CDCl_3$) spectrum of **L2**

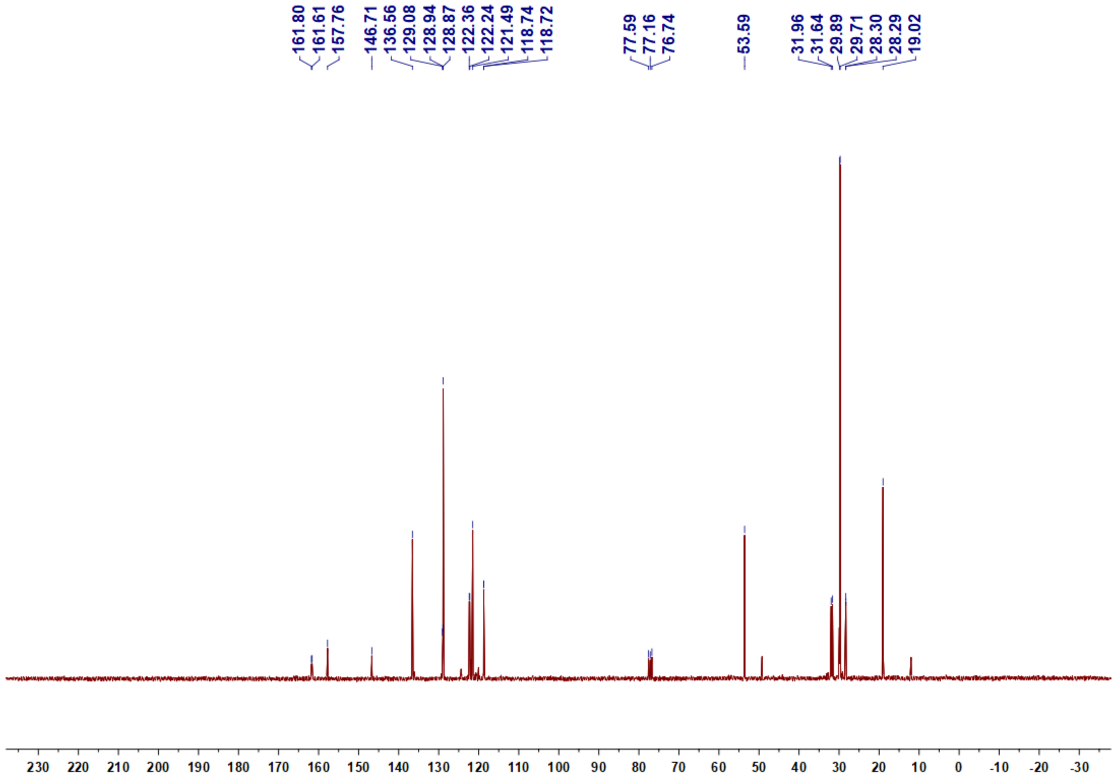

Figure S63. ^{13}C NMR (75 MHz, $CDCl_3$) spectrum of **L2**

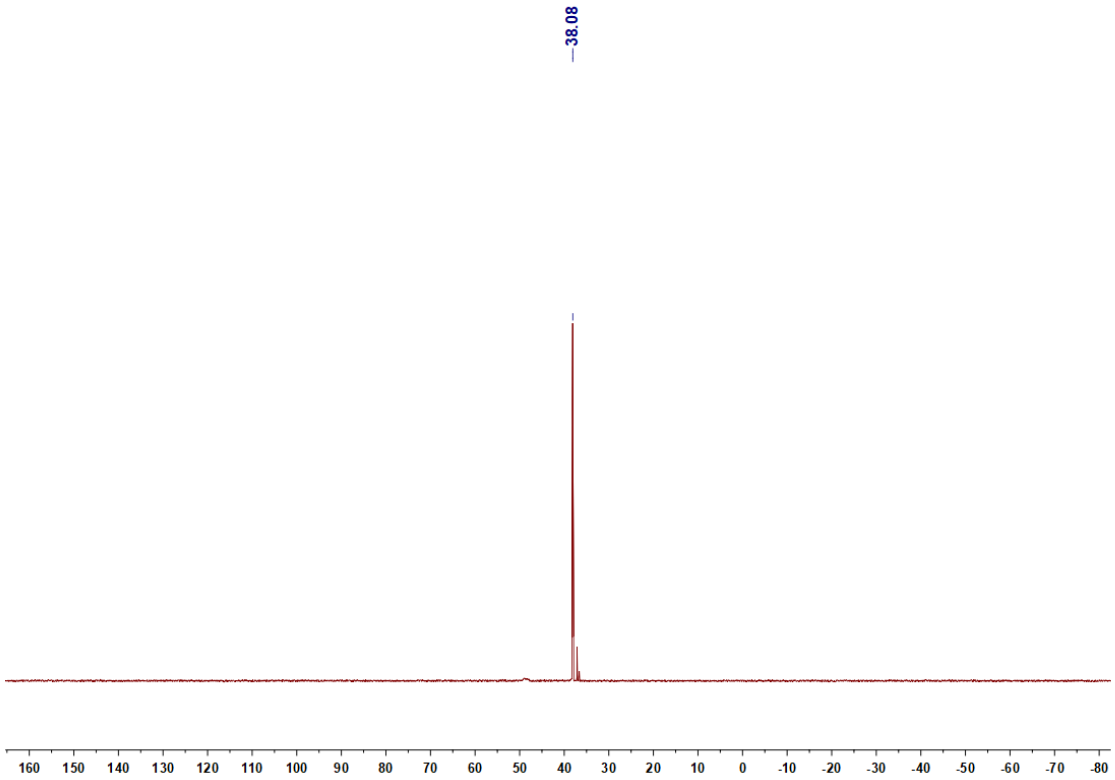

Figure S64. ^{31}P NMR (121 MHz, $CDCl_3$) spectrum of **L2**

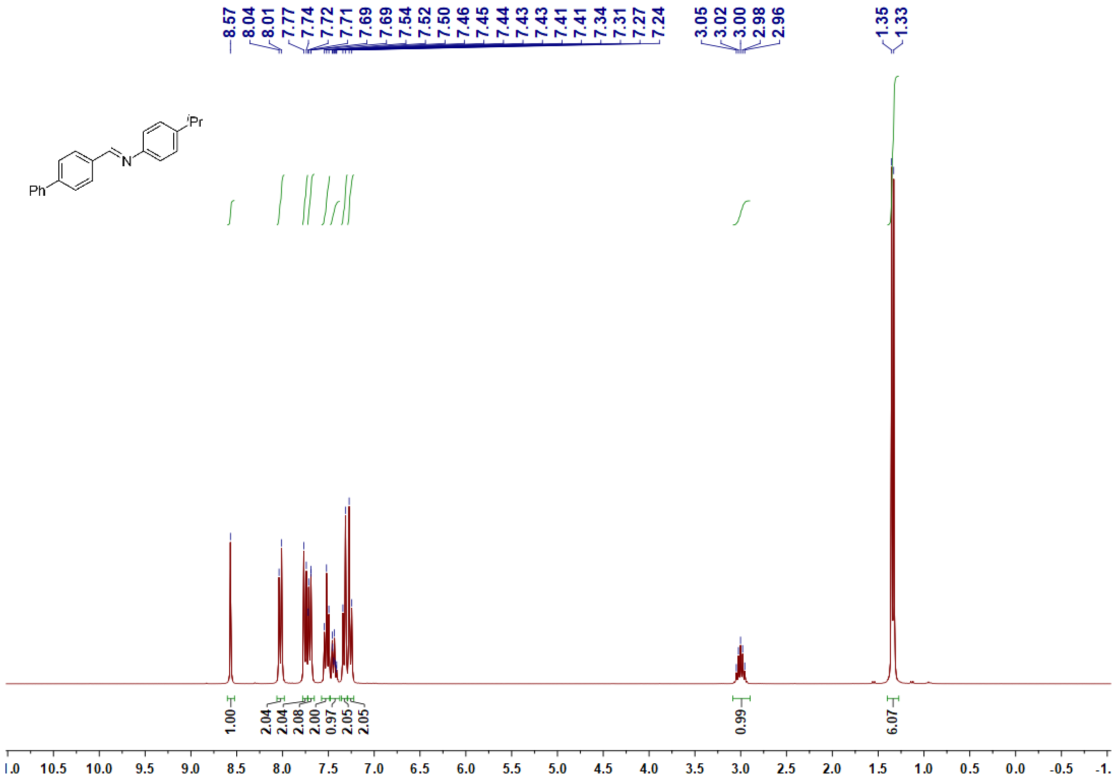

Figure S65. ^{1}H NMR (300 MHz, $CDCl_3$) spectrum of **1l**

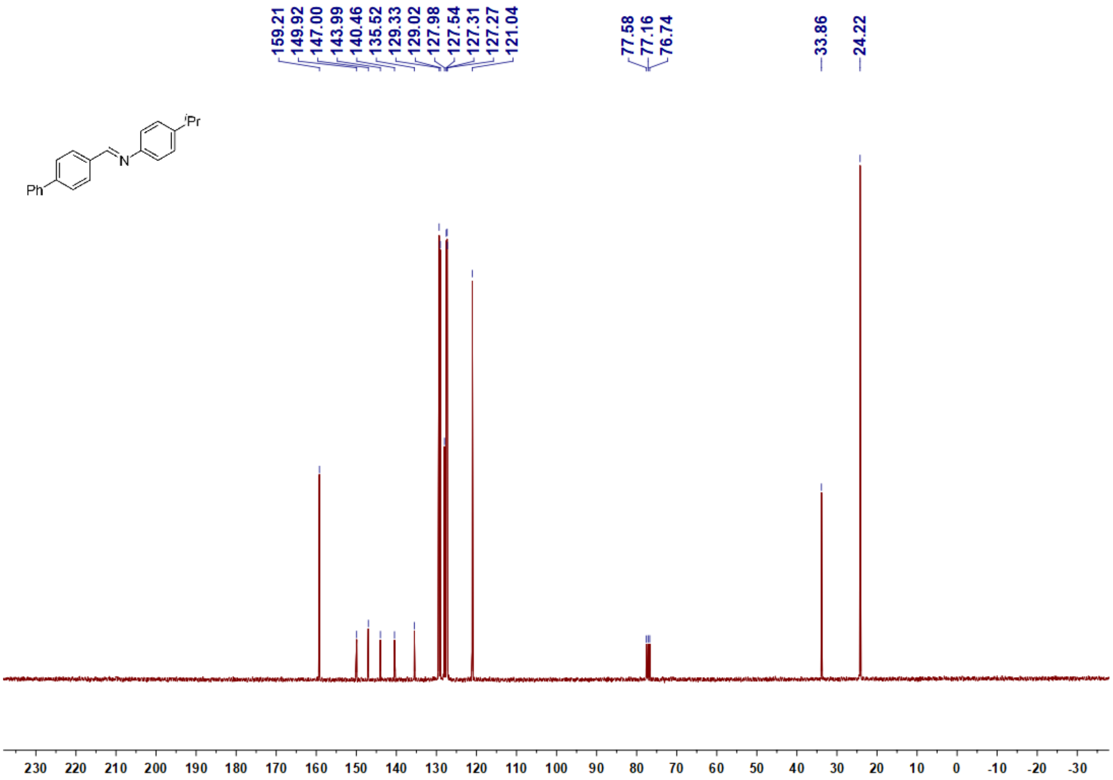

Figure S66. ^{13}C NMR (75 MHz, $CDCl_3$) spectrum of **1l**

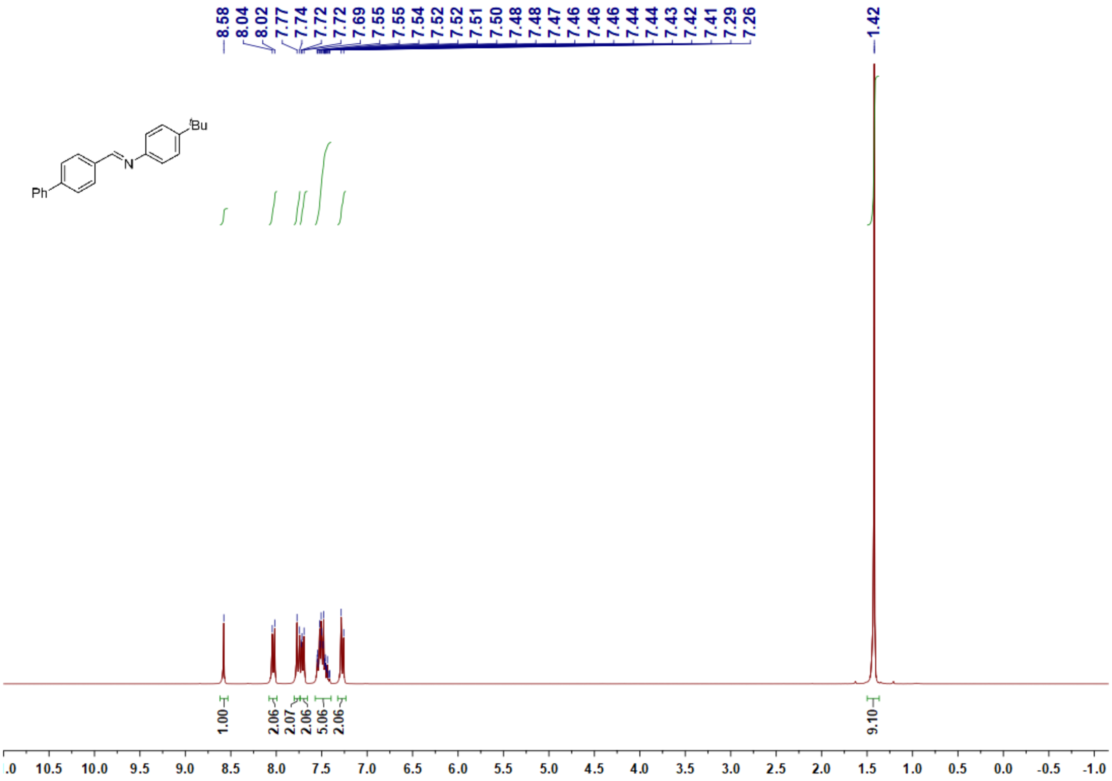

Figure S67. ^{1}H NMR (300 MHz, $CDCl_3$) spectrum of **1m**

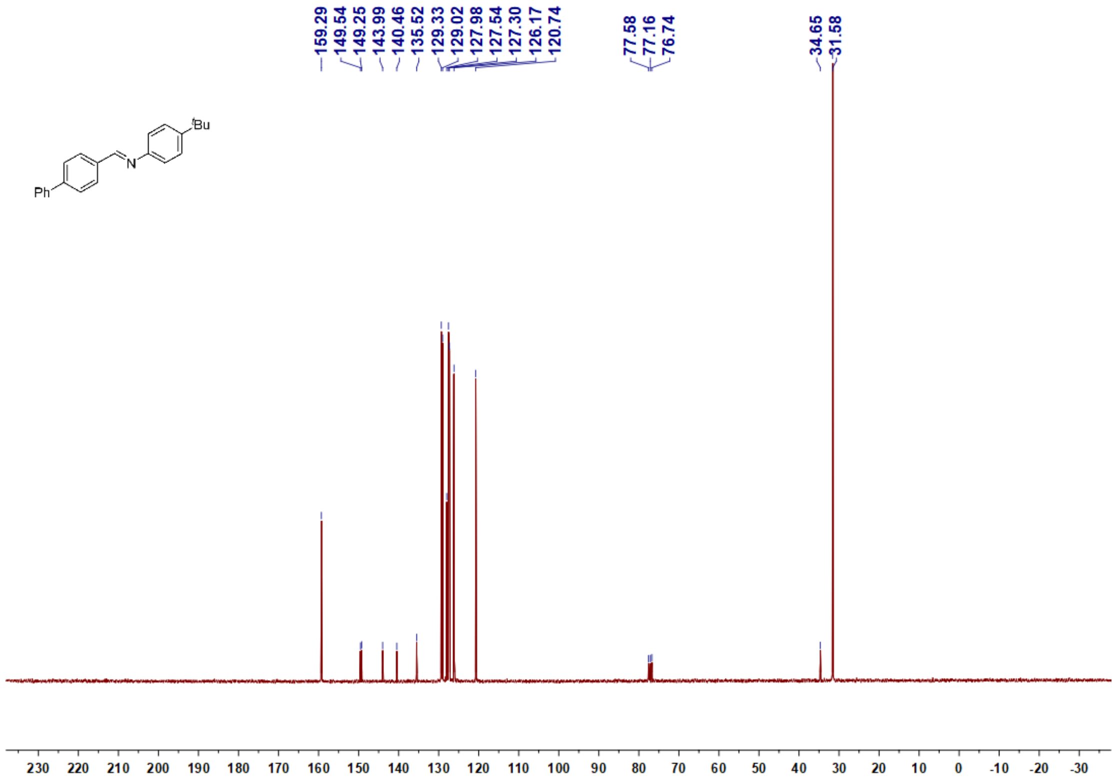

Figure S68. ^{13}C NMR (75 MHz, $CDCl_3$) spectrum of **1m**

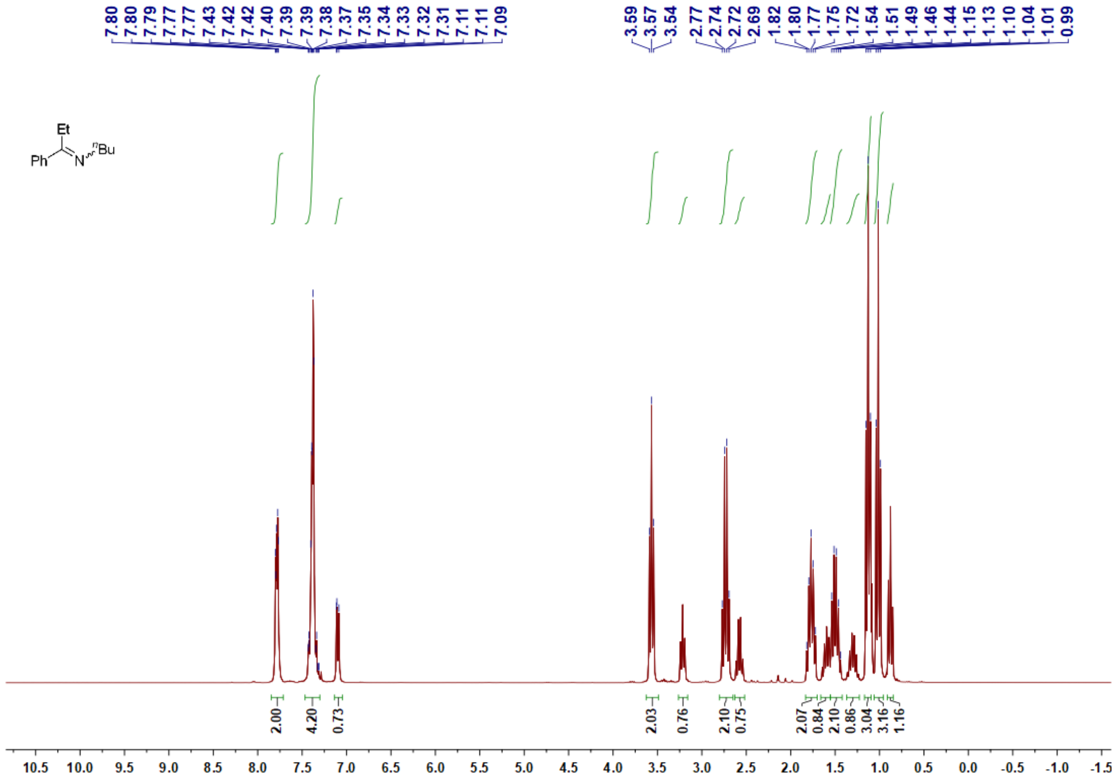

Figure S69. ^{1}H NMR (300 MHz, $CDCl_3$) spectrum of **1r**

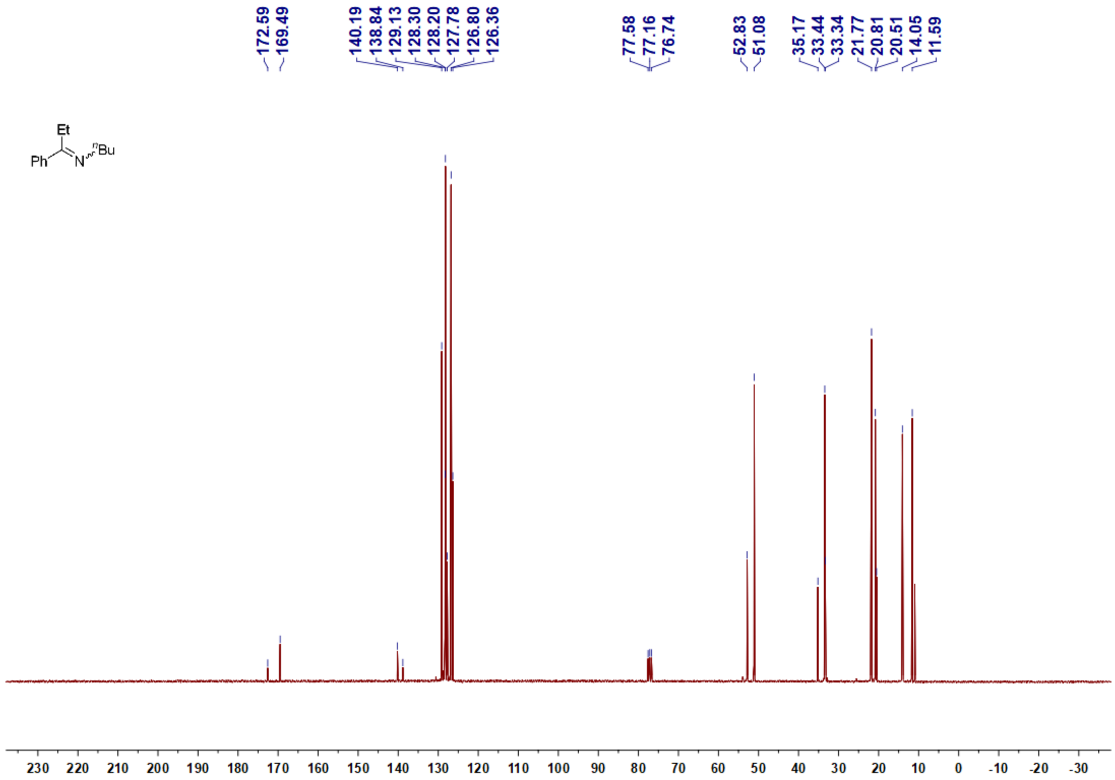

Figure S70. ^{13}C NMR (75 MHz, $CDCl_3$) spectrum of **1r**

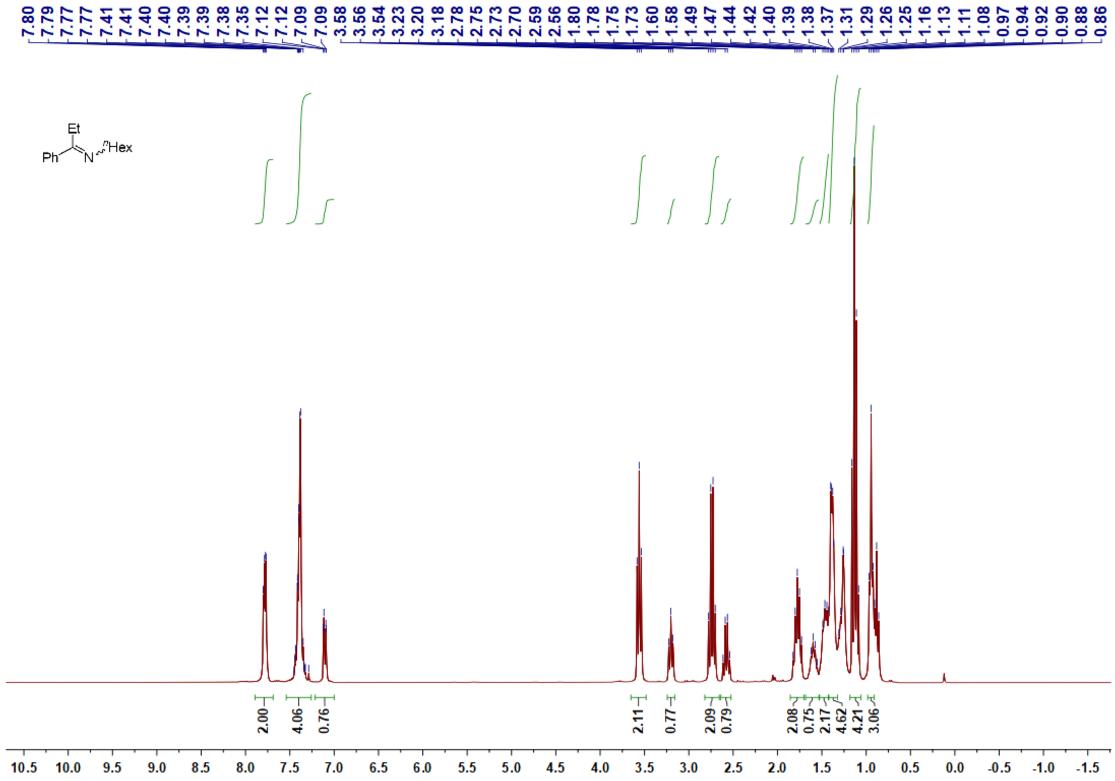

Figure S71. ^{1}H NMR (300 MHz, $CDCl_3$) spectrum of **1s**

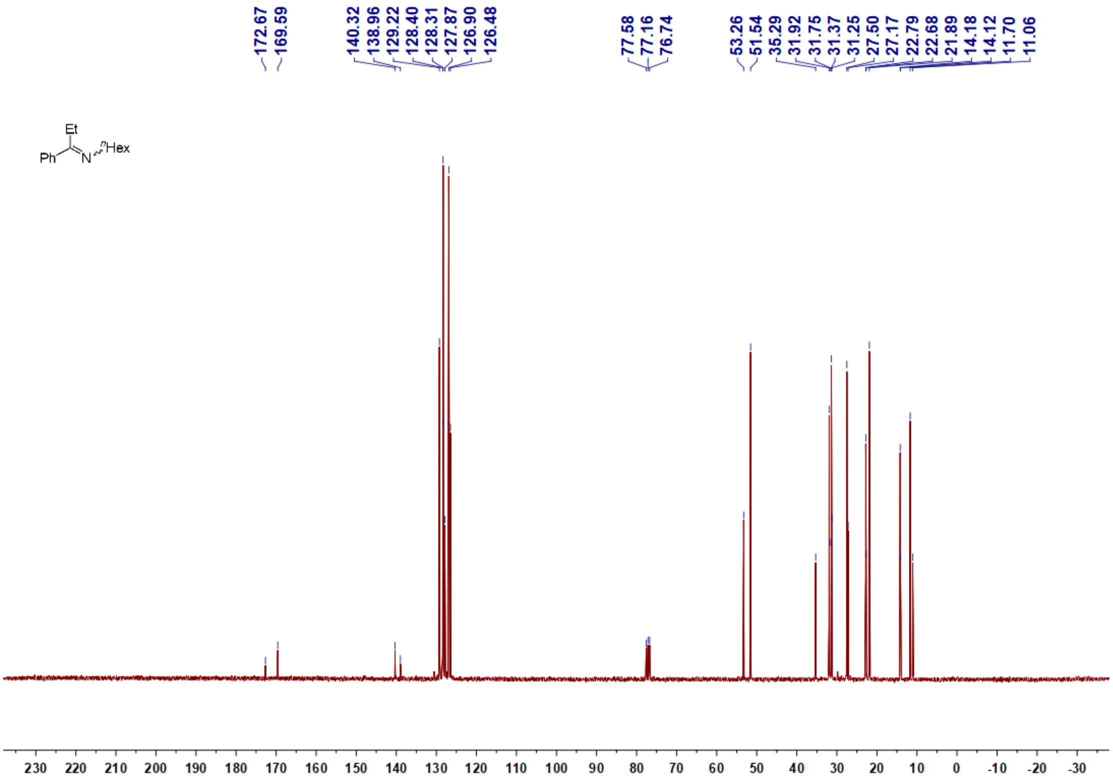

Figure S72. ^{13}C NMR (75 MHz, $CDCl_3$) spectrum of **1s**

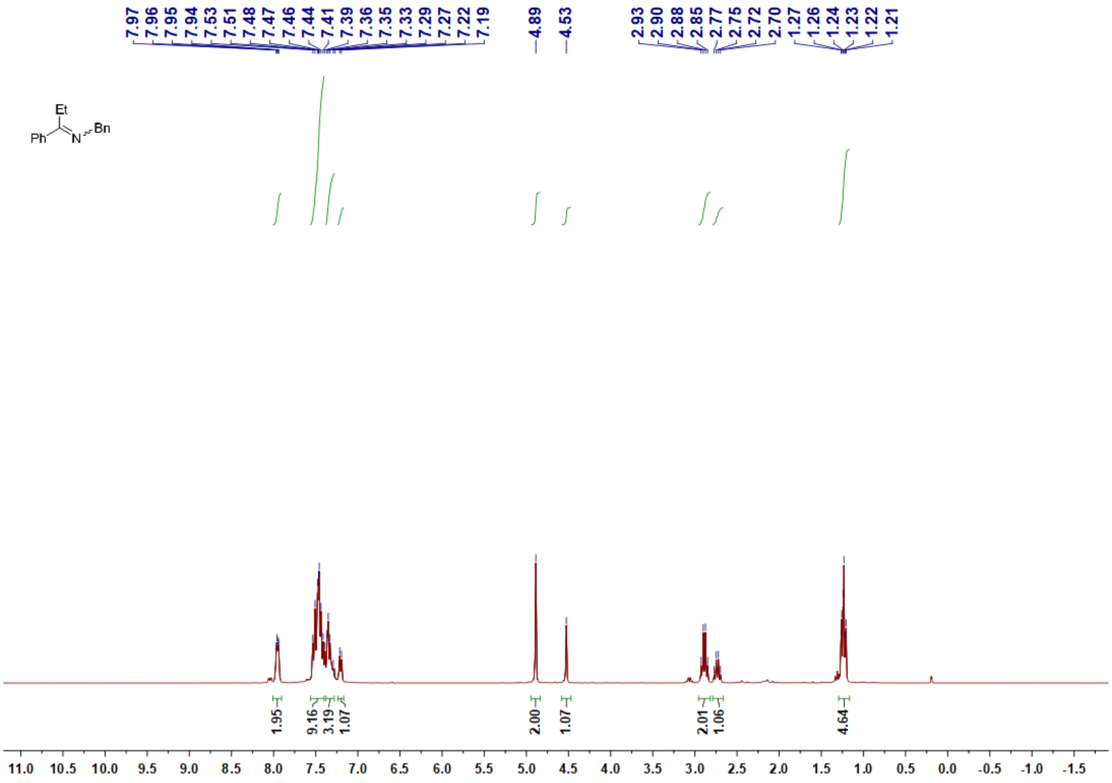

Figure S73. ^{1}H NMR (300 MHz, $CDCl_3$) spectrum of **1t**

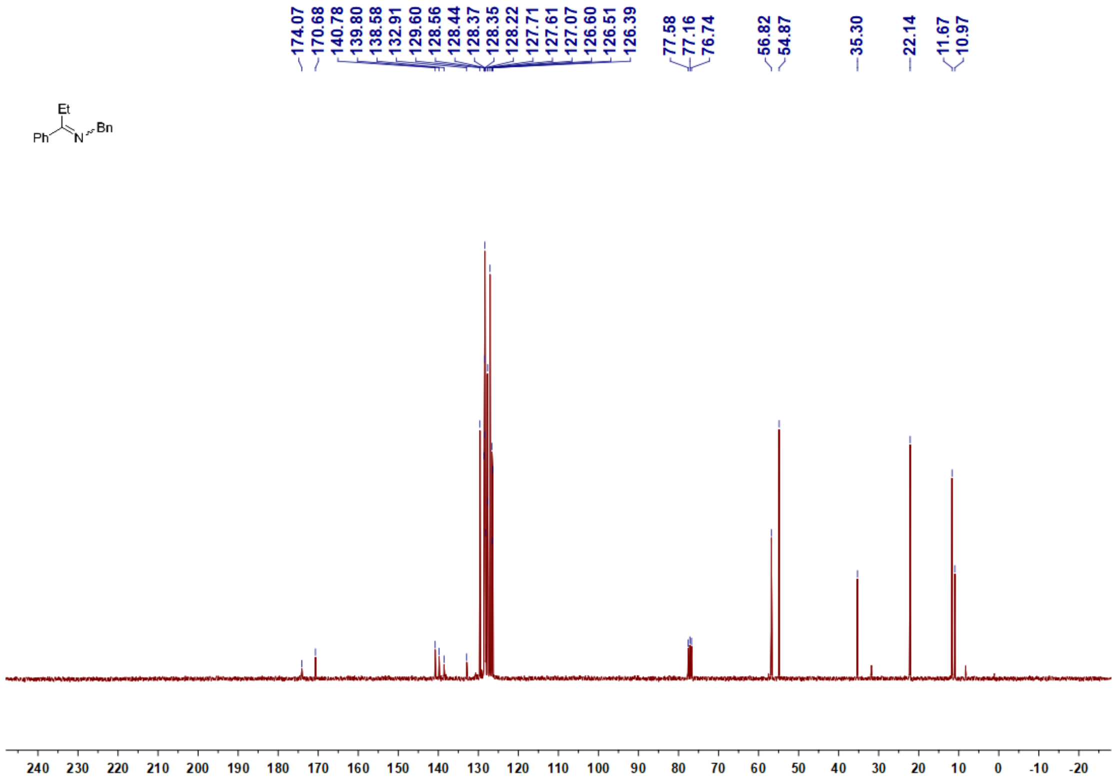

Figure S74. ^{13}C NMR (75 MHz, $CDCl_3$) spectrum of **1t**

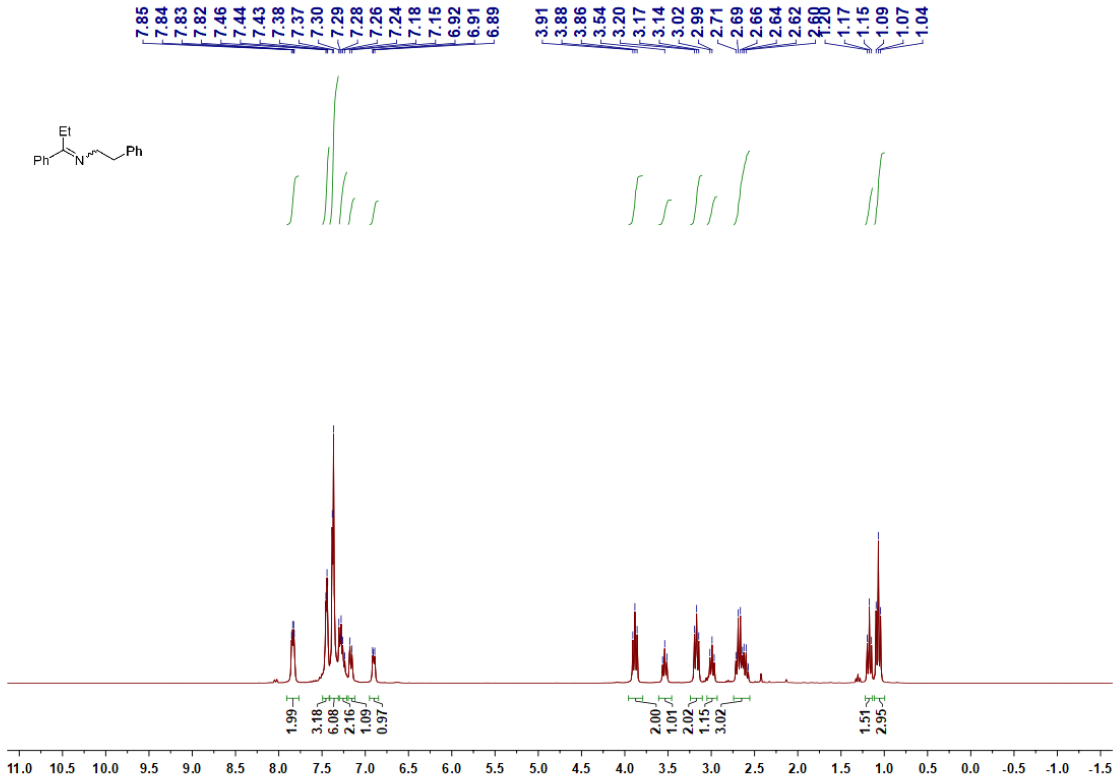

Figure S75. ^{1}H NMR (300 MHz, $CDCl_3$) spectrum of **1u**

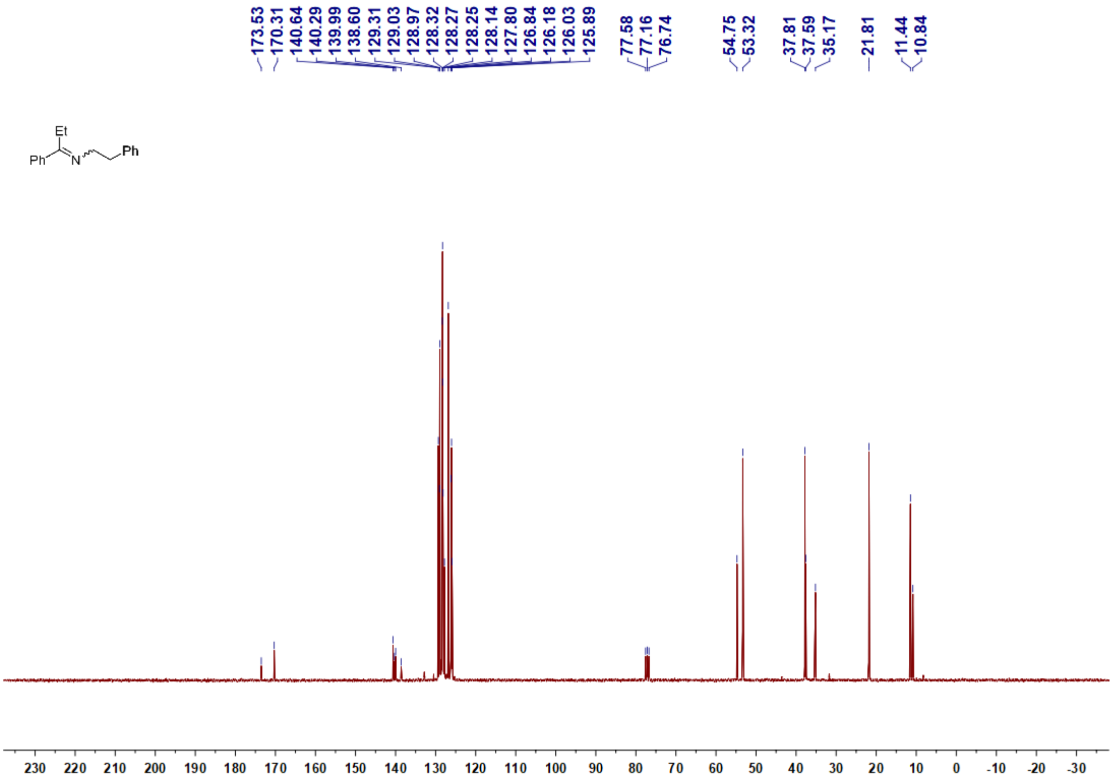

Figure S76. ^{13}C NMR (75 MHz, $CDCl_3$) spectrum of **1u**

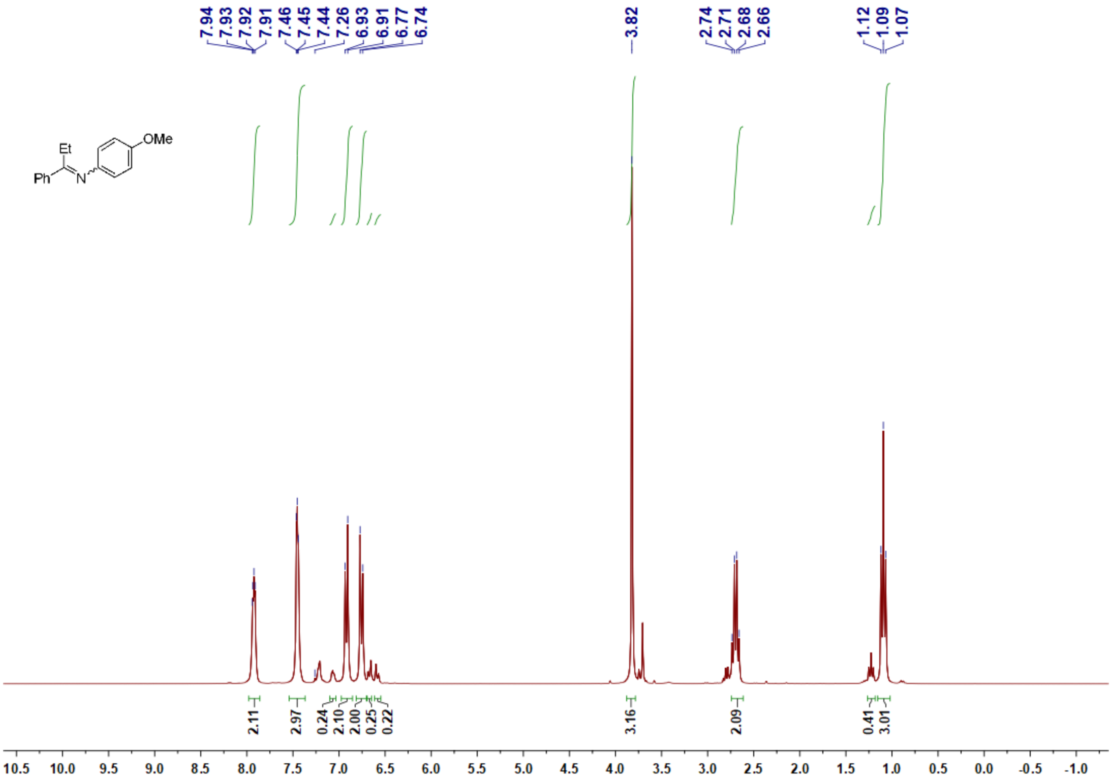

Figure S77. ^{1}H NMR (300 MHz, $CDCl_3$) spectrum of **1v**

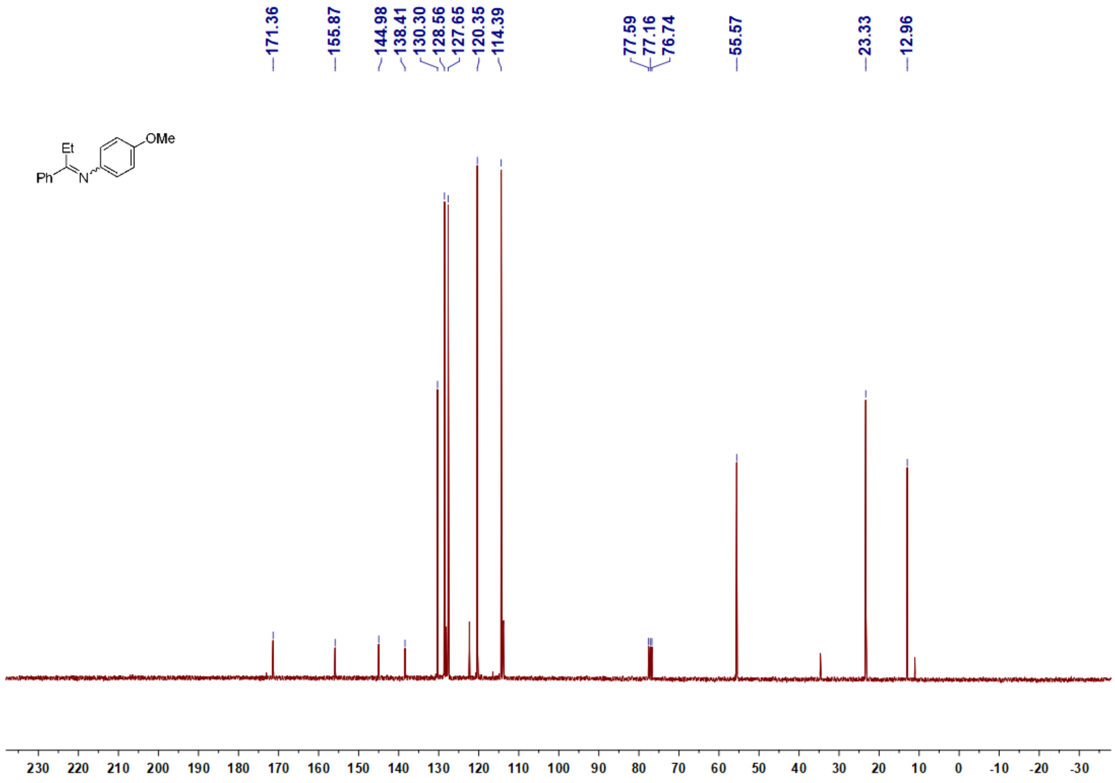

Figure S78. ^{13}C NMR (75 MHz, $CDCl_3$) spectrum of **1v**

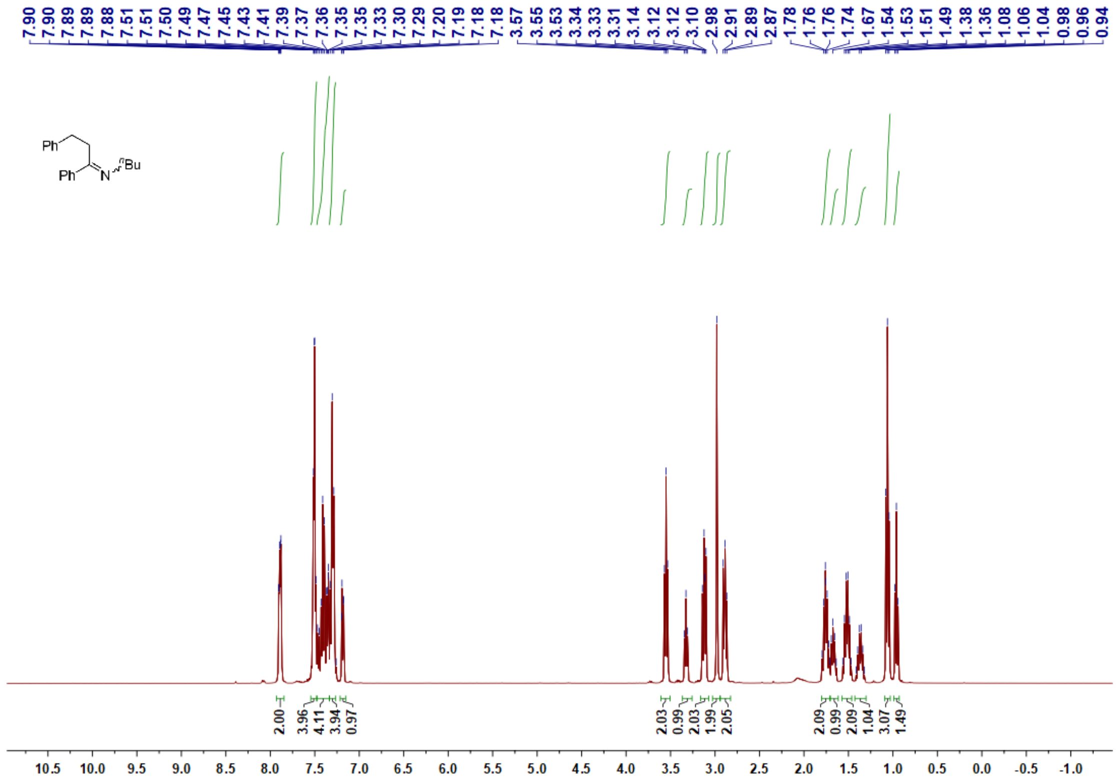

Figure S79. ^{1}H NMR (400 MHz, $CDCl_3$) spectrum of **1w**

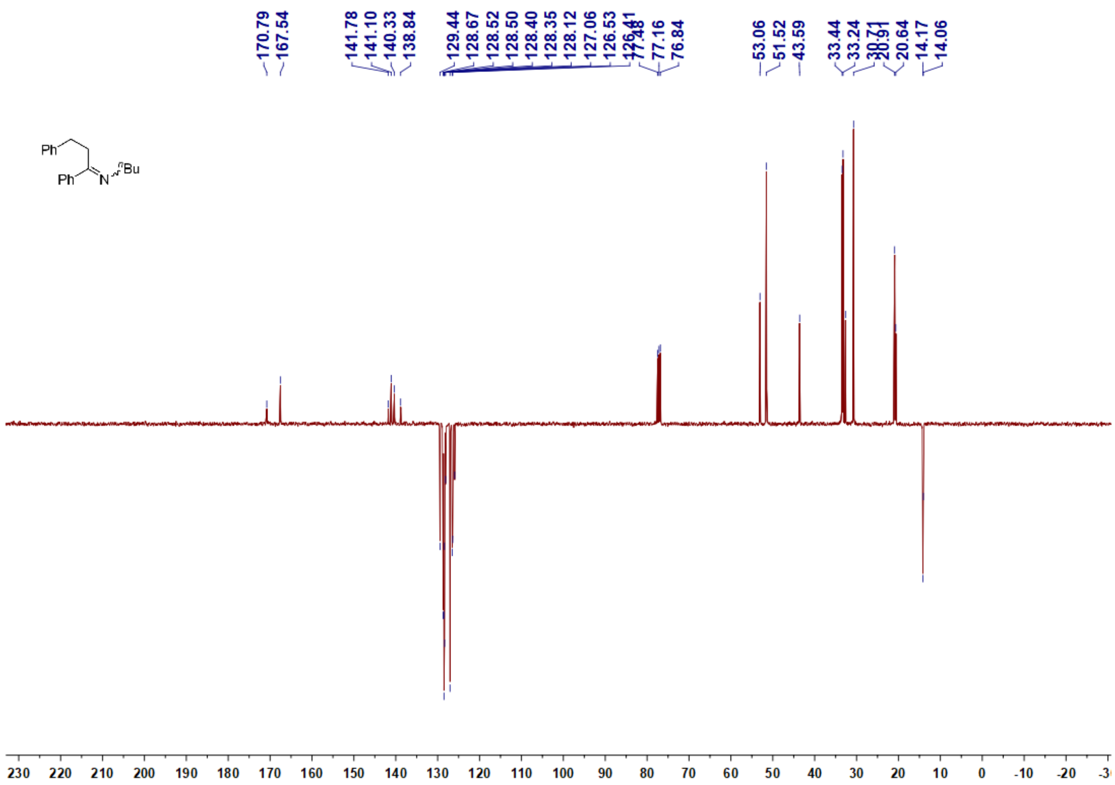

Figure S80. ^{13}C-DEPTQ NMR (101 MHz, $CDCl_3$) spectrum of **1w**

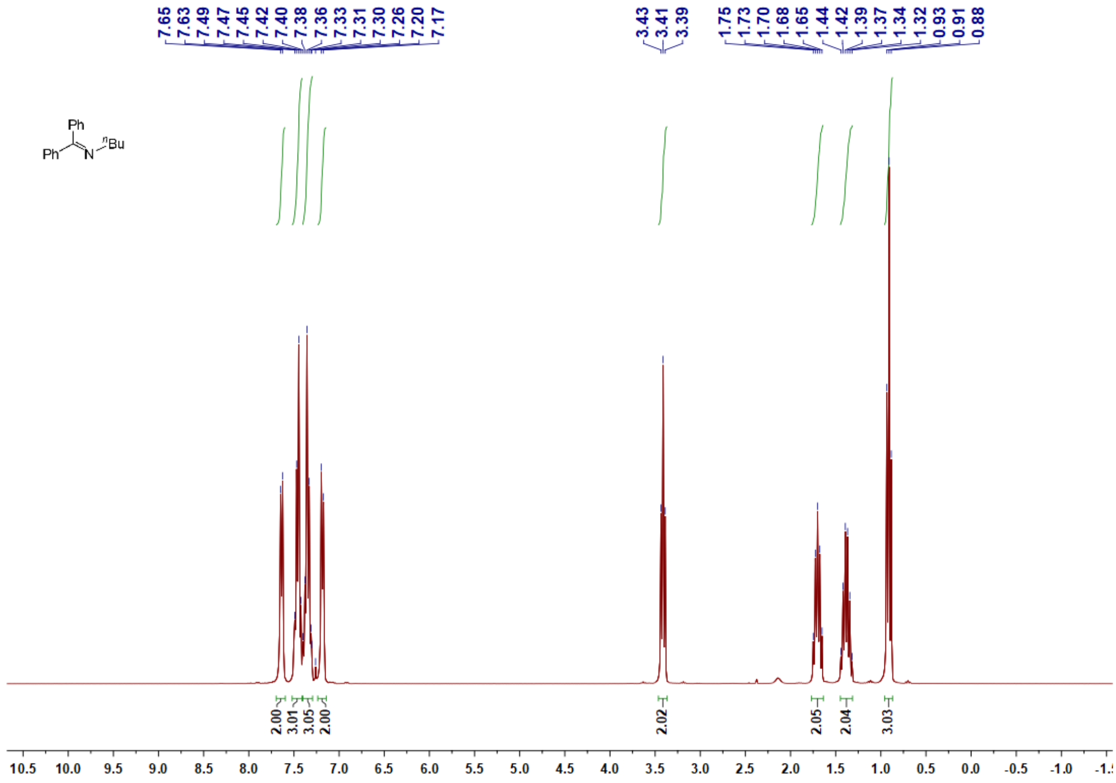

Figure S81. ^{1}H NMR (300 MHz, $CDCl_3$) spectrum of **1x**

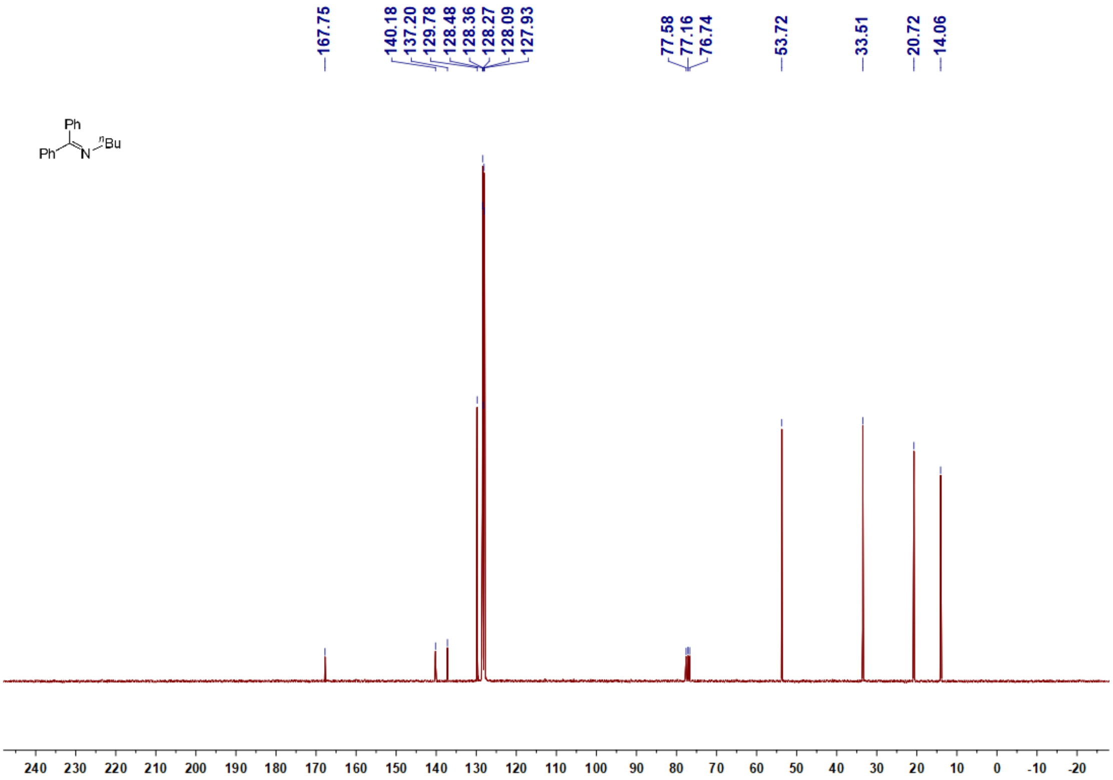

Figure S82. ^{13}C NMR (75 MHz, $CDCl_3$) spectrum of **1x**

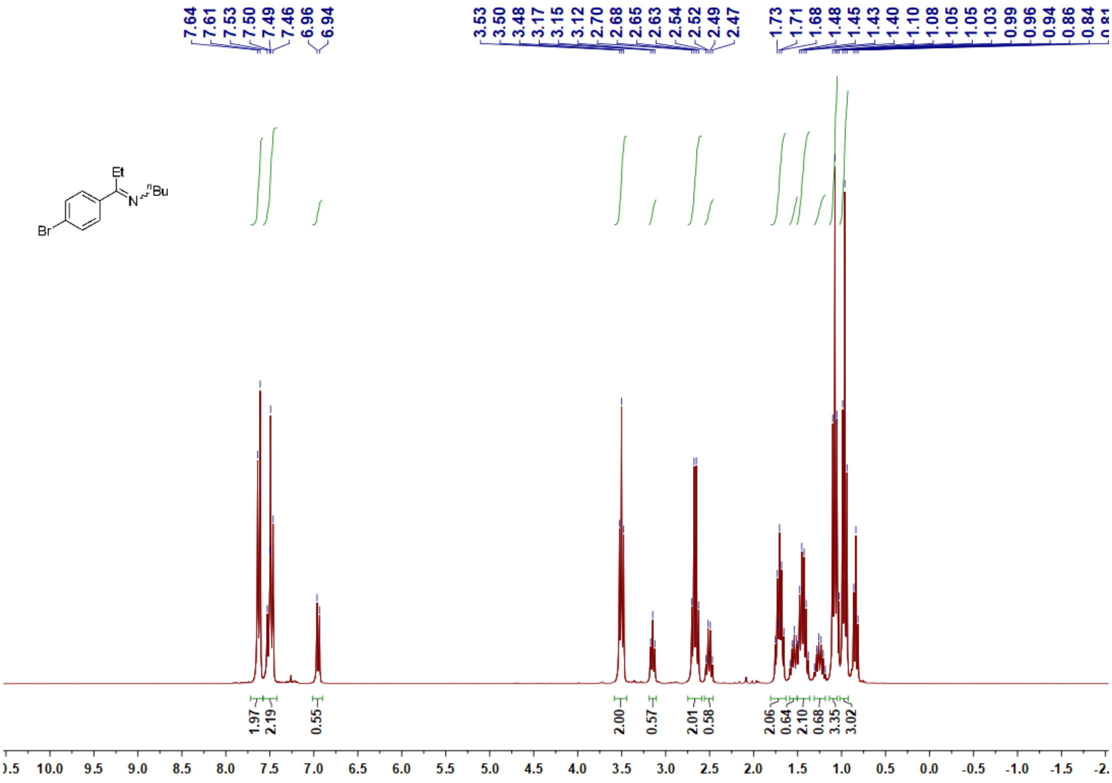

Figure S83. ^{1}H NMR (300 MHz, $CDCl_3$) spectrum of **1y**

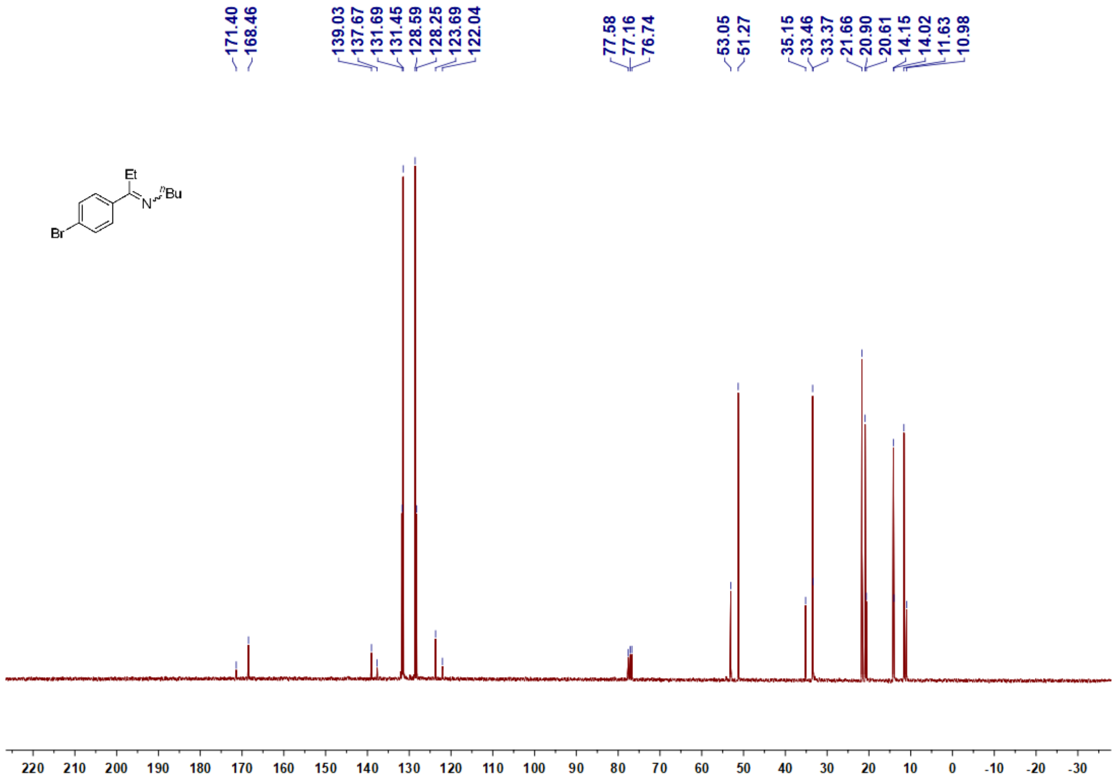

Figure S84. ^{13}C NMR (75 MHz, $CDCl_3$) spectrum of **1y**

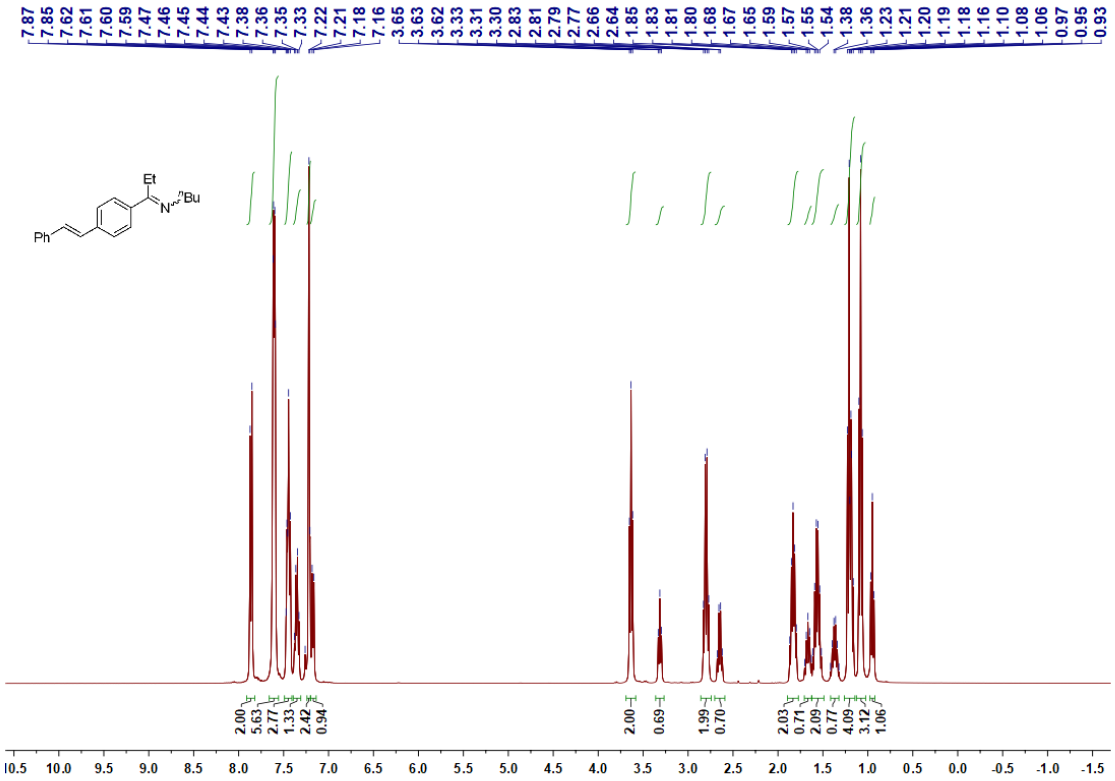

Figure S85. ^{1}H NMR (400 MHz, $CDCl_3$) spectrum of **1z**

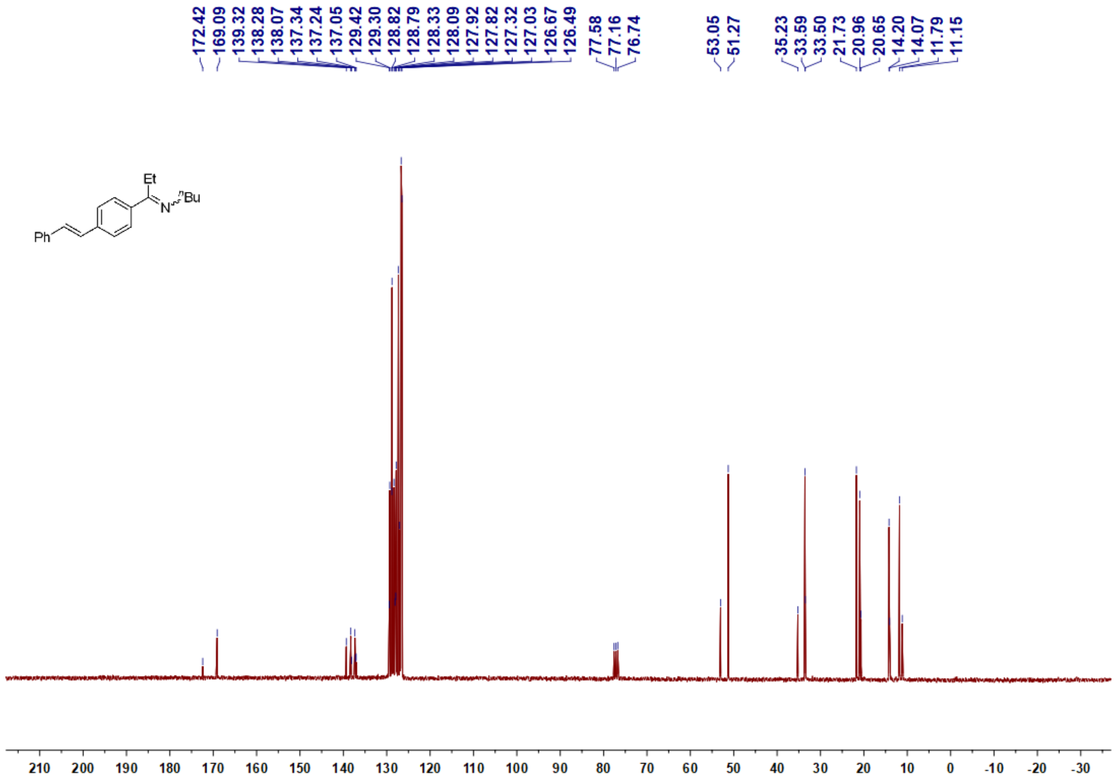

Figure S86. ^{13}C NMR (75 MHz, CDCl$_3$) spectrum of **1z**

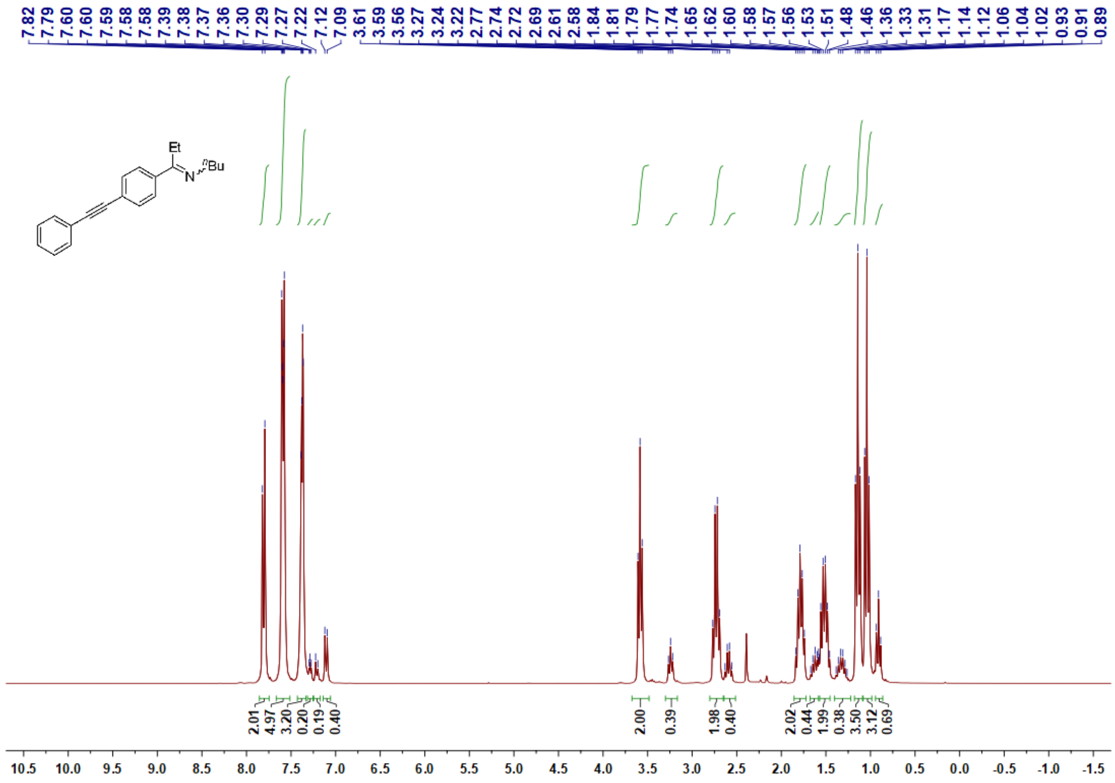

Figure S87. ^{1}H NMR (300 MHz, CDCl$_3$) spectrum of **1aa**

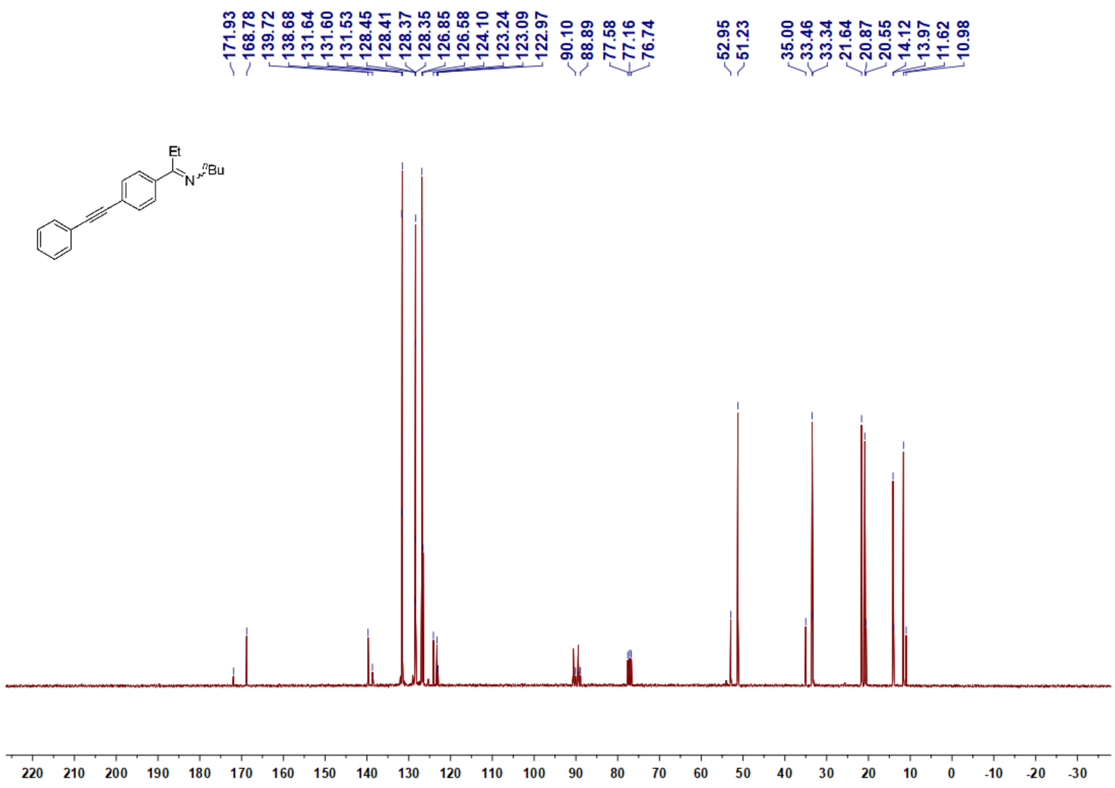

Figure S88. ^{13}C NMR (75 MHz, $CDCl_3$) spectrum of **1aa**

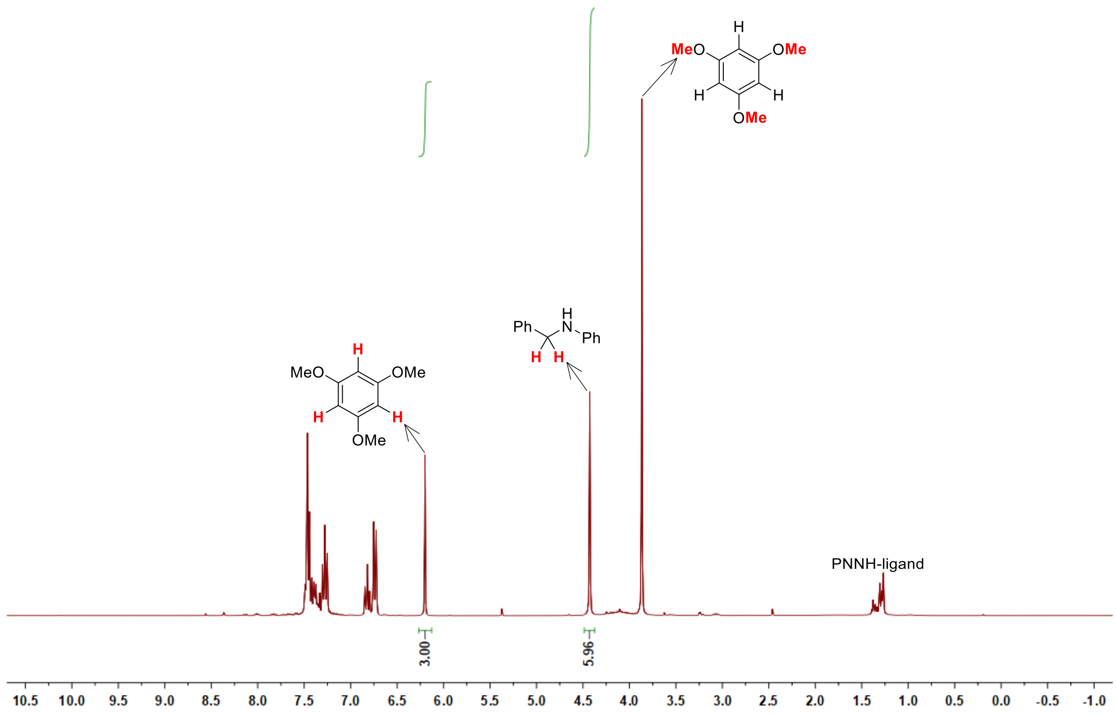

Figure S89. ^{1}H NMR (300 MHz, $CDCl_3$) spectrum of **2a**

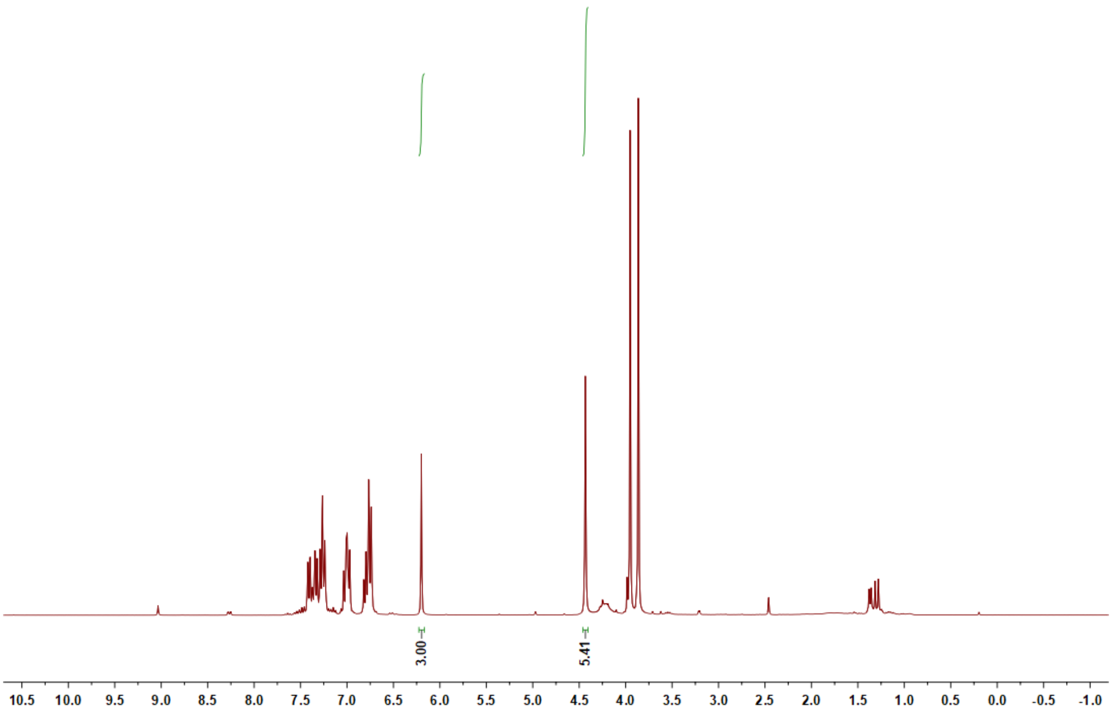

Figure S90. ^{1}H NMR (300 MHz, $CDCl_3$) spectrum of **2b**

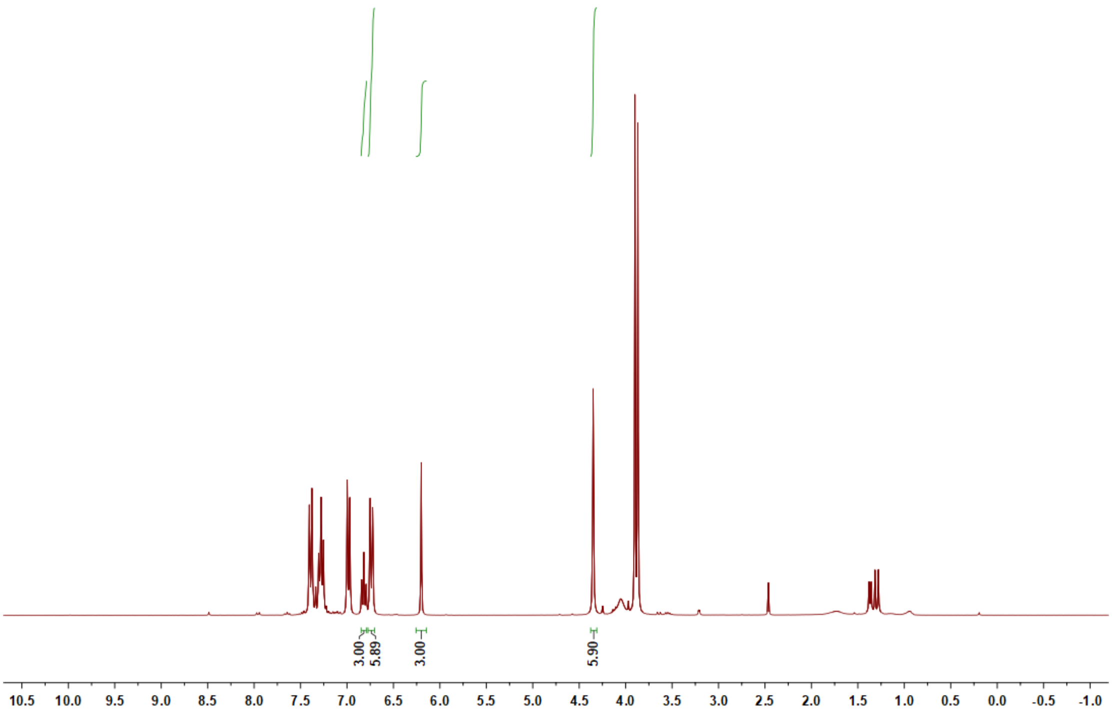

Figure S91. ^{1}H NMR (300 MHz, $CDCl_3$) spectrum of **2c**

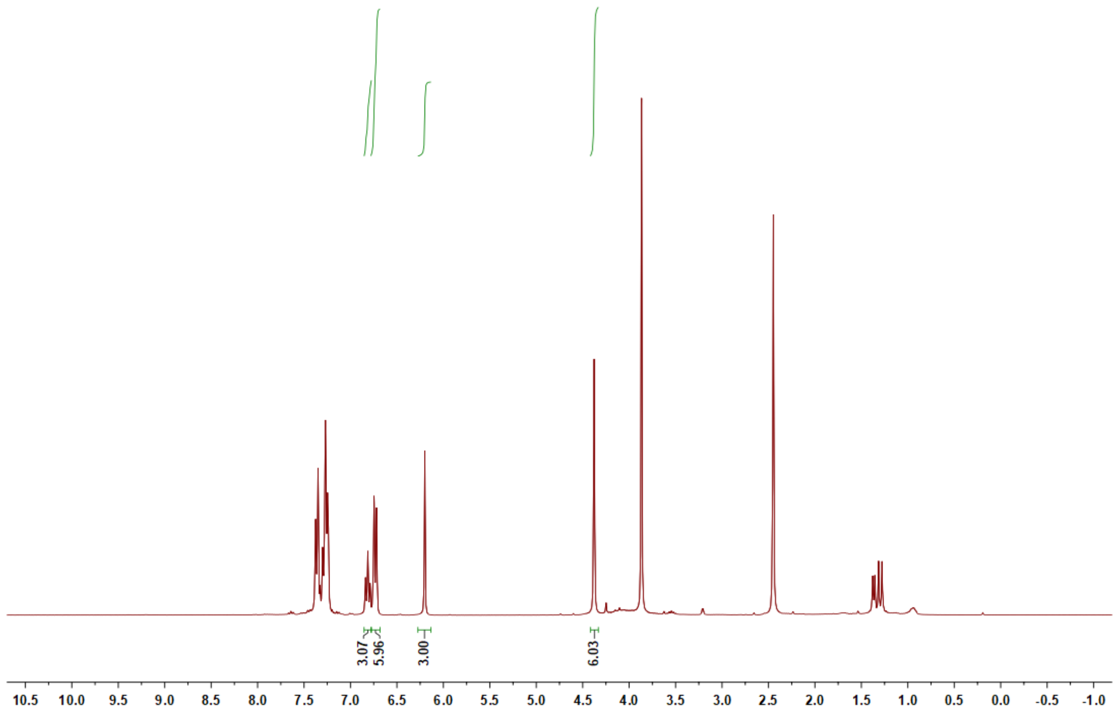

Figure S92. ^{1}H NMR (300 MHz, $CDCl_3$) spectrum of **2d**

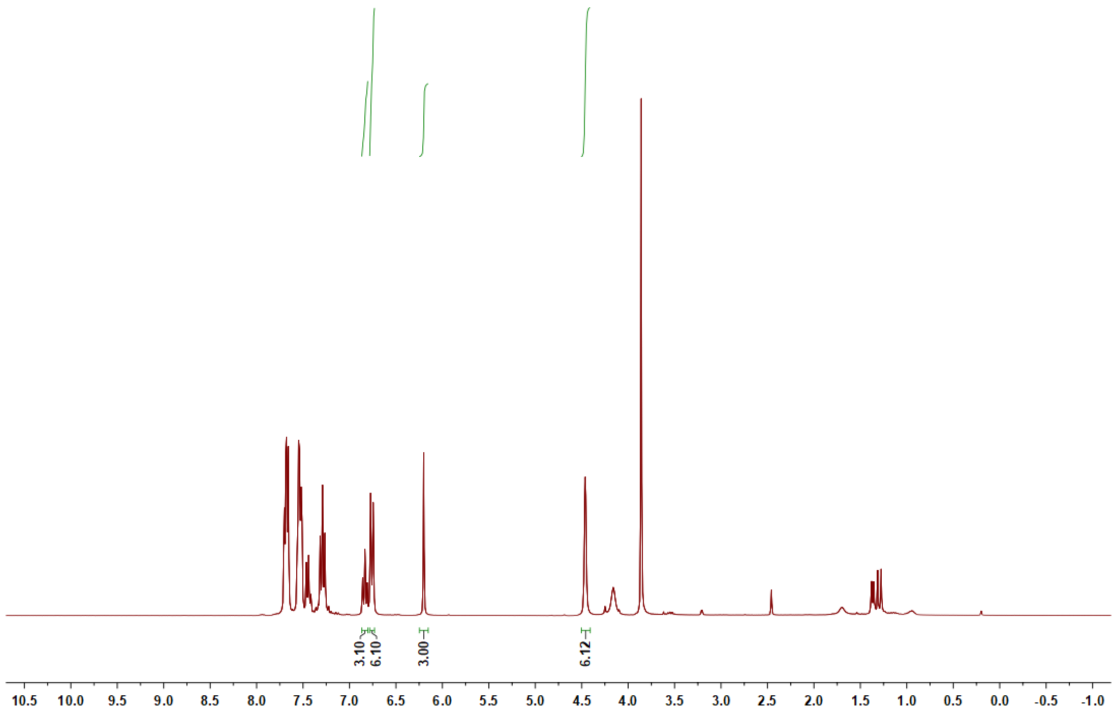

Figure S93. ^{1}H NMR (300 MHz, $CDCl_3$) spectrum of **2e**

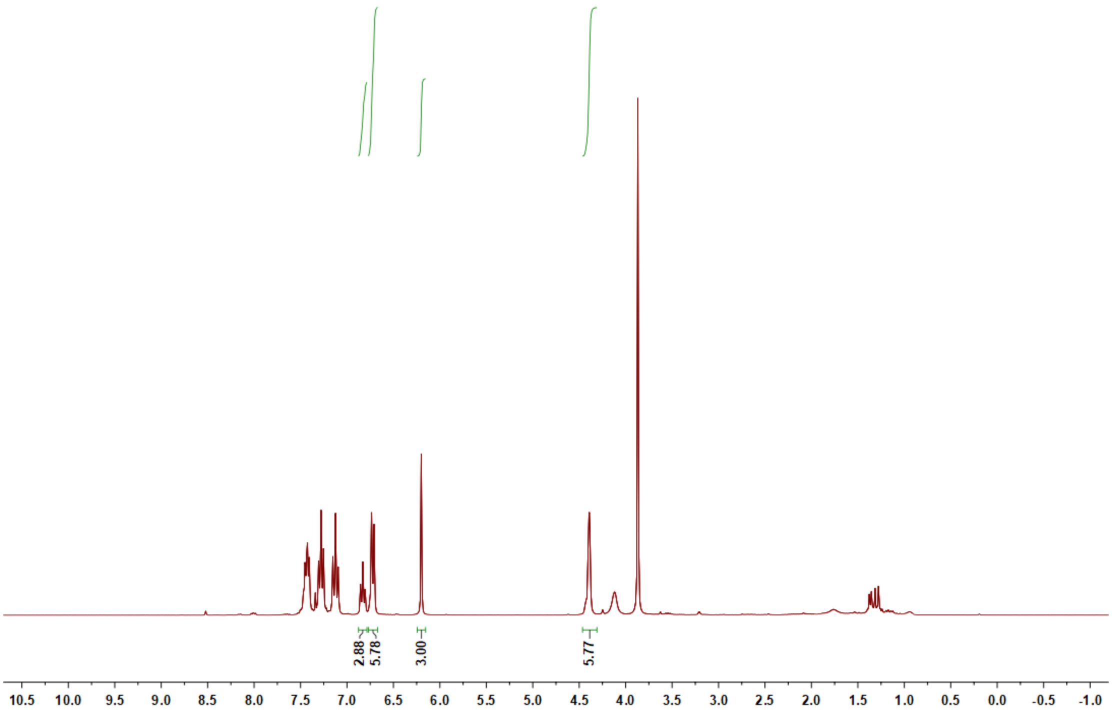

Figure S94. ^{1}H NMR (300 MHz, $CDCl_3$) spectrum of **2f**

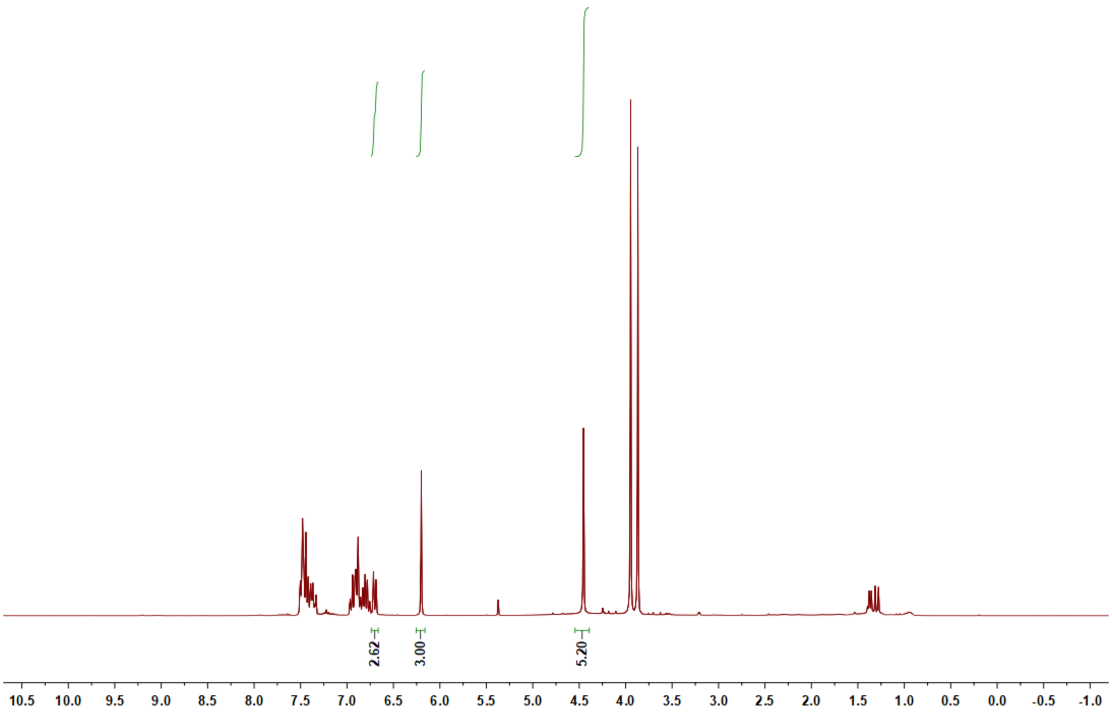

Figure S95. ^{1}H NMR (300 MHz, $CDCl_3$) spectrum of **2g**

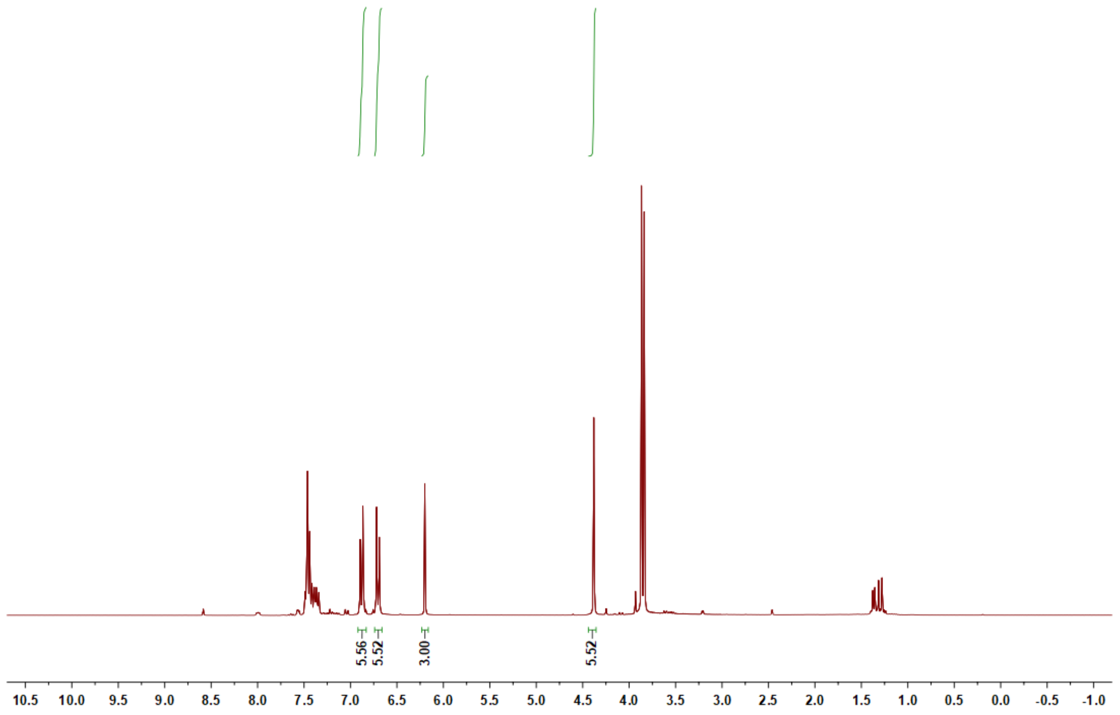

Figure S96. ^{1}H NMR (300 MHz, $CDCl_3$) spectrum of **2h**

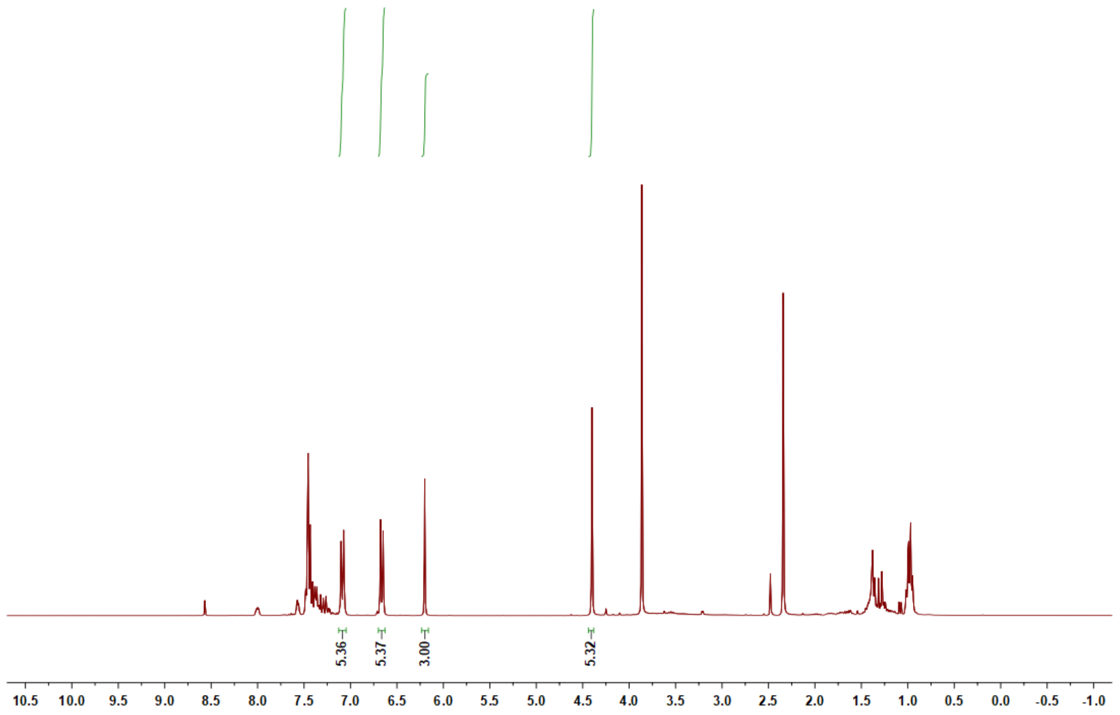

Figure S97. ^{1}H NMR (300 MHz, $CDCl_3$) spectrum of **2i**

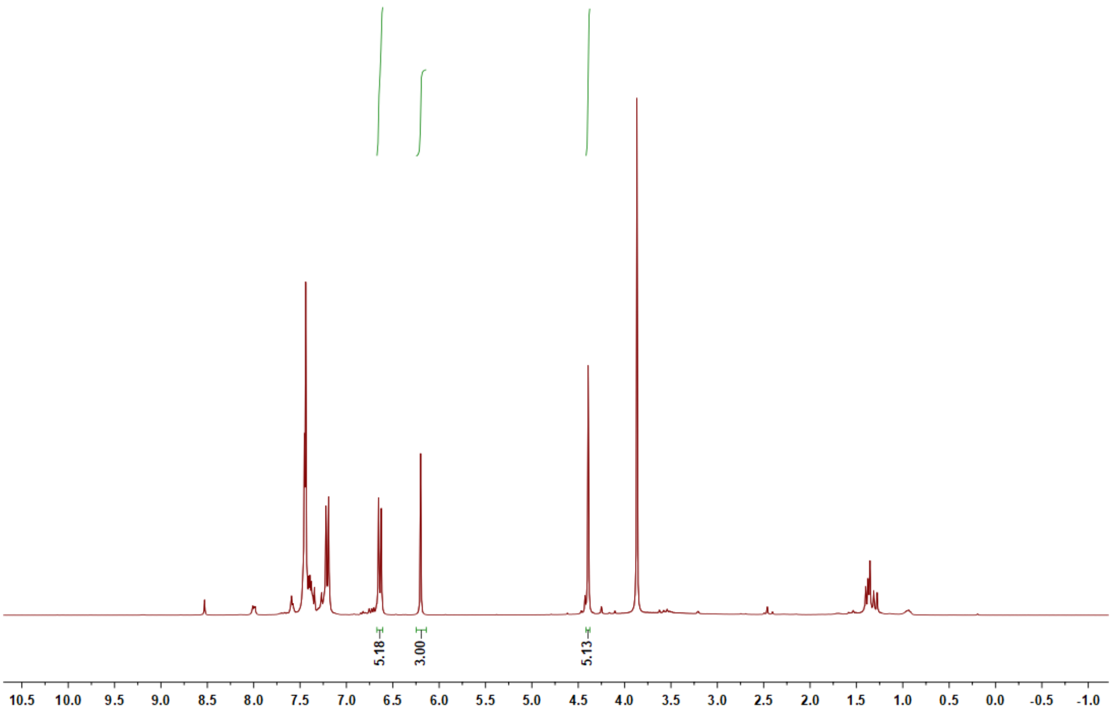

Figure S98. ^{1}H NMR (300 MHz, $CDCl_3$) spectrum of **2j**

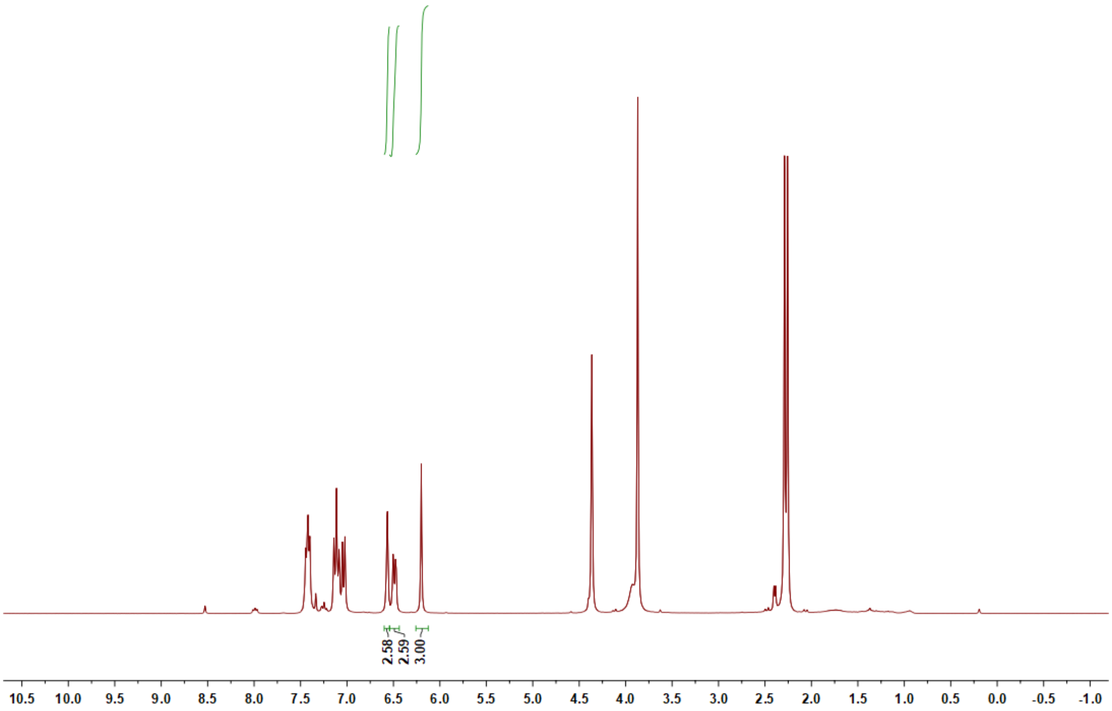

Figure S99. ^{1}H NMR (300 MHz, $CDCl_3$) spectrum of **2k**

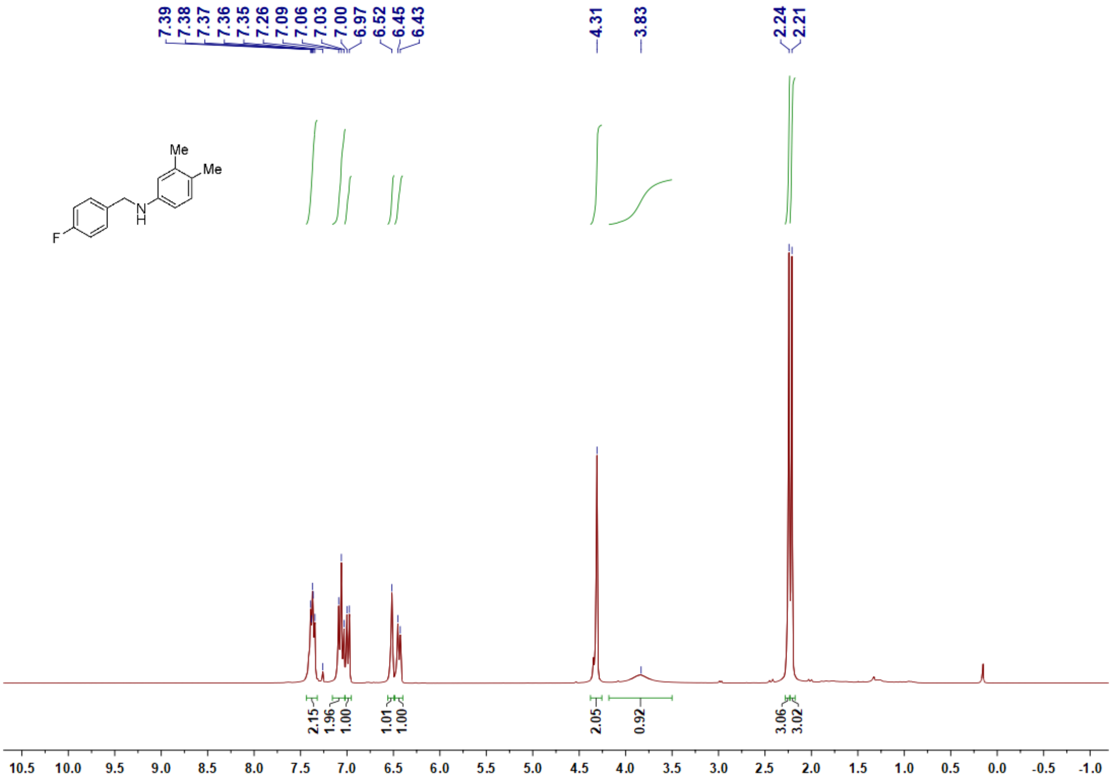

Figure S100. ^{1}H NMR (300 MHz, $CDCl_3$) spectrum of **2k** (after isolation)

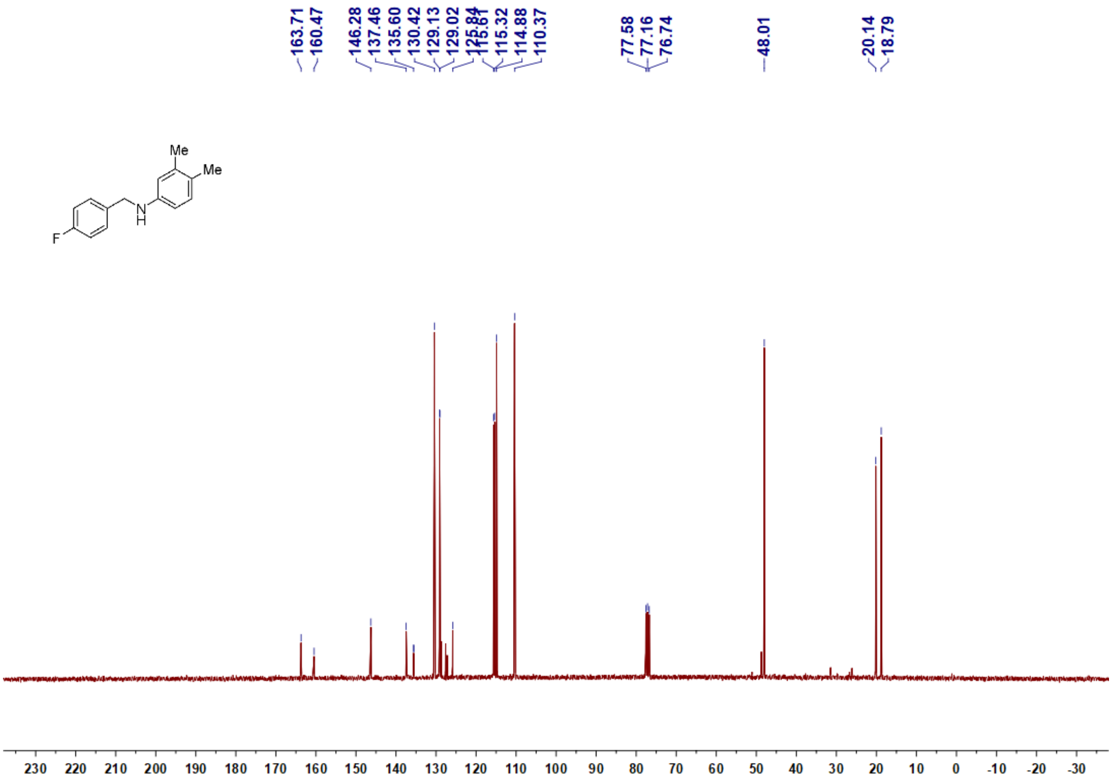

Figure S101. ^{13}C NMR (75 MHz, $CDCl_3$) spectrum of **2k** (after isolation)

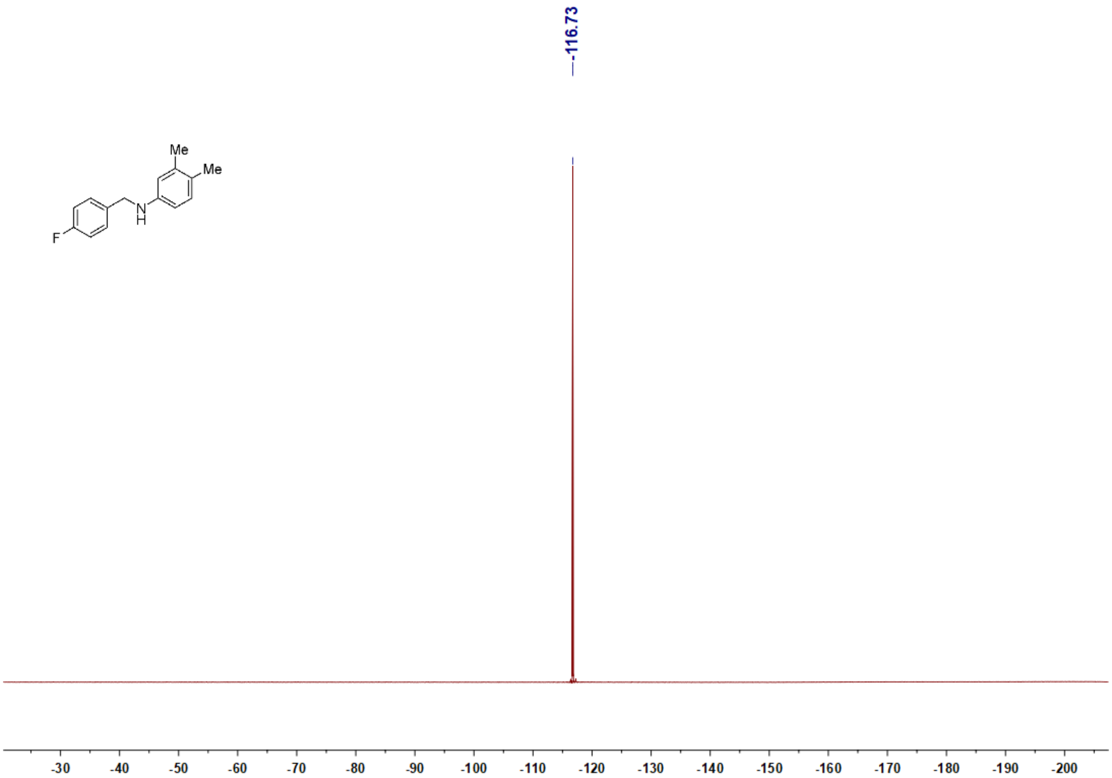

Figure S102. ^{19}F NMR (282 MHz, $CDCl_3$) spectrum of **2k** (after isolation)

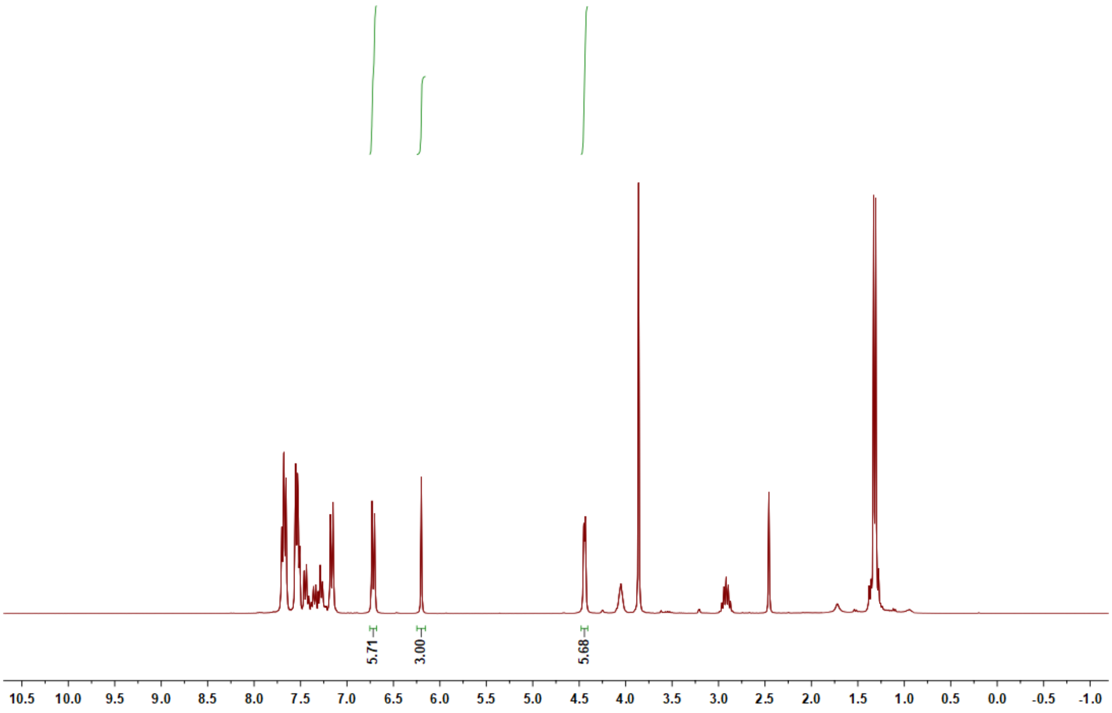

Figure S103. ^{1}H NMR (300 MHz, $CDCl_3$) spectrum of **2l**

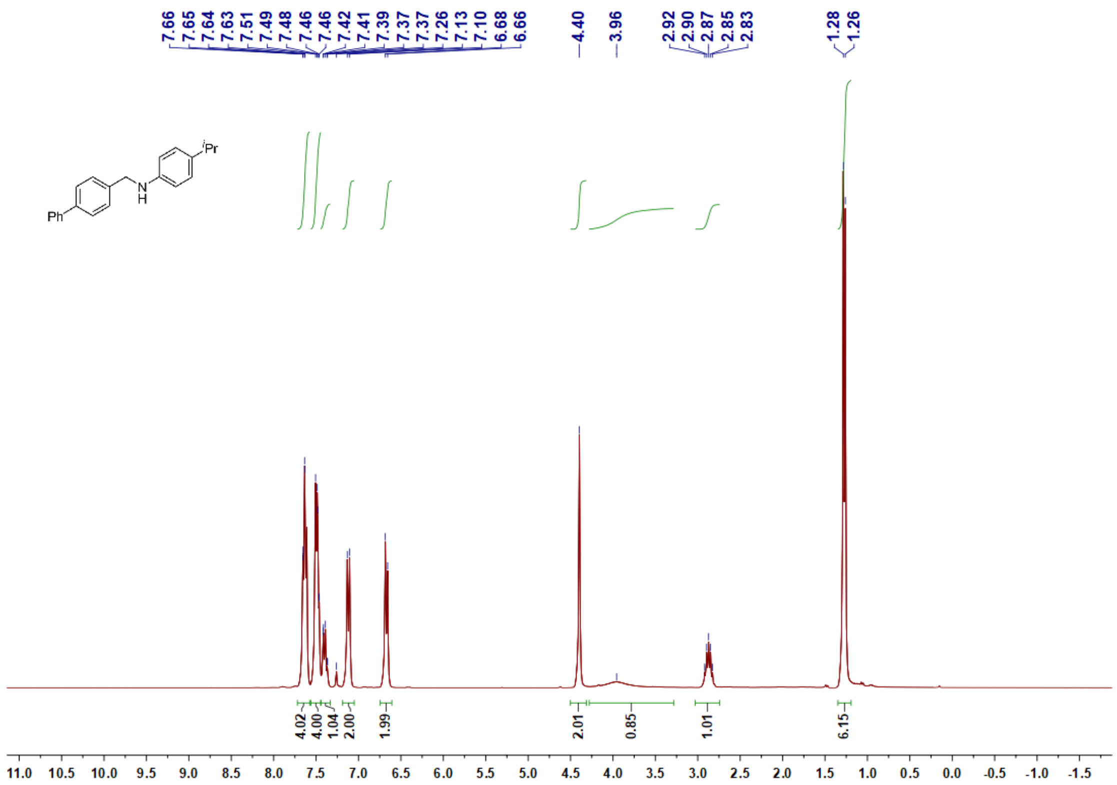

Figure S104. ^{1}H NMR (300 MHz, $CDCl_3$) spectrum of **2l** (after isolation)

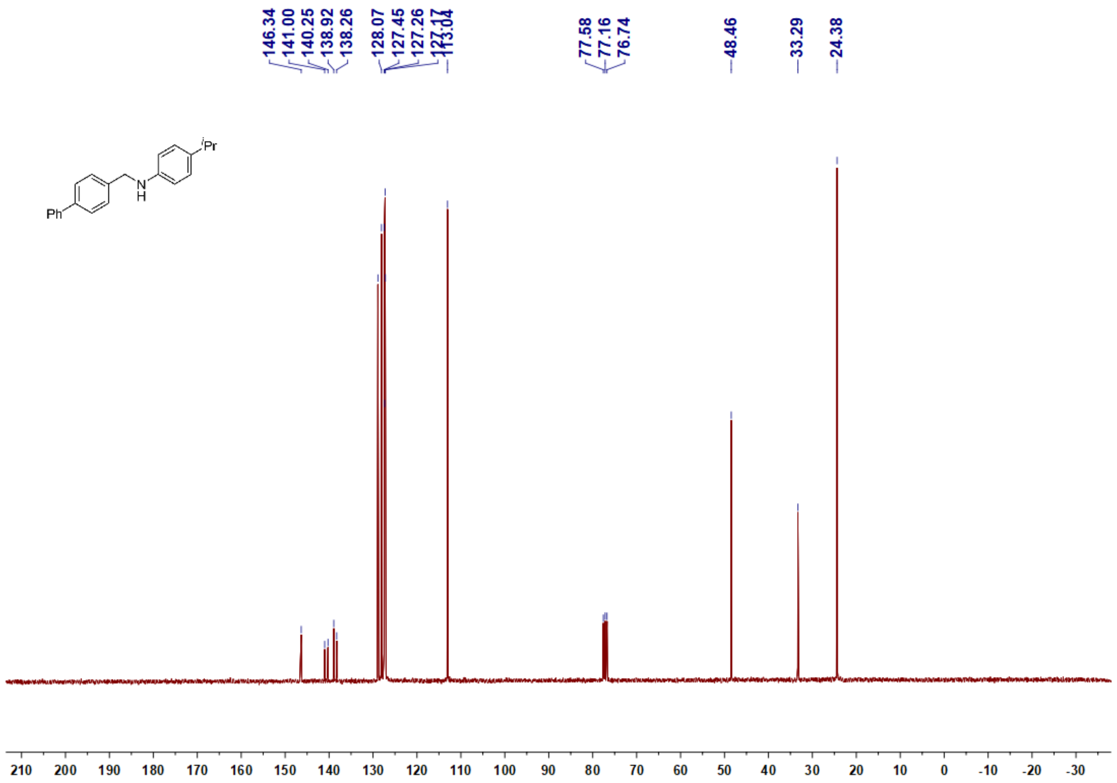

Figure S105. ^{13}C NMR (75 MHz, $CDCl_3$) spectrum of **2l** (after isolation)

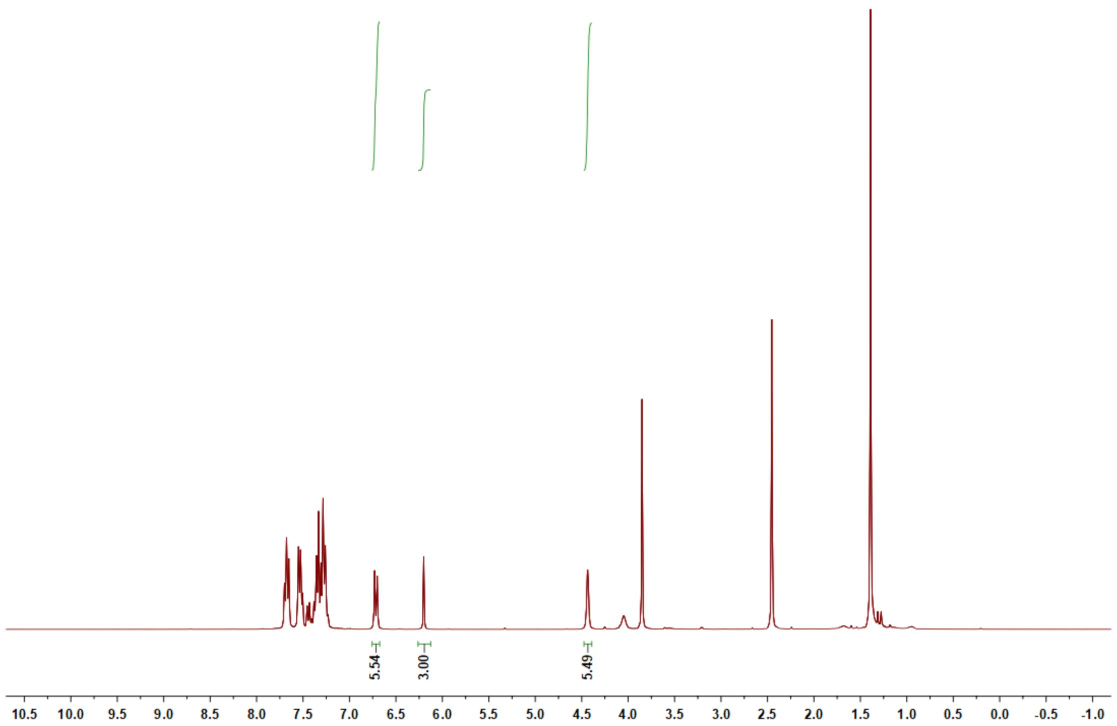

Figure S106. ^{1}H NMR (300 MHz, $CDCl_3$) spectrum of **2m**

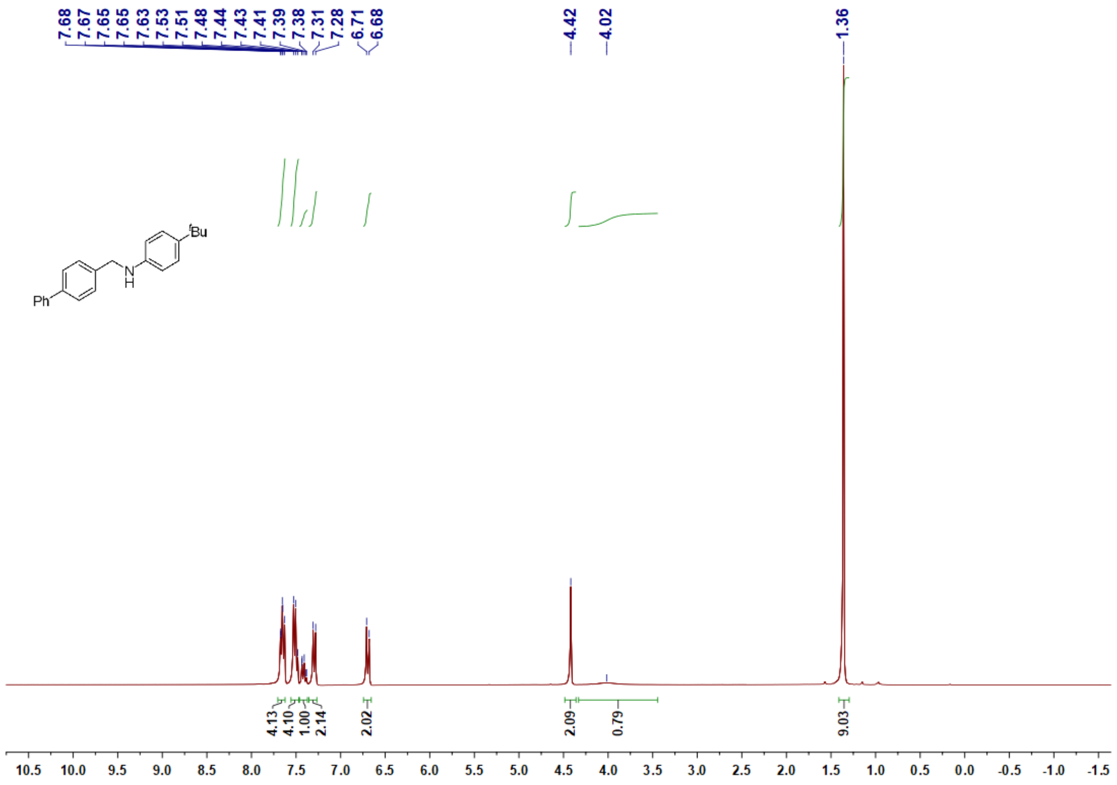

Figure S107. ^{1}H NMR (300 MHz, $CDCl_3$) spectrum of **2m** (after isolation)

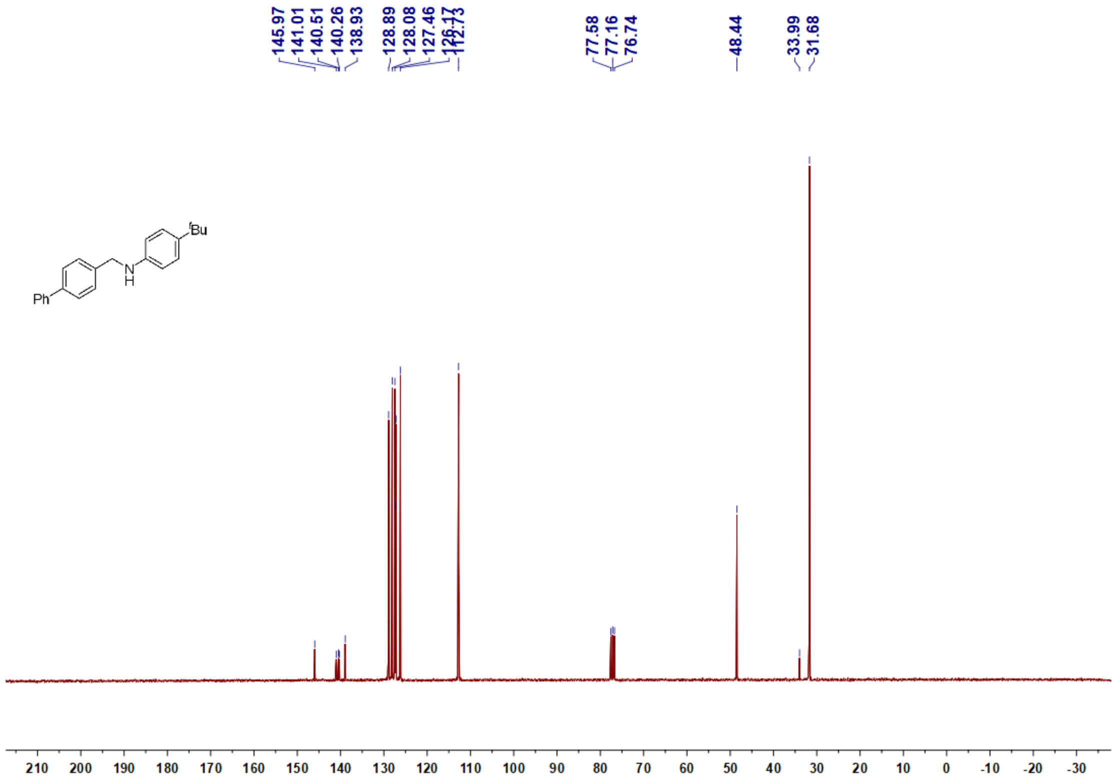

Figure S108. ^{13}C NMR (75 MHz, $CDCl_3$) spectrum of **2m** (after isolation)

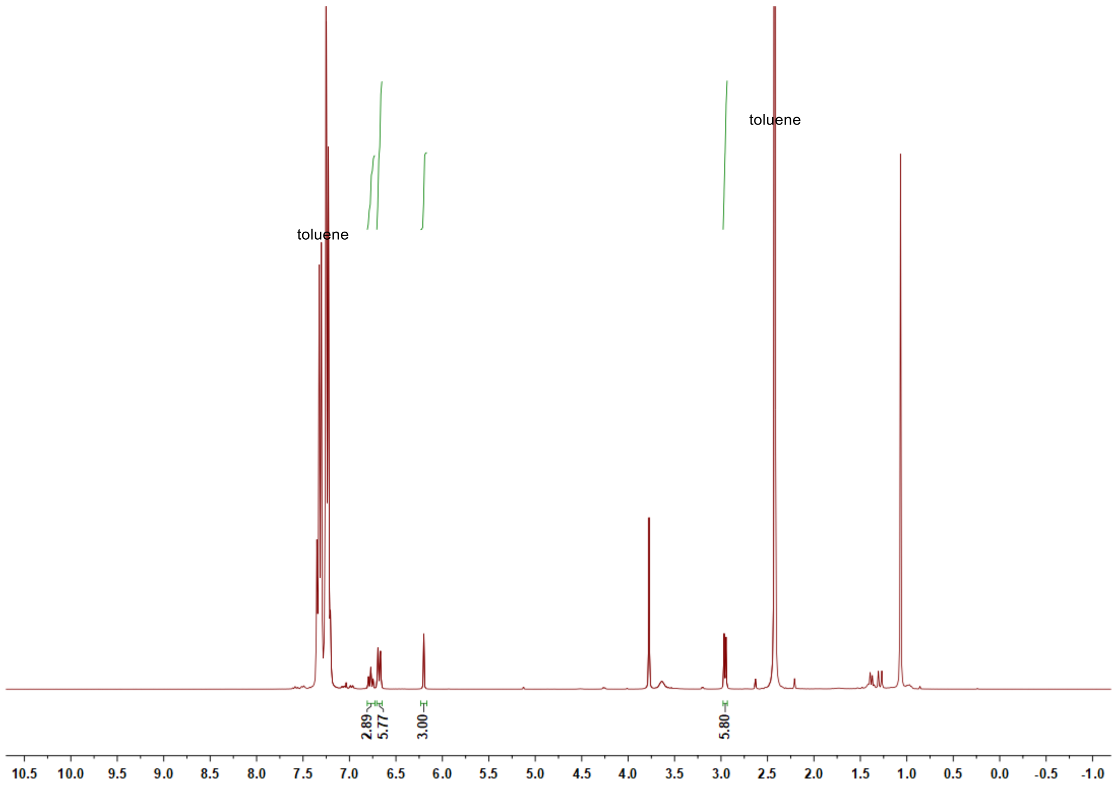

Figure S109. ^{1}H NMR (300 MHz, $CDCl_3$) spectrum of **2n**

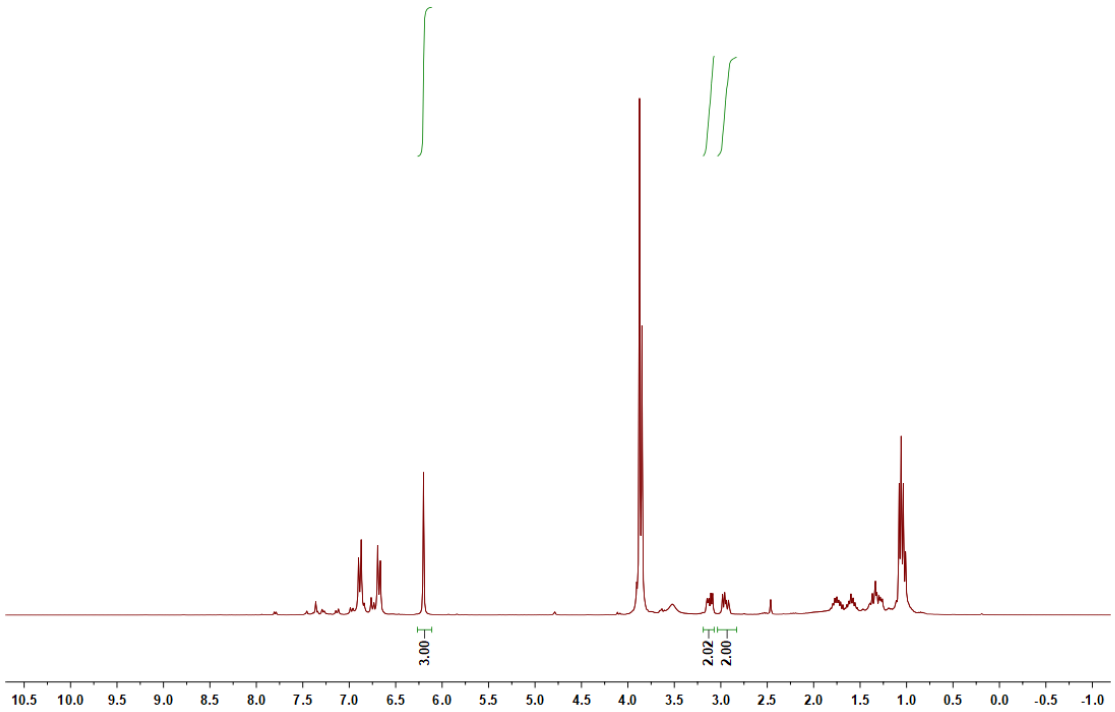

Figure S110. ^{1}H NMR (300 MHz, $CDCl_3$) spectrum of **2o**

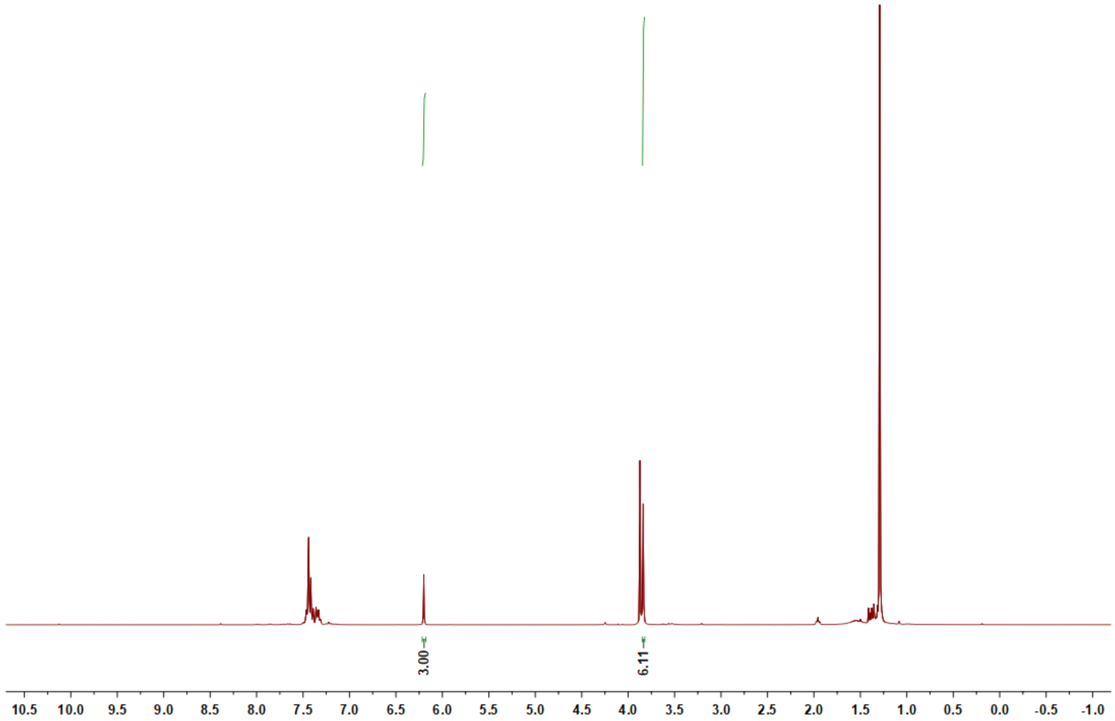

Figure S111. ^{1}H NMR (300 MHz, $CDCl_3$) spectrum of **2p**

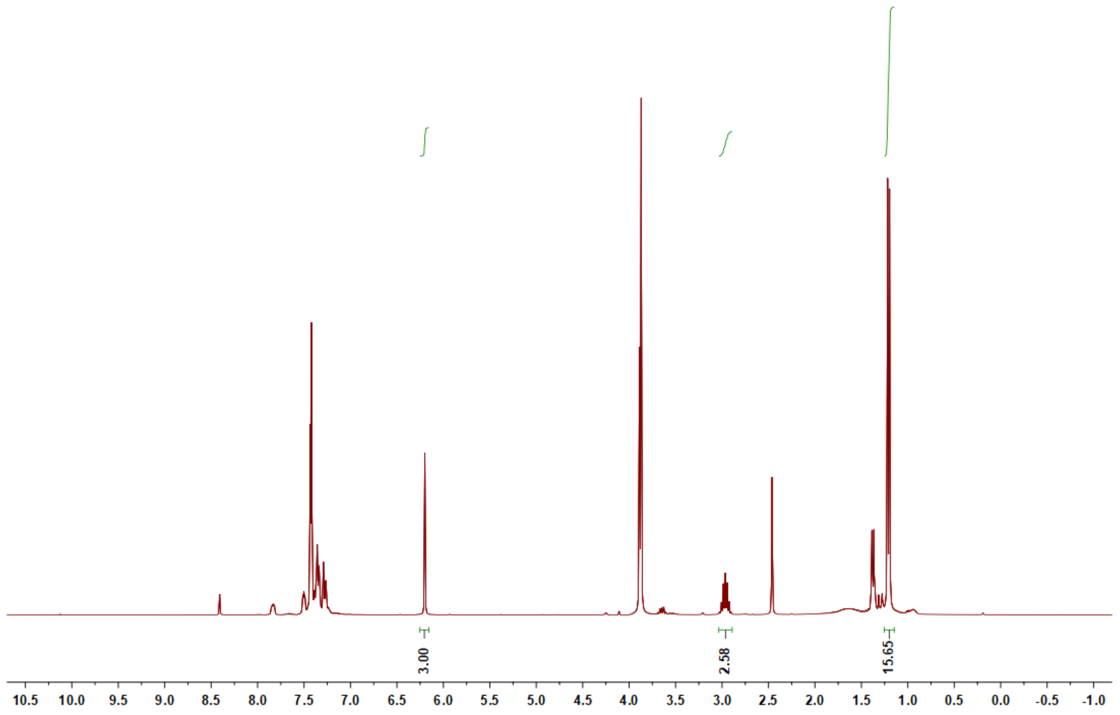

Figure S112. ^{1}H NMR (300 MHz, $CDCl_3$) spectrum of **2q**

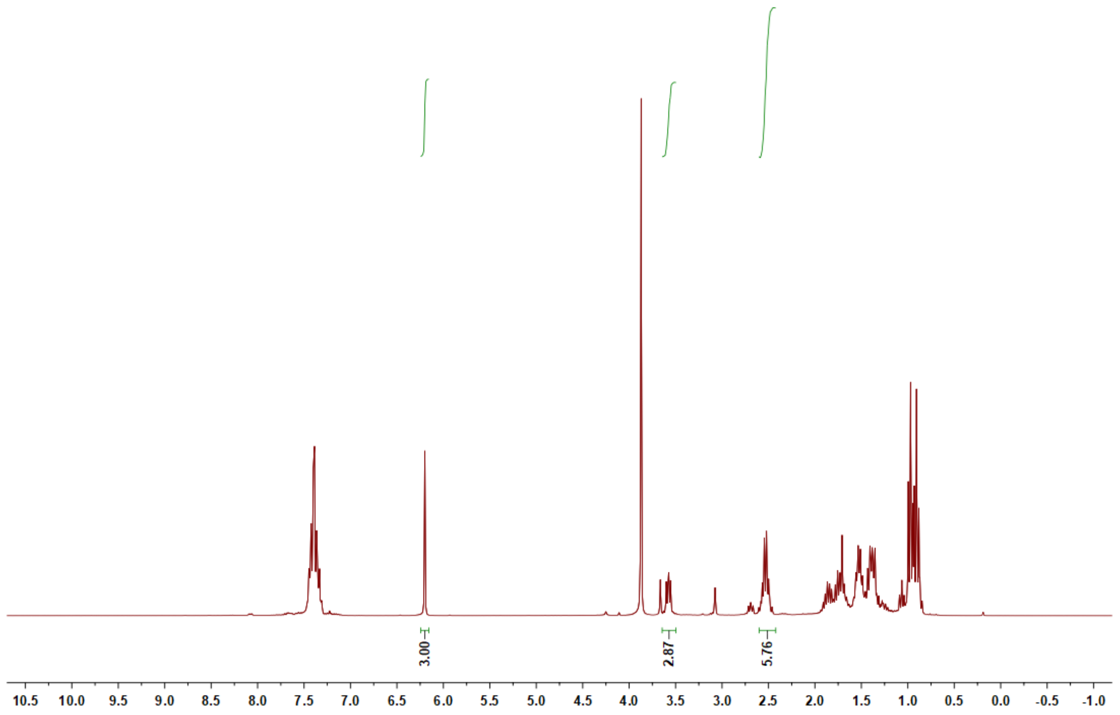

Figure S113. ^{1}H NMR (300 MHz, $CDCl_3$) spectrum of **2r**

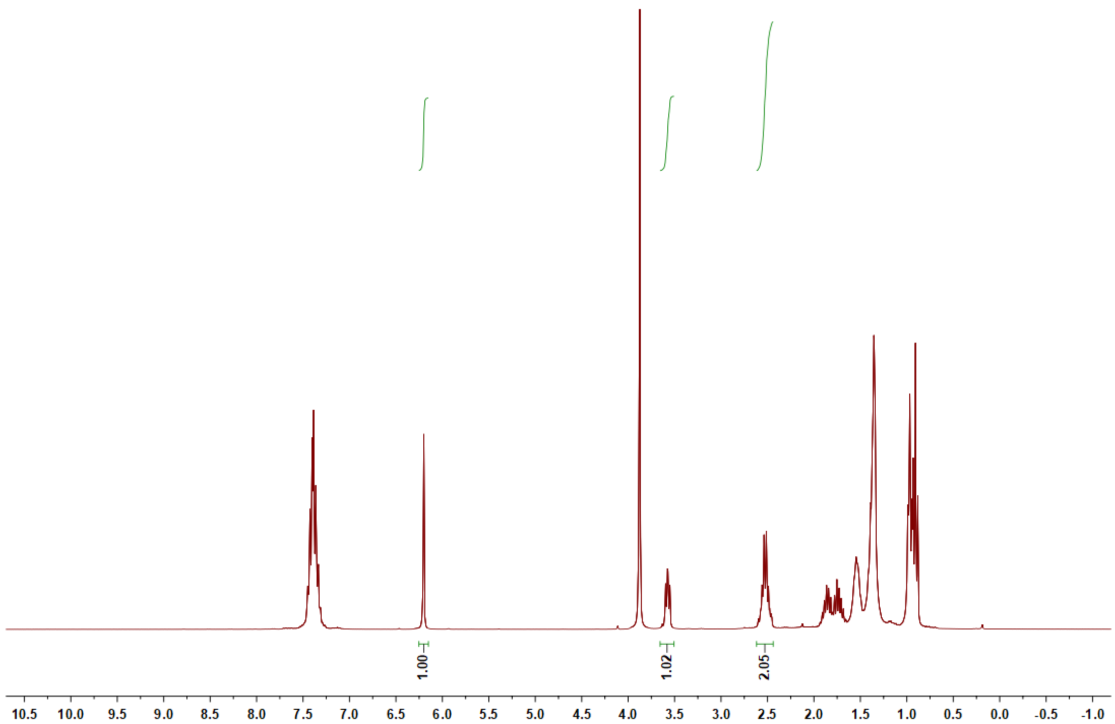

Figure S114. ^{1}H NMR (300 MHz, $CDCl_3$) spectrum of **2s**

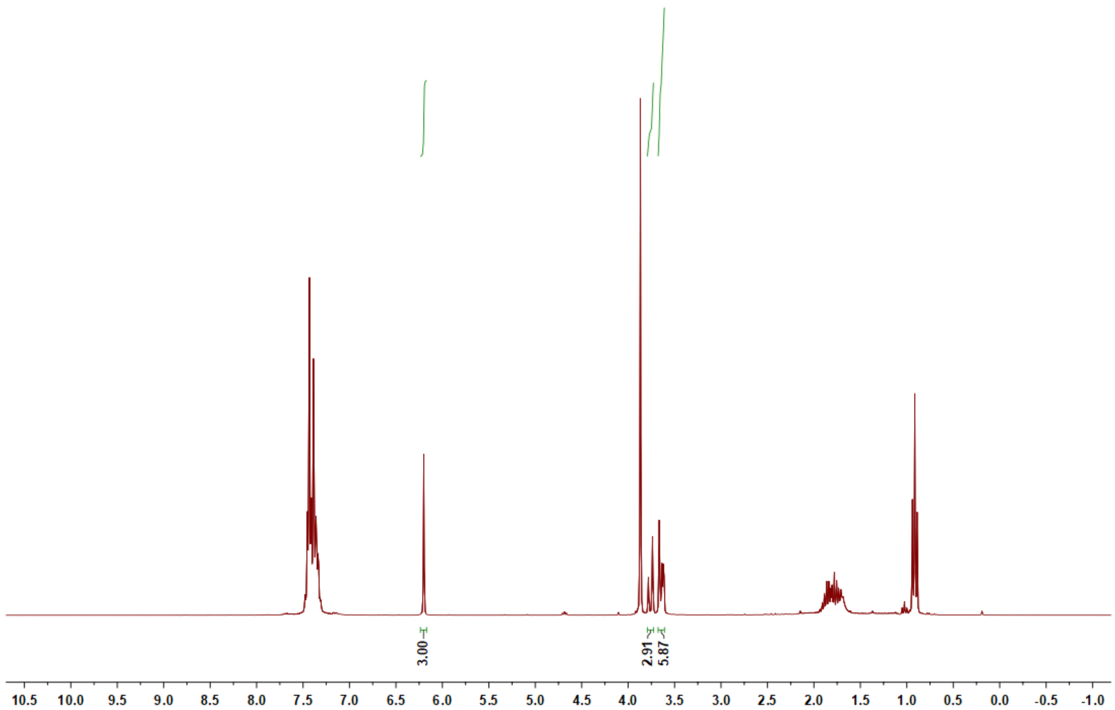

Figure S115. ^{1}H NMR (300 MHz, $CDCl_3$) spectrum of **2t**

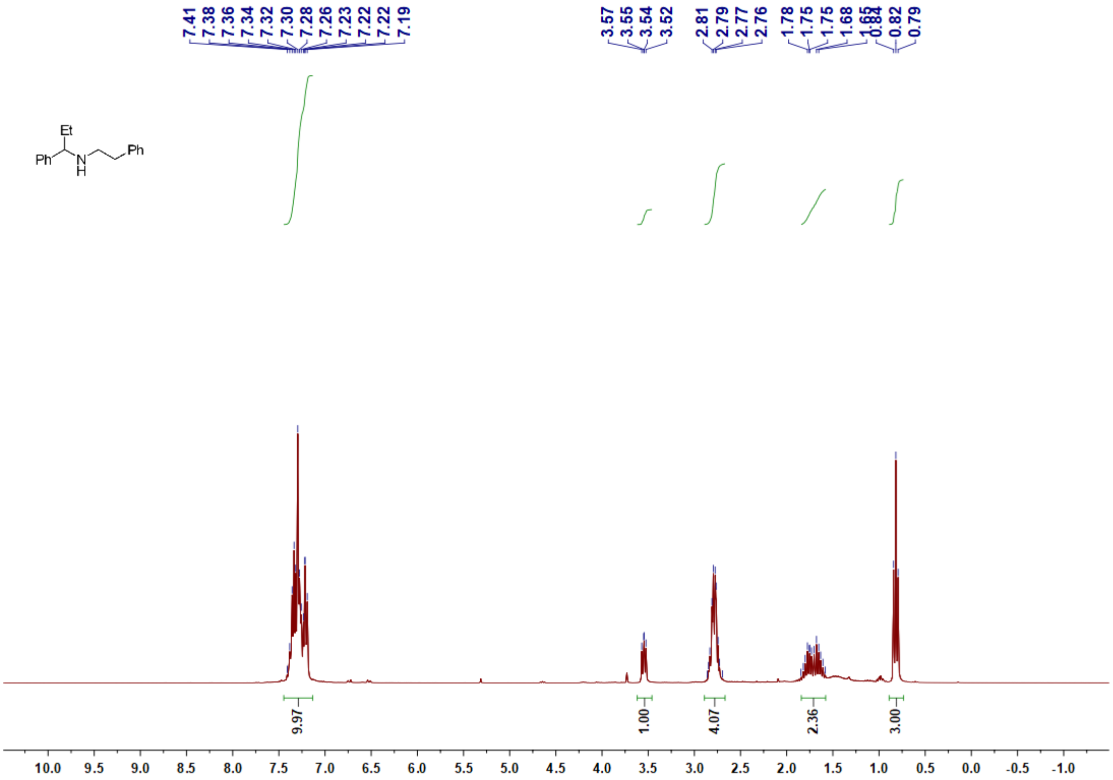

Figure S116. ^{1}H NMR (300 MHz, $CDCl_3$) spectrum of **2u**

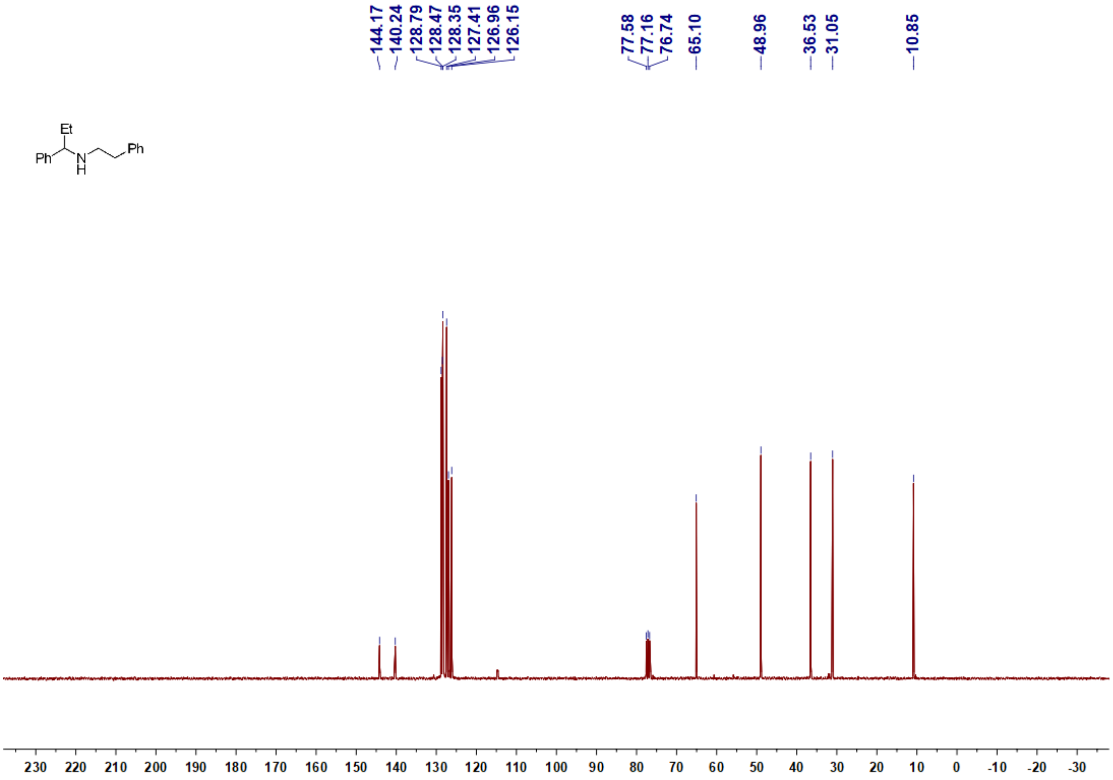

Figure S117. ^{13}C NMR (75 MHz, $CDCl_3$) spectrum of **2u**

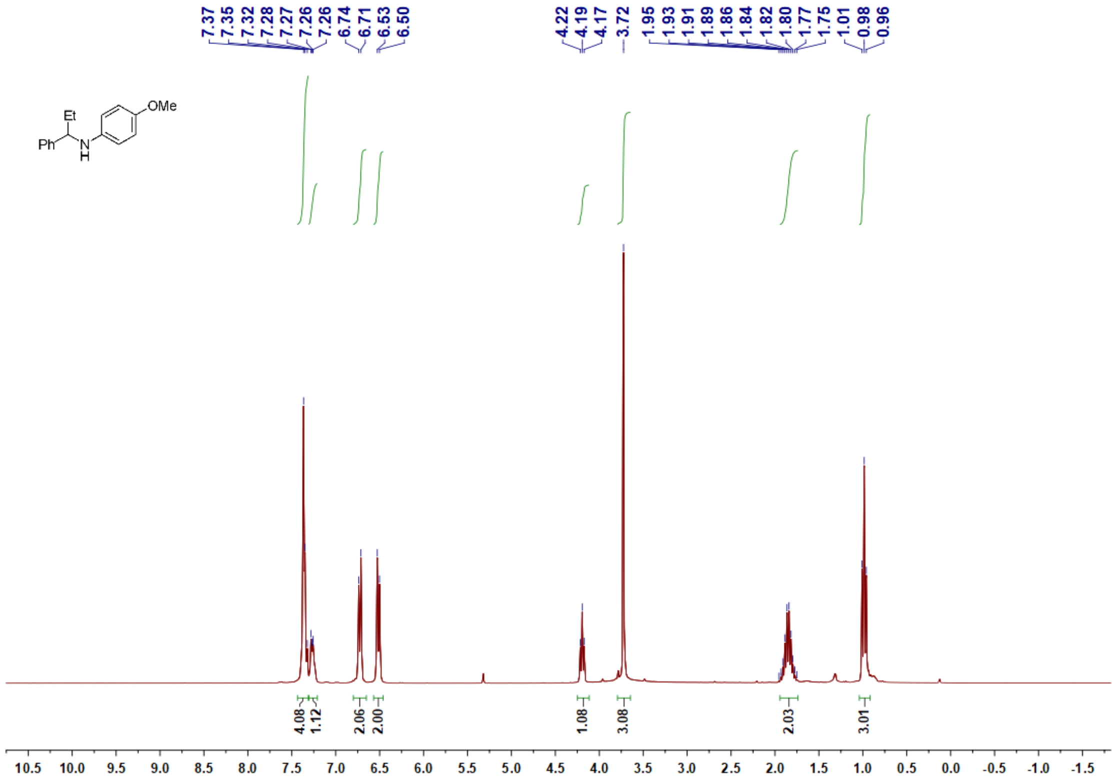

Figure S118. ^{1}H NMR (300 MHz, $CDCl_3$) spectrum of **2v**

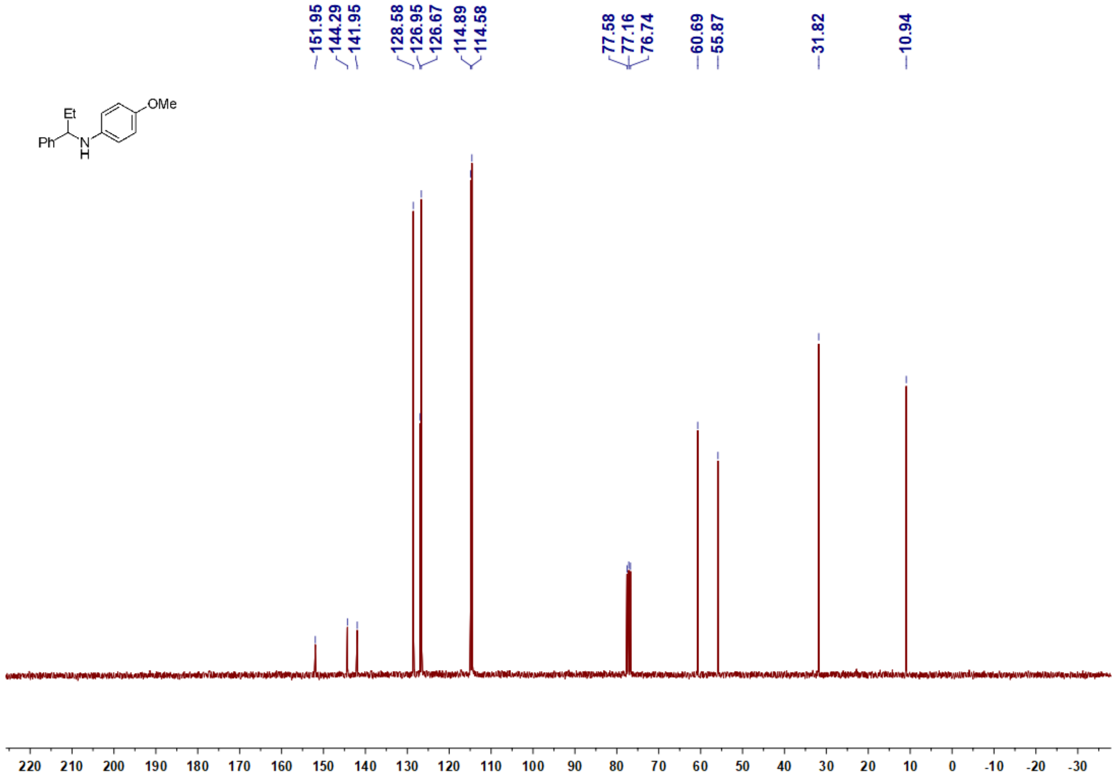

Figure S119. ^{13}C NMR (75 MHz, $CDCl_3$) spectrum of **2v**

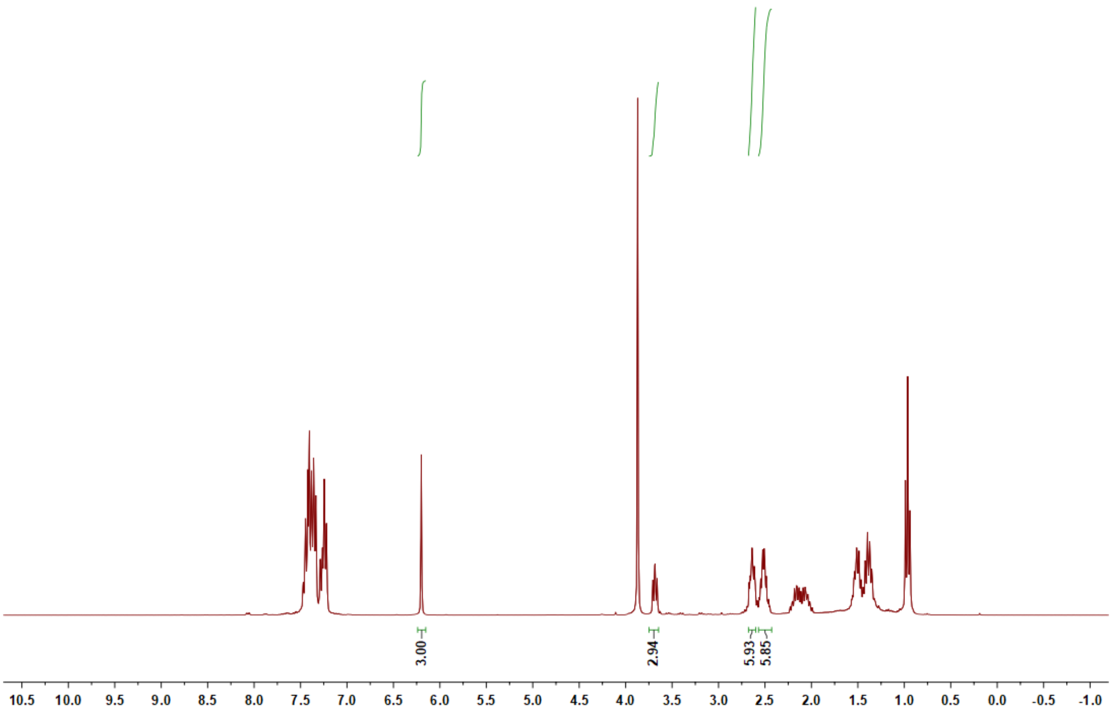

Figure S120. ^{1}H NMR (300 MHz, $CDCl_3$) spectrum of **2w**

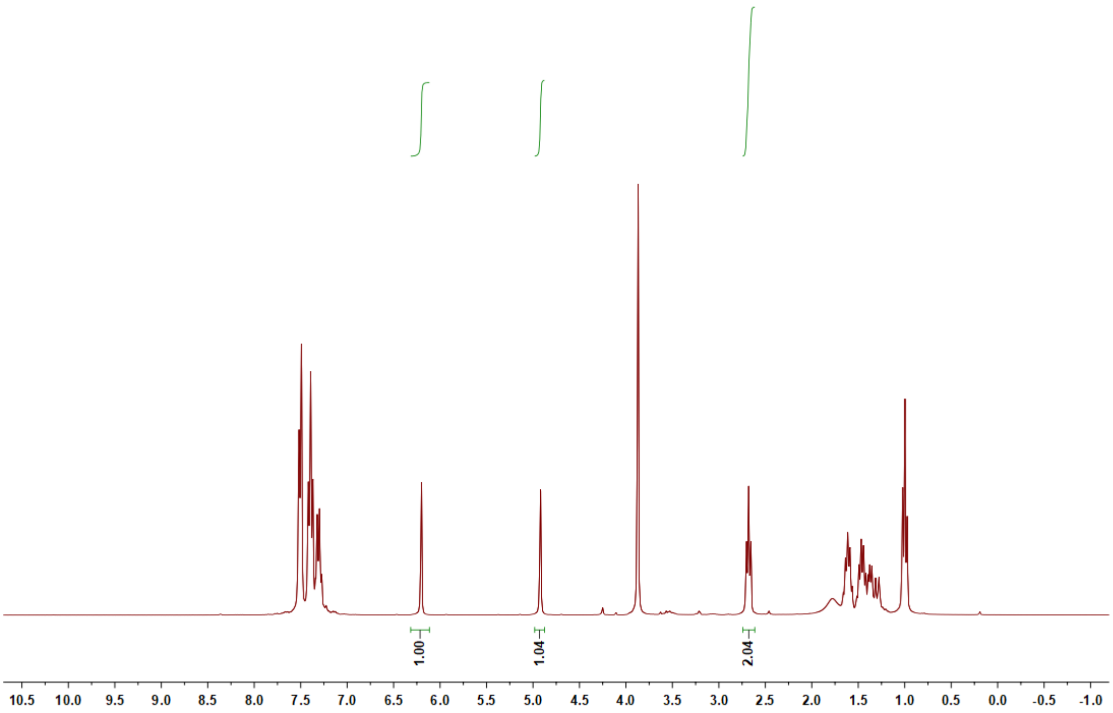

Figure S121. ^{1}H NMR (300 MHz, $CDCl_3$) spectrum of **2x**

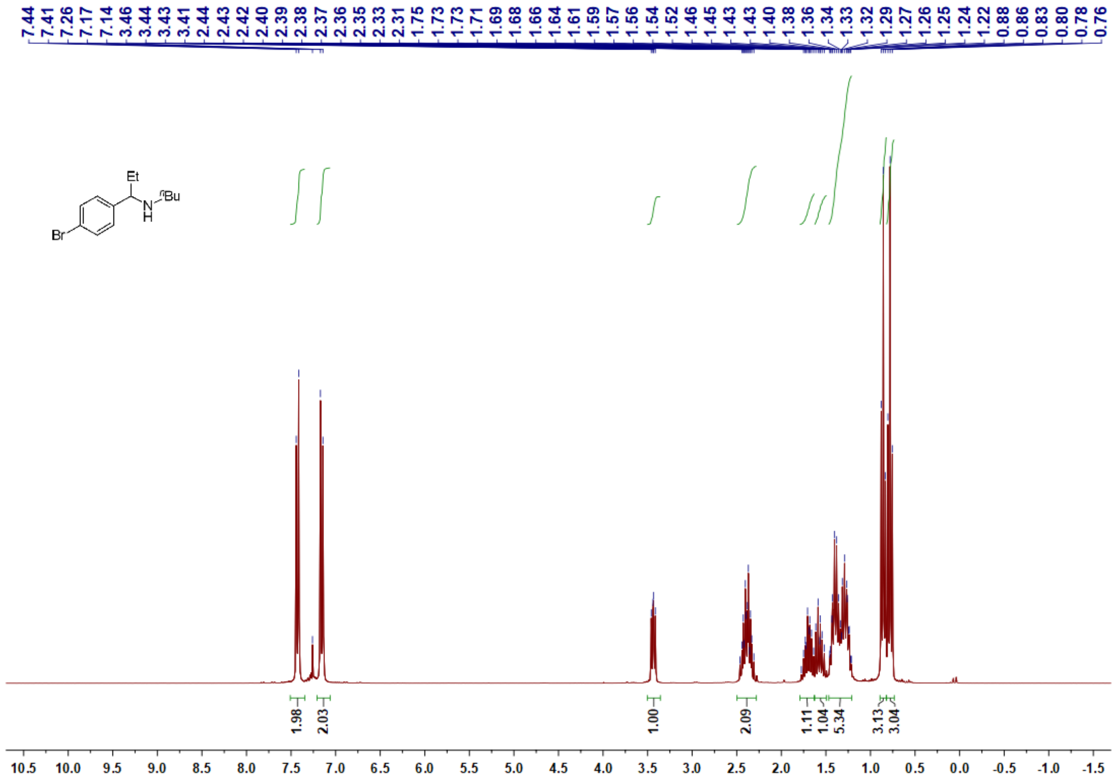

Figure S122. ^{1}H NMR (300 MHz, $CDCl_3$) spectrum of **2y**

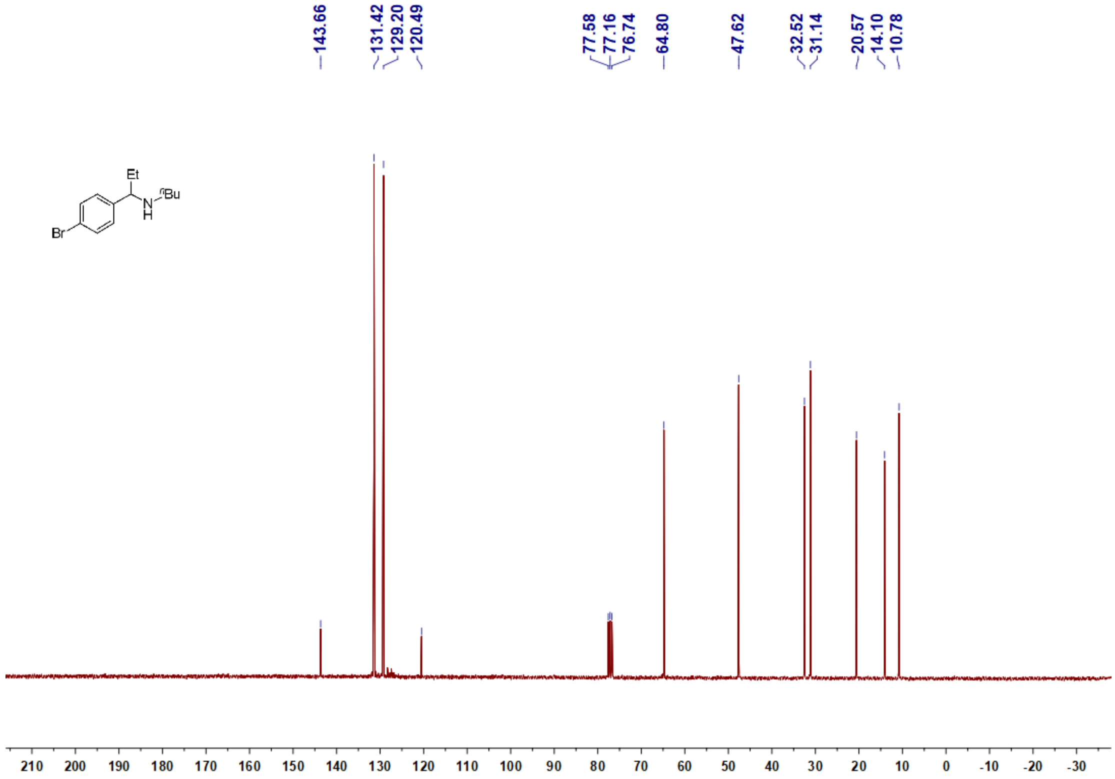

Figure S123. ^{13}C NMR (75 MHz, $CDCl_3$) spectrum of **2y**

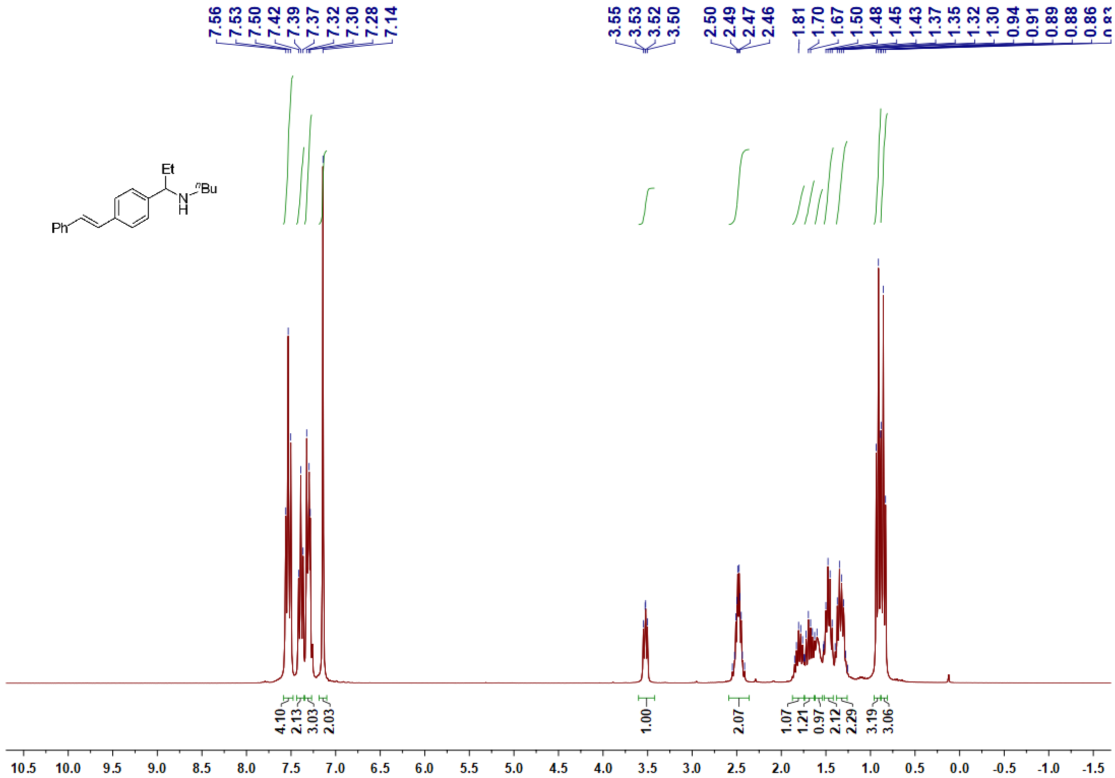

Figure S124. ^{1}H NMR (300 MHz, $CDCl_3$) spectrum of **2z**

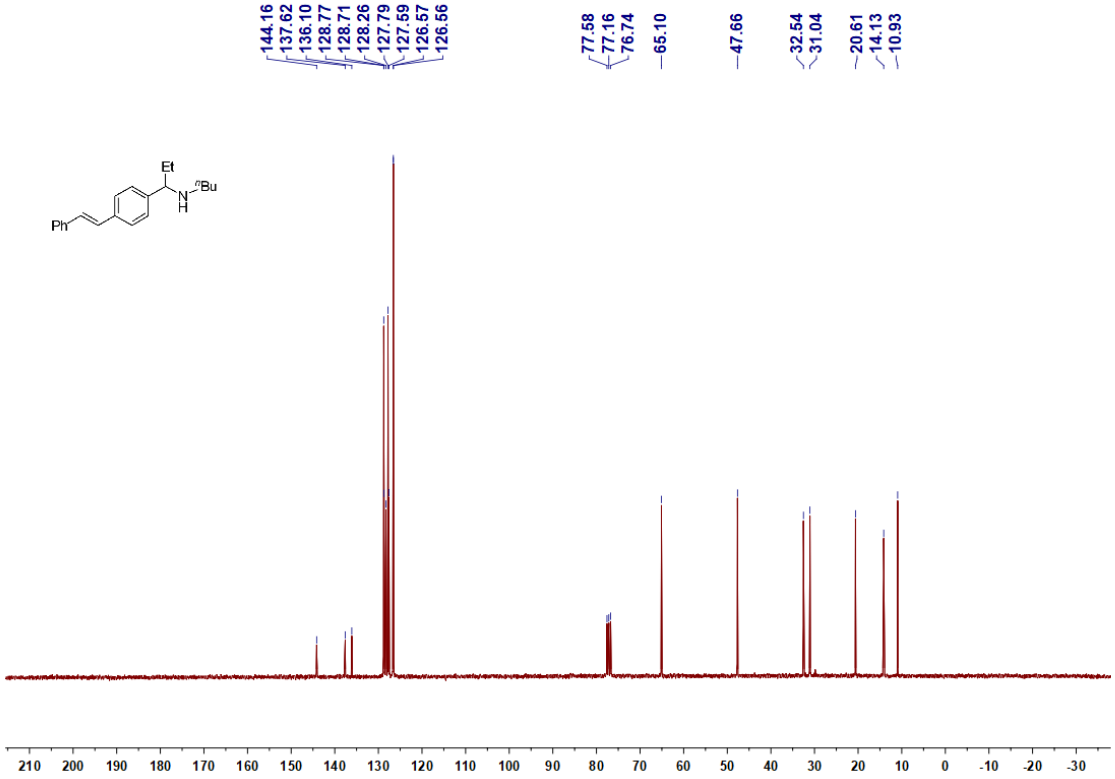

Figure S125. ^{13}C NMR (75 MHz, $CDCl_3$) spectrum of **2z**

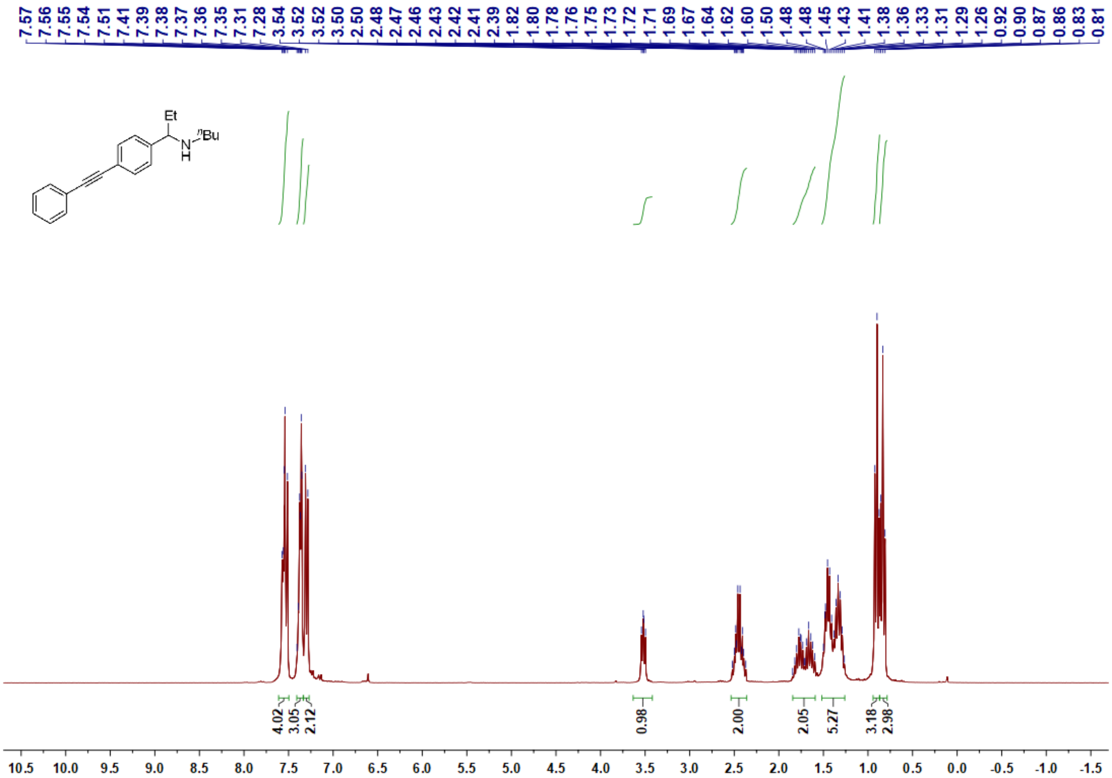

Figure S126. ^{1}H NMR (300 MHz, $CDCl_3$) spectrum of **2aa**

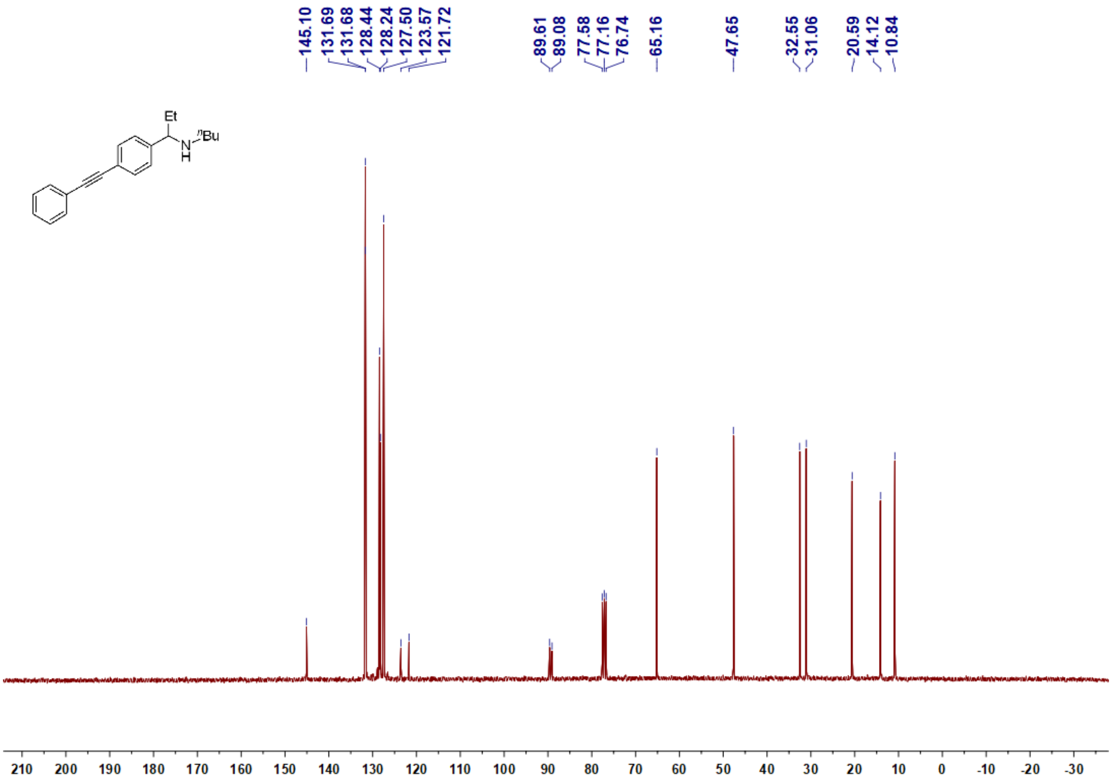

Figure S127. ^{13}C NMR (75 MHz, $CDCl_3$) spectrum of **2aa**

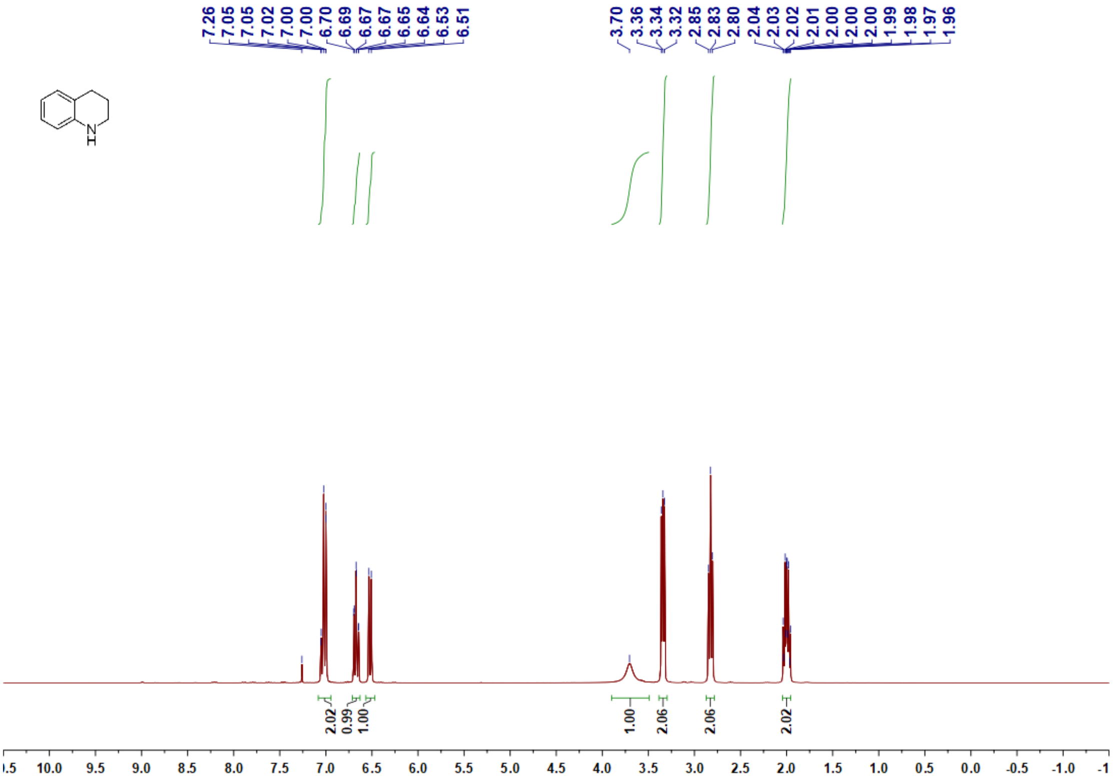

Figure S128. ^{1}H NMR (300 MHz, $CDCl_3$) spectrum of **4a**

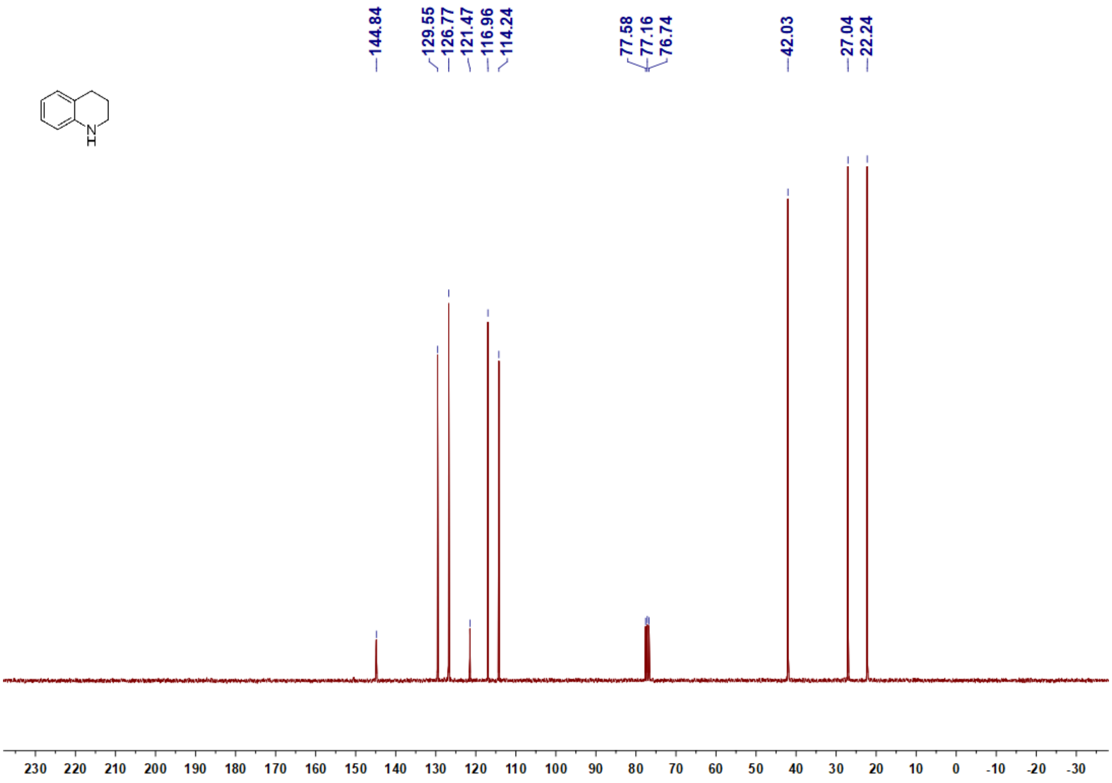

Figure S129. ^{13}C NMR (75 MHz, $CDCl_3$) spectrum of **4a**

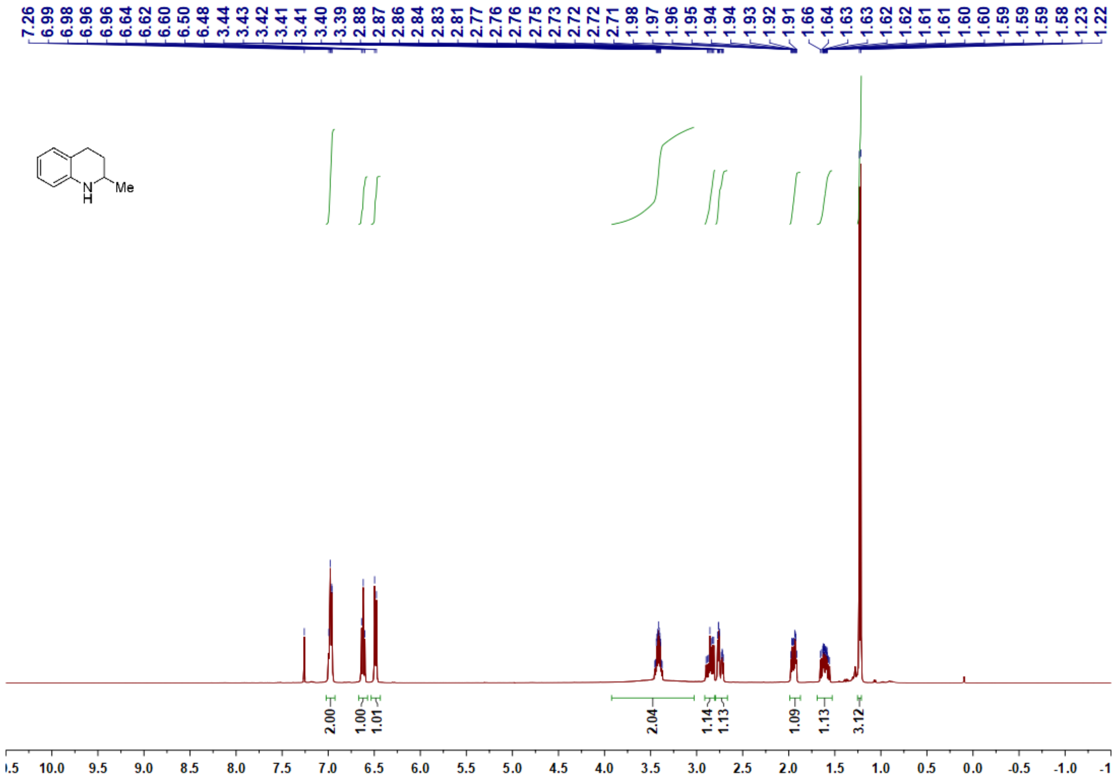

Figure S130. ^{1}H NMR (400 MHz, $CDCl_3$) spectrum of **4b**

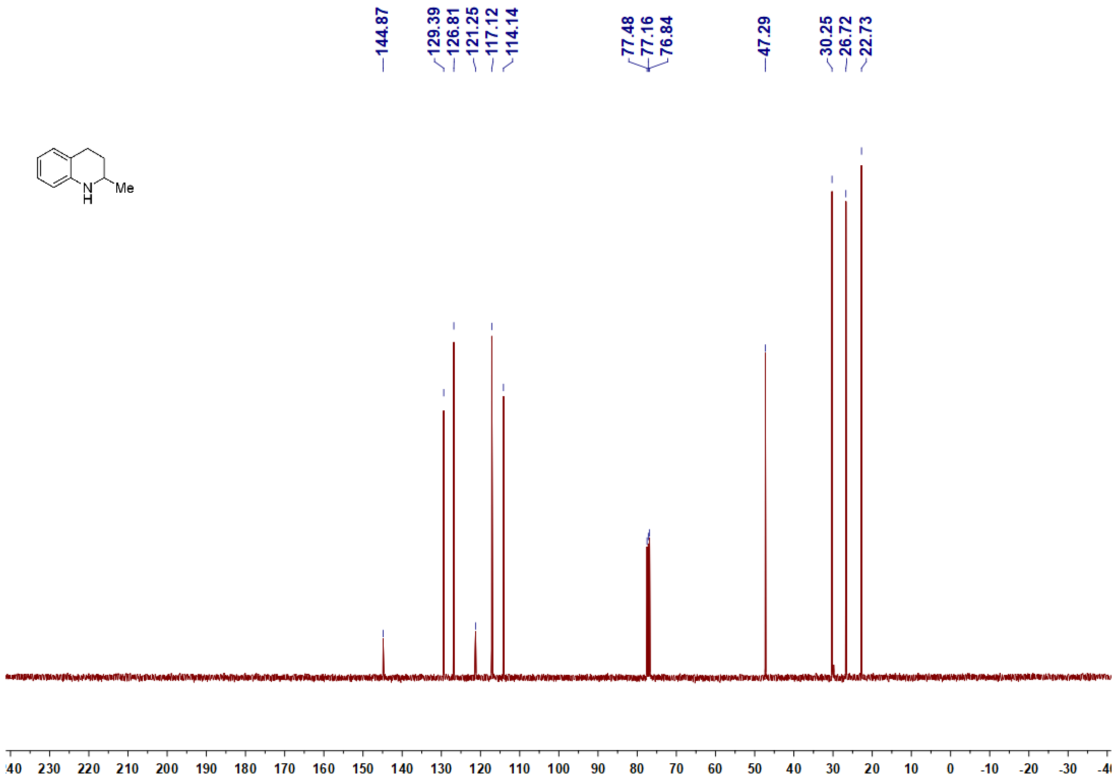

Figure S131. ^{13}C NMR (101 MHz, $CDCl_3$) spectrum of **4b**

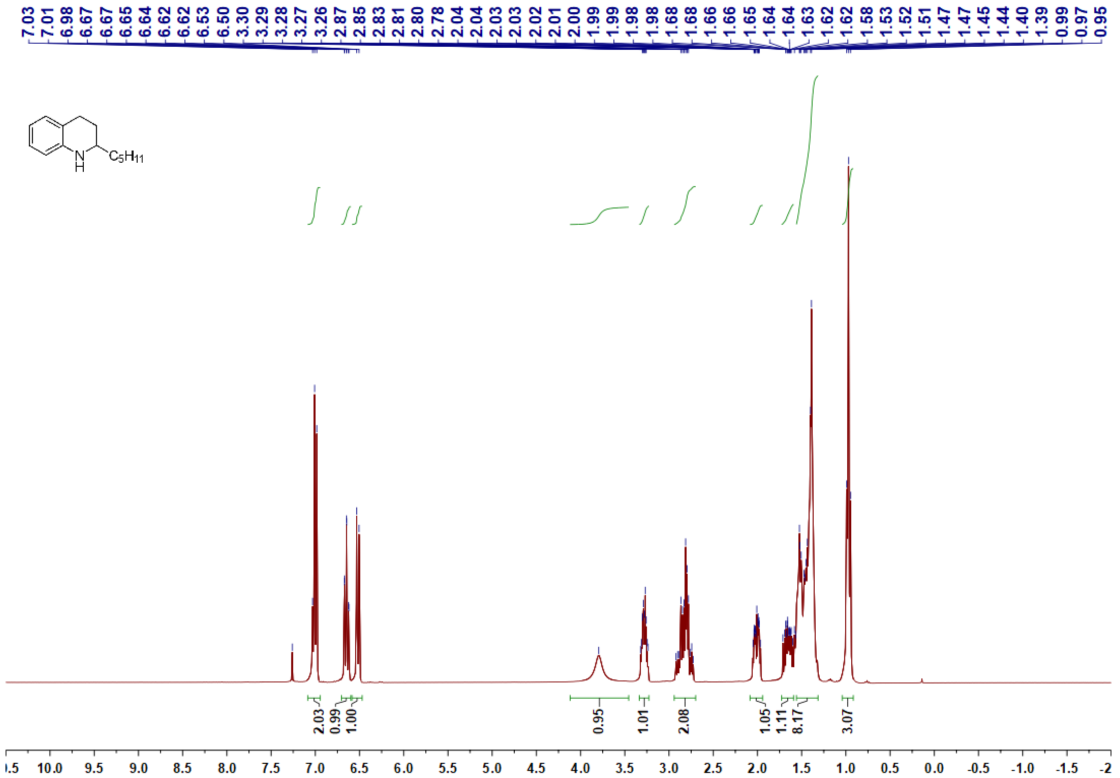

Figure S132. ^{1}H NMR (300 MHz, $CDCl_3$) spectrum of **4c**

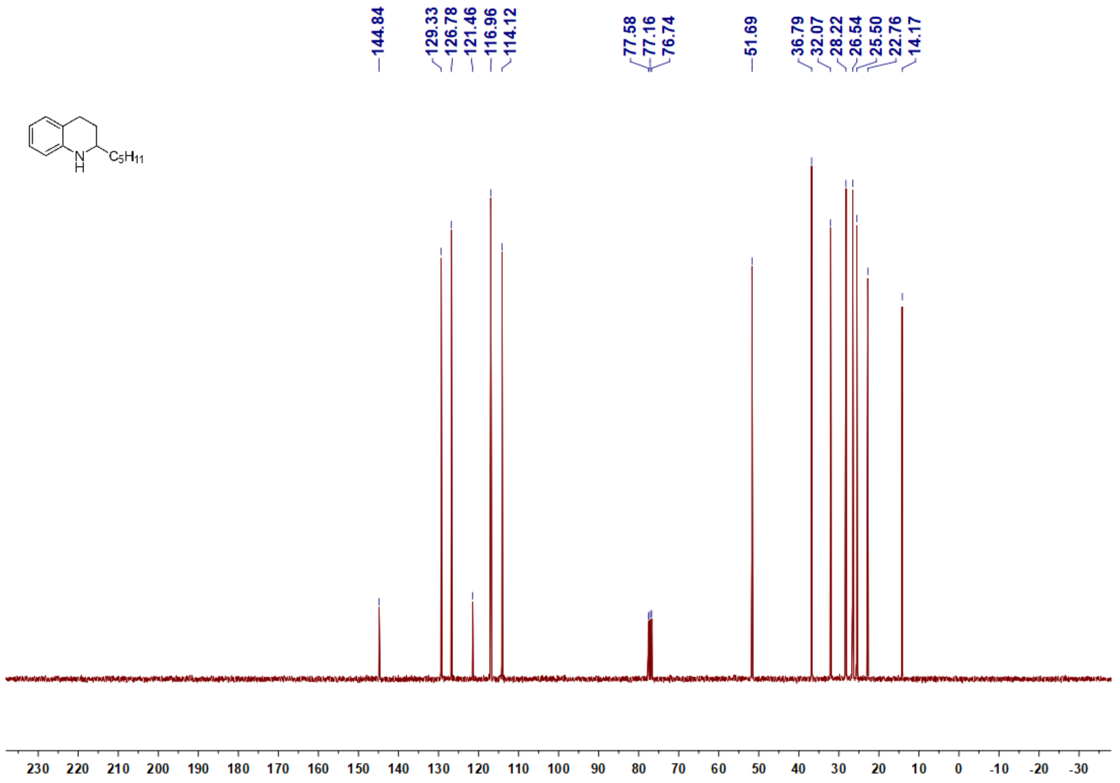

Figure S133. ^{13}C NMR (75 MHz, $CDCl_3$) spectrum of **4c**

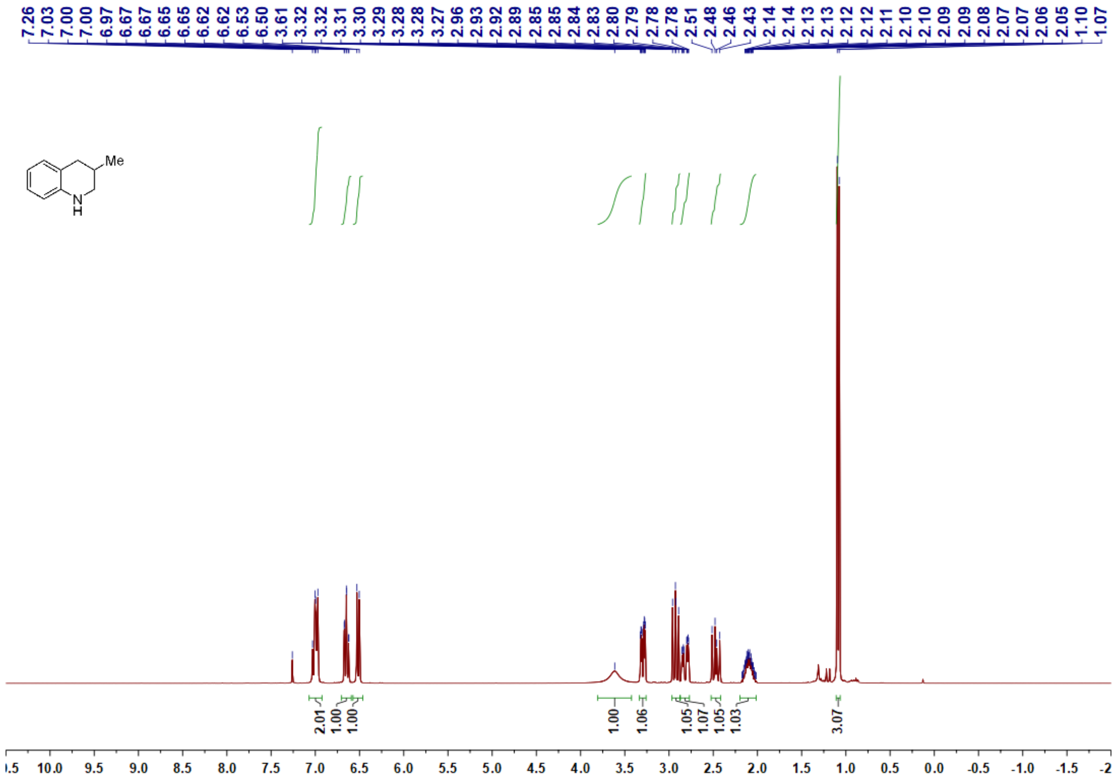

Figure S134. ^{1}H NMR (300 MHz, $CDCl_3$) spectrum of **4d**

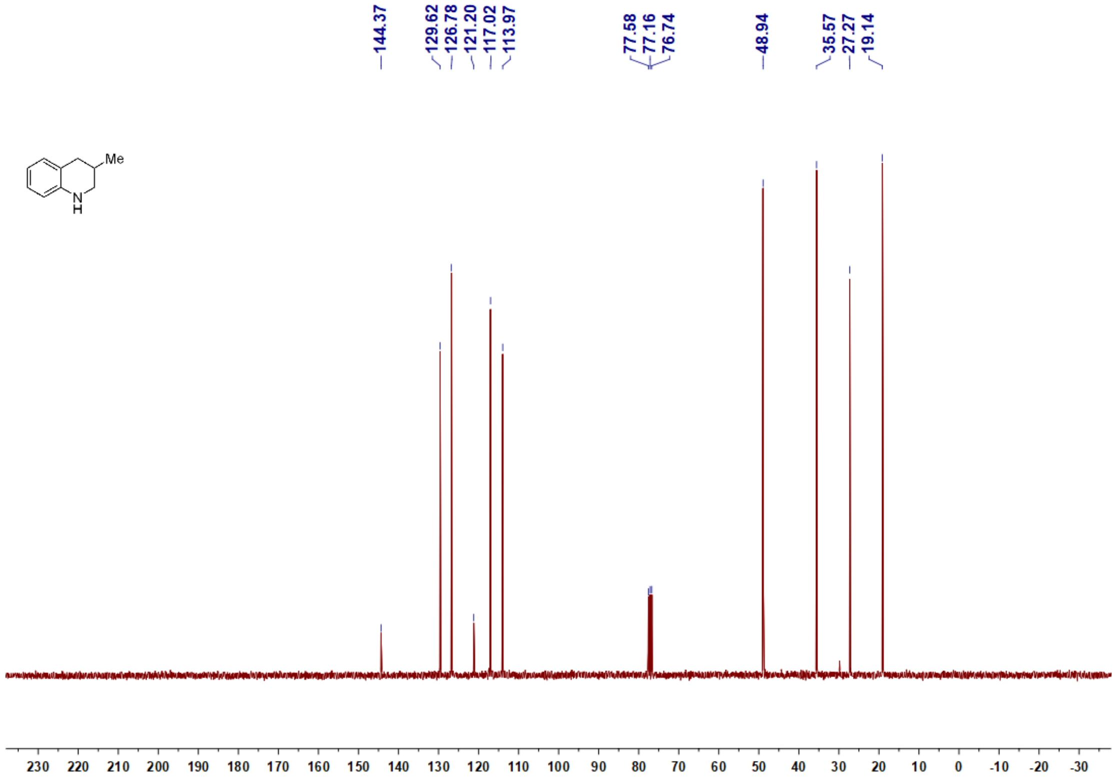

Figure S135. ^{13}C NMR (75 MHz, $CDCl_3$) spectrum of **4d**

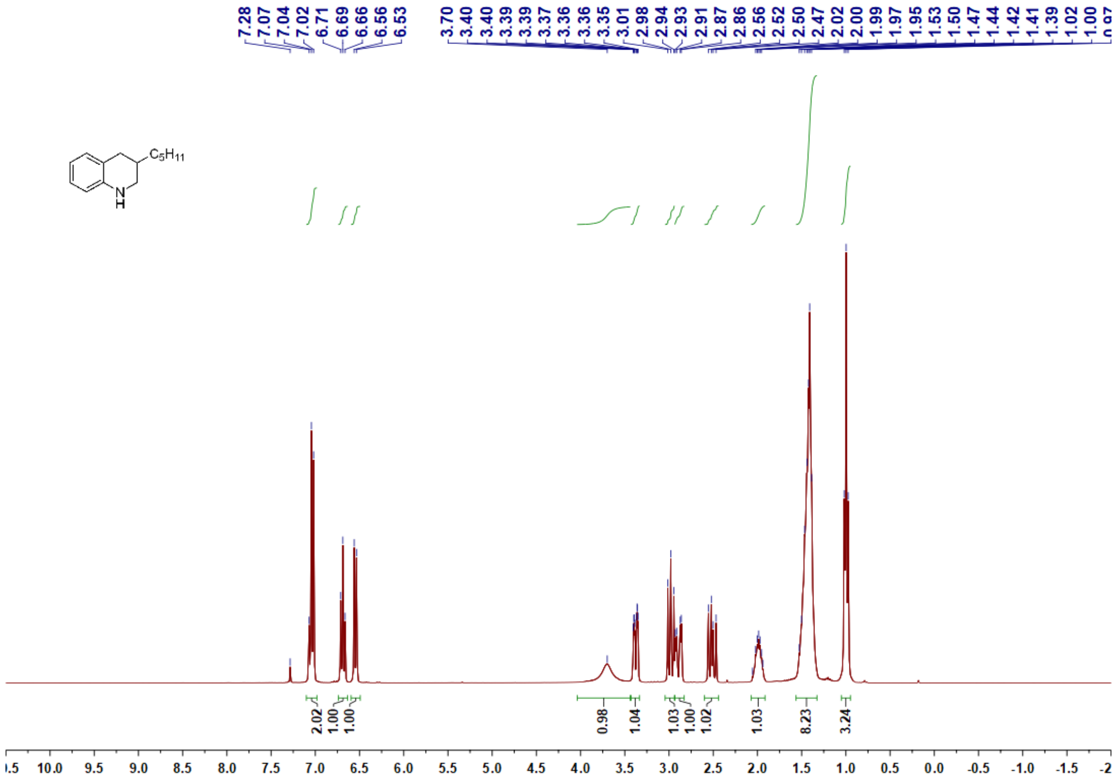

Figure S136. ^{1}H NMR (300 MHz, $CDCl_3$) spectrum of **4e**

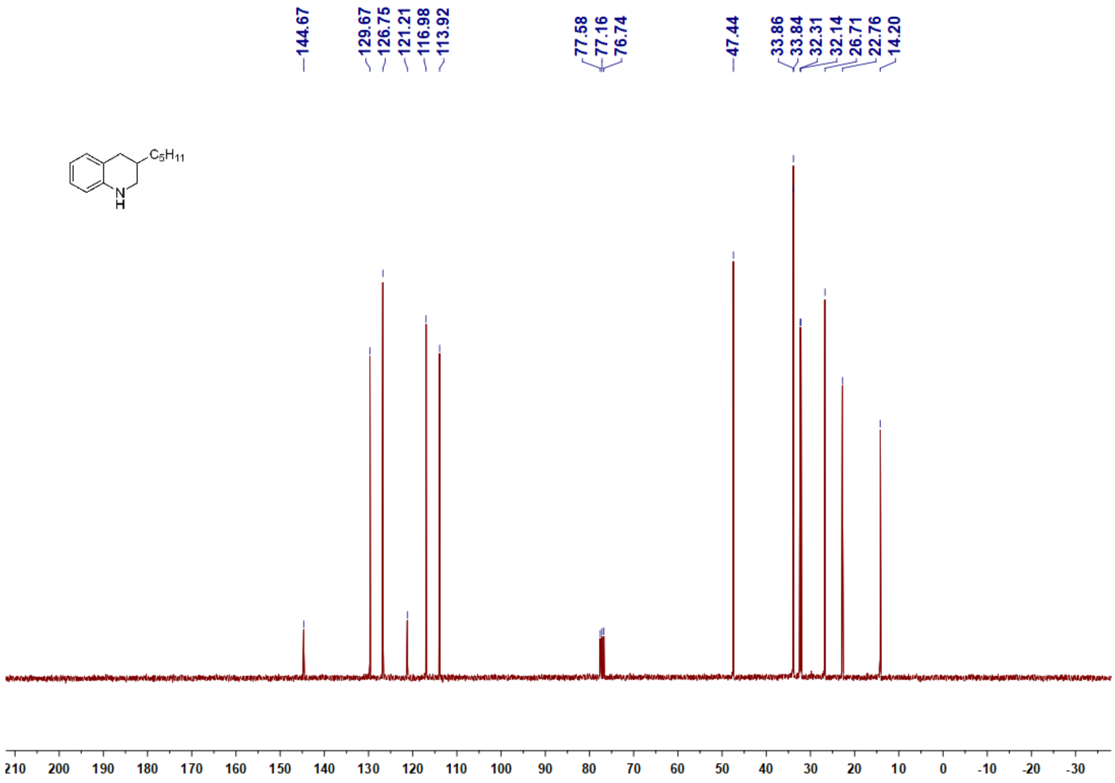

Figure S137. ^{13}C NMR (75 MHz, $CDCl_3$) spectrum of **4e**

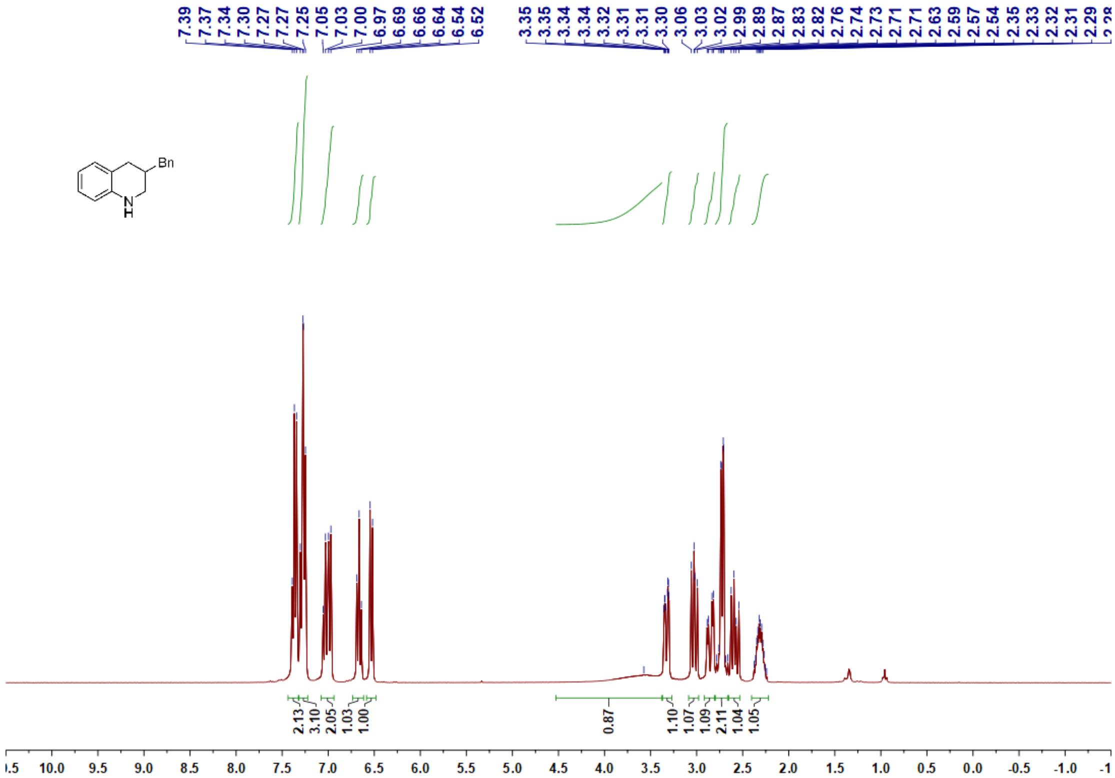

Figure S138. ^{1}H NMR (300 MHz, $CDCl_3$) spectrum of **4f**

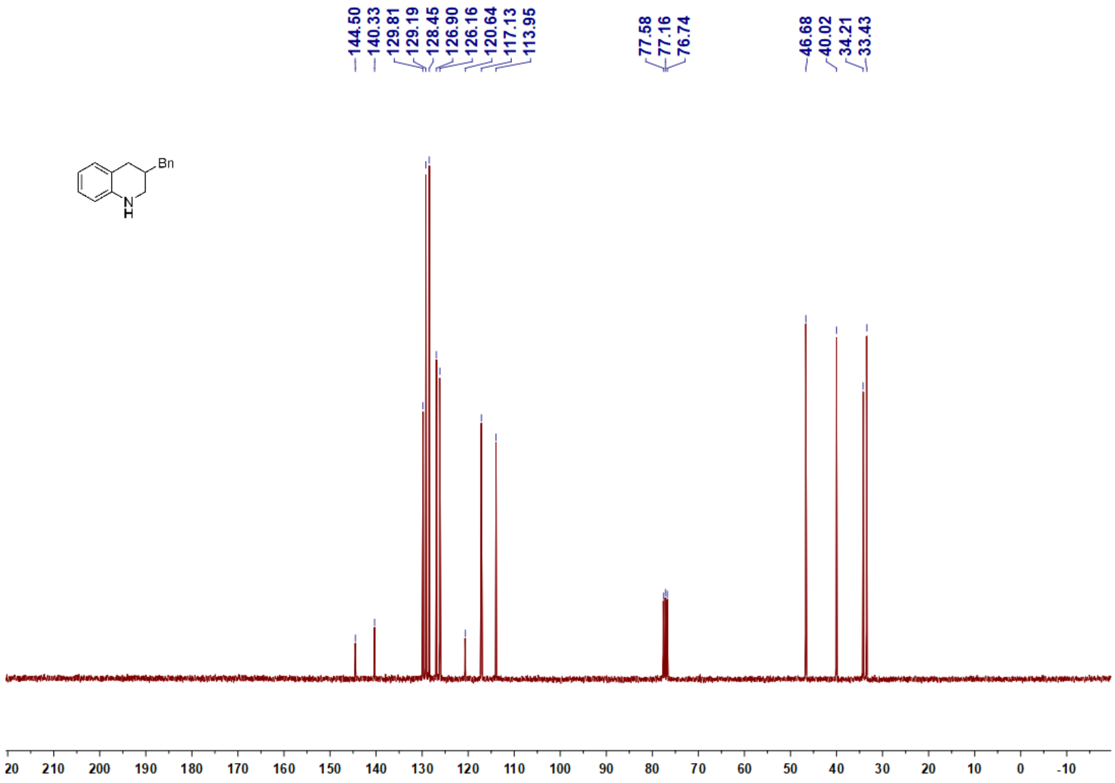

Figure S139. ^{13}C NMR (75 MHz, $CDCl_3$) spectrum of **4f**

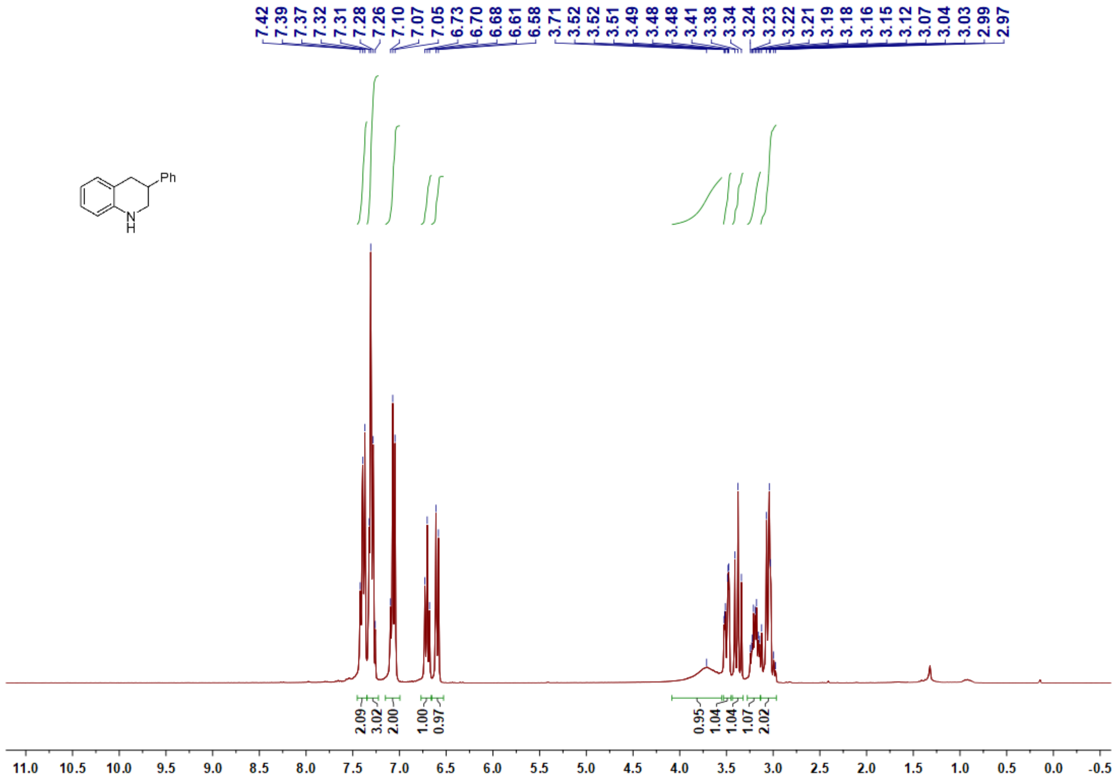

Figure S140. ^{1}H NMR (300 MHz, $CDCl_3$) spectrum of **4g**

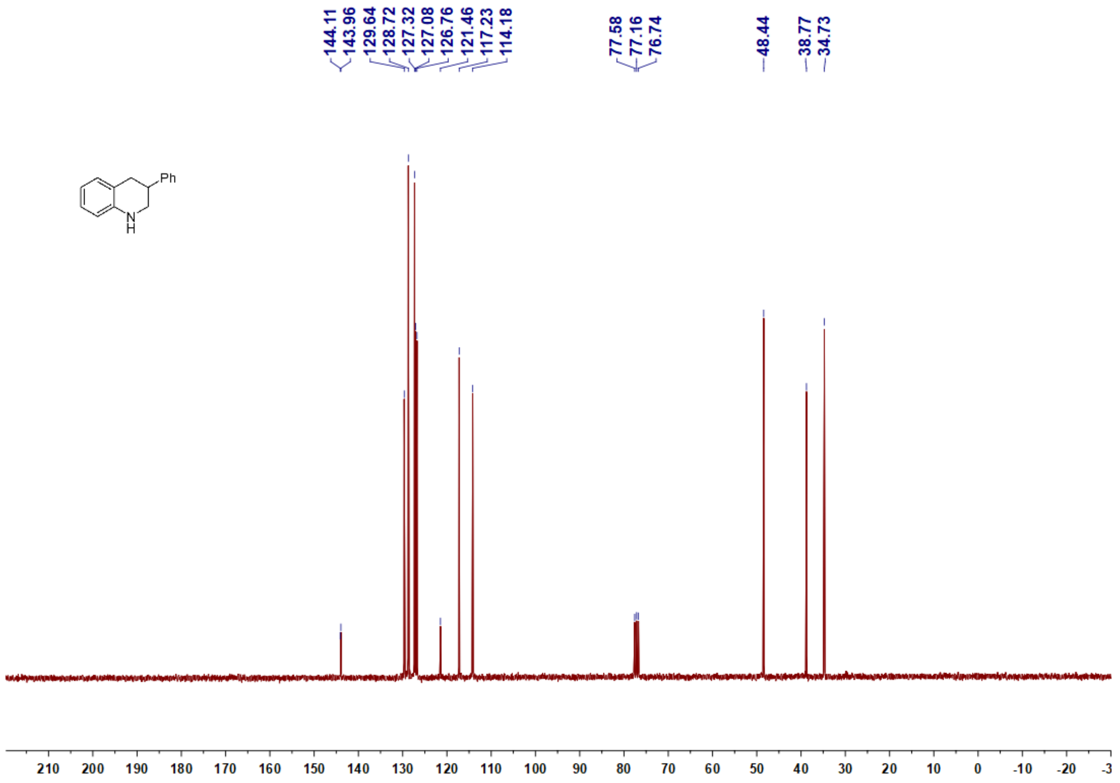

Figure S141. ^{13}C NMR (75 MHz, $CDCl_3$) spectrum of **4g**

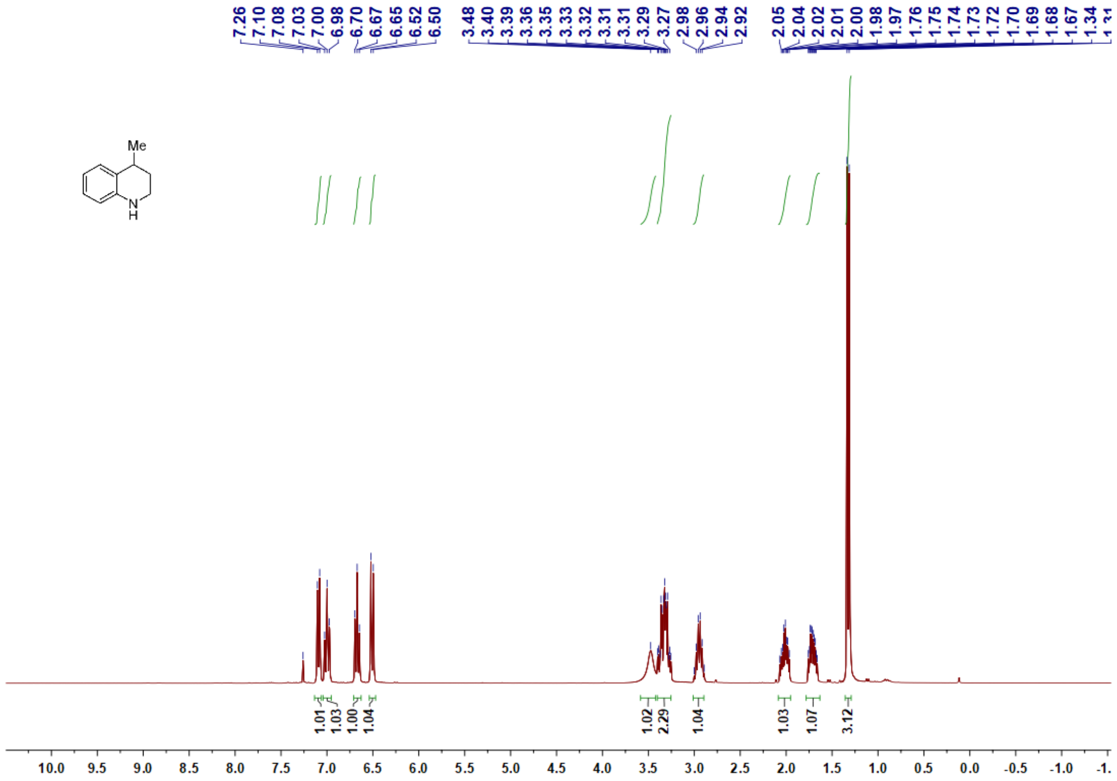

Figure S142. ^{1}H NMR (300 MHz, $CDCl_3$) spectrum of **4h**

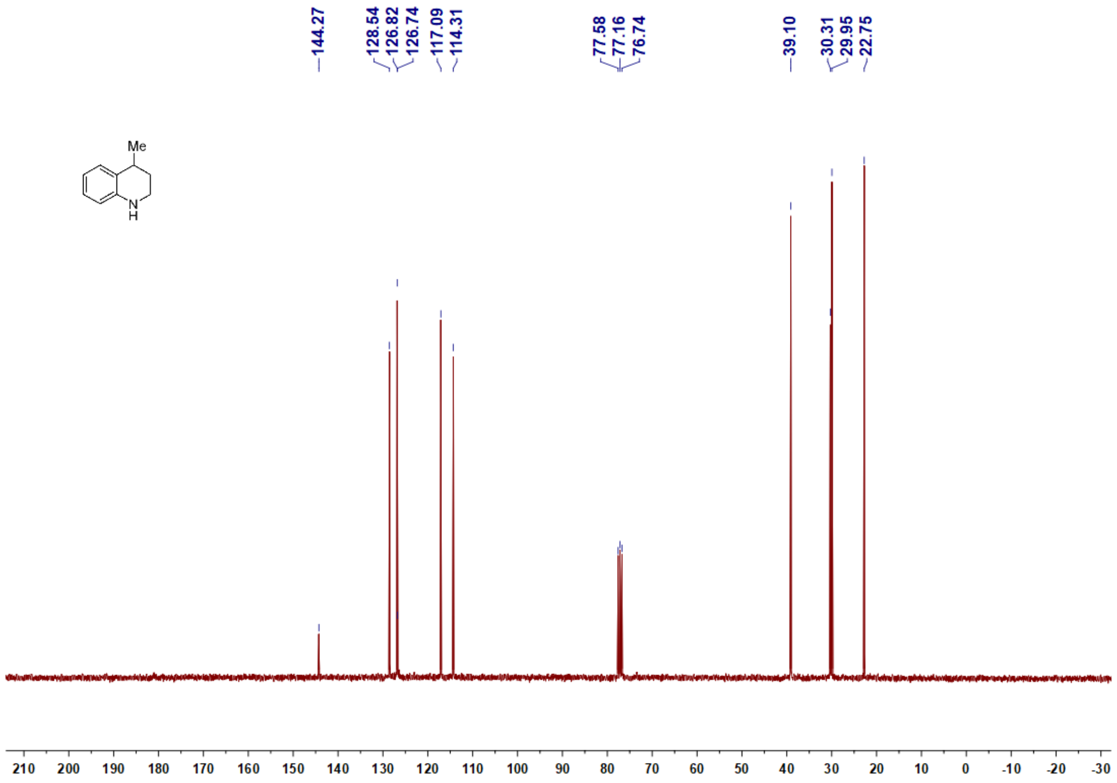

Figure S143. ^{13}C NMR (75 MHz, $CDCl_3$) spectrum of **4h**

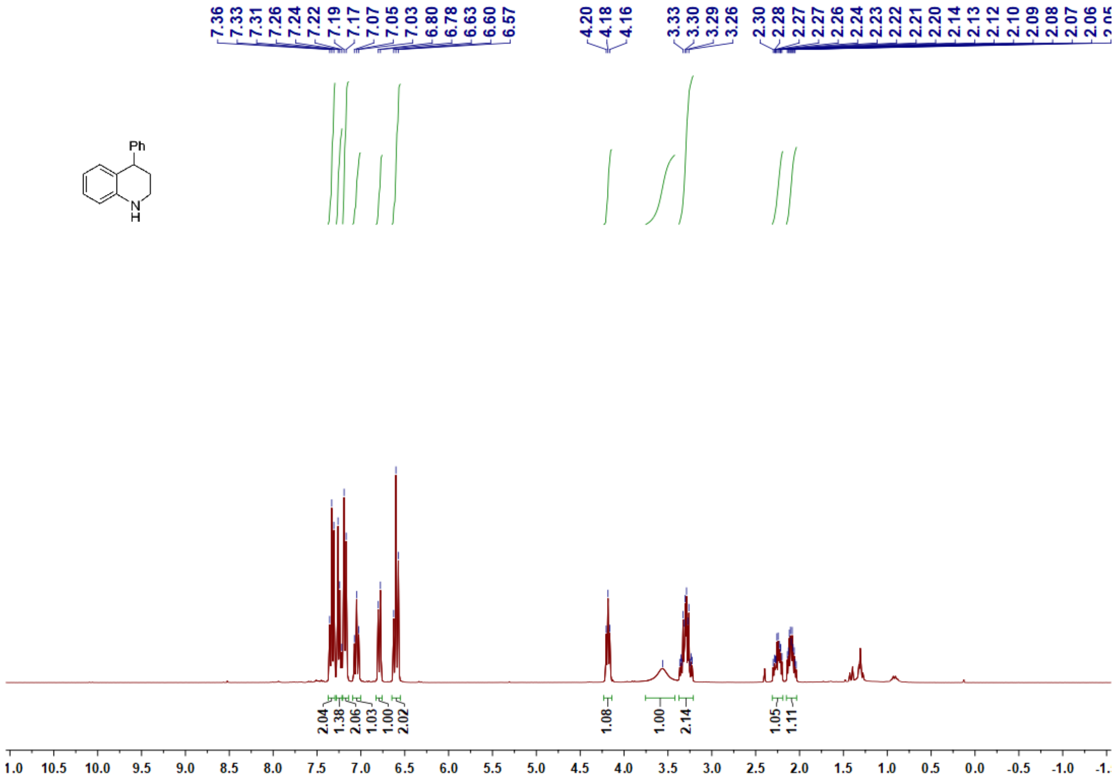

Figure S144. ^{1}H NMR (300 MHz, $CDCl_3$) spectrum of **4i**

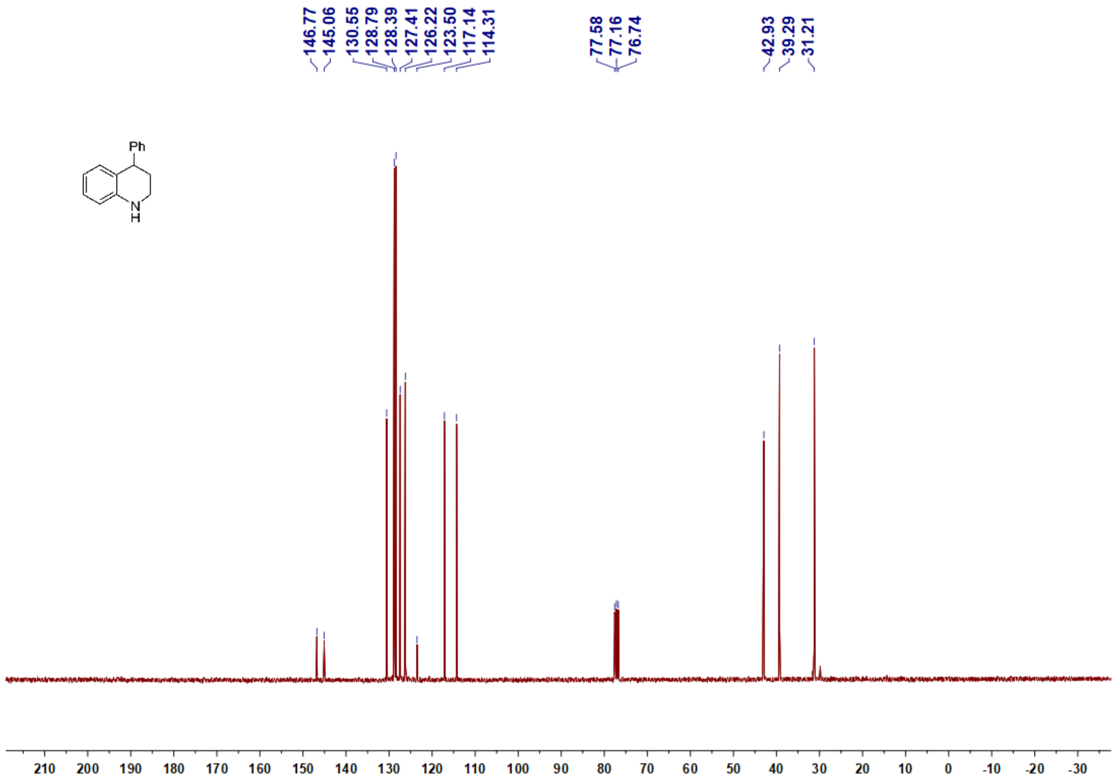

Figure S145. ^{13}C NMR (75 MHz, $CDCl_3$) spectrum of **4i**

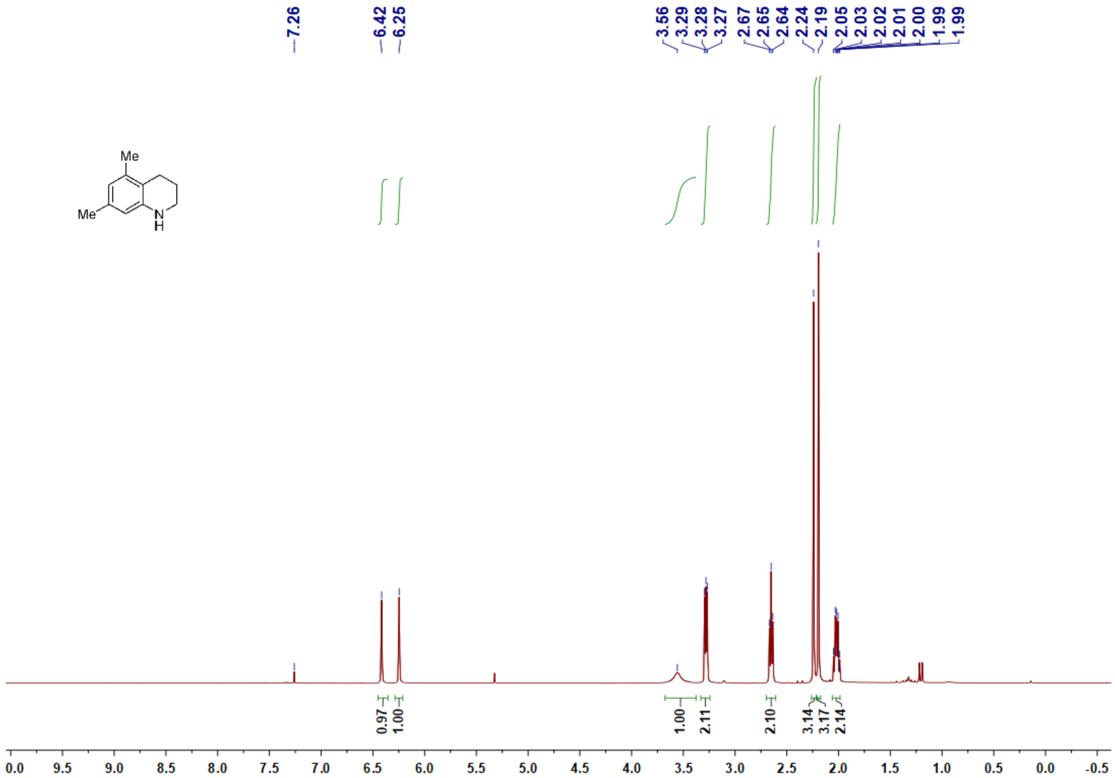

Figure S146. ^{1}H NMR (400 MHz, $CDCl_3$) spectrum of **4j**

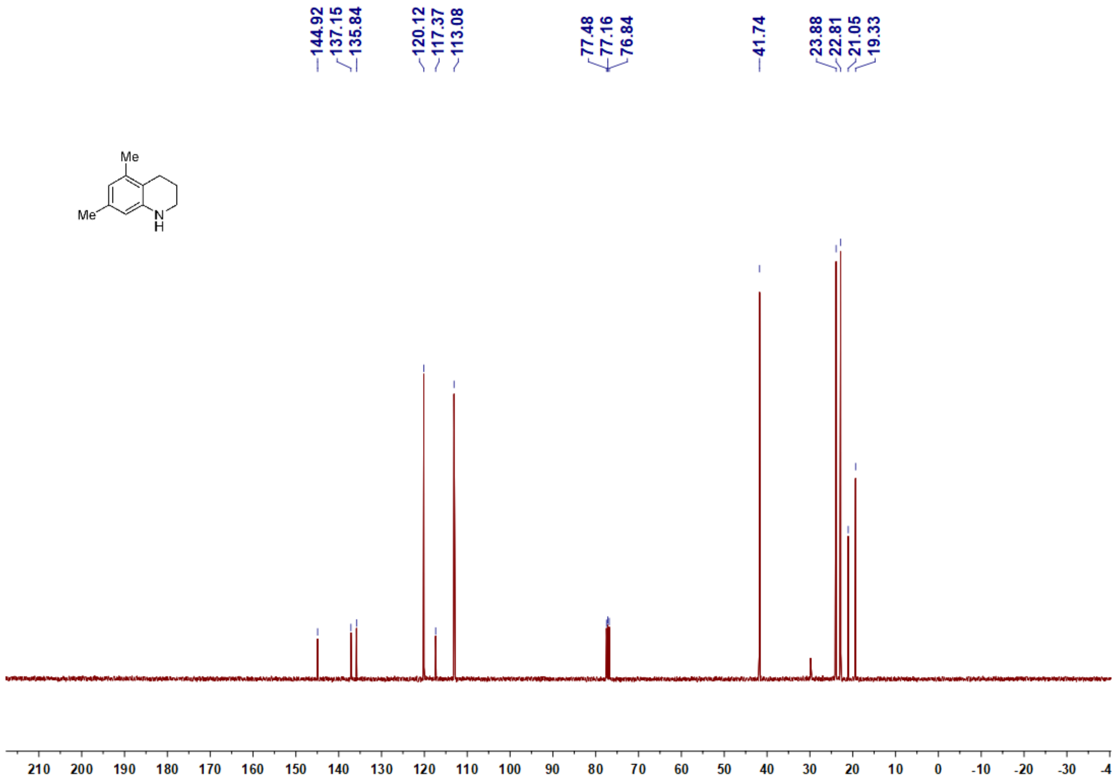

Figure S147. ^{13}C NMR (101 MHz, $CDCl_3$) spectrum of **4j**

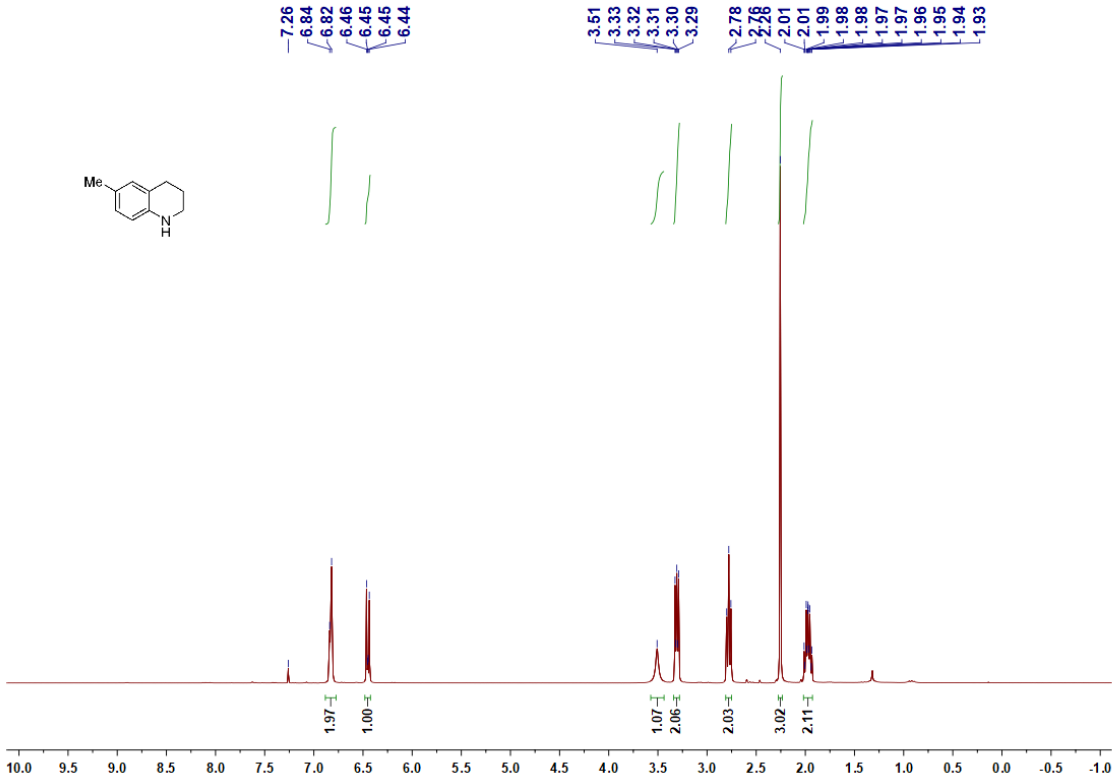

Figure S148. ^{1}H NMR (300 MHz, $CDCl_3$) spectrum of **4k**

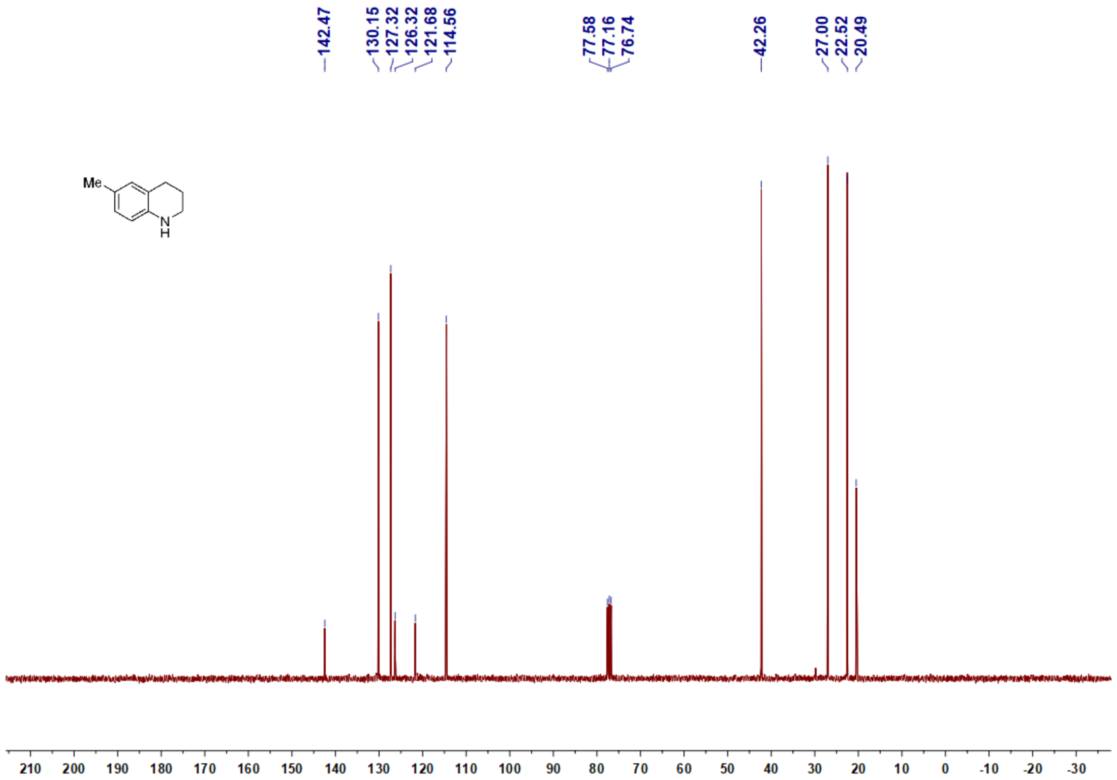

Figure S149. ^{13}C NMR (75 MHz, $CDCl_3$) spectrum of **4k**

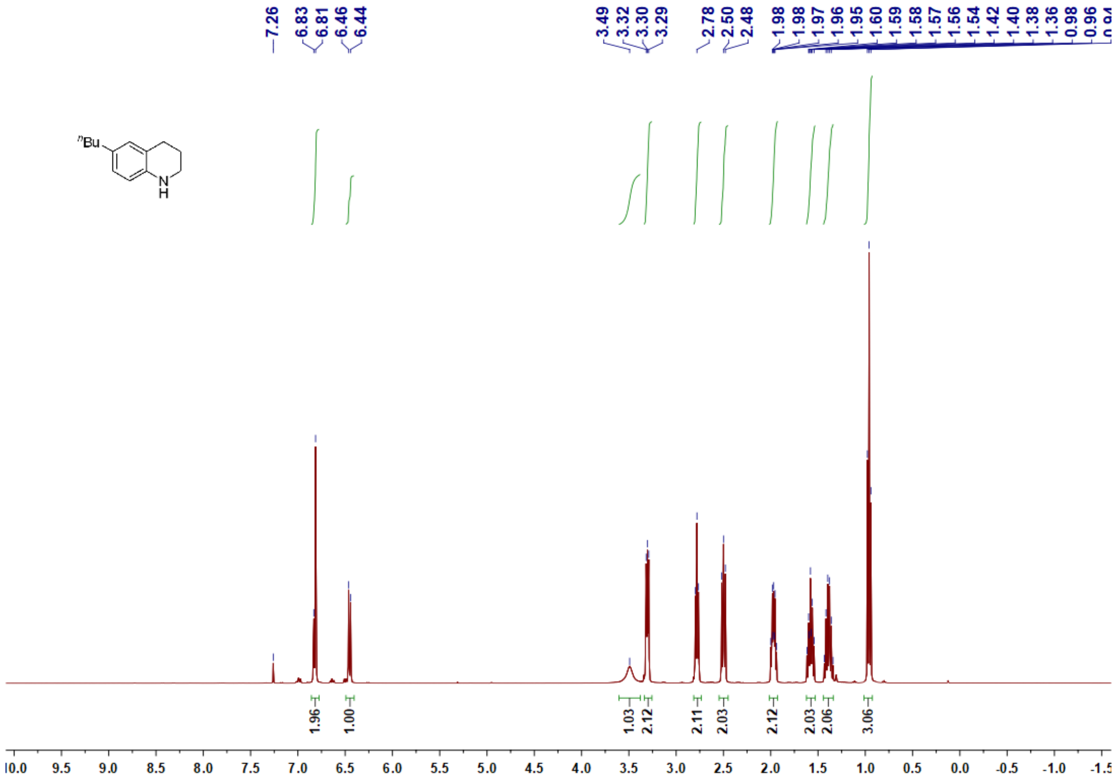

Figure S150. ^{1}H NMR (400 MHz, $CDCl_3$) spectrum of **4l**

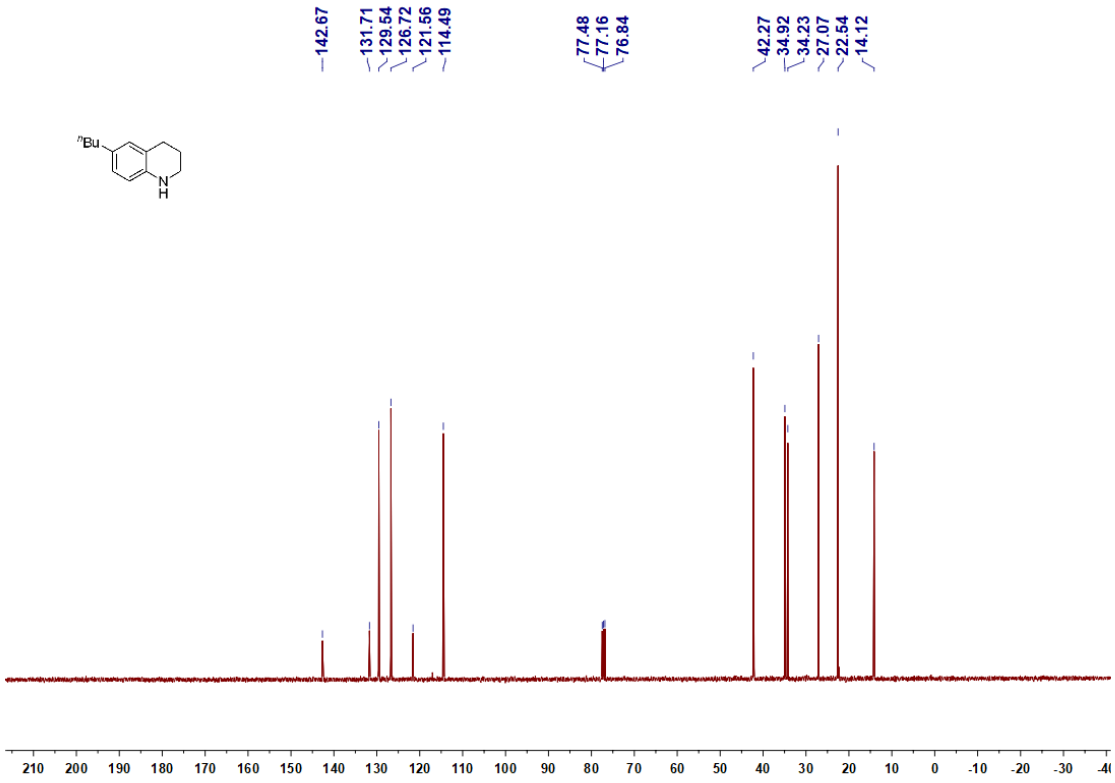

Figure S151. ^{13}C NMR (101 MHz, $CDCl_3$) spectrum of **4l**

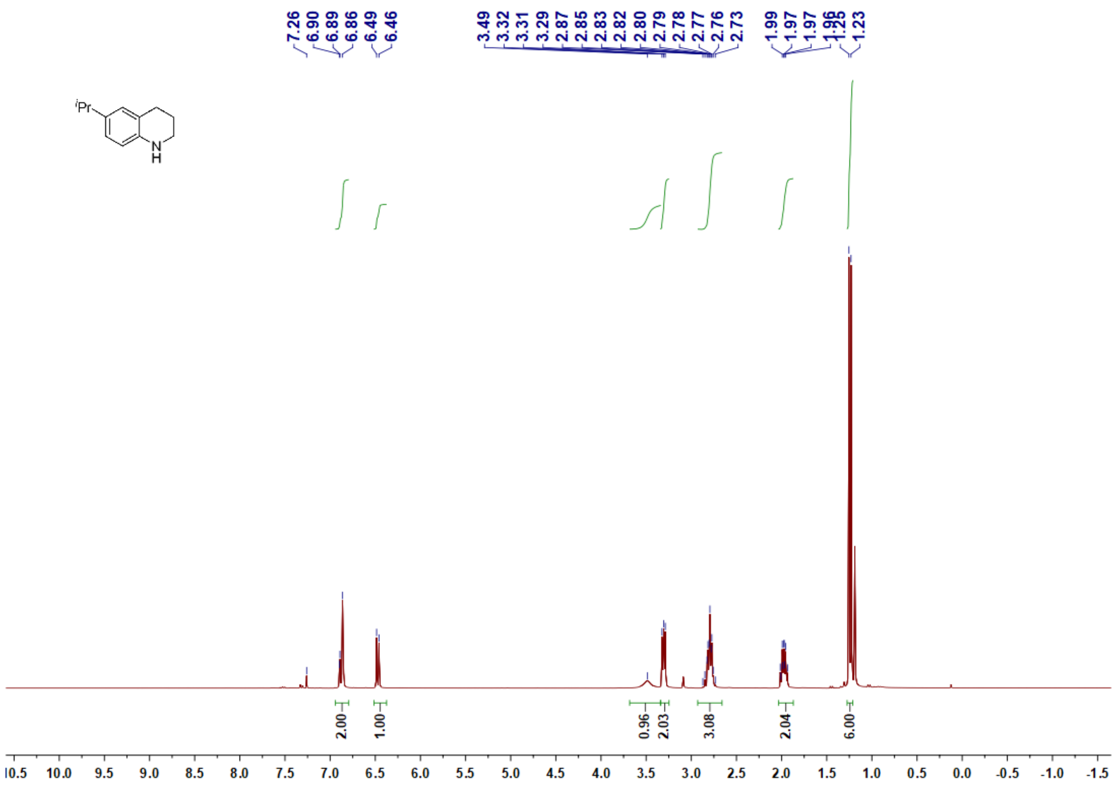

Figure S152. ^{1}H NMR (300 MHz, $CDCl_3$) spectrum of **4m**

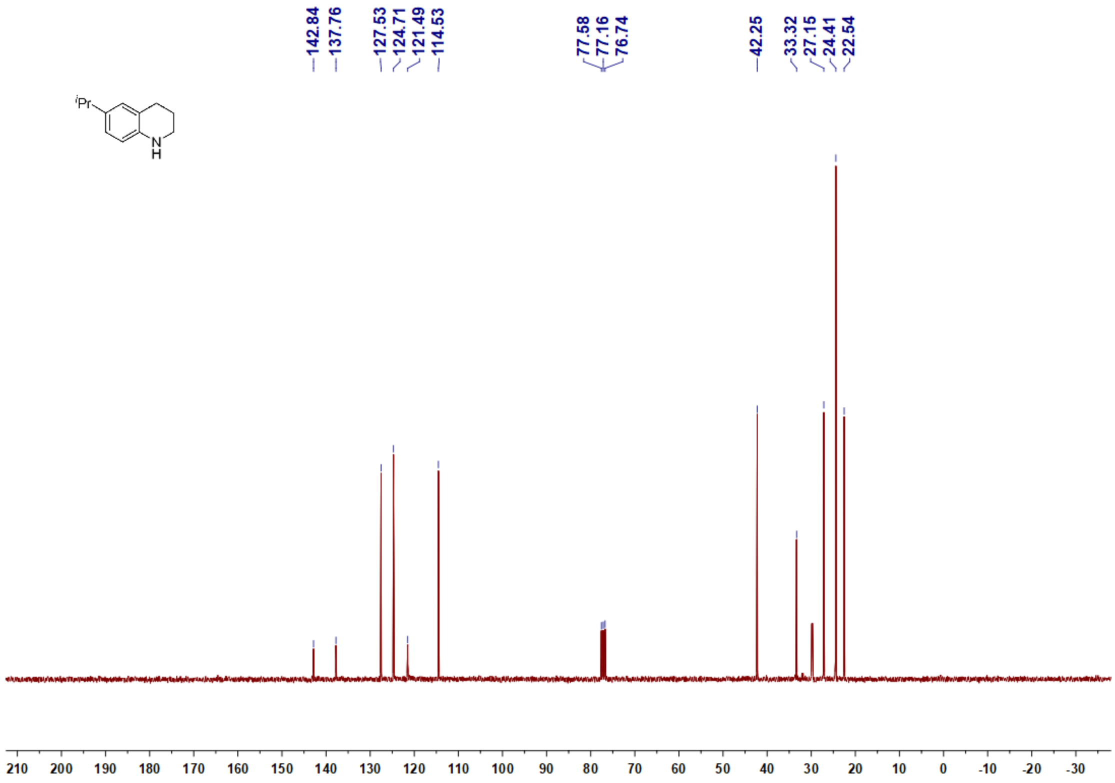

Figure S153. ^{13}C NMR (75 MHz, $CDCl_3$) spectrum of **4m**

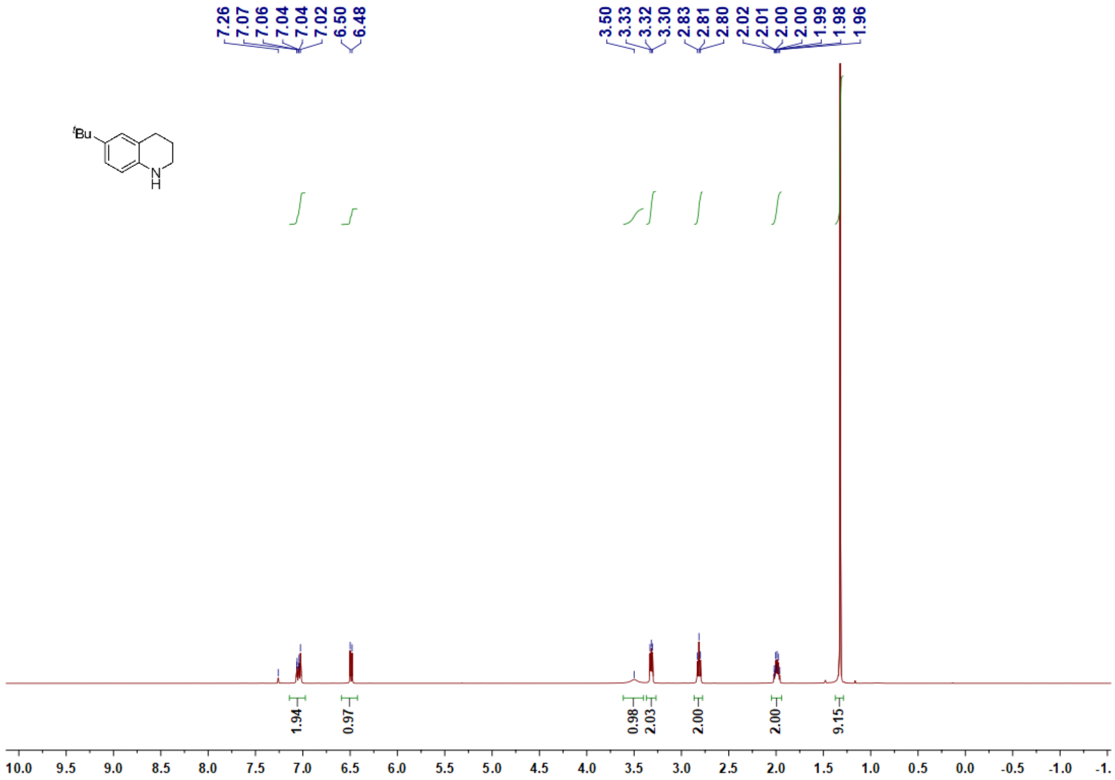

Figure S154. ^{1}H NMR (400 MHz, $CDCl_3$) spectrum of **4n**

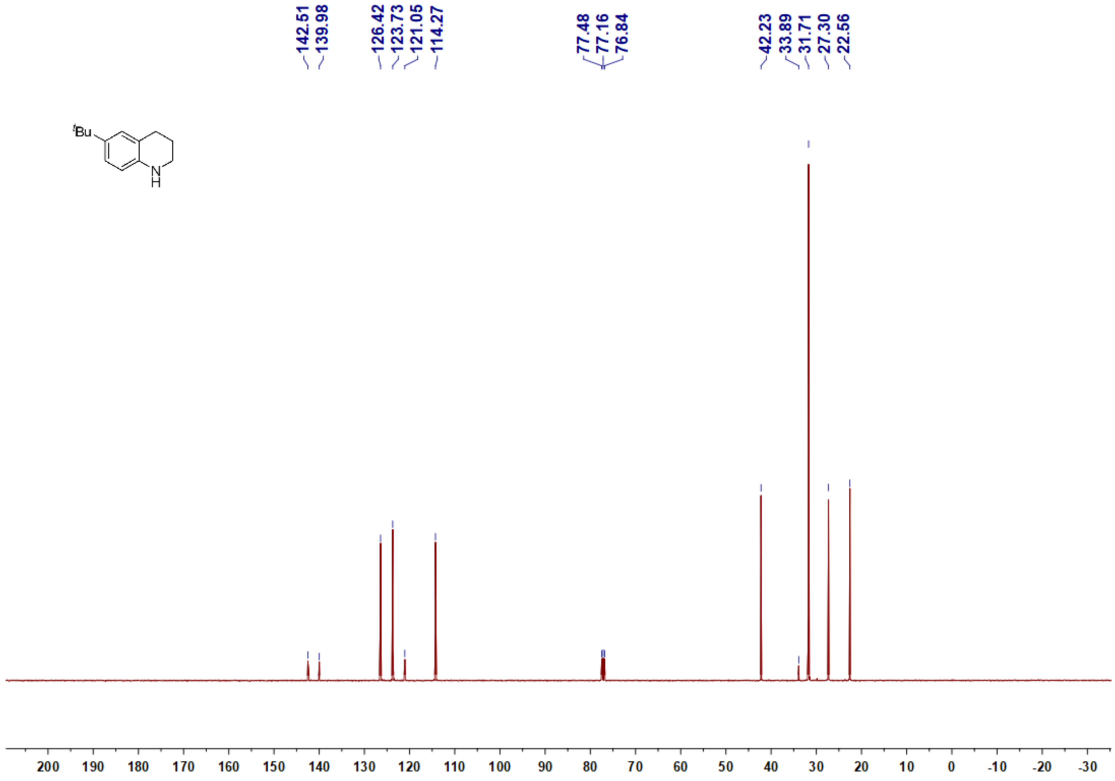

Figure S155. ^{13}C NMR (101 MHz, $CDCl_3$) spectrum of **4n**

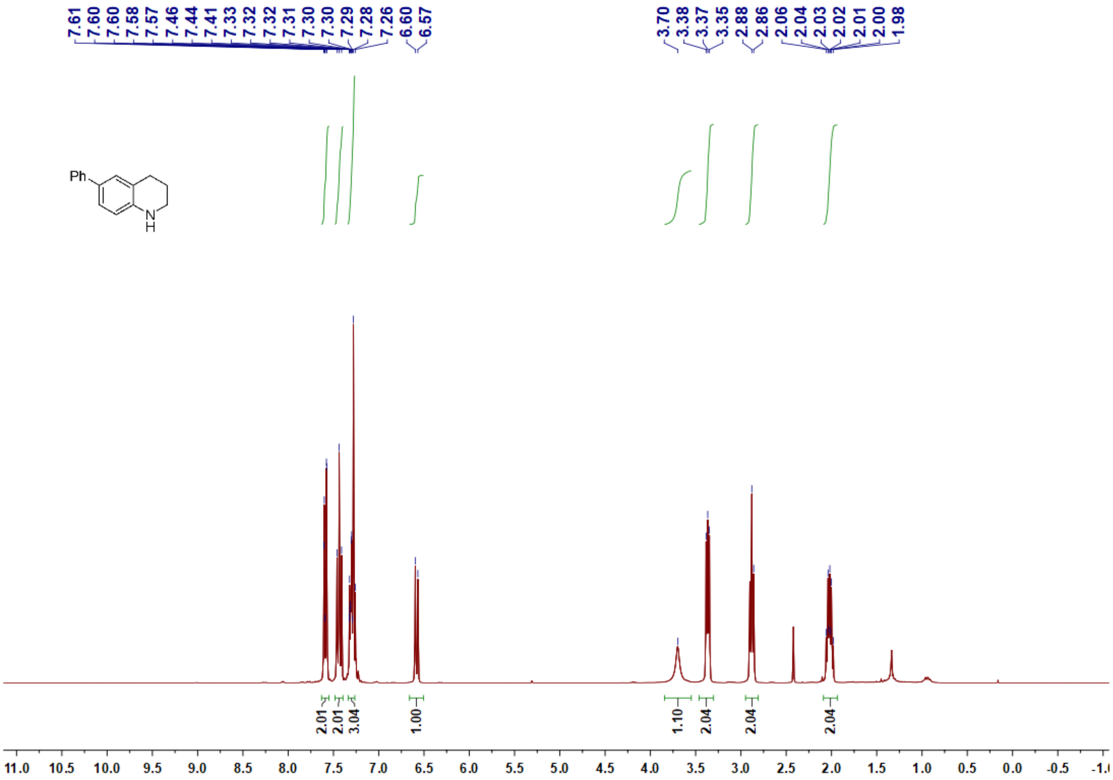

Figure S156. ^{1}H NMR (300 MHz, $CDCl_3$) spectrum of **4o**

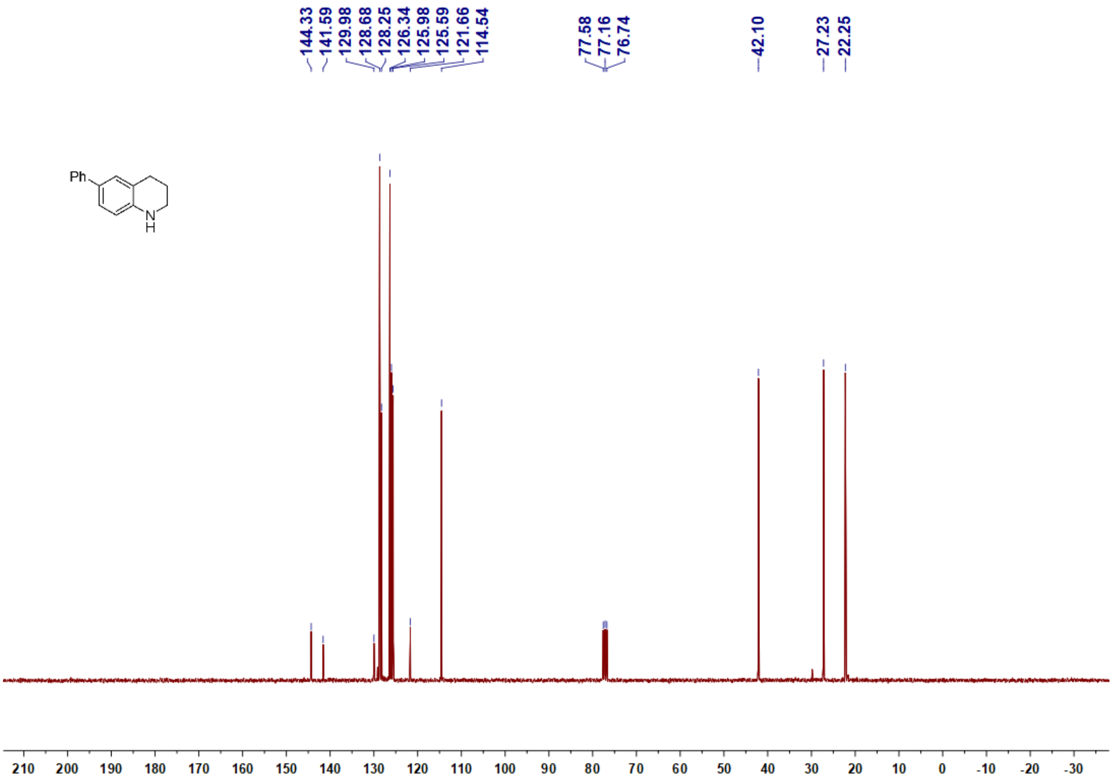

Figure S157. ^{13}C NMR (75 MHz, $CDCl_3$) spectrum of **4o**

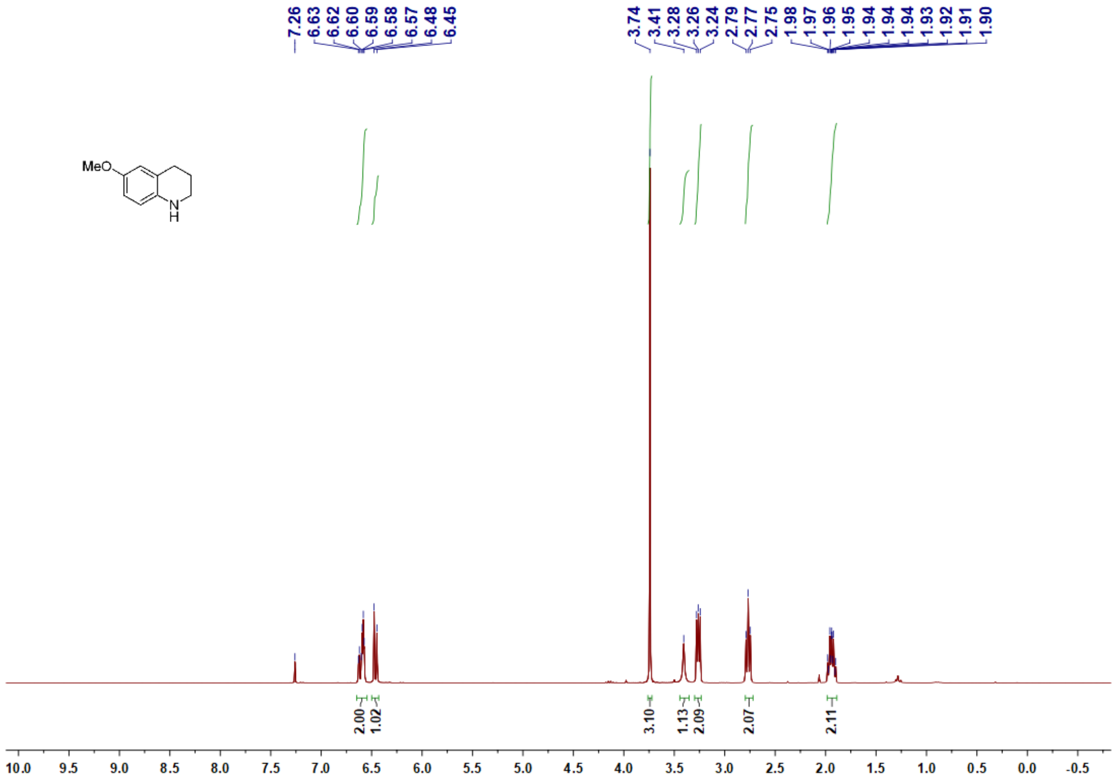

Figure S158. ^{1}H NMR (300 MHz, $CDCl_3$) spectrum of **4p**

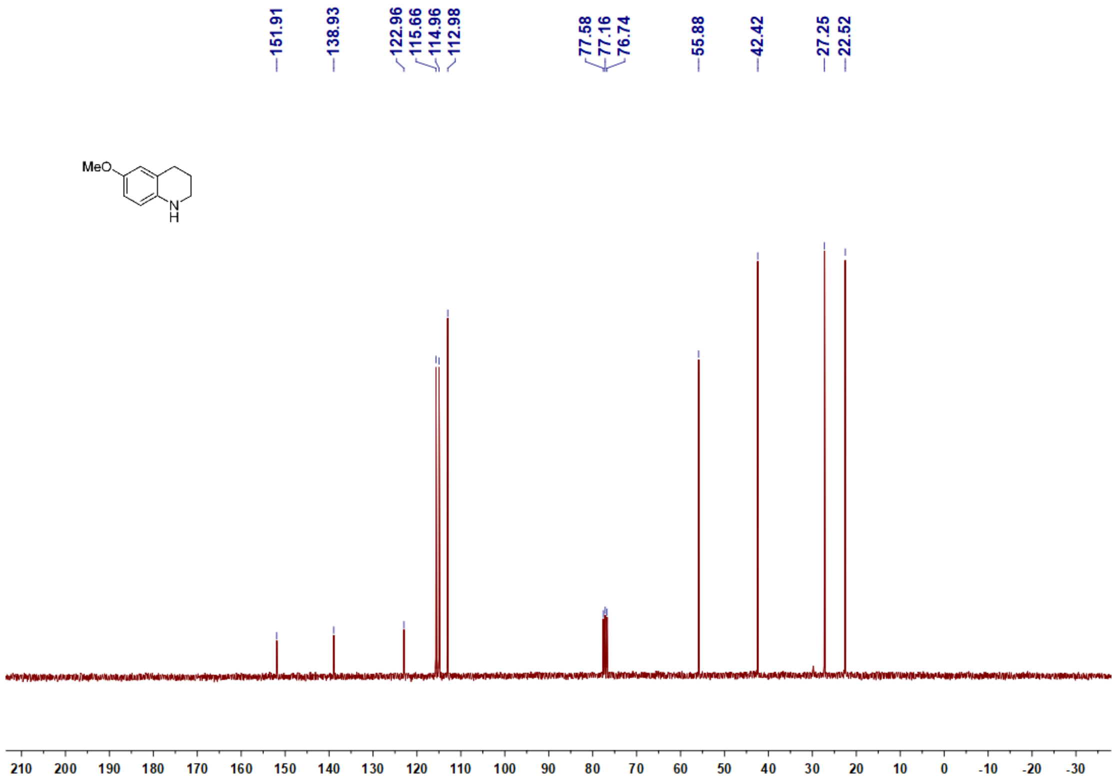

Figure S159. ^{13}C NMR (75 MHz, $CDCl_3$) spectrum of **4p**

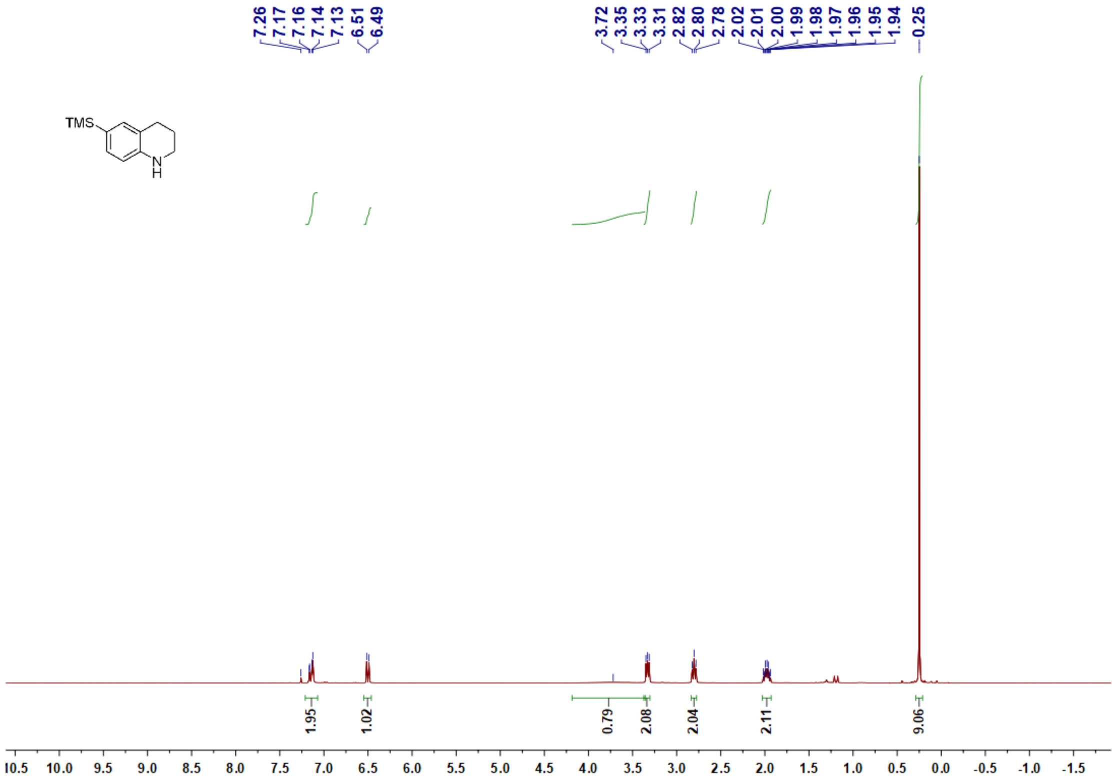

Figure S160. ^{1}H NMR (300 MHz, $CDCl_3$) spectrum of **4q**

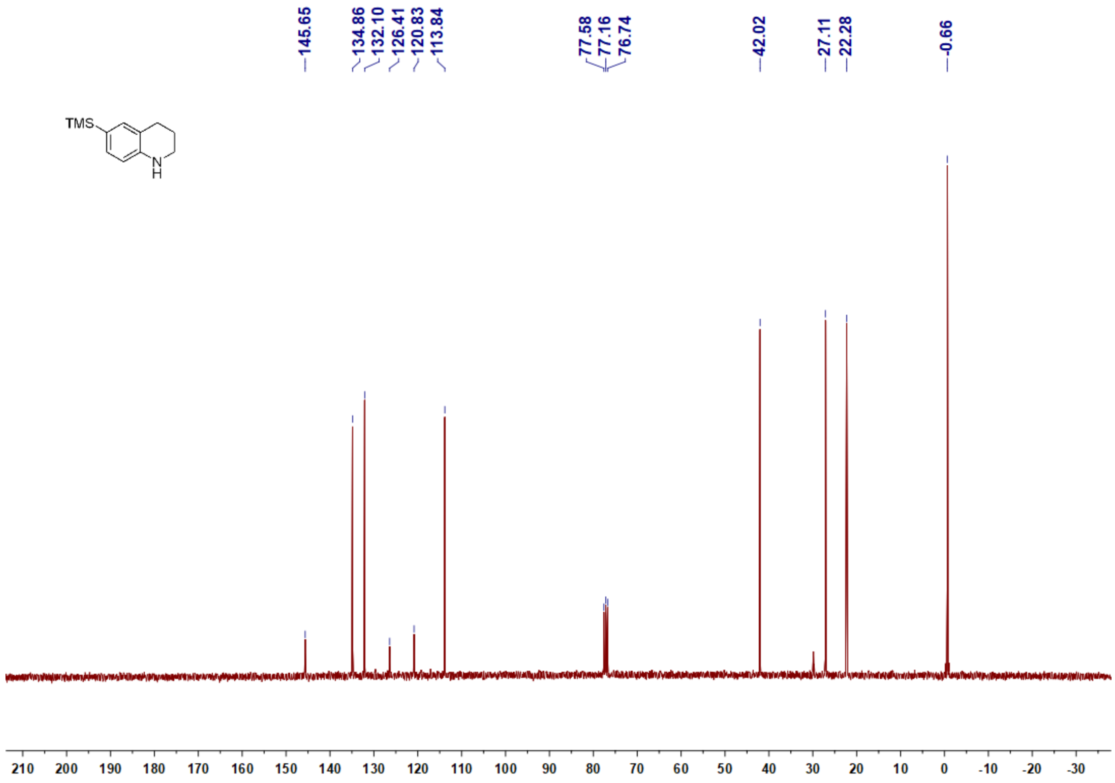

Figure S161. ^{13}C NMR (75 MHz, $CDCl_3$) spectrum of **4q**

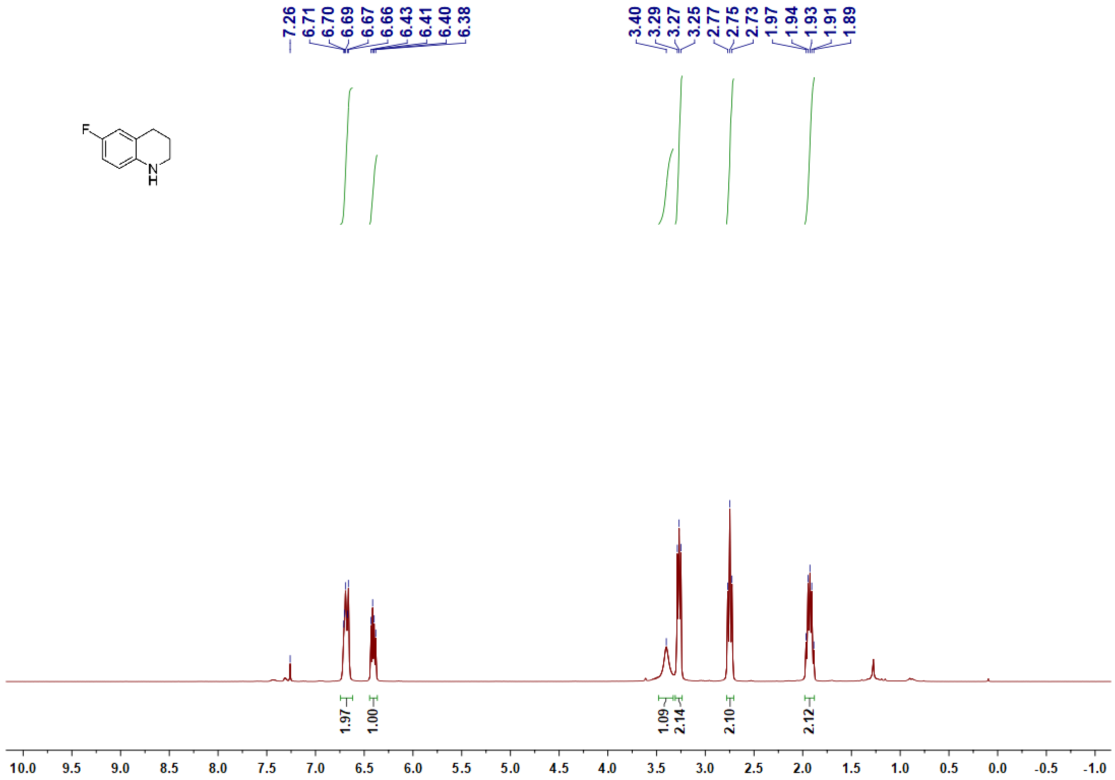

Figure S162. ^{1}H NMR (300 MHz, $CDCl_3$) spectrum of **4r**

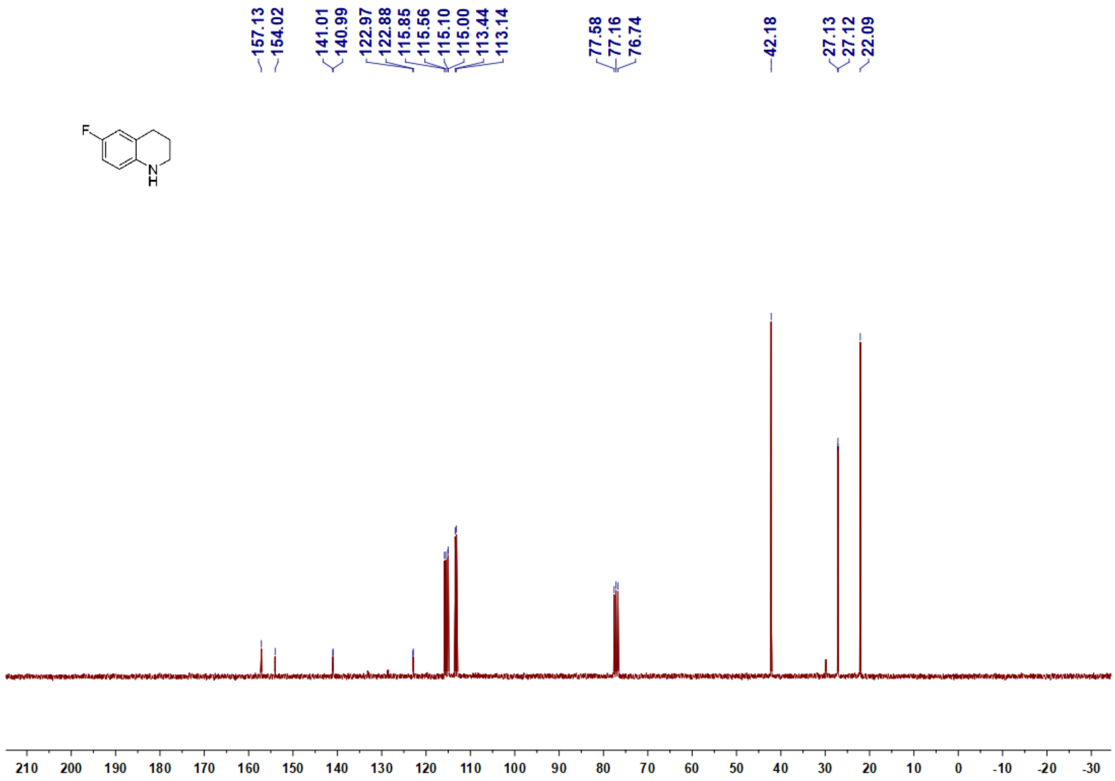

Figure S163. ^{13}C NMR (75 MHz, $CDCl_3$) spectrum of **4r**

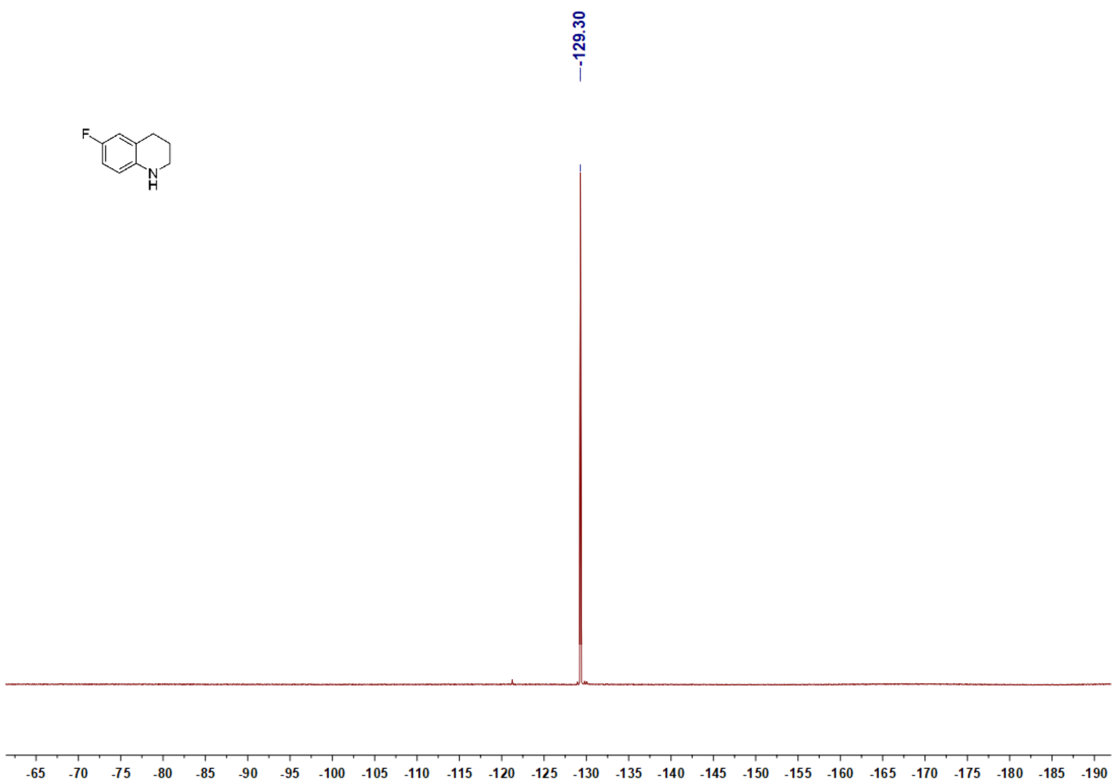

Figure S164. ^{19}F NMR (282 MHz, $CDCl_3$) spectrum of **4r**

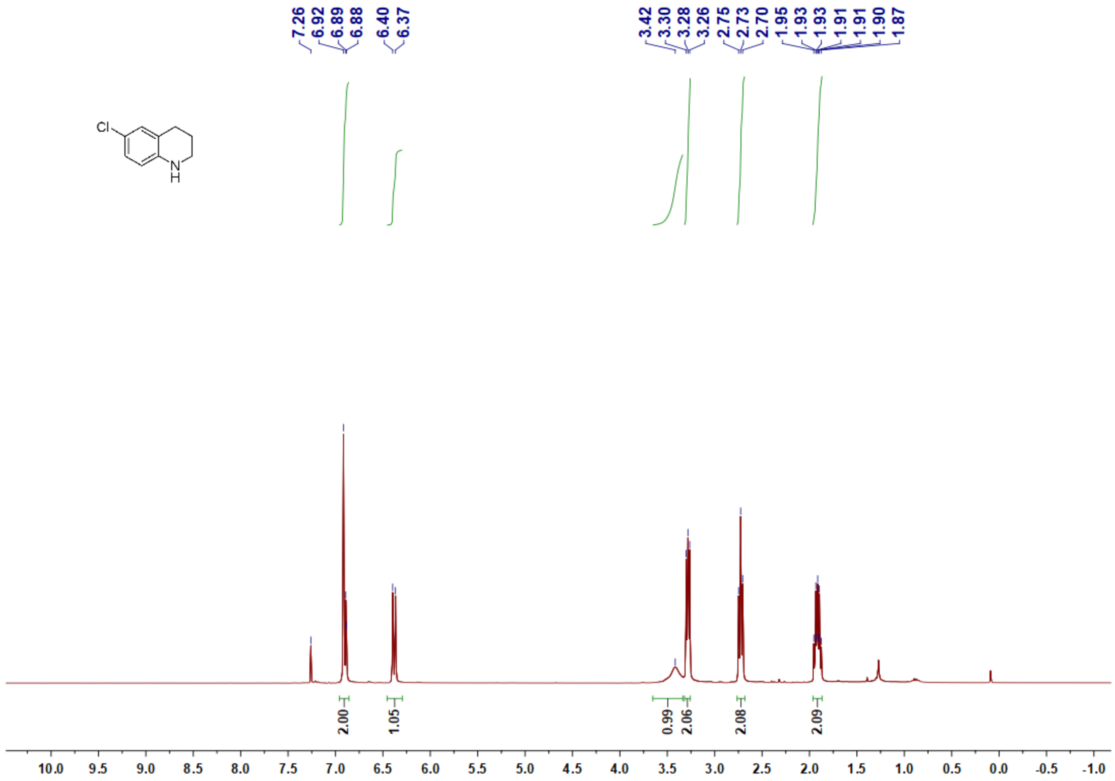

Figure S165. ^{1}H NMR (300 MHz, $CDCl_3$) spectrum of **4s**

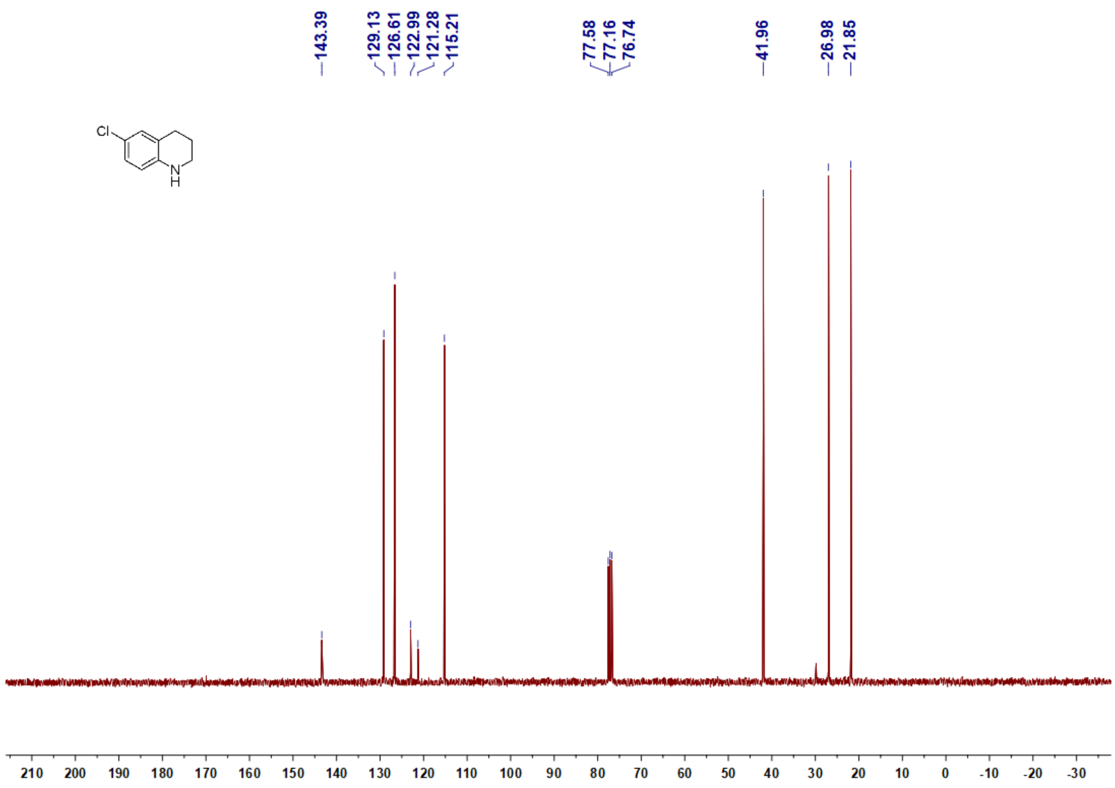

Figure S166. ^{13}C NMR (75 MHz, $CDCl_3$) spectrum of **4s**

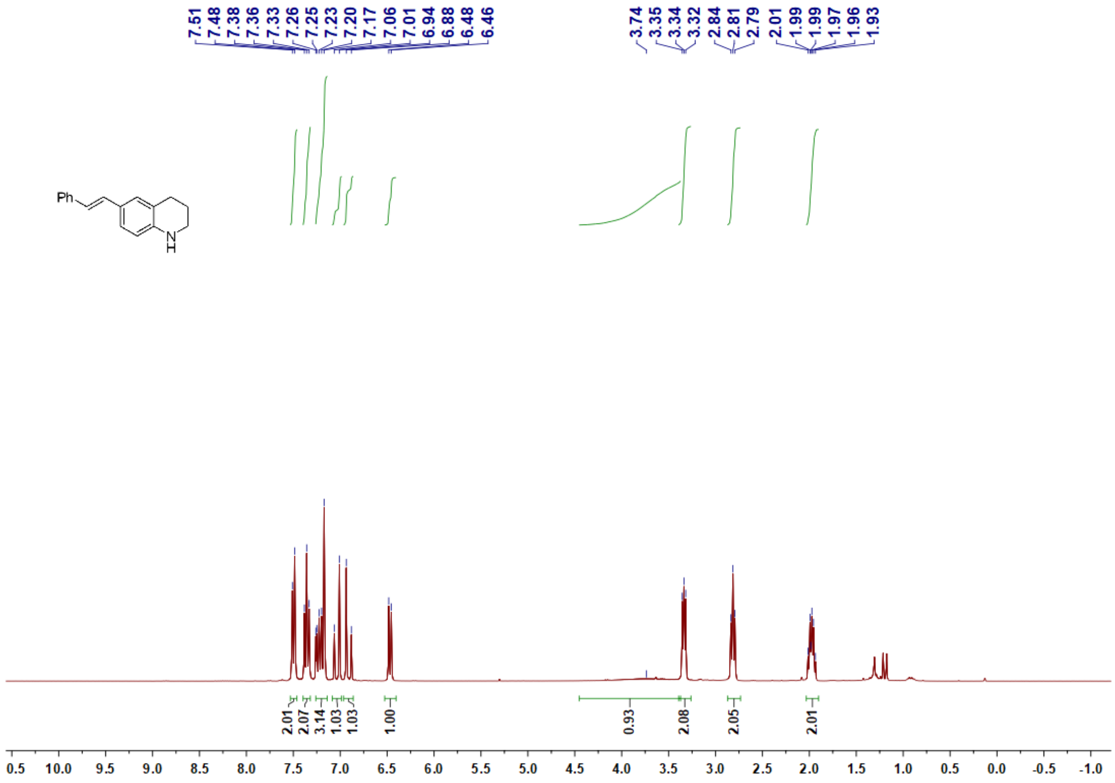

Figure S167. ^{1}H NMR (300 MHz, $CDCl_3$) spectrum of **4t**

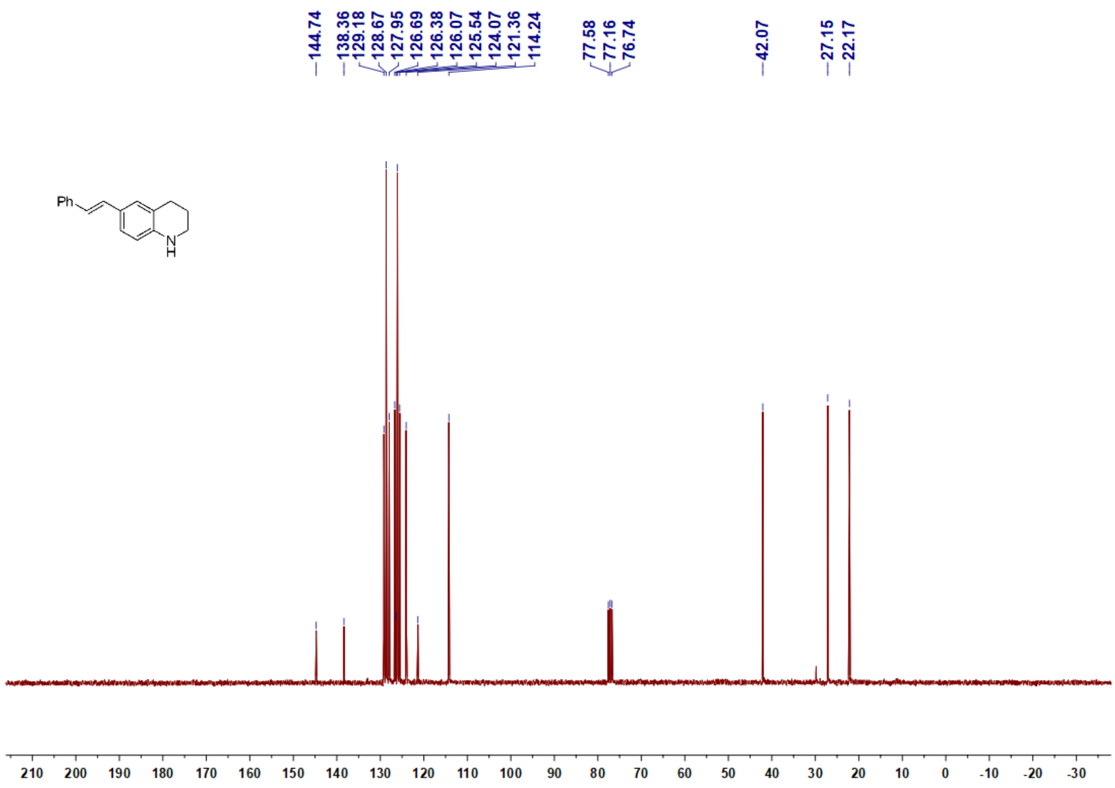

Figure S168. ^{13}C NMR (75 MHz, $CDCl_3$) spectrum of **4t**

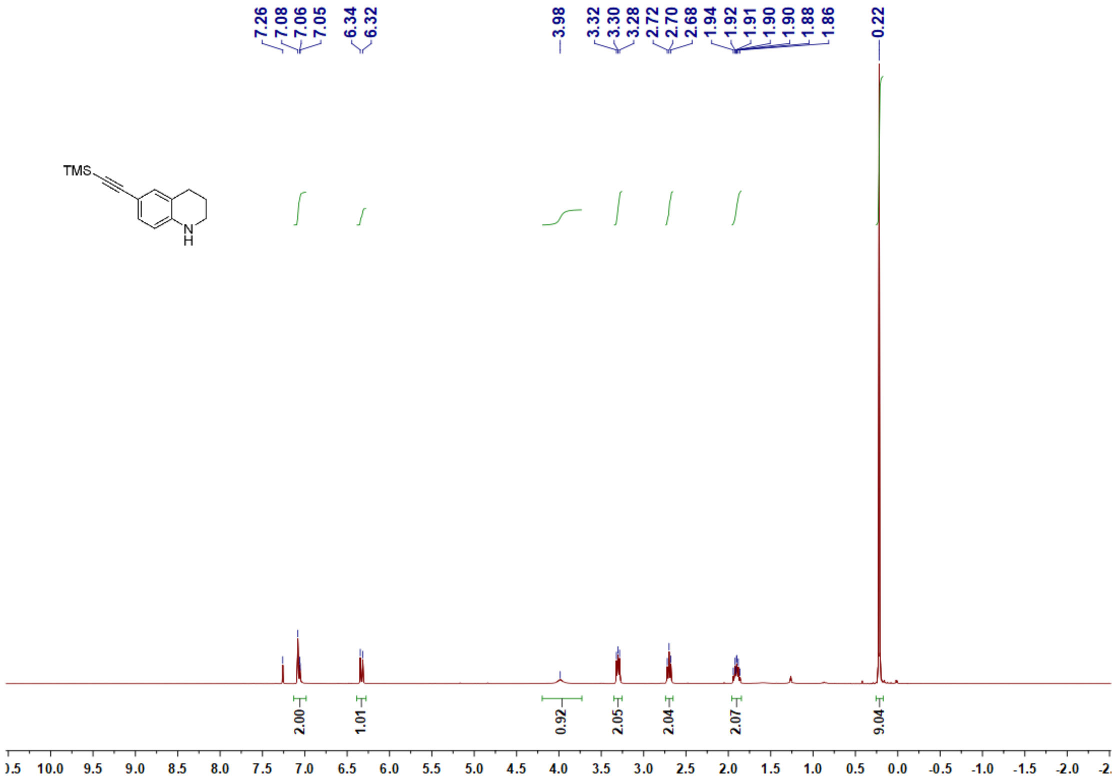

Figure S169. ^{1}H NMR (300 MHz, $CDCl_3$) spectrum of **4u**

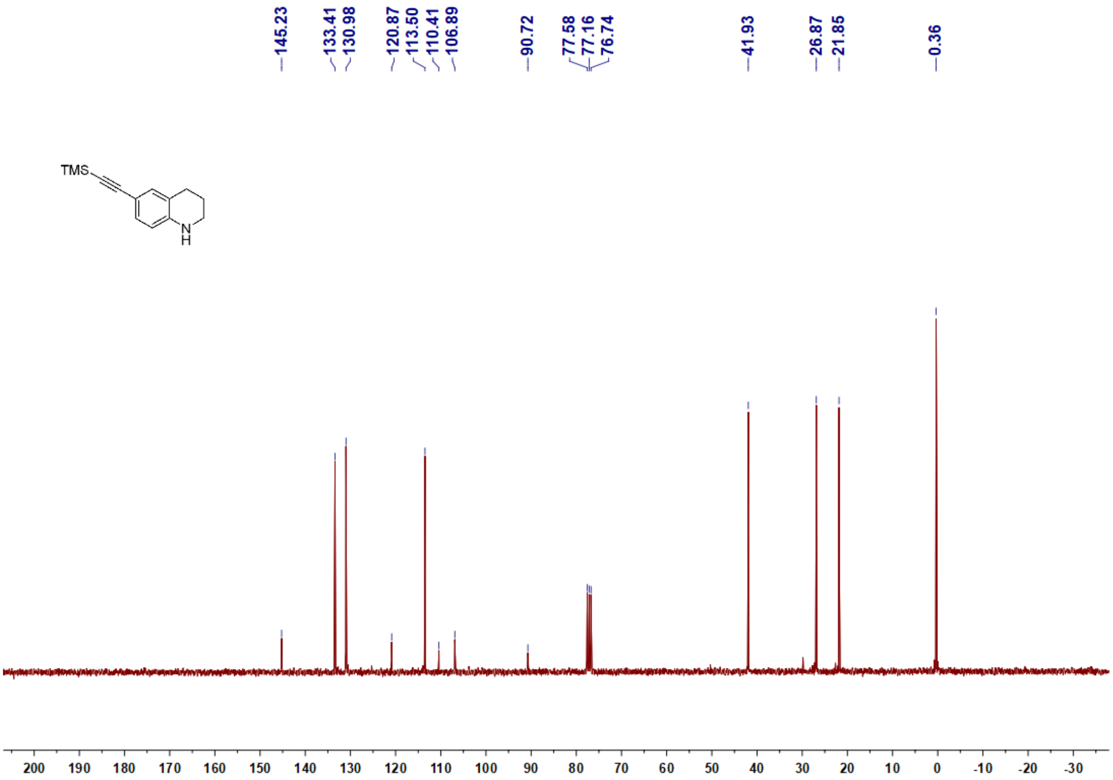

Figure S170. ^{13}C NMR (75 MHz, $CDCl_3$) spectrum of **4u**

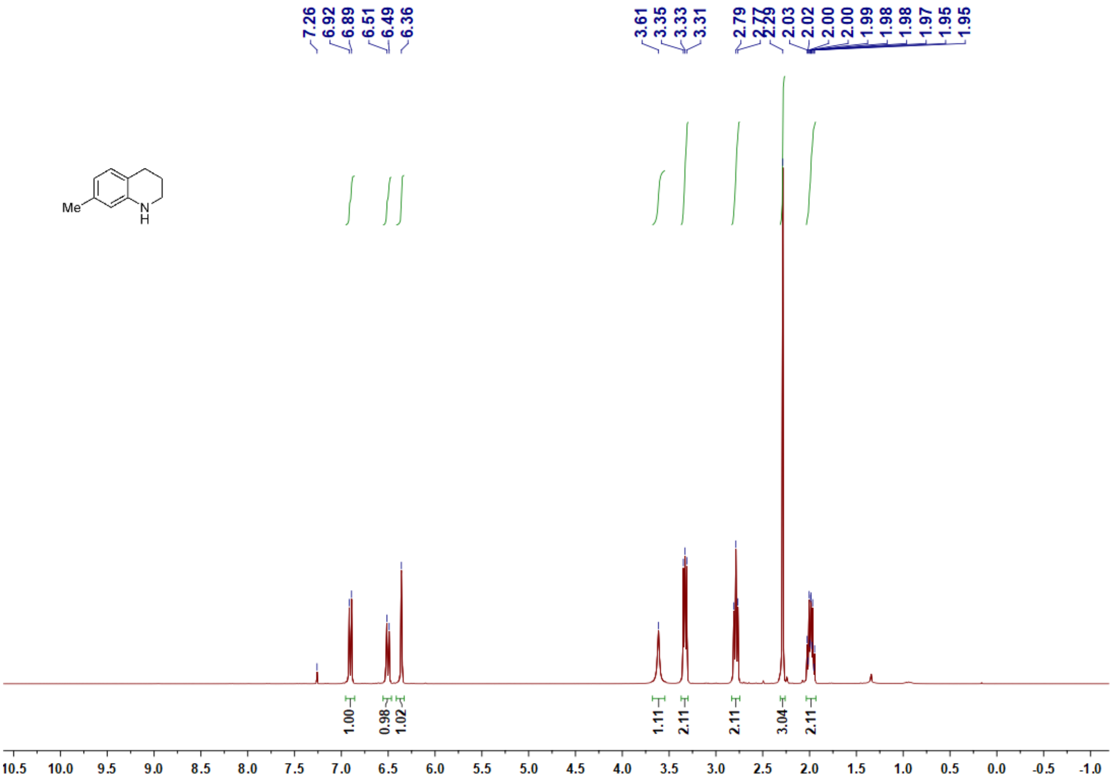

Figure S171. ^{1}H NMR (300 MHz, $CDCl_3$) spectrum of **4v**

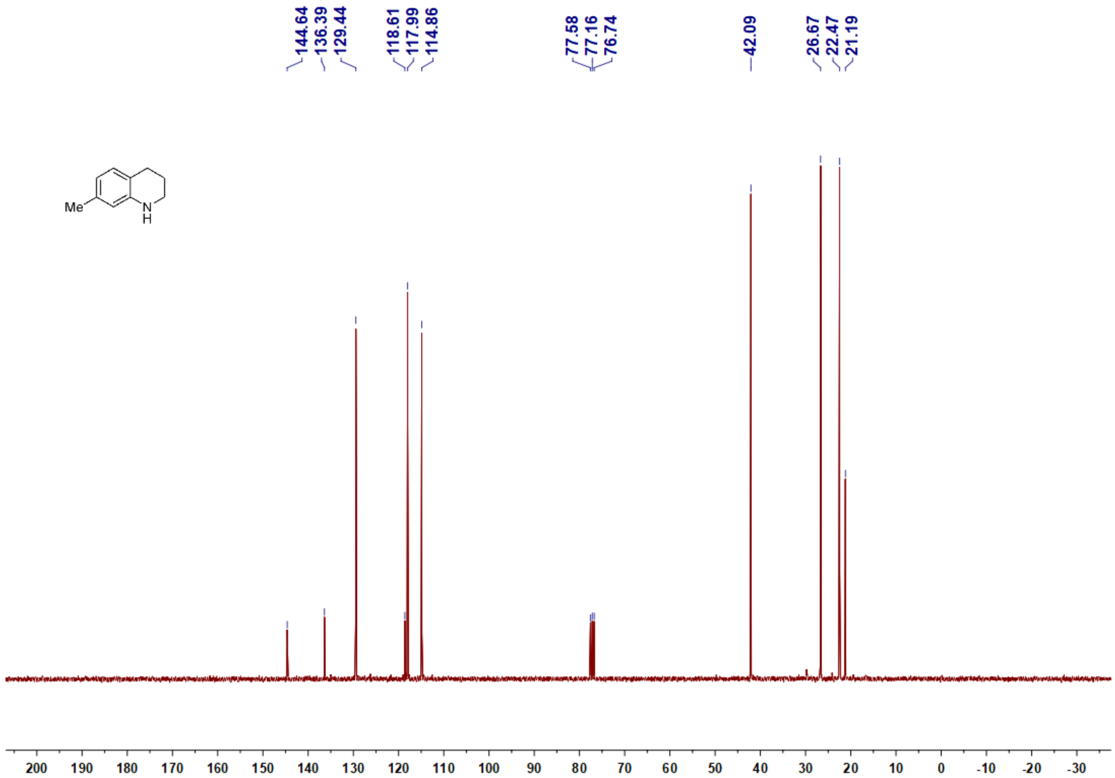

Figure S172. ^{13}C NMR (75 MHz, $CDCl_3$) spectrum of **4v**

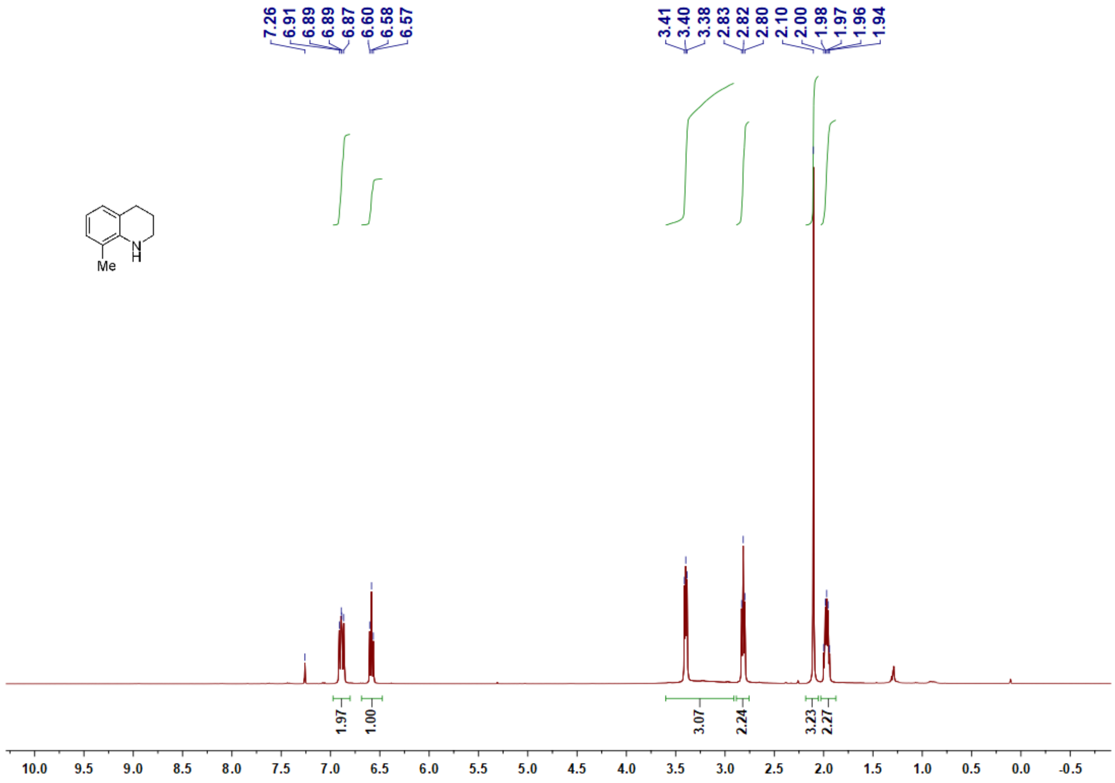

Figure S173. ^{1}H NMR (400 MHz, $CDCl_3$) spectrum of **4w**

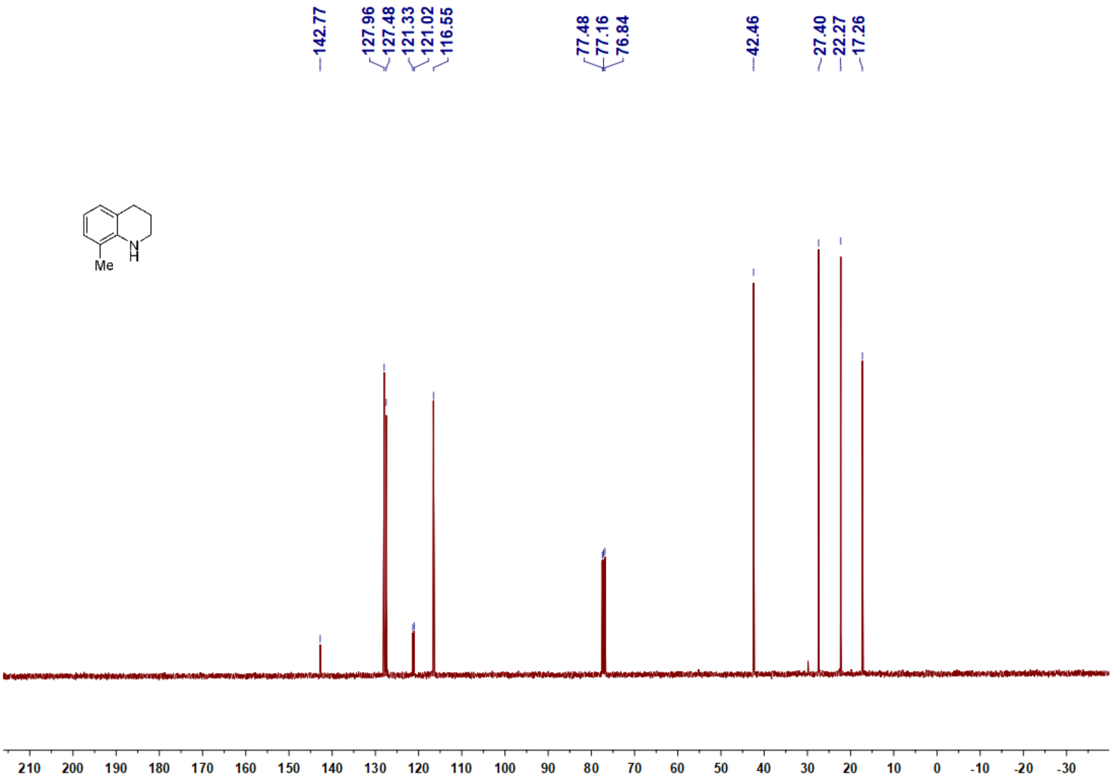

Figure S174. ^{13}C NMR (101 MHz, $CDCl_3$) spectrum of **4w**

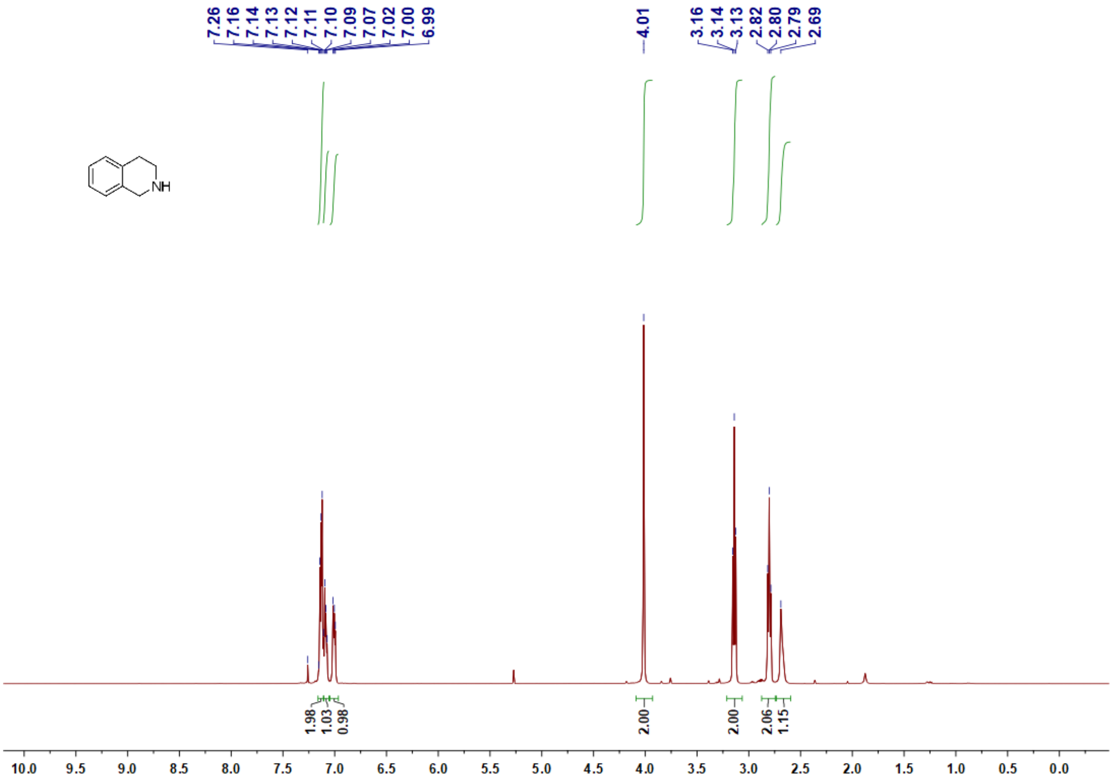

Figure S175. ^{1}H NMR (400 MHz, $CDCl_3$) spectrum of **4x**

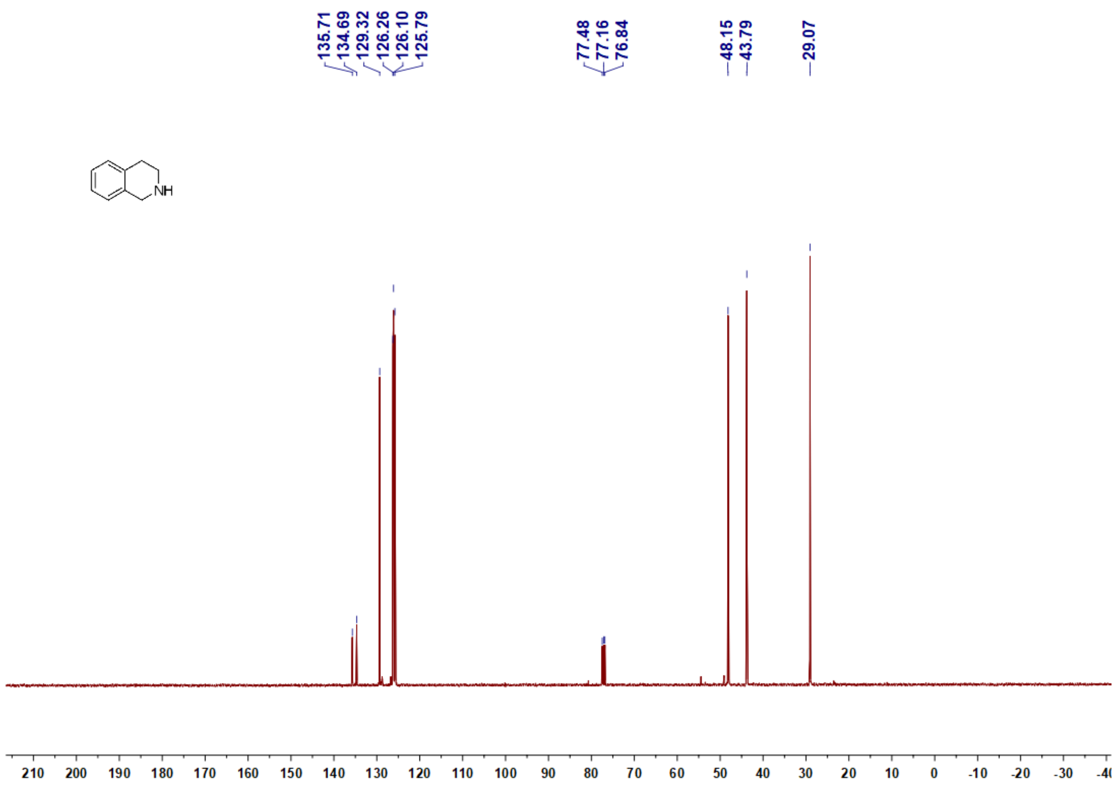

Figure S176. ^{13}C NMR (101 MHz, $CDCl_3$) spectrum of **4x**

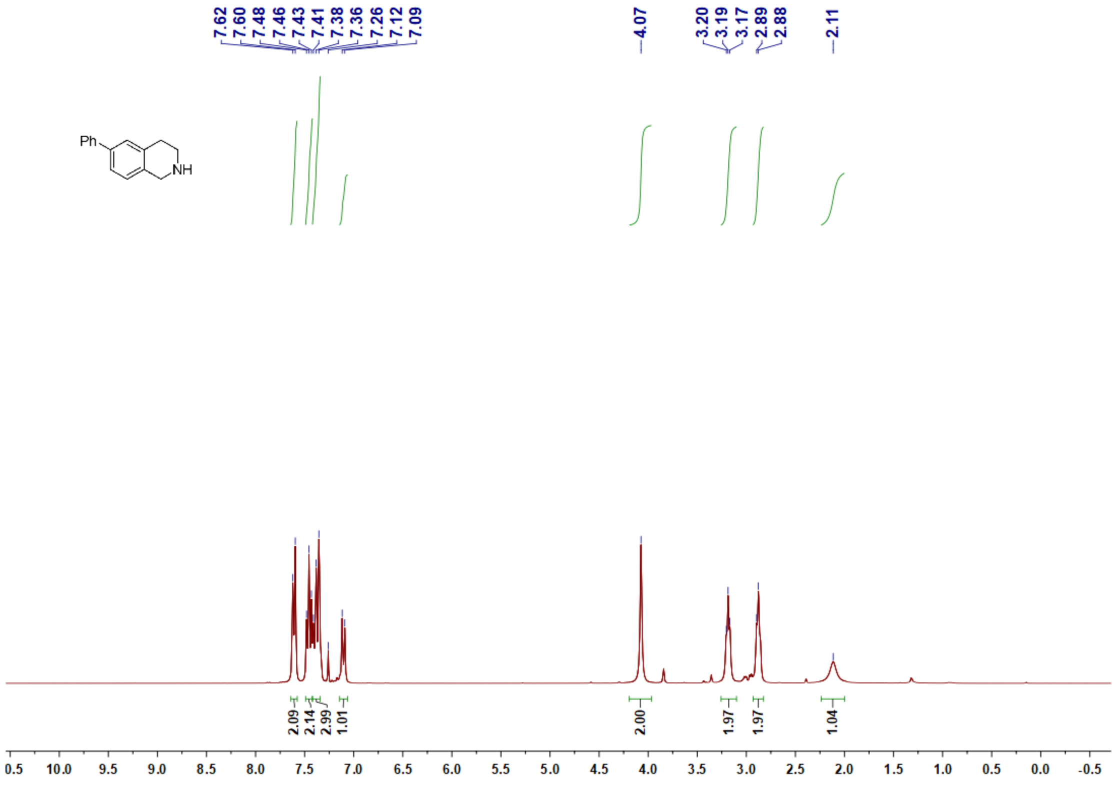

Figure S177. ^{1}H NMR (300 MHz, $CDCl_3$) spectrum of **4y**

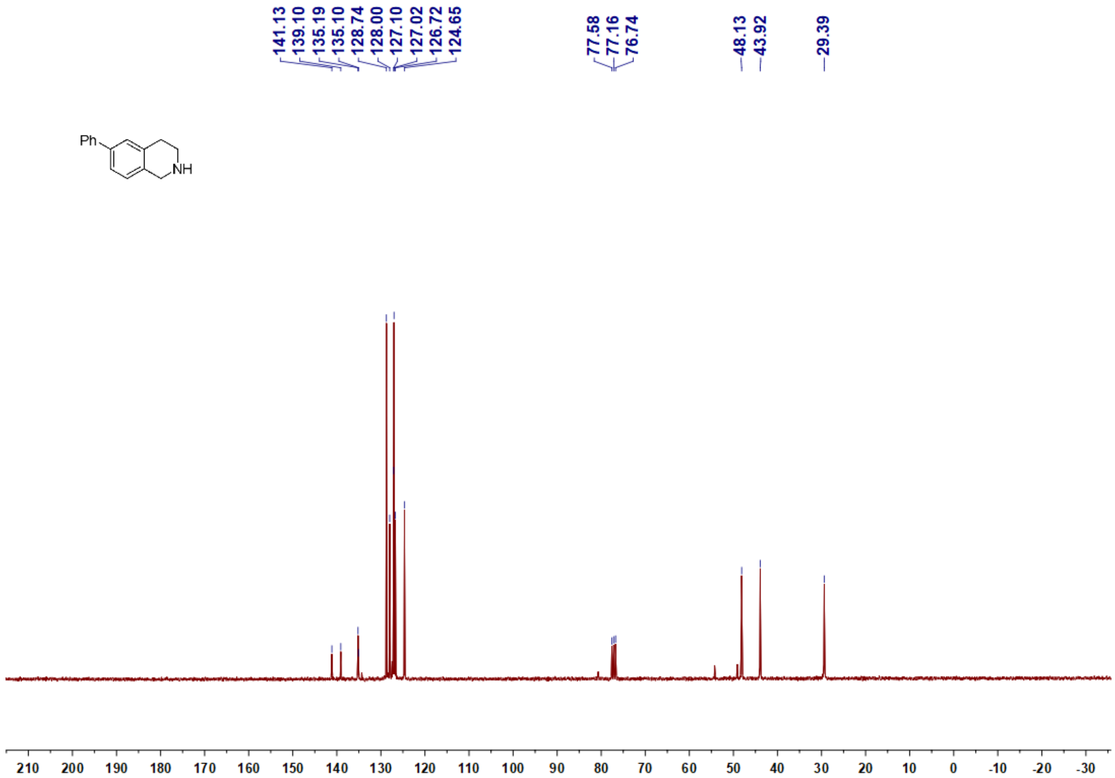

Figure S178. ^{13}C NMR (75 MHz, $CDCl_3$) spectrum of **4y**

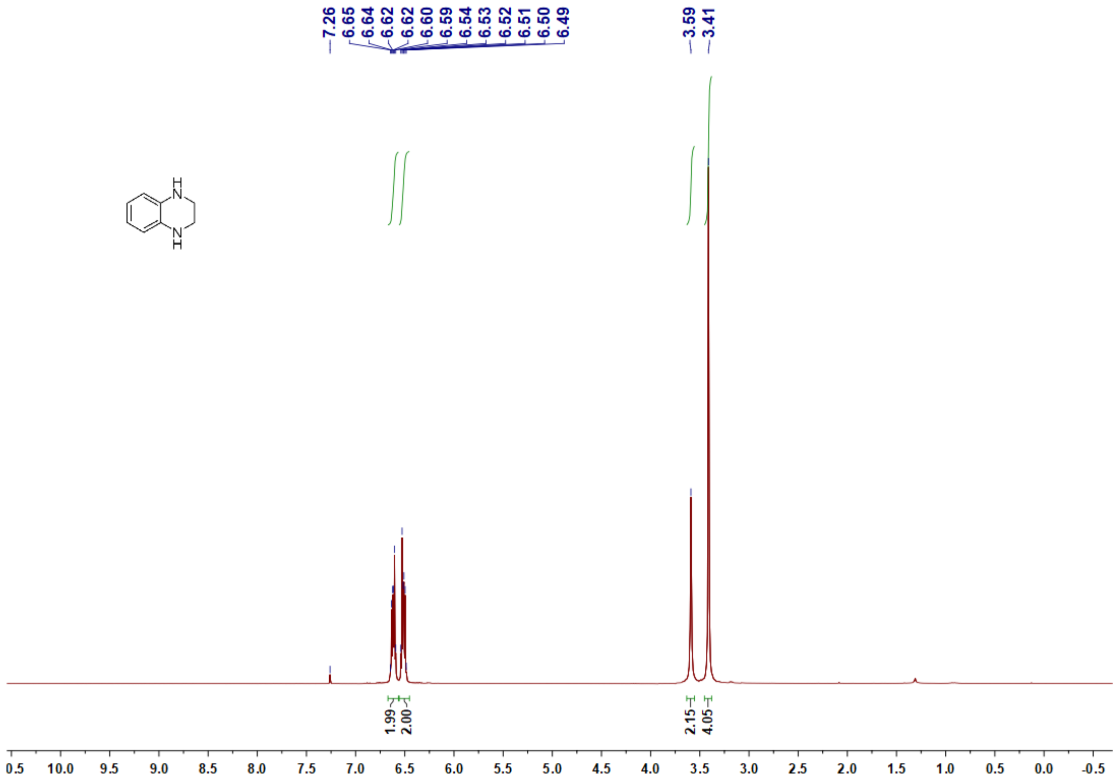

Figure S179. ^{1}H NMR (300 MHz, $CDCl_3$) spectrum of **4z**

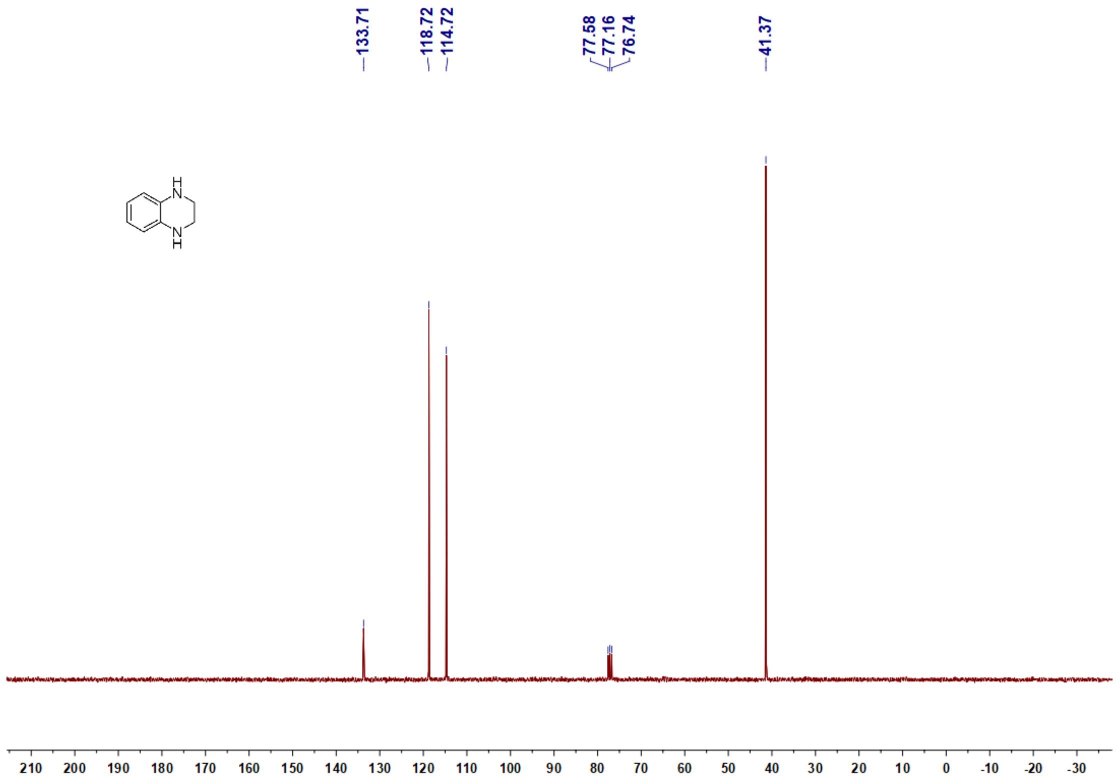

Figure S180. ^{13}C NMR (75 MHz, $CDCl_3$) spectrum of **4z**

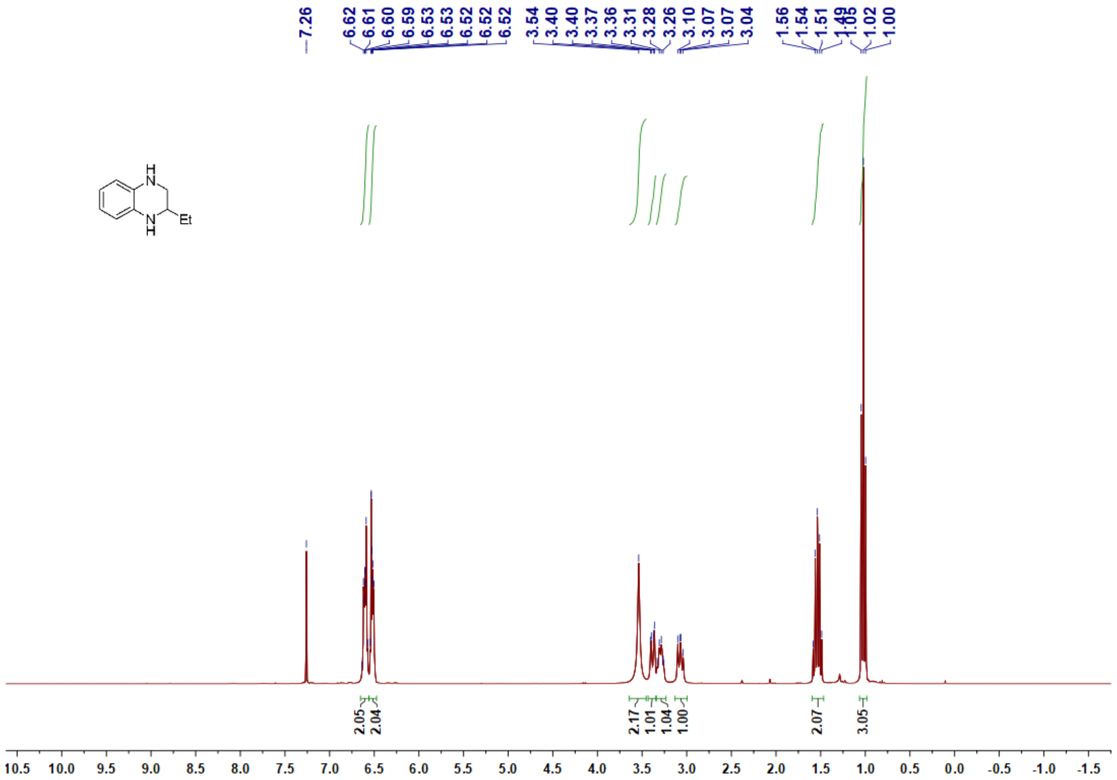

Figure S181. ^{1}H NMR (300 MHz, $CDCl_3$) spectrum of **4aa**

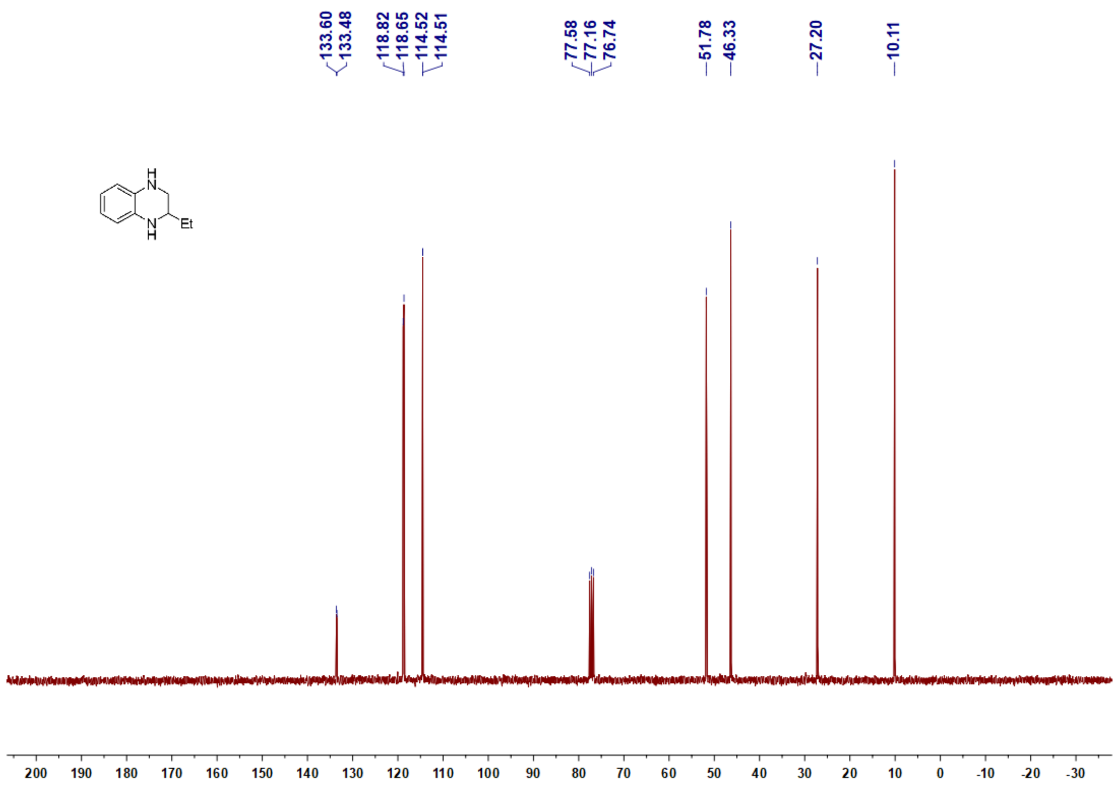

Figure S182. ^{13}C NMR (75 MHz, $CDCl_3$) spectrum of **4aa**

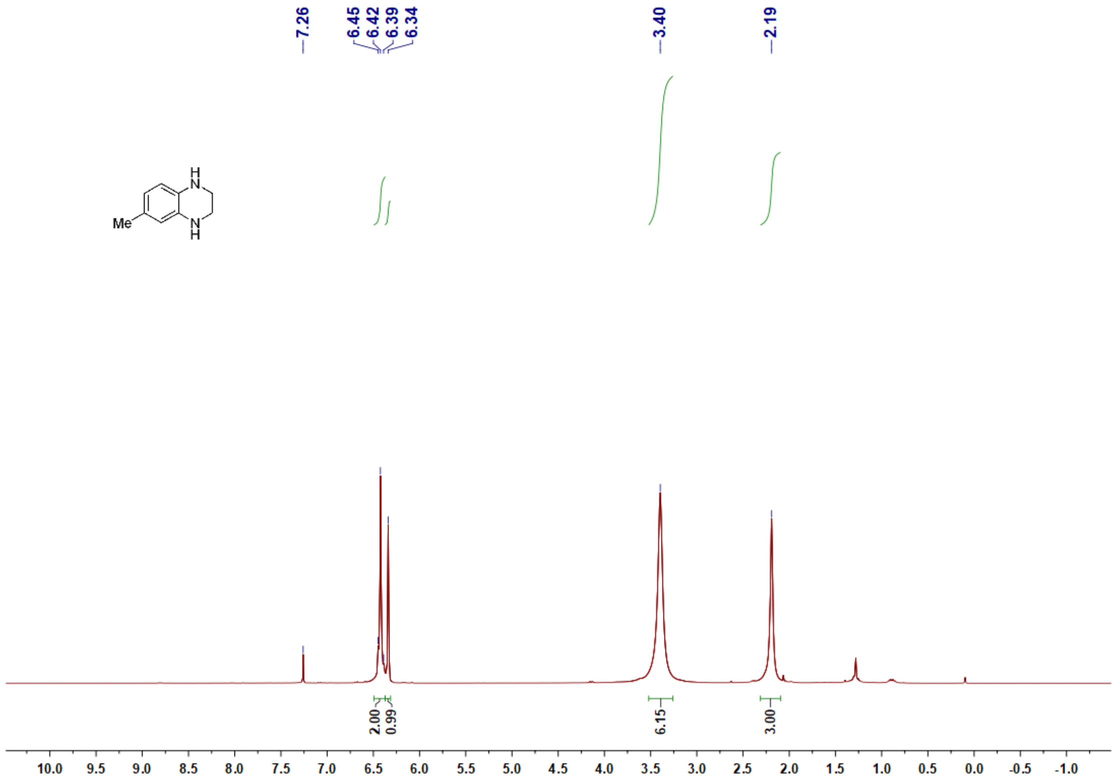

Figure S183. ^{1}H NMR (300 MHz, $CDCl_3$) spectrum of **4ab**

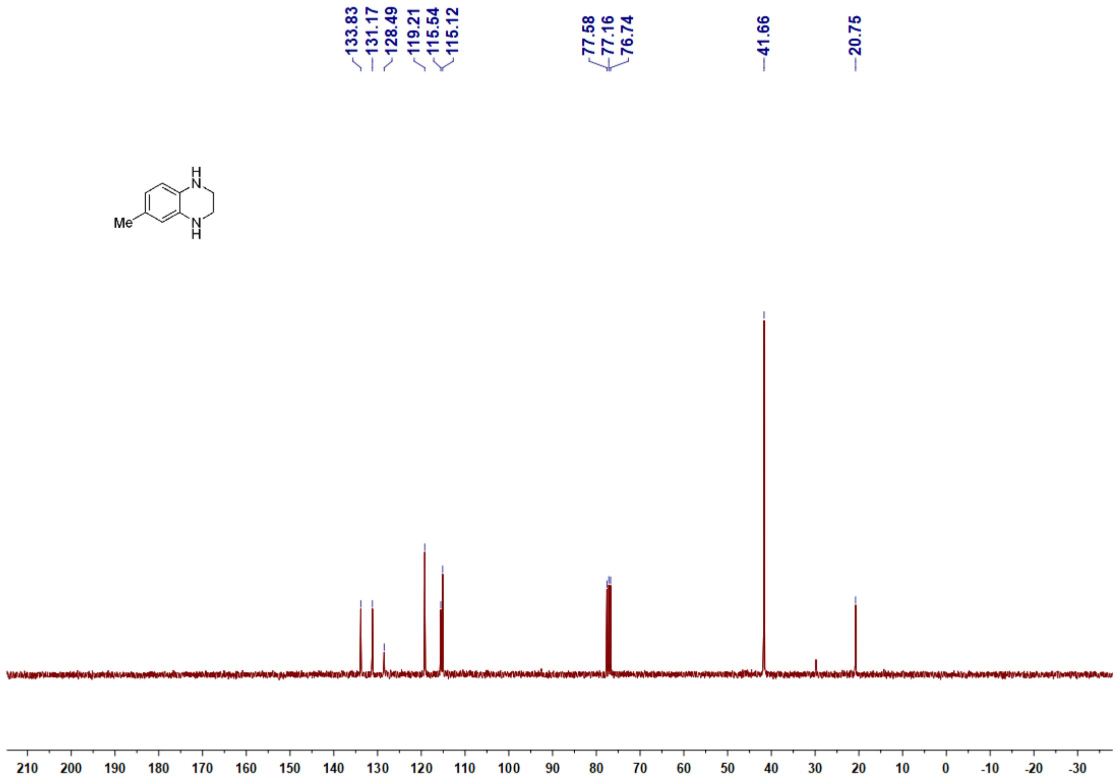

Figure S184. ^{13}C NMR (75 MHz, $CDCl_3$) spectrum of **4ab**

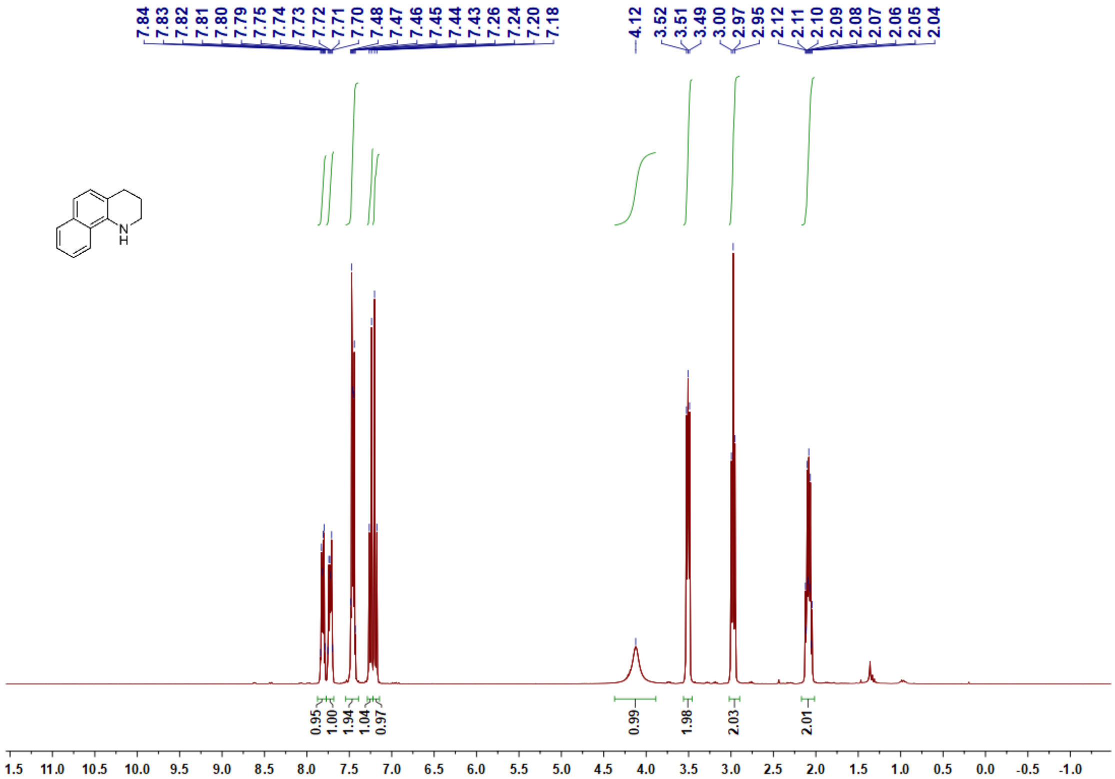

Figure S185. ^{1}H NMR (300 MHz, $CDCl_3$) spectrum of **4ac**

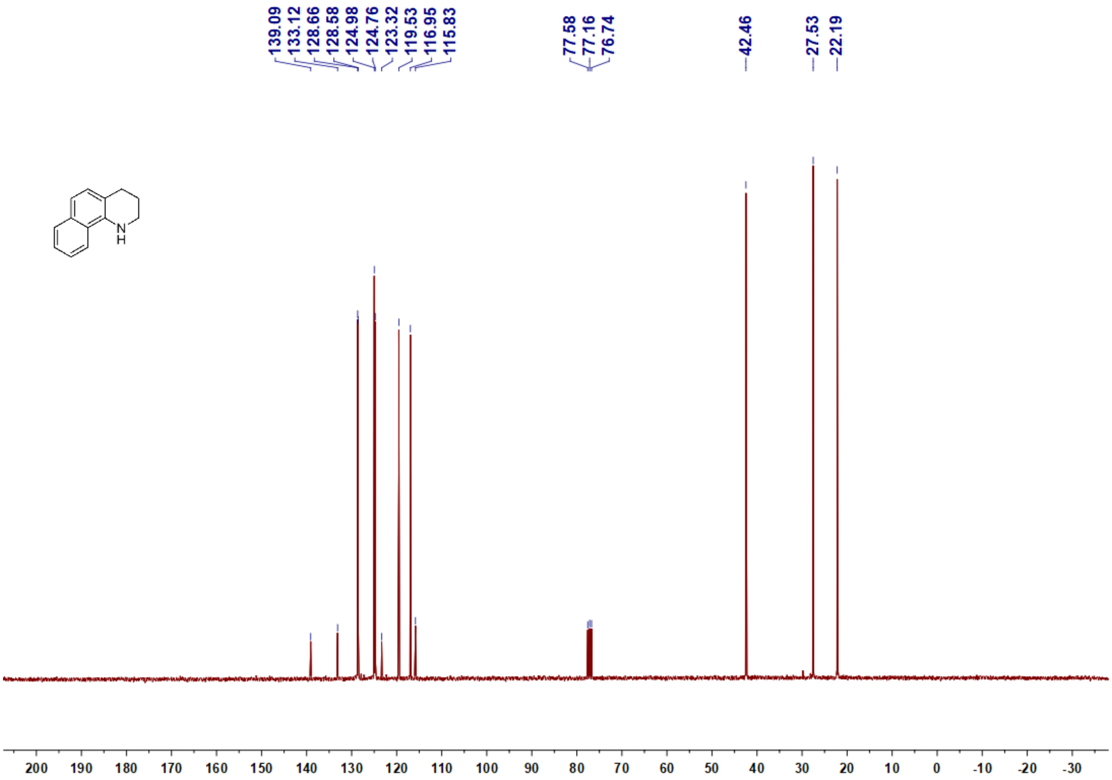

Figure S186. ^{13}C NMR (75 MHz, $CDCl_3$) spectrum of **4ac**

8 References

1. Tang, Z.; Otten, E.; Reek, J. N. H.; van der Vlugt, J. I.; de Bruin, B. Dynamic Ligand Reactivity in a Rhodium Pincer Complex. *Chem. Eur. J.* **2015**, *21*, 12683–12693.
2. Rigaku Corporation.
3. Sheldrick, G. M. *SHELXT*–Integrated Space-Group and Crystal-Structure Determinatio. *Acta Crystallogr A* **2015**, *71*, 3–8.
4. Sheldrick, G. M. Crystal Structure Refinement with *SHELXL*. *Acta Crystallogr. C* **2015**, *71*, 3–8.
5. Dolomanov, O. V.; Bourhis, L. J.; Gildea, R. J.; Howard, J. A. K.; Puschmann, H. *OLEX2*: A Complete Structure Solution, Refinement and Analysis Program. *J. Appl. Crystallogr*. **2009**, *42*, 339–341.
6. Wang, X.; Andrews, L. Infrared Spectra of Magnesium Hydride Molecules, Complexes, and Solid Magnesium Dihydride. *J. Phys. Chem. A* **2004**, *108*, 11511–11520.
7. Lawson, J. R.; Wikins, L. C.; Melen, R. L. Tris(2,4,6-trifluorophenyl)borane: An

Efficient Hydroboration Catalyst. *Chem. Eur. J.* **2017**, *23*, 10997–11000.

8. Gladfelder, J. J.; Ghosh, S.; Podunavac, M.; Cook, A. W.; Ma, Y.; Woltornist, R. A.; Keresztes, I.; Hayton, T. W.; Collum, D. B.; Zakarian, A. Enantioselective Alkylation of 2-Alkylpyridines Controlled by Organolithium Aggregation. *J. Am. Chem. Soc.* **2019**, *141*, 15024−15028.
9. Cheng, C.-C.; Yan, S.-J. The Friedländer Synthesis of Quinolines. *Org. React.* **1982**, *28*, 37−201.
10. Ma, W.; Zhang, J.; Xu, C.; Chen, F.; He, Y.-M.; Fan, Q.-H. Highly Enantioselective Direct Synthesis of Endocyclic Vicinal Diamines through Chiral Ru(diamine)-Catalyzed Hydrogenation of 2,2′-Bisquinoline Derivatives. *Angew. Chem. Int. Ed.* **2016**, *55*, 12891−12894.
11. Nishida, T.; Ida, H.; Kuninobu, Y.; Kanai, M. Regioselective Trifluoromethylation of *N*-Heteroaromatic Compounds Using Trifluoromethyldifluoroborane Activator. *Nat. Commun.* **2014**, *5*, 3387.
12. Dutta, U.; Lupton, D. W.; Maiti, D. Aryl Nitriles from Alkynes Using *tert*-Butyl Nitrite: Metal-Free Approach to C≡C Bond Cleavage. *Org. Lett.* **2016**, *18*, 860−863.
13. Bera, A.; Sk, M.; Singh, K.; Banerjee, D. Nickel-Catalysed Dehydrogenative Coupling of Aromatic Diamines with Alcohols: Selective Synthesis of Substituted Benzimidazoles and Quinoxalines. *Chem. Commun.* **2019**, *55*, 5958−5961.
14. Cramer, C. J. *Essentials of Computational Chemistry: Theories and Models in 2nd edition*; John Wiley, and Sons Ltd: West Sussex, England, 2014.
15. Sparta, M.; Riplinger, C.; Neese, F. Mechanism of Olefin Asymmetric Hydrogenation Catalyzed by Iridium Phosphino-Oxazoline: A Pair Natural Orbital Coupled Cluster Study. *J. Chem. Theory and Computation* **2014**, *10*, 1099−1108.
16. Gaussian 16, Revision C.01, Frisch, M. J.; Trucks, G. W.; H. Schlegel, B.; Scuseria, G. E.; Robb, M. A.; Cheeseman, J. R.; Scalmani, G.; Barone, V.; Petersson, G. A.; Nakatsuji, H.; Li, X.; Caricato, M.; Marenich, A. V.; Bloino, J.; Janesko, B. G.; Gomperts, R.; Mennucci, B.; Hratchian, H. P.; Ortiz, J. V.; Izmaylov, A. F.; Sonnenberg, J. L.; Williams-Young, D.; Ding, F.; Lipparini, F.; Egidi, F.; Goings, J.; Peng, B.; Petrone, A.; Henderson, T.; Ranasinghe, D.; Zakrzewski, V. G.; Gao, J.;

Rega, N.; Zheng, G.; Liang, W.; Hada, M.; Ehara, M.; Toyota, K.; Fukuda, R.; Hasegawa, J.; Ishida, M.; Nakajima, T.; Honda, Y.; Kitao, O.; Nakai, H.; Vreven, T.; Throssell, K.; Montgomery, Jr., J. A.; Peralta, J. E.; Ogliaro, F.; Bearpark, M. J.; Heyd, J. J.; Brothers, E. N.; Kudin, K. N.; Staroverov, V. N.; Keith, T. A.; Kobayashi, R.; Normand, J.; Raghavachari, K.; Rendell, A. P.; Burant, J. C.; Iyengar, S. S.; Tomasi, J.; Cossi, M.; Millam, J. M.; Klene, M.; Adamo, C.; Cammi, R.; Ochterski, J. W.; Martin, R. L.; Morokuma, K.; Farkas, O.; Foresman, J. B.; Fox, D. J. Gaussian, Inc., Wallingford CT, 2016.

17. Zhao, Y.; Truhlar, D. G. A New Local Density Functional for Main-Group Thermochemistry, Transition Metal Bonding, Thermochemical Kinetics and Noncovalent Interactions. *J. Chem. Phys*. **2006**, *125*, 194101/1−18.
18. Weigend, F.; Ahlrichs, R. Balanced Basis Sets of Split Valence, Triple Zeta Valence and Quadruple Zeta Valence Quality for H to Rn: Design and Assessment of Accuracy. *Phys. Chem. Chem. Phys.* **2005**, *7*, 3297−3305.
19. Weigend, F. Accurate Coulomb-Fitting Basis Sets for H to Rn. *Phys. Chem. Chem. Phys*. **2006**, *8*, 1057−1065.
20. Grimme, S.; Antony, J.; Ehrlich, S.; Krieg, H. A Consistent and Accurate ab Initio Parametrization of Density Functional Dispersion Correction (DFT-D) for the 94 Elements HPu. *J. Chem. Phys.* **2010**, *132*, 154104/1−19.
21. Neese, F. Software Update: the ORCA Program System, Version 4.0. *WIREs Computational Molecular Science* **2018**, *8*, e1327−e1332.
22. Mardirossian, N.; Head-Gordon, M. ωB97X-V: A 10-Parameter, Range-Separated Hybrid, Generalized Gradient Approximation Density Functional with Nonlocal Correlation, Designed by a Survival-of-the-Fittest Strategy. *Phys. Chem. Chem. Phys.* **2014**, *16*, 9904−9924.
23. Vydrova, O. A.; Voorhis, T. V. Nonlocal van der Waals Density Functional: The Simpler the Better. *J. Chem. Phys.* **2010**, *133*, 244103/1−9.
24. Hujo, W.; Grimme, S. Performance of the van der Waals Density Functional VV10 and (Hybrid)GGA Variants for Thermochemistry and Noncovalent Interactions. *J. Chem. Theory Comput.* **2011**, *7*, 3866−3871.
